# Trends and projections of caesarean section rates: global and regional estimates

**DOI:** 10.1136/bmjgh-2021-005671

**Published:** 2021-06-15

**Authors:** Ana Pilar Betran, Jiangfeng Ye, Ann-Beth Moller, João Paulo Souza, Jun Zhang

**Affiliations:** 1 UNDP/UNFPA/UNICEF/WHO/World Bank Special Programme of Research, Development and Research Training in Human Reproduction, Department of Sexual and Reproductive Health and Research, World Health Organization, Geneve, Switzerland; 2 Ministry of Education—Shanghai Key Laboratory of Children’s Environmental Health, Xinhua Hospital, Shanghai Jiao Tong University School of Medicine, Shanghai, China; 3 Department of Social Medicine, University of Sao Paulo, Sao Paulo, Brazil

**Keywords:** maternal health, public health, obstetrics, epidemiology

## Abstract

**Background:**

The caesarean section (CS) rate continues to increase across high-income, middle-income and low-income countries. We present current global and regional CS rates, trends since 1990 and projections for 2030.

**Methods:**

We obtained nationally representative data on the CS rate from countries worldwide from 1990 to 2018. We used routine health information systems reports and population-based household surveys. Using the latest available data, we calculated current regional and subregional weighted averages. We estimated trends by a piecewise analysis of CS rates at the national, regional and global levels from 1990 to 2018. We projected the CS rate and the number of CS expected in 2030 using autoregressive integrated moving-average models.

**Results:**

Latest available data (2010–2018) from 154 countries covering 94.5% of world live births shows that 21.1% of women gave birth by caesarean worldwide, averages ranging from 5% in sub-Saharan Africa to 42.8% in Latin America and the Caribbean. CS has risen in all regions since 1990. Subregions with the greatest increases were Eastern Asia, Western Asia and Northern Africa (44.9, 34.7 and 31.5 percentage point increase, respectively) while sub-Saharan Africa and Northern America (3.6 and 9.5 percentage point increase, respectively) had the lowest rise. Projections showed that by 2030, 28.5% of women worldwide will give birth by CS (38 million caesareans of which 33.5 million in LMIC annually) ranging from 7.1% in sub-Saharan Africa to 63.4% in Eastern Asia.

**Conclusion:**

The use of CS has steadily increased worldwide and will continue increasing over the current decade where both unmet need and overuse are expected to coexist. In the absence of global effective interventions to revert the trend, Southern Asia and sub-Saharan Africa will face a complex scenario with morbidity and mortality associated with the unmet need, the unsafe provision of CS and with the concomitant overuse of the surgical procedure which drains resources and adds avoidable morbidity and mortality. If the Sustainable Development Goals are to be achieved, comprehensively addressing the CS issue is a global priority.

**Key questions**

**What is already known?**

- Caesarean section (CS) rates continue to increase worldwide. There is growing international concern over the health consequences of unnecessary or unsafely conducted operations.
- Monitoring maternal health practices including the use of CS is essential to assess progress toward health goals and to provide the basis for data-driven discussions.
- Estimates and trends on CS rates have been published in 2007 and 2016 playing and important role for policy, monitoring and documenting the large variation between countries and regions.

**What are the new findings?**

- Our trend analysis confirms the increasing trend in all regions although at different pace. Worldwide, 21.1% of women give birth by CS. While in sub-Saharan Africa an average of 5% indicates underuse, the average of 42.8% in Latin America and the Caribbean is suggestive of overuse.
- The increase over the last three decades has been the largest in Eastern Asia, Western Asia and Northern Africa (44.9, 34.7 and 31.5 percentage points increase, respectively) and the least in sub-Saharan Africa with an increase of 3.6 percentage points and Northern America with 9.5 percentage points.
- Our projections suggest that, at the current pace, by 2030, 28.5% of women worldwide will give birth by CS (38 million caesareans annually) ranging from 7.1% in sub-Saharan Africa to 63.4% in Eastern Asia.

**What do the new findings imply?**

- In the absence of global effective interventions to revert the trend, regions like Southern Asia and sub-Saharan Africa will face a complex scenario with morbidity and mortality associated with the unmet need, the unsafe provision of CS and with the concomitant overuse of the surgical procedure which drains resources and adds avoidable morbidity and mortality.
- If the sustainable development goals are to be achieved within the next decade, comprehensively addressing the CS issue is a global priority.
- Given the continuing increase in the use of CS globally, the persistent inequalities among countries and the unknown consequences, routine global monitoring of CS should remain a priority to generate awareness and advocate for appropriate, evidence based, and respectful care during pregnancy and childbirth.
- Monitoring within country variation is also crucial and policy-makers should consider the use of monitoring strategies and systems such as the Robson classification to evaluate trends on CS rates and maternal and infant outcomes in a more action-oriented and meaningful manner.

**Key questions**

**What is already known?**

- Caesarean section (CS) rates continue to increase worldwide. There is growing international concern over the health consequences of unnecessary or unsafely conducted operations.
- Monitoring maternal health practices including the use of CS is essential to assess progress toward health goals and to provide the basis for data-driven discussions.
- Estimates and trends on CS rates have been published in 2007 and 2016 playing and important role for policy, monitoring and documenting the large variation between countries and regions.

**What are the new findings?**

- Our trend analysis confirms the increasing trend in all regions although at different pace. Worldwide, 21.1% of women give birth by CS. While in sub-Saharan Africa an average of 5% indicates underuse, the average of 42.8% in Latin America and the Caribbean is suggestive of overuse.
- The increase over the last three decades has been the largest in Eastern Asia, Western Asia and Northern Africa (44.9, 34.7 and 31.5 percentage points increase, respectively) and the least in sub-Saharan Africa with an increase of 3.6 percentage points and Northern America with 9.5 percentage points.
- Our projections suggest that, at the current pace, by 2030, 28.5% of women worldwide will give birth by CS (38 million caesareans annually) ranging from 7.1% in sub-Saharan Africa to 63.4% in Eastern Asia.

**What do the new findings imply?**

- In the absence of global effective interventions to revert the trend, regions like Southern Asia and sub-Saharan Africa will face a complex scenario with morbidity and mortality associated with the unmet need, the unsafe provision of CS and with the concomitant overuse of the surgical procedure which drains resources and adds avoidable morbidity and mortality.
- If the sustainable development goals are to be achieved within the next decade, comprehensively addressing the CS issue is a global priority.
- Given the continuing increase in the use of CS globally, the persistent inequalities among countries and the unknown consequences, routine global monitoring of CS should remain a priority to generate awareness and advocate for appropriate, evidence based, and respectful care during pregnancy and childbirth.
- Monitoring within country variation is also crucial and policy-makers should consider the use of monitoring strategies and systems such as the Robson classification to evaluate trends on CS rates and maternal and infant outcomes in a more action-oriented and meaningful manner.

## Introduction

Vaginal birth is a natural and physiological process. However, in certain circumstances, a caesarean section (CS) may be required to protect the woman and the baby’s health. In those circumstances, underuse of CS contributes to increased maternal and perinatal mortality and morbidity. Conversely, overuse (ie, the use of CS with no medical indication) has not shown benefits and may create harm and waste of human and financial resources.^1–3^ Thus, optimising the use of CS is of global concern and a challenge in public health.^4 5^

In this context, many countries face a double burden related to CS (ie, unmet need of CS coupled with CS provided unsafely) while others, mostly due to inequity in health, face a triple burden (ie, the double burden that affects a fraction of the population is aggravated by overuse of CS in other fraction of the population).^6 7^ Considering the prospects of significant population growth in countries affected by the double and triple burden, it could be anticipated that the overuse of CS, unsafe provision of CS and unmet need of CS may emerge as an obstacle to achieve the Sustainable Development Goals (SDGs) in 2030.

Global and regional estimates on the use of CS have been published previously to monitor improvements and changes towards global health targets.^6 8^ We set up to update these previous estimates and generate projections for 2030 within the time frame for achieving the SDGs.^9^ Based on these projections, policy-makers and other stakeholders could develop measures to mitigate the burden of unmet need of CS, overuse of CS and unsafe CS.

## Methods

### Source of caesarean section rates at national level

We defined the rate of CS as a percentage calculated by dividing the number of caesarean births over the total number of live births in a given year.^6 8^ We searched to identify national-level data on CS rates which were derived from two main sources: (1) routine health information system reports from ministries of health (MoH) or national statistics offices (NSO) and (2) population-based household surveys such as the Demographic and Health Surveys (DHS),^10^ the Multiple Indicator Cluster Surveys (MICS),^11^ Reproductive Health Surveys^12^ and other national surveys (eg, Family Health Surveys), as well as special national perinatal studies.^13^

Both MoH and NSO websites were systematically searched for all 194 WHO Member States (www.who.int/countries/en/) without any language restrictions. MoH websites were identified via Google searches applying the search terms ‘(country name) ministry of health’. NSO were identified from the UN website, which provides links to all NSOs (https://unstats.un.org/home/nso_sites/). These pages were searched using terms such as ‘annual MoH or NSO reports’, ‘annual MoH or NSO statistics’ and ‘perinatal and reproductive health statistics’. They used the website search functions for terms such as CS and abdominal delivery. Although a tool for standardised appraisal of public health information systems has not been developed and applied for high-income countries,^14^ routine data for health indicators are normally used for monitoring and international comparisons.^15^

Data from population-based household surveys were used mainly for low-income and middle-income countries where routine health information systems in some cases are not deemed reliable or complete. The DHS^10^ and the MICS^11^ are considered the best available source of several types of demographic and health indicators in these countries. During the last three decades, these surveys have been extensively used in epidemiology and health policy planning at country level.^6 8 16 17^ In both programmes, surveys are conducted by trained personnel using standardised questionnaires and rigorous methods for data collection and processing. As these surveys are typically conducted about every 5 years, comparisons over time are possible. The figures for CS rates obtained through the DHS refer to births between 3 and 5 years before the survey’s date; in the MICS, they refer to births occurring in the two previous years.

Data on CS rates at the national level was compiled from 1990 up to 2019. The final database (May 2020) for analysis included 2024 data points for 182 countries (online supplemental table 5). Countries with the latest available CS rate records in or after 2010 were included in the current global and regional estimates (n=154, coverage=94.5%); those countries with a minimum of two observed CS rate data points within the period were included in the trend analyses (n=159, coverage=96.9%).

10.1136/bmjgh-2021-005671.supp1 Supplementary data

### Global and regional estimates

The latest available data from each country was used to calculate the current global and regional CS rates. However, if the most recent available data predated 2010, then the country was excluded from this analysis because we considered it too old. Countries were grouped according to the United Nations' geographical grouping (online supplemental box 2).^18^ Regional and subregional averages for the proportion of CS were calculated as weighted means based on the country’s share of live births in 2018 in the region or subregion, respectively.^19^

‘Coverage’ was used as a measure to express how representative an estimate was regarding the region or subregion. Regional and subregional coverage were calculated as the proportion of total regional and subregional live births for which nationally representative data on CS were available. Estimates for subregions with coverage less than 60% were not calculated. We followed the Guidelines for Accurate and Transparent Health Estimates Reporting (GATHER) statement in developing the database, analysis and presentation of the study (online supplemental box 1).^20^

### Trends on caesarean section rates

We analysed the piecewise trend of CS rates at the national, regional and global levels from 1990 to 2018 in three periods: 1990–2000, 2000–2010 and 2010–2018. Two countries (Zambia and Zimbabwe) had data pertaining to 2019 and were included. Countries with a minimum of two data points (observed CS rates) within the period (1990–2018) were included in the analyses. As most countries did not have CS rate records yearly, we performed data imputation. First, we conducted a linear interpolation between available data points (observed CS rates) for each country. Second, missing values from 1990 through the first available data point and the latest available data point through 2018 were filled in using multiple imputations. A Markov chain Monte Carlo method with five imputations was performed to impute all the missing values of CS rate for each country.^21 22^ We described the CS rate changes at the regional, subregional and global levels. The CS rate changes at the national level were calculated by subtracting the earliest CS rate from the latest CS rate during each period. Regional, subregional and global averages for the CS rate changes were calculated as the weighted means of the CS rate changes at the national level using the number of live births of each country in 2005 as the weight. The method described above has been used in previous published trend analysis of CS.^6^

### Projections of caesarean section rates for 2030

We generated projections of CS rates in 2021, 2025 and 2030 to predict the trend of CS rates. Predictions were calculated using the autoregressive integrated moving-average (ARIMA) models^23^ fitted for the CS rate at the subregional level, which represented what would happen if the past decades’ CS rate trajectory continued until 2030.

The subregions were categorised into three groups based on the availability of nationally representative data on CS rates during the periods of 2010–2018, 2000–2018 or 1990–2018. Given the number of data points required for generating reliable projections of CS rates, the period of reference was determined based on the availability of nationally representative data on CS rates. In subregions with more than 80% of data on national CS rates from 2010 to 2018, we used data from 2005-2018 to fit the ARIMA models. For subregions with more than 80% of data from 2000 to 2018, but with insufficient data from 2010 to 2018, CS rates from 2000 to 2018 were included for the projection. In subregions with less than 80% of data from 2000 to 2018, the ARIMA models were fitted with data for the whole period (1990–2018).

Stationarity of the CS rate series was judged by examining the autocorrelation function plots. For non-stationary series, differencing was performed to transform it into a stationary series. The minimum information criterion, extended sample autocorrelation function and the smallest canonical correlation method were performed to identify the orders of ARIMA processes tentatively. Candidate models with the smallest BIC statistics and the residuals’ autocorrelations were non-significant at the level of 0.05 were selected. Based on the final selected models, we forecasted CS rates at subregional in 2021, 2025 and 2030. The projections at the regional and global level were calculated as weighted means based on the share of live births by subregion in the corresponding year.^23^

We used PROC MI, PROC GLIMMIX, and PROC ARIMA in SAS V.9.4 (SAS Institute) to perform the analyses. Detailed description of statistical methods and codes is available in online supplemental appendix.

## Results

### Current caesaeran section rates worldwide

A total of 154 countries with CS rate records in 2010 or later were included to describe current CS rates worldwide. This represents 94.5% of the world’s live births in 2018. The global CS rate was 21.1% with averages of 8.2%, 24.2% and 27.2% in the least, less and more developed regions, respectively (table 1). Lowest rates are found in sub-Saharan Africa (5.0%, 39 countries, 88.6% births coverage) and highest rates in Latin America and the Caribbean (42.8%, 23 countries, 91.2% births coverage). Estimates of current CS rates using data with imputation are available in the online supplemental table 1. The weighted mean of the difference between estimate CS rates at subregional level using original dataset and the imputed dataset is 2.5% (95% CI 0.1% to 4.9%). The top five countries with the highest CS rate worldwide were: Dominican Republic (58.1%), Brazil (55.7%), Cyprus (55.3%), Egypt (51.8%) and Turkey (50.8%), which also represented the highest CS rate in Americas, Asia, and Africa. In Europe, the highest CS rate was found in Romania (46.9%). The five countries with the lowest CS rate around the world belong to Africa: Chad (1.4%), Niger (1.4%), Ethiopia (1.9%), Madagascar (2%) and Cameroon (2.4%). Countries with the lowest CS rates in other regions were Timor-Leste (3.5%) in Asia, Papua New Guinea (3.0%) in Oceania, Netherlands (14.9%) in Europe and Haiti (5.4%) in Latin America (online supplemental table 5).

**Table 1:** Caesarean section (CS) rates in countries categorised according to United Nations geographical grouping in 2018*

Region/subregion	Estimated CS rate (%, 95% CI)	Range (min-max, %)	Coverage of estimates (%)
Africa (n=44)	9.2 (5.2 to 13.2)	1.4–51.8	89.9
Northern Africa (n=5)	32.0 (5.9 to 58.2)	9.1–51.8	97.8
Sub-Saharan Africa (n=39)	5.0 (3.5 to 6.6)	1.4–50.7	88.6
Asia (n=40)	23.1 (19.9 to 26.3)	3.5–55.3	96.7
Central Asia (n=5)	12.5 (6.5 to 18.4)	5.3–18	100
Eastern Asia (n=5)	33.7 (27.3 to 40.1)	12.9–39.1	100
South-eastern Asia (n=8)	15.9 (9.6 to 22.3)	3.5–32.7	95.1
Southern Asia (n=7)	19.0 (13.7 to 24.3)	6.6–40	96.3
Western Asia (n=15)	31.7 (22.7 to 40.6)	4.8–55.3	91.0
Europe (n=38)	25.7 (23.4 to 28.0)	14.9–46.9	98.8
Eastern Europe (n=10)	25.0 (18.7 to 31.3)	17.9–46.9	100
Northern Europe (n=10)	25.3 (21.5 to 29.1)	15.9–32.6	100
Southern Europe (n=11)	30.1 (27.5 to 32.7)	21.2–34.1	93.0
Western Europe (n=7)	24.2 (18.3 to 30.2)	14.9–32.7	100
Americas (n=25)	39.3 (34.6 to 44.0)	5.4–58.1	93.7
Latin America and the Caribbean (n=23)	42.8 (37.6 to 48.0)	5.4–58.1	91.2
Northern America (n=2)	31.6 (20.5 to 42.8)	28.8–31.9	100
Oceania (n=7)	21.4 (6.6 to 36.2)	3.0–34.6	96.4
Australia and New Zealand (n=2)	33.5 (1.9 to 65.1)	27.9–34.6	100
Melanesia, Micronesia, and Polynesia (n=5)	3.6 (0.7 to 6.6)	3.0–17.4	91.6
World total (n=154)	21.1 (18.8 to 23.3)	1.4–58.1	94.5
More developed countries (n=45)	27.2 (25.2 to 29.2)	14.9–55.3	99.3
Less developed countries (n=70)	24.2 (20.9 to 27.5)	2.4–58.1	94.6
Least developed countries (n=39)	8.2 (5.2 to 11.2)	1.4–32.7	92.0

*Countries with the latest CS rate record available in 2010 or late were included.

### Trends of caesaeran section rates

**Figure 1:**
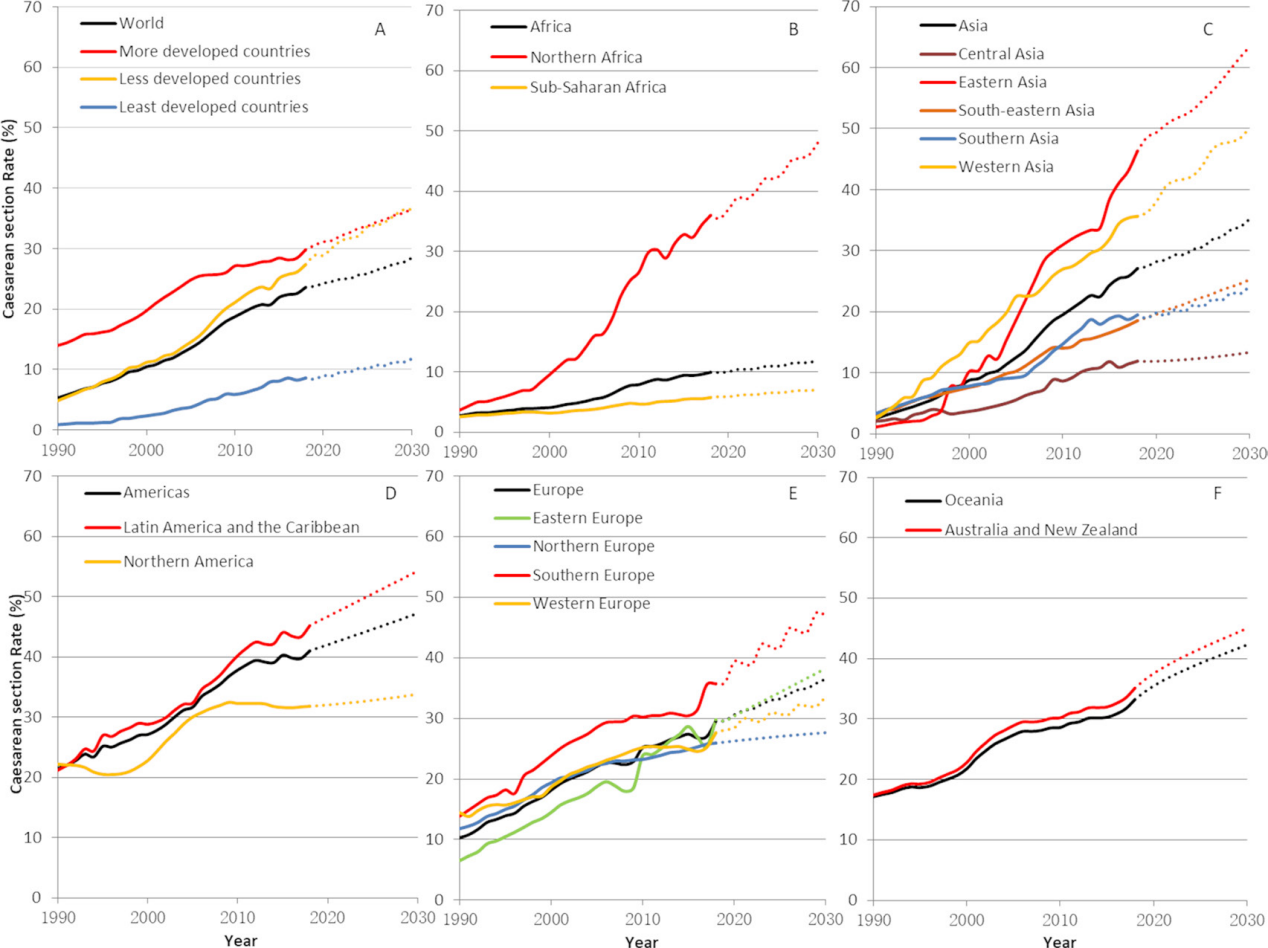
Trends (1990–2018) and projections (2030) in global, regional and subregional estimates of CS rates. Solid lines are trend estimates and dotted lines are projections. (A) World; (B) Africa; (C) Asia; (D) Americas; (E) Europe; (F) Oceania. Rates and projections for the Melanesia, Micronesia, and Polynesia were not calculated due to the low coverage of data in this subregion of Oceania.

Table 2 shows the changes in the global, regional and subregional CS rates from 1990 to 2018 based on data from 159 countries covering 96.9% of live births worldwide. Changes of CS rates in the Melanesia, Micronesia and Polynesia were not calculated due to the low data coverage in this subregion (10.5%). Figure 1 depicts the regional and subregional trends of CS rates by the UN geographical grouping (one panel for each region). During the whole period, the CS rates increased in all subregions.

**Table 2:** Caesarean section (CS) rate changes in regions/subregions from 1990 to 2018

Region/subregion*	N	Coverage (%)	Rate changes (%, 95% CI)			
			1990–2000	2000–2010	2010–2018	1990–2018
Africa	48	94.4	1.5 (0.7 to 2.4)	3.7 (1.5 to 5.8)	2.3 (1.3 to 3.3)	7.5 (3.7 to 11.3)
Northern Africa	5	97.4	6.2 (0.4 to 12.0)	16.2 (-2.2 to 34.7)	9.1 (3.3 to 14.8)	31.5 (2.6 to 60.5)
Sub-Saharan Africa	43	93.9	0.8 (0.3 to 1.3)	1.6 (1.1 to 2.2)	1.2 (0.8 to 1.6)	3.6 (2.4 to 4.8)
Asia	42	98.4	6.2 (5.1 to 7.2)	10.6 (8.1 to 13.1)	7.7 (5.9 to 9.6)	24.5 (19.5 to 29.4)
Central Asia	5	100	1.8 (0.3 to 3.2)	4.8 (2.3 to 7.2)	3.4 (2.1 to 4.8)	9.9 (5.2 to 14.6)
Eastern Asia	5	100	8.7 (5.4 to 11.9)	20.9 (11.3 to 30.4)	15.3 (9.0 to 21.6)	44.9 (28.8 to 60.9)
South-eastern Asia	8	90.1	4.5 (1.7 to 7.3)	6.7 (4.1 to 9.3)	4.5 (1.5 to 7.6)	15.8 (8.7 to 22.8)
Southern Asia	8	100	4.8 (3.3 to 6.3)	6.7 (4.2 to 9.3)	4.9 (1.9 to 7.9)	16.4 (10.9 to 21.9)
Western Asia	16	99.0	12.1 (9.0 to 15.2)	12.9 (7.2 to 18.6)	9.7 (7.3 to 12.0)	34.7 (24.0 to 45.3)
Europe	38	98.5	7.1 (6.1 to 8.1)	7.3 (5.9 to 8.7)	4.5 (3.5 to 5.5)	18.9 (16.1 to 21.8)
Eastern Europe	10	100	7.9 (5.9 to 9.8)	9.7 (6.8 to 12.7)	6.3 (4.6 to 7.9)	23.9 (17.7 to 30.0)
Northern Europe	10	100	7.4 (5.4 to 9.5)	3.8 (2.3 to 5.2)	2.8 (1.0 to 4.5)	14.0 (9.8 to 18.1)
Southern Europe	11	92.4	9.3 (7.3 to 11.2)	6.1 (3.3 to 8.9)	5.4 (3.1 to 7.7)	20.7 (16.2 to 25.3)
Western Europe	7	100	4.3 (2.9 to 5.7)	6.6 (3.3 to 9.9)	2.1 (0.1 to 4.1)	13.0 (7.1 to 18.9)
Americas	25	96.0	5.6 (3.1 to 8.2)	11.2 (9.5 to 12.8)	3.5 (1.7 to 5.3)	20.3 (15.1 to 25.5)
Latin America and the Caribbean	23	94.3	7.8 (5.2 to 10.4)	11.8 (9.9 to 13.8)	5.2 (3.5 to 6.9)	24.9 (19.5 to 30.3)
Northern America	2	100	0.5 (−14.2 to 15.2)	9.5 (−7.2 to 26.2)	−0.5 (−17.5 to 16.4)	9.5 (−5.4 to 24.5)
Oceania†	6	60.8	4.7 (1.3 to 8.2)	6.6 (2.2 to 11)	4.4 (2.2 to 6.7)	15.8 (5.9 to 25.6)
Australia and New Zealand	2	100	5.4 (−6.8 to 17.6)	7.3 (−19.7 to 34.3)	4.8 (−0.2 to 9.8)	17.5 (−26.6 to 61.7)
World total	159	96.9	5.0 (4.3 to 5.6)	8.7 (7.5 to 9.9)	5.7 (4.8 to 6.5)	19.4 (16.9 to 21.9)
More developed countries	45	99.1	4.8 (3.7 to 6.0)	7.6 (6.5 to 8.7)	2.6 (1.6 to 3.6)	15.1 (12.7 to 17.4)
Less developed countries	75	98.8	6.0 (5.0 to 7.0)	10.2 (8.3 to 12.2)	6.7 (5.4 to 8.0)	22.9 (19.1 to 26.8)
Least developed countries	39	89.5	1.4 (1.0 to 1.8)	3.7 (2.2 to 5.3)	3.4 (1.9 to 5.0)	8.6 (5.3 to 11.8)

*Countries categorised according to the UN geographical grouping.

†Changes of CS rates in Melanesia, Micronesia and Polynesia are not presented due to the low coverage in this subregion (10.5%).

Worldwide, the average CS rate increased 19 percentage points from 1990 to 2018 (table 2). The increase was largest in less developed countries (22.9 percentage points) and smallest in least developed countries (8.6 percentage points). Subregions with the greatest increases were Eastern Asia, Western Asia and Northern Africa (44.9, 34.7 and 31.5 percentage points, respectively) while sub-Saharan Africa with 3.6 percentage points of increase and Northern America with 9.5 percentage points of increase had the lowest rise. Compared with the 1990–2000 and 2010–2018, the largest increases occurred in 2000–2010. The only reduction in CS rates was observed in Northern America during the period 2010–2018 (0.5 percentage points).

Over the whole period, Turkey, Andorra and Egypt have shown an increase in CS rate of more than 50 percentage points; China, Dominican Republic, Romania, Mauritius, Georgia and Paraguay present an increase of more than 40 percentage points. Online supplemental figure 1 shows the top 10 countries with the largest increase from 1990 to 2018 and separately for each of the three periods studied (1990–2000, 2000–2010 and 2010–2018).

### Projections of caesarean section rates for 2021–2030

Projections for Northern Europe and Northern America were based on data from 2005 to 2018. Projection for Central Asia was based on data from 2000 to 2018. For all the other subregions, projections were developed based on data for the whole period (1990–2018). Online supplemental table 2 presents the number of observed CS rate data points and sampling period for forecasting in each subregion.

We estimated that the global average of the CS rate will increase from the current 21.1% to 28.5% (95% CI 23.9% to 33.1%) with more than 38 million caesarean births in 2030 (online supplemental tables 3 and 4). Nearly 80% of these CS will be conducted in less developed countries, 9.4% in least developed countries and 11.7% in more developed countries. Figure 1 depicts projections of the CS rate by region and development status for the present decade. By 2030, the CS rate will be similar in more and in less developed countries at 36.6% (95% CI 31.7% to 41.4%) and 36.5% (95% CI 32.7% to 40.3%), respectively (figure 1A). In least developed countries, the CS rate will be 11.8% (95% CI 9.7% to 13.8%). In Africa, while the Northern subregion will surge to 48.1% (95% CI 37.4% to 58.8%) CS rate in 2030, the sub-Saharan subregion will remain with the lowest rate at 7.1% (95% CI 6.4% to 7.9%) (figure 1B). Figure 1C shows that two subregions will reach the 50% threshold in Asia by 2030: Eastern and Western Asia with CS rates at 63.4% (95% CI 52.9% to 74.0%) and 50.2% (95% CI 47.4% to 52.9%). By contrast, Central Asia presents the lowest prediction in this region with a CS rate of 13.3% (95% CI 2.0% to 24.6%). Projections for the Americas showed that in Latin America and the Caribbean 54.3% (95% CI 48.3% to 60.2%) of women will give birth by CS in 2030 while the predicted 33.8% (95% CI 22.8% to 44.8%) in Northern America reveals a small change in the use of CS (figure 1D). Projections for Europe are shown in figure 1E. Highest CS rates are predicted in Southern Europe at 47% (95% CI 38.8% to 53.3%), while CS will be used in 27.6% (95% CI 16.2% to 39.1%) of the births in Northern Europe with almost no change during the current decade. The use of CS will continue to increase in Australia and New Zealand up to 45% (95% CI 38.1% to 52.0%) in 2030 (figure 1E). Projections for the Melanesia, Micronesia and Polynesia were not calculated due to the low data coverage in this subregion (10.5%).

## Discussion

Our estimates suggest that the current global CS rate is around 21%. While CS rates have steadily increased worldwide in the last three decades, sub-Saharan Africa continues to present the lowest CS rates and Latin America and the Caribbean remain at the highest. The projection of CS rates to 2021–2030 suggests that by 2030 the global CS rate will be nearing 30% with 38 million CS being performed in 2030 worldwide. Sub-Saharan Africa is expected to remain well below the 10% CS rate threshold while CS may become the most frequent mode of birth in Eastern and Western Asia, and Latin America.

Both trends and projections show a ‘two-speed growth’ in Africa which results in two different emergencies in this continent. A policy dialogue for research on determinants and for action to improve intrapartum quality of care may be prioritised in Northern Africa while in sub-Saharan Africa, international support to strengthen health systems remains essential to deliver crucial interventions.^4^ It is noteworthy that trends over last decade showed some signs of stabilisation in more developed regions such as Northern and Western Europe and North America. The underlying reasons for this trend need to be evaluated and may be complex given the multiple dimensions of the challenge. Research is needed to understand how societal, public health policies and clinical developments may have contributed to the trend.

The increase in the use of CS worldwide involves multiple factors and interactions, including women and families’ preferences, health professional’s views and beliefs, convenience, remuneration, healthcare organisation and financing structures.^4 24^ Some of these factors are country-specific, but others are universal and aligned with the values and perceptions underpinning contemporary societies. In this context, reducing overuse of CS in a sustainable manner has proven difficult to achieve and our estimates show that CS has already become the most frequent mode of birth in several countries.

Although technology has rendered CS very safe where the conditions and skills are available, this is not the case in many settings in LMIC which lack the facilities or capacity to conduct safe surgery or treat complications.^3^ In addition, the growing use of CS in human parturition has health implications, ranging from short-term health benefits in certain situations to increased morbidity and mortality, including the possibility of long-term health effects to both women and children, some of which are not fully understood.^1 2^ In the absence of global effective interventions to revert the current trend, in 2030, the CS rate in LMIC will catch up with that in more developed countries. Subregions like Northern Africa, Eastern Asia, Western Asia and Latin America will be around or beyond 50% of births by CS. Of the 38 million predicted CS in 2030, only 11.7% will occur in more developed regions with about 33.5 million CS occurring in less and least developed countries. Under current or similar care conditions, we hypothesise that the predicted number of CS in these regions will likely be associated with an important increase in maternal and perinatal morbidity and mortality.

Recognising the increasing weight of the non-medical factors in the decision-making of mode of birth, in 2018, WHO released recommendations on non-clinical interventions to reduce unnecessary use of the CS.^24 25^ Implementation and success requires time and commitment because of the multiplicity of factors involved and the inherent complexity of the interactions between them.^4^ WHO also highlights the need to tailor interventions to local determinants and consider the views and needs of all involved in the decision-making for mode of birth.^24 26–28^ Financial, regulatory and legislative interventions have emerged recently with focus on the reduction of unnecessary CS such as various payment methods for health workers or health organisations, financial incentive policies or legislatively imposed clinical guidelines.^29^ However, the evidence is inconclusive with inconsistent effects and low-quality evidence.

National CS rates and trends such as those included in this analysis are paramount for global monitoring but, as any average, they mask differences and inequalities within countries.^7^ For example, in China, large and super cities had undergone rapid increase in CS rates in 1990s and early 2000s but the CS rates in those places have actually been declining over the past decade while CS rates in rural areas have been continuously increasing.^30^ It is essential to consider the use of monitoring strategies and systems such as the Robson classification to evaluate trends of CS rates and maternal and infant outcomes in a more action-oriented and comparable manner.^5 31 32^ Despite the continuous growth of national CS rates globally, it should be noted that for a substantial proportion of the global population, CS need is frequently unmet,^7^ and no ‘optimal’ CS rate ensures that all women who need a CS do undergo the procedure.

### Strength and limitations

The present estimates and projections are based on a global, broad and systematic review of CS rates’ data covering more than 95% of live births worldwide. It builds on previous efforts, and the estimation methods have been further developed to limit imprecision. CS data derived from the sources used are considered reliable and are routinely used to conduct epidemiological analysis and monitor trends.^6 7 33^ However, a degree of caution should be exercised when considering our findings. We use various types of data sources (routine data and surveys). Most countries did not have annual records for CS rates, and interpolation was performed. If the data in 2018 was not available, the CS rate for the latest year available (in 2010 or later) was used for all subsequent years up to 2018. This conservative assumption potentially contributed to the smaller increase estimated from 2010 to 2017. This assumption coupled with the smaller number of years included in the later period (2010–2018) may have resulted in the trend towards stabilisation of CS rates observed in our estimates during this later period (eg, flattening of some CS rate curves in figure 1). If this is the case, the stabilisation of the rates could be a statistical artefact rather than the existence of a true CS rate ceiling. Lastly, our estimates do not consider a possible impact of the COVID-19 pandemic to the CS rates.

### Implications for research and practice

The expanding use of CS around the world has important implications for planning the allocation of resources and care organisation in the next decade. Understanding the future demand and the gap between what is currently offered and what is likely to happen or should happen could guide investments in research and services, capacity building and infrastructure. The evidence is building for non-medicalised birth and when possible, minimal interference with physiological processes is preferable.^34^ Acquiring a complete understanding of the long-term effects for women, children and the civilisation itself should be considered a research priority in the next decade. Addressing the CS double or triple burden that high maternal mortality countries face is essential to improve global maternal and newborn health and achieve the SDGs.

## Conclusion

Current trends and projections of CS use worldwide reveal that the present-day societies are continually moving towards medicalisation and overmedicalisation of childbirth. Southern Asia and sub-Saharan African countries face a complex scenario related to women’s mode of birth with morbidity and mortality associated with the unmet need, the unsafe provision of CS, and instances of overuse of the surgical procedure which drains resources and adds avoidable morbidity and mortality. If the SDGs are to be achieved within next decade, comprehensively addressing the CS issue is a global priority.

## Data Availability

All data relevant to the study are included in the article or uploaded as online supplemental information. No additional data.

## References

[1] Keag OE, Norman JE, Stock SJ. Long-Term risks and benefits associated with cesarean delivery for mother, baby, and subsequent pregnancies: systematic review and meta-analysis. PLoS Med 2018;15:e1002494. 10.1371/journal.pmed.1002494 29360829 PMC5779640

[2] Sandall J, Tribe RM, Avery L, et al. Short-Term and long-term effects of caesarean section on the health of women and children. Lancet 2018;392:1349–57. 10.1016/S0140-6736(18)31930-5 30322585

[3] Sobhy S, Arroyo-Manzano D, Murugesu N, et al. Maternal and perinatal mortality and complications associated with caesarean section in low-income and middle-income countries: a systematic review and meta-analysis. Lancet 2019;393:1973–82. 10.1016/S0140-6736(18)32386-9 30929893

[4] Betrán AP, Temmerman M, Kingdon C, et al. Interventions to reduce unnecessary caesarean sections in healthy women and babies. Lancet 2018;392:1358–68. 10.1016/S0140-6736(18)31927-5 30322586

[5] Betrán AP, Torloni MR, Zhang JJ, et al. Who statement on caesarean section rates. BJOG 2016;123:667–70. 10.1111/1471-0528.13526 26681211 PMC5034743

[6] Betrán AP, Ye J, Moller A-B, et al. The increasing trend in caesarean section rates: global, regional and national estimates: 1990-2014. PLoS One 2016;11:e0148343. 10.1371/journal.pone.0148343 26849801 PMC4743929

[7] Boatin AA, Schlotheuber A, Betran AP, et al. Within country inequalities in caesarean section rates: observational study of 72 low and middle income countries. BMJ 2018;360:k55. 10.1136/bmj.k55 29367432 PMC5782376

[8] Betrán AP, Merialdi M, Lauer JA, et al. Rates of caesarean section: analysis of global, regional and national estimates. Paediatr Perinat Epidemiol 2007;21:98–113. 10.1111/j.1365-3016.2007.00786.x 17302638

[9] Sustainable development goals United Nations department of economic and social Affairs, New York, 2015. Available: https://sustainabledevelopment.un.org/index.html

[10] USAID. The DHS Program - Demographic and Health Surveys, 2020. Available: http://dhsprogram.com/

[11] UNICEF. Multiple indicator cluster surveys. Available: http://mics.unicef.org/

[12] CDC. Reproductive health surveys. Atlanta, GA National Center for Chronic Disease Prevention and Health Promotion, Division of Reproductive Health; 2006. http://www.cdc.gov/reproductivehealth/global/tools/surveys.htm

[13] EURO-PERISTAT Project. European perinatal health report; 2008.

[14] Chen H, Yu P, Hailey D, et al. Methods for assessing the quality of data in public health information systems: a critical review. Stud Health Technol Inform 2014;204:13–18. 25087521

[15] WHO. Trends in maternal mortality 2000 to 2017: estimates by WHO, UNICEF, UNFPA, World bank group and the United Nations population division. Licence: CC BY-NC-SA 3.0 IGO. Geneva World Health Organization; 2019.

[16] Stanton CK, Dubourg D, De Brouwere V, et al. Reliability of data on caesarean sections in developing countries. Bull World Health Organ 2005;83:449–55. doi:/S0042-96862005000600013 15976896 PMC2626266

[17] Marsh AD, Muzigaba M, Diaz T, et al. Effective coverage measurement in maternal, newborn, child, and adolescent health and nutrition: progress, future prospects, and implications for quality health systems. Lancet Glob Health 2020;8:e730–6. 10.1016/S2214-109X(20)30104-2 32353320 PMC7196884

[18] United Nationas Statistics Division. Geographic regions, 2021. Available: https://unstats.un.org/unsd/methodology/m49/

[19] United Nations, Population Division, Department of Economic and Social Affairs. World population prospects 2019. New York; 2019.

[20] Stevens GA, Alkema L, Black RE, et al. Guidelines for accurate and transparent health estimates reporting: the gather statement. Lancet 2016;388:e19–23. 10.1016/S0140-6736(16)30388-9 27371184

[21] Schafer JL. Multiple imputation: a primer. Stat Methods Med Res 1999;8:3–15. 10.1177/096228029900800102 10347857

[22] SAS Institute Inc. SAS／STAT 9 User’s Guide. North Carolina, US SAS lnstitute Inc; 2003.

[23] SAS Institute Inc. SAS/ETS® 14.3 User’s Guide. North Carolina, US SAS Institute Inc; 2017.

[24] Opiyo N, Kingdon C, Oladapo OT, et al. Non-Clinical interventions to reduce unnecessary caesarean sections: WHO recommendations. Bull World Health Organ 2020;98:66–8. 10.2471/BLT.19.236729 31902964 PMC6933434

[25] Chen I, Opiyo N, Tavender E, et al. Non-Clinical interventions for reducing unnecessary caesarean section. Cochrane Database Syst Rev 2018;9:CD005528. 10.1002/14651858.CD005528.pub3 30264405 PMC6513634

[26] Kingdon C, Downe S, Betrán AP. Women's and communities' views of targeted educational interventions to reduce unnecessary caesarean section: a qualitative evidence synthesis. Reprod Health 2018;15:130. 10.1186/s12978-018-0570-z 30041661 PMC6057083

[27] Kingdon C, Downe S, Betrán AP. Interventions targeted at health professionals to reduce unnecessary caesarean sections: a qualitative evidence synthesis. BMJ Open 2018;8:e025073. 10.1136/bmjopen-2018-025073 PMC6303601 30559163

[28] Kingdon C, Downe S, Betrán AP. Non-Clinical interventions to reduce unnecessary caesarean section targeted at organisations, facilities and systems: systematic review of qualitative studies. PLoS One 2018;13:e0203274. 10.1371/journal.pone.0203274 30180198 PMC6122831

[29] Opiyo N, Young C, Requejo JH, et al. Reducing unnecessary caesarean sections: scoping review of financial and regulatory interventions. Reprod Health 2020;17:133. 10.1186/s12978-020-00983-y 32867791 PMC7457477

[30] Li H-T, Luo S, Trasande L, et al. Geographic variations and temporal trends in cesarean delivery rates in China, 2008-2014. JAMA 2017;317:69–76. 10.1001/jama.2016.18663 28030701

[31] Torloni MR, Betran AP, Souza JP, et al. Classifications for cesarean section: a systematic review. PLoS One 2011;6:e14566. 10.1371/journal.pone.0014566 21283801 PMC3024323

[32] Betrán AP, Vindevoghel N, Souza JP, et al. A systematic review of the Robson classification for caesarean section: what works, doesn't work and how to improve it. PLoS One 2014;9:e97769. 10.1371/journal.pone.0097769 24892928 PMC4043665

[33] Betran AP, Torloni MR, Zhang J, et al. What is the optimal rate of caesarean section at population level? A systematic review of ecologic studies. Reprod Health 2015;12:57. 10.1186/s12978-015-0043-6 26093498 PMC4496821

[34] WHO. WHO recommendations: intrapartum care for a positive childbirth experience. Geneva, Switzerland World Health Organization; 2018. 30070803

