# Supplementary material for: Trends and projections of caesarean section rates: global and regional estimates
Source: BMJ Glob Health. 2021 Jun 15;6(6):e005671. doi: 10.1136/bmjgh-2021-005671 (PMC8208001; doi:10.1136/bmjgh-2021-005671)
Supplement: Supplementary data [file bmjgh-2021-005671supp001.pdf]

Trends and projections of caesarean section rates: global and regional estimates

Appendix

Supplementary material

Betrán AP, Ye J, Moller AB,1 MSc, Souza JP, Zhang JJ.

Contents

1. GATHER Statement 2
2. United Nations Population Division (UNPD) regions and subregions 3
3. Detailed description of statistical methods and codes 5
4. Supplementary tables and other results 21
5. Data points included in the analysis by country and year and source of data 29

1. GATHER Statement

Supplementary box 1: GATHER Statement.

Item #	Checklist item	Reported on page #
**Objectives and funding**		
**1**	Define the indicator(s), populations (including age, sex, and geographic entities), and time period(s) for which estimates were made.	4-6
**2**	List the funding sources for the work.	12
**Data Inputs**		
For all data inputs from multiple sources that are synthesized as part of the study:		
**3**	Describe how the data were identified and how the data were accessed.	4-5
**4**	Specify the inclusion and exclusion criteria. Identify all ad-hoc exclusions.	4-6
**5**	Provide information on all included data sources and their main characteristics. For each data source used, report reference information or contact name/institution, population represented, data collection method, year(s) of data collection, sex and age range, diagnostic criteria or measurement method, and sample size, as relevant.	Appendix pp 29-282
**6**	Identify and describe any categories of input data that have potentially important biases (e.g., based on characteristics listed in item 5).	NA
For data inputs that contribute to the analysis but were not synthesized as part of the study:		
**7**	Describe and give sources for any other data inputs.	5-6
For all data inputs:		
**8**	Provide all data inputs in a file format from which data can be efficiently extracted (e.g., a spreadsheet rather than a PDF), including all relevant meta-data listed in item 5. For any data inputs that cannot be shared because of ethical or legal reasons, such as third-party ownership, provide a contact name or the name of the institution that retains the right to the data.	Appendix pp 29-282
**Data analysis**		
**9**	Provide a conceptual overview of the data analysis method. A diagram may be helpful.	5-7, Appendix pp 5-20
**10**	Provide a detailed description of all steps of the analysis, including mathematical formulae. This description should cover, as relevant, data cleaning, data pre-processing, data adjustments and weighting of data sources, and mathematical or statistical model(s).	Appendix pp 5-20
**11**	Describe how candidate models were evaluated and how the final model(s) were selected.	Appendix pp 5-20
**12**	Provide the results of an evaluation of model performance, if done, as well as the results of any relevant sensitivity analysis.	Appendix pp 5-20
**13**	Describe methods for calculating uncertainty of the estimates. State which sources of uncertainty were, and were not, accounted for in the uncertainty analysis.	5-7
**14**	State how analytic or statistical source code used to generate estimates can be accessed.	Appendix pp 5-20
**Results and Discussion**		
**15**	Provide published estimates in a file format from which data can be efficiently extracted.	14-15, appendix pp 21-28
**16**	Report a quantitative measure of the uncertainty of the estimates (e.g. uncertainty intervals).	14-15 appendix pp 21-28
**17**	Interpret results in light of existing evidence. If updating a previous set of estimates, describe the reasons for changes in estimates.	10-11
**18**	Discuss limitations of the estimates. Include a discussion of any modelling assumptions or data limitations that affect interpretation of the estimates.	11-12

Source: http://gather-statement.org/.

2. United Nations Population Division (UNPD) regions and subregions

Supplementary box 2: United Nations Population Division (UNDP) regions and subregions.

Regions	Countries
**Africa**	
Northern Africa	Algeria, Egypt, Morocco, State of Libya, Sudan, Tunisia.
Sub-Saharan Africa	Angola, Benin, Botswana, Burkina Faso, Burundi, Cabo Verde, Cameroon, Central African Republic, Chad, Comoros, Congo, Côte d'Ivoire, Democratic Republic of the Congo, Djibouti, Equatorial Guinea, Eritrea, Ethiopia, Gabon, Gambia, Ghana, Guinea, Guinea-Bissau, Kenya, Lesotho, Liberia, Madagascar, Malawi, Mali, Mauritania, Mauritius, Mozambique, Namibia, Niger, Nigeria, Rwanda, Sao Tome and Principe, Senegal, Seychelles, Sierra Leone, Somalia, South Africa, South Sudan, Eswatini, Togo, Uganda, United Republic of Tanzania, Zambia, Zimbabwe.
**Asia**	
Central Asia	Kazakhstan, Kyrgyzstan, Tajikistan, Turkmenistan, Uzbekistan.
Eastern Asia	China, Democratic People's Republic of Korea, Japan, Mongolia, Republic of Korea.
South-eastern Asia	Brunei Darussalam, Cambodia, Indonesia, Lao People's Democratic Republic, Malaysia, Myanmar, Philippines, Singapore, Thailand, Timor-Leste, Viet Nam.
Southern Asia	Afghanistan, Bangladesh, Bhutan, India, Iran (Islamic Republic of), Maldives, Nepal, Pakistan, Sri Lanka.
Western Asia	Armenia, Azerbaijan, Bahrain, Cyprus, Georgia, Iraq, Israel, Jordan, Kuwait, Lebanon, Oman, Qatar, Saudi Arabia, Syrian Arab Republic, Turkey, United Arab Emirates, Yemen.
**Europe**	
Eastern Europe	Belarus, Bulgaria, Czechia, Hungary, Poland, Republic of Moldova, Romania, Russian Federation, Slovakia, Ukraine.
Northern Europe	Denmark, Estonia, Finland, Iceland, Ireland, Latvia, Lithuania, Norway, Sweden, United Kingdom of Great Britain and Northern Ireland.
Southern Europe	Albania, Bosnia and Herzegovina, Croatia, Greece, Italy, Malta, Montenegro, Portugal, Republic of North Macedonia, Serbia, Slovenia, Spain.
Western Europe	Austria, Belgium, France, Germany, Luxembourg, Netherlands, Switzerland.
**Americas**	
Latin America and the Caribbean	Antigua and Barbuda, Argentina, Bahamas, Barbados, Belize, Bolivia (Plurinational State of), Brazil, Chile, Colombia, Costa Rica, Cuba, Dominica, Dominican Republic, Ecuador, El Salvador, Grenada, Guatemala, Guyana, Haiti, Honduras, Jamaica, Mexico, Nicaragua, Panama, Paraguay, Peru, Saint Kitts and Nevis, Saint Lucia, Saint Vincent and the Grenadines, Suriname, Trinidad and Tobago, Uruguay, Venezuela (Bolivarian Republic of).
Northern America	Canada, United States of America
**Oceania**	
Australia and New Zealand	Australia and New Zealand
Melanesia, Micronesia, and Polynesia	Fiji, Kiribati, Micronesia (Federated States of), Papua New Guinea, Samoa, Solomon Islands, Tonga, Vanuatu.

Betran AP, *et al. BMJ Global Health* 2021; 6:e005671. doi: 10.1136/bmjgh-2021-005671

Regions	Countries
**More developed countries**	Albania, Australia, Austria, Belarus, Belgium, Bosnia and Herzegovina, Bulgaria, Canada, Croatia, Czechia, Denmark, Estonia, Finland, France, Germany, Greece, Hungary, Iceland, Ireland, Italy, Japan, Latvia, Lithuania, Luxembourg, Malta, Montenegro, Netherlands, New Zealand, Norway, Poland, Portugal, Republic of Moldova, Republic of North Macedonia, Romania, Russian Federation, Serbia, Slovakia, Slovenia, Spain, Sweden, Switzerland, Ukraine, United Kingdom of Great Britain and Northern Ireland.
**Less developed countries**	Afghanistan, Algeria, Angola, Antigua and Barbuda, Argentina, Armenia, Azerbaijan, Bahamas, Bahrain, Bangladesh, Barbados, Belize, Benin, Bhutan, Bolivia (Plurinational State of), Botswana, Brazil, Brunei Darussalam, Burkina Faso, Burundi, Cabo Verde, Cambodia, Cameroon, Central African Republic, Chad, Chile, China, Colombia, Comoros, Congo, Costa Rica, Côte d'Ivoire, Cuba, Cyprus, Democratic People's Republic of Korea, Democratic Republic of the Congo, Djibouti, Dominican Republic, Ecuador, Egypt, El Salvador, Equatorial Guinea, Eritrea, Ethiopia, Eswatini, Fiji, Gabon, Gambia, Georgia, Ghana, Grenada, Guatemala, Guinea, Guinea-Bissau, Guyana, Haiti, Honduras, India, Indonesia, Iran (Islamic Republic of), Iraq, Israel, Jamaica, Jordan, Kazakhstan, Kenya, Kiribati, Kuwait, Kyrgyzstan, Lao People's Democratic Republic, Lebanon, Lesotho, Liberia, State of Libya, Madagascar, Malawi, Malaysia, Maldives, Mali, Mauritania, Mauritius, Mexico, Micronesia (Federated States of), Mongolia, Morocco, Mozambique, Myanmar, Namibia, Nepal, Nicaragua, Niger, Nigeria, Oman, Pakistan, Panama, Papua New Guinea, Paraguay, Peru, Philippines, Puerto Rico, Qatar, Republic of Korea, Rwanda, Saint Lucia, Saint Vincent and the Grenadines, Samoa, Sao Tome and Principe, Saudi Arabia, Senegal, Seychelles, Sierra Leone, Singapore, Solomon Islands, Somalia, South Africa, South Sudan, Sri Lanka, Sudan, Suriname, Syrian Arab Republic, Tajikistan, Thailand, Timor-Leste, Togo, Tonga, Trinidad and Tobago, Tunisia, Turkey, Turkmenistan, Uganda, United Arab Emirates, United Republic of Tanzania, Uruguay, Uzbekistan, Vanuatu, Venezuela (Bolivarian Republic of), Viet Nam, Yemen, Zambia, Zimbabwe.
**Least developed countries**	Afghanistan, Angola, Bangladesh, Benin, Bhutan, Burkina Faso, Burundi, Cambodia, Central African Republic, Chad, Comoros, Democratic Republic of the Congo, Djibouti, Eritrea, Ethiopia, Gambia, Guinea, Guinea-Bissau, Haiti, Kiribati, Lao People’s Democratic Republic, Lesotho, Liberia, Madagascar, Malawi, Mali, Mauritania, Mozambique, Myanmar, Nepal, Niger, Rwanda, Sao Tome and Principe, Senegal, Sierra Leone, Solomon Islands, Somalia, South Sudan, Sudan, Timor-Leste, Togo, Uganda, United Republic of Tanzania, Vanuatu, Yemen, Zambia.

Source: UNPD region (https://unstats.un.org/unsd/methodology/m49/).

3. Detailed description of statistical methods and codes

Statistical methods

Data imputation
As most countries did not have CS rate records yearly, we performed data imputation. First, we conducted a linear interpolation between available data points (observed CS rates) for each country. Second, missing values from 1990 through the first available data point and the latest available data point through 2018 were filled in using multiple imputations. A Markov chain Monte Carlo (MCMC) method with five imputations was performed to impute all the missing values of CS rate for each country.

Current global and regional caesarean section rates estimates
The latest available data from each country was used to calculate the current global and regional CS rates. Countries with the most recent available data in or after 2010 were included in this analysis. Countries were grouped according to the United Nations' geographical grouping. Regional and sub-regional averages for the proportion of CS were calculated as weighted means based on the country's share of live births in 2018 in the region or sub-region, respectively [using the original (Table 1) and imputed dataset (Appendixt Table 1), respectively]. The average difference between CS rate estimates using the original and the imputed datasets was calculated as the weighted mean and 95% confidence intervals of the difference between the estimate CS rates at subregional level using the two datasets respectively.

"Coverage" was used as a measure to express how representative an estimate was regarding the region or sub-region. Regional and sub-regional coverage were calculated as the proportion of total regional and sub-regional live births for which nationally representative data on CS were available. Estimates for sub-regions with coverage less than 60% were not calculated.

Trends on caesarean section rates
We analyzed the piecewise trend of CS rates at the national, regional and global levels from 1990 to 2018 in three periods: 1990 to 2000, 2000 to 2010, and 2010 to 2018. Countries with a minimum of two data points (observed CS rates) within the period (1990 – 2018) were included in the analyses. We described the CS rate changes at the national, regional, sub-regional, and global levels using the imputed dataset. The CS rate changes at the national level were calculated by subtracting the earliest CS rate from the latest CS rate during each period. Regional, sub-regional and global averages for the CS rate changes were calculated as the weighted means of the CS rate changes at the national level using the number of live births of each country in 2005 as the weight.

Projections of caesarean section rates for 2030
We generated projections of CS rates in 2021, 2025 and 2030 to predict the trend of CS rates. Predictions were calculated using the autoregressive integrated moving-average (ARIMA) models fitted for the CS rate at the sub-regional level, which represented what would happen if the past decades' CS rate trajectory continued until 2030. The sub-regions were categorized into three groups based on the availability of nationally representative data on CS rates during the periods of 2010-2018, 2000-2018, or 1990-2018. Given the number of data points required for generating reliable projections of CS rates, the period of reference was determined based on the availability of nationally representative data on CS rates. Sub-regions with more than 80% of data on nationally CS rates from 2010 to 2018, we used data from this period to fit the ARIMA models. Sub-regions with more than 80% of data from 2000 to 2018, but with insufficient data from 2010 to 2018, CS rates from 2000 to 2018 were included for the projection. Sub-regions with less than 80% of data from 2000 to 2018, the ARIMA models were fitted with data for the whole period (1990-2018).

Stationarity of the CS rate series was judged by examining the autocorrelation function plots (ACF). For non-stationary series, differencing was performed to transform it into a stationary series. The minimum information criterion (MINIC), extended sample autocorrelation function (ESACF) and the smallest canonical (SCAN) correlation method were performed to identify the orders of ARIMA processes tentatively. Candidate models with the smallest BIC statistics and the residuals' autocorrelations were non-significant at the level of 0.05 were selected. Based on the final selected models, we forecasted CS rates at sub-regional level in 2021, 2025, and 2030. The projections at the regional and global level were calculated as weighted means based on the share of live births by sub-region in the corresponding year.

Codes

```
/*data imputation*/


/*MI*/

/*
A total of 194 countries exclude:
14 countries csincount=0,
9 countries csincount=1,
8 countries csincount=2: these 8 countries with 1 observed cs rate records in two consecutive years,
9 countries with complete csincount=29 not need to do MI:DNK,EST,FIN,GBR,ISL,NOR,PRT,SWE,USA,
A total of 154 countries need MI
*/

/*MCMC MI*/
/*MI var: csinter */

proc sort data=csinterext; by ISO_Code;run;

/*estimate mi mu0*/
proc means data=csinterext;
var csinter;
weight lb;
run;

/*mi succeed in 113 countries (163-9(countries with complete data)-41(countries not complete mi) */
proc mi data=csinterext seed=100 out=csinmi1 nimpute=5 round=0.1 maximum=70 minimum=0 mu0=15;
  mcmc impute=full;
  var csinter year;
  by ISO_Code;
  where 2<csincount<29
and ISO_Code^="AFG" and ISO_Code^="AND" and ISO_Code^="ARE" and ISO_Code^="BDI" and
ISO_Code^="CPV"
and ISO_Code^="CYP" and ISO_Code^="FSM" and ISO_Code^="MDV" and ISO_Code^="SMR" and
ISO_Code^="GUY"
and ISO_Code^="SUR" and ISO_Code^="SWZ" and ISO_Code^="SLB" and ISO_Code^="BGD" and
ISO_Code^="LAO"
and ISO_Code^="BIH" and ISO_Code^="COD" and ISO_Code^="CHN" and ISO_Code^="GMB" and
ISO_Code^="GNB"
and ISO_Code^="IRQ" and ISO_Code^="LBR" and ISO_Code^="MCO" and ISO_Code^="MKD" and
ISO_Code^="MYS"
and ISO_Code^="NAM" and ISO_Code^="NPL" and ISO_Code^="PAN" and ISO_Code^="SAU" and
ISO_Code^="SDN"
and ISO_Code^="SRB" and ISO_Code^="TLS" and ISO_Code^="TON" and ISO_Code^="VNM" and
ISO_Code^="WSM"
and ISO_Code^="CAF" and ISO_Code^="PRK" and ISO_Code^="STP" and ISO_Code^="SYC" and
ISO_Code^="UZB"
and ISO_Code^="KHM";
run;

/*retry mcmc mi in 41 countries*/
/*
data retry;
set csinterext;
```

```
where
  ISO_Code="AFG" or ISO_Code="AND" or ISO_Code="ARE" or ISO_Code="BDI" /*or ISO_Code="CPV"
or ISO_Code="CYP"*/ or ISO_Code="FSM" /*or ISO_Code="MDV"*/ or ISO_Code="SMR" or
ISO_Code="GUY"
or ISO_Code="SUR" /*or ISO_Code="SWZ" or ISO_Code="SLB"*/ or ISO_Code="BGD" or ISO_Code="LAO"
or ISO_Code="BIH" or ISO_Code="COD" or ISO_Code="CHN" or ISO_Code="GMB" or ISO_Code="GNB"
/*or ISO_Code="IRQ"*/ or ISO_Code="LBR" or ISO_Code="MCO" /*or ISO_Code="MKD"*/ or
ISO_Code="MYS"
or ISO_Code="NAM" /*or ISO_Code="NPL"*/ or ISO_Code="PAN" /*or ISO_Code="SAU"*/ or
ISO_Code="SDN"
or ISO_Code="SRB" or ISO_Code="TLS" or ISO_Code="TON" or ISO_Code="VNM" or ISO_Code="WSM"
/*or ISO_Code="CAF" or ISO_Code="PRK" or ISO_Code="STP" or ISO_Code="SYC" or ISO_Code="UZB" or
ISO_Code="KHM"*/;
run;

proc sort data=retry;
by ISO_Code;
run;

proc mi data=retry seed=100 out=csinmi2 nimpute=5 round=0.1 maximum=100 minimum=0 mu0=15
MINMAXITER=1500;
  mcmc impute=full;
  var csinter year;
  by ISO_Code;

run;
*/


/*retry1 mi succeed in 4 countries: MKD,SAU,SYC,UZB
(increase minmaxiter)*/

data retry1;
set csinterext;
where
  ISO_Code="MKD" or ISO_Code="SAU" or ISO_Code="SYC" or ISO_Code="UZB";
run;

proc sort data=retry1;
by ISO_Code;
run;

proc mi data=retry1 seed=100 out=csinmiretry1 nimpute=5 round=0.1 maximum=100 minimum=0 mu0=15
MINMAXITER=1500;
  mcmc impute=full;
  var csinter year;
  by ISO_Code;

run;

/*retry2 mi succeeded in 7 countries (CPV,CYP,IRQ,MDV,NPL,SLB,SWZ)
(increase em maxiter=1000)*/

data retry2;
set csinterext;
where ISO_Code="CPV" or ISO_Code="CYP" or ISO_Code="IRQ" or ISO_Code="MDV" or ISO_Code="NPL"
```

```
or ISO_Code="SLB" or ISO_Code="SWZ";
run;

proc sort data=retry2;
by ISO_Code;
run;

proc mi data=retry2 seed=100 out=csinmiretry2 nimpute=5 round=0.1 maximum=100 minimum=0 mu0=15
MINMAXITER=3000;
  mcmc impute=full;
  em /*CONVERGE=0.001*/ maxiter=1000;
  var csinter year;
  by ISO_Code;
  run;

/*retry3:3 countries (CAF, PRK, STP)
 WARNING: The posterior covariance matrix is singular. Imputed values for some variables may be fixed.
*/
data retry3;
set csinterext;
where ISO_Code="CAF" or ISO_Code="PRK" or ISO_Code="STP";
run;

proc sort data=retry3;
by ISO_Code;
run;

proc mi data=retry3 seed=100 out=csinmiretry3 nimpute=5 round=0.1 maximum=100 minimum=0 mu0=15
MINMAXITER=3000;
  mcmc impute=full;
  /*em CONVERGE=0.001;*/
  var csinter year;
  by ISO_Code;
run;

/*retry4: 15 countries:BGD,BIH,CHN,COD,GMB,GNB, LAO, PAN, SDN, SRB, TLS, TON, VNM, WAM, KHM
 An imputed variable value is not in the specified range after 3000 tries.
*/

/*retry4_1: 6 countries:COD, GMB, PAN, TLS, VNM, KHM
(minimum=-5)*/

data retry4_1;
set csinterext;
where
  ISO_Code="COD" or ISO_Code="GMB" or ISO_Code="PAN" or
  ISO_Code="TLS" or ISO_Code="VNM" or ISO_Code="KHM";
run;

proc sort data=retry4_1;
by ISO_Code;
run;

proc mi data=retry4_1 seed=100 out=csinmiretry4_1 nimpute=5 round=0.1 maximum=100 minimum=-5 mu0=15
MINMAXITER=8000;
  mcmc impute=full;
```

```
  em maxiter=1000;
  var csinter year;
  by ISO_Code;
run;

proc univariate data=csinmiretry4_1;
var csinter;
run;


/*retry4_2 in 8 countries: An imputed variable value is not in the specified range after 8000 tries.
ISO_Code="BGD" or ISO_Code="CHN" or ISO_Code="LAO" or
ISO_Code="SRB" or ISO_Code="TON" or ISO_Code="GNB" or ISO_Code="WSM" or ISO_Code="SDN"

minimum=-10
*/

data retry4_2;
set csinterext;
where
ISO_Code="BGD" or ISO_Code="CHN" or ISO_Code="LAO" or
ISO_Code="SRB" or ISO_Code="TON" or ISO_Code="GNB" or ISO_Code="WSM" or ISO_Code="SDN";
run;

proc sort data=retry4_2;
by ISO_Code;
run;

proc mi data=retry4_2 seed=100 out=csinmiretry4_2 nimpute=5 round=0.1 maximum=100 minimum=-10 mu0=15
MINMAXITER=10000;
  mcmc impute=full;
  em CONVERGE=0.001 maxiter=2000;
  var csinter year;
  by ISO_Code;
run;

proc univariate data=csinmiretry4_2;
var csinter;
run;


/*retry4-3:BIH
minimum=-15
*/

data retry4_3;
set csinterext;
where
ISO_Code="BIH";
run;

proc mi data=retry4_3 seed=100 out=csinmiretry4_3 nimpute=5 round=0.1 maximum=100 minimum=-25 mu0=15
MINMAXITER=10000;
  mcmc impute=full;
  em CONVERGE=0.001 maxiter=500;
  var csinter year;
```

```
run;

proc univariate data=csinmiretry4_3;
var csinter;
run;

/*csinmiretry4*/
data csinmiretry4 (rename=(csinterrev=csinter));
set csinmiretry4_1-csinmiretry4_3;

if csinter>=0 then csinterrev=csinter;
if csinter<0 then csinterrev=0;
drop csinter;
run;


/*retry5: 12 countries:ISO_Code="AFG" or ISO_Code="AND" or ISO_Code="ARE" or ISO_Code="BDI"
or ISO_Code="FSM" or ISO_Code="SMR" or ISO_Code="GUY" or ISO_Code="SUR"
or ISO_Code="LBR" or ISO_Code="MCO" or ISO_Code="MYS" or ISO_Code="NAM"

(em CONVERGE=0.001 maxiter=1000)
*/


data retry5;
set csinterext;
where
  ISO_Code="AFG" or ISO_Code="AND" or ISO_Code="ARE" or ISO_Code="BDI"
or ISO_Code="FSM" or ISO_Code="SMR" or ISO_Code="GUY" or ISO_Code="SUR"
or ISO_Code="LBR" or ISO_Code="MCO" or ISO_Code="MYS" or ISO_Code="NAM" ;
run;

proc sort data=retry5;
by ISO_Code;
run;

proc mi data=retry5 seed=100 out=csinmiretry5 nimpute=5 round=0.1 maximum=100 minimum=0 mu0=15
MINMAXITER=10000;
  mcmc impute=full;
  em CONVERGE=0.001 maxiter=2000;
  var csinter year;
  by ISO_Code;
run;


/*combine data csinmi (163 countries)
 9 countries with complete csinter (completere)
+113 countries MI(cdinmi1)
+ 4 countries retry MI(csinmiretry1)
+7 countries (csinmiretry2)
+3 countries (csinmiretry3)
+15 countries (csinmiretry4): including 6 countries(csinmitrtry4_1)+ 8 countries (csinmiretry4_2)+1 country
(csinmiretry4_3)
+12 countries (csinmiretry5)
=163 countries included in data csinmi*/
```

```
data csinmi;
set completere csinmi1 csinmiretry1-csinmiretry5;
run;


/* Current global and regional caesarean section rates estimates*/

/*cs by region*/
/*cs*/
proc sql;
create table cscrossinmiregion as
select distinct Region_Name, count(csinter) as csnum, sum(csinter*lb2018)/sum(lb2018) as lastcs,min (csinter) as
mincs,max(csinter) as maxcs,sum(csinter*lb2018)/100*1000 as lastcsnum,sum(lb2018)*1000 as lastlbnum
from Cslastcrossinmiave
where lb2018^=. and lastcsinyear>=2010
group by Region_Name
order by Region_Name;
quit;
/*coverage*/
data cscrossinmiregion;
merge cscrossinmiregion cs.lbregion;
coverage=lastlbnum/lbregion2018*100;
drop lbregion1990-lbregion2017;

run;

/*lastcs by un subregion*/

/*cs*/
proc sql;
create table cscrossinmisubregion as
select distinct Region_Name, Sub_region_Name, count(csinter) as csnum, sum(csinter*lb2018)/sum(lb2018) as
lastcs,min (csinter) as mincs,max(csinter) as maxcs,sum(csinter*lb2018)/100*1000 as lastcsnum,sum(lb2018)*1000
as lastlbnum
from Cslastcrossinmiave
where lb2018^=. and lastcsinyear>=2010
group by Sub_region_Name
order by Region_Name,Sub_region_Name;
quit;
/*coverage*/
data cscrossinmisubregion;
merge cscrossinmisubregion cs.lbsubregion;
coverage=lastlbnum/lbsubregion2018*100;
drop lbsubregion1990-lbsubregion2017;
run;

/*lastcs world total*/
/*cs*/
proc sql;
create table cscrossinmiworld as
select distinct count(csinter) as csnum, sum(csinter*lb2018)/sum(lb2018) as lastcs,min (csinter) as
mincs,max(csinter) as maxcs,sum(csinter*lb2018)/100*1000 as lastcsnum,sum(lb2018)*1000 as lastlbnum
from Cslastcrossinmiave
where lb2018^=. and lastcsinyear>=2010;
quit;
/*coverage*/
```

```
data cscrossinmiworld;
merge cscrossinmiworld cs.lbworld;
coverage=lastlbnum/lbworld2018*100;
drop lbworld1990-lbworld2017;
run;
/*lastcs by develop3*/
/*cs*/
proc sql;
create table cscrossinmidevelop as
select distinct develop3, count(csinter) as csnum, sum(csinter*lb2018)/sum(lb2018) as lastcs,min (csinter) as
mincs,max(csinter) as maxcs,sum(csinter*lb2018)/100*1000 as lastcsnum,sum(lb2018)*1000 as lastlbnum
from Cslastcrossinmiave
where lb2018^=. and lastcsinyear>=2010
group by develop3
order by develop3;
quit;
/*coverage*/
data cscrossinmidevelop;
merge cscrossinmidevelop cs.lbdevelop;
coverage=lastlbnum/lbdevelop2018*100;

drop lbdevelop1990-lbdevelop2017;
run;

/* Trends on caesarean section rates*/

/*by region*/
proc sort data=csinmiavehor;
by Region_Name;
run;
/*change9000*/
proc glimmix data=csinmiavehor method=laplace; /* conditional mean */
class ISO_Code;
model changenum9000/lb2005num = /solution;
random intercept/subject=ISO_Code;
estimate 'intercept' intercept 1 / cl ilink;
by Region_Name;
run;
/*change0010*/
proc glimmix data=csinmiavehor method=laplace; /* conditional mean */
class ISO_Code;
model changenum0010/lb2005num = /solution;
random intercept/subject=ISO_Code;
estimate 'intercept' intercept 1 / cl ilink;
by Region_Name;
run;
/*change1018*/
proc glimmix data=csinmiavehor method=laplace; /* conditional mean */
class ISO_Code;
model changenum1018/lb2005num = /solution;
random intercept/subject=ISO_Code;
estimate 'intercept' intercept 1 / cl ilink;
by Region_Name;
run;
/*change9018*/
proc glimmix data=csinmiavehor method=laplace; /* conditional mean */
```

```
class ISO_Code;
model changenum9018/lb2005num = /solution;
random intercept/subject=ISO_Code;
estimate 'intercept' intercept 1 / cl ilink;
by Region_Name;
run;

/*by subregion*/
proc sort data=csinmiavehor;
by Region_Name Sub_region_Name;
run;
/*change9000*/
proc glimmix data=csinmiavehor method=laplace; /* conditional mean */
class ISO_Code;
model changenum9000/lb2005num = /solution;
random intercept/subject=ISO_Code;
estimate 'intercept' intercept 1 / cl ilink;
by Region_Name Sub_region_Name;
run;


/*change0010*/
proc glimmix data=csinmiavehor method=laplace; /* conditional mean */
class ISO_Code;
model changenum0010/lb2005num = /solution;
random intercept/subject=ISO_Code;
estimate 'intercept' intercept 1 / cl ilink;
by Region_Name Sub_region_Name;
run;
/*change1018*/
proc glimmix data=csinmiavehor method=laplace; /* conditional mean */
class ISO_Code;
model changenum1018/lb2005num = /solution;
random intercept/subject=ISO_Code;
estimate 'intercept' intercept 1 / cl ilink;
by Region_Name Sub_region_Name;
run;
/*change9018*/
proc glimmix data=csinmiavehor method=laplace; /* conditional mean */
class ISO_Code;
model changenum9018/lb2005num = /solution;
random intercept/subject=ISO_Code;
estimate 'intercept' intercept 1 / cl ilink;
by Region_Name Sub_region_Name;
run;

/* Projections of caesarean section rates for 2030*/
/*Northern Africa*/
ods output Forecasts=pnorafr;
proc arima data=csinmiregsubdev;
identify var=csinter(1)  minic scan esacf  stationarity=(adf=(5,6,7,8));
where Sub_region_Name="Northern Africa";
estimate p=4 q=0  plot;
forecast lead=12 interval=year id=time out=results;
run;
quit;
```

```
ods output close;

data pnorafr;
set pnorafr;
Region_Name="Africa";
Sub_region_Name="Northern Africa";
run;

data norafr ;
set results;
Region_Name="Africa";
Sub_region_Name="Northern Africa";
where time=mdy(1,1,2021) or time=mdy(1,1,2025) or time=mdy(1,1,2030);
run;

/*Sub-Saharan Africa*/
ods output Forecasts=psubafr;
proc arima data=csinmiregsubdev;
identify var=csinter(3) minic scan esacf  stationarity=(adf=(5,6,7,8));
where Sub_region_Name="Sub-Saharan Africa";

estimate p=0 q=1  plot;
forecast lead=12 interval=year id=time out=results;
run;
quit;
ods output close;

data psubafr;
set psubafr;
Region_Name="Africa";
Sub_region_Name="Sub-Saharan Africa";
run;

data subafr ;
set results;
Region_Name="Africa";
Sub_region_Name="Sub-Saharan Africa";
where time=mdy(1,1,2021) or time=mdy(1,1,2025) or time=mdy(1,1,2030);
run;

/*Latin America and the Caribbean*/
ods output Forecasts=platame;
proc arima data=csinmiregsubdev;
identify var=csinter(2) minic scan esacf  stationarity=(adf=(5,6,7,8));
where Sub_region_Name="Latin America and the Caribbean";
estimate p=0 q=1  plot;
forecast lead=12 interval=year id=time out=results;
run;
quit;
ods output close;

data platame;
set platame;
Region_Name="Americas";
Sub_region_Name="Latin America and the Caribbean";
run;
```

```
data latame ;
set results;
Region_Name="Americas";
Sub_region_Name="Latin America and the Caribbean";
where time=mdy(1,1,2021) or time=mdy(1,1,2025) or time=mdy(1,1,2030);
run;


/*Northern America*/
ods output Forecasts=pnorame;
proc arima data=csinmiregsubdev;
identify var=csinter(1) nlag=6 minic scan esacf stationarity=(adf=(1,2,3));
where Sub_region_Name="Northern America" and 2005<=year<=2018;
estimate p=1 q=1  plot;
forecast lead=12 interval=year id=time out=results;
run;
quit;
ods output close;

data pnorame;
set pnorame;
Region_Name="Americas";
Sub_region_Name="Northern America";
run;

data norame ;
set results;
Region_Name="Americas";
Sub_region_Name="Northern America";
where time=mdy(1,1,2021) or time=mdy(1,1,2025) or time=mdy(1,1,2030);
run;

/*Central Asia*/
ods output Forecasts=pcenasi;
proc arima data=csinmiregsubdev;
identify var=csinter(1,1) nlag=6 minic scan esacf  stationarity=(adf=(1,2,3));
where Sub_region_Name="Central Asia" and 2000<=year<=2018;
estimate p=0 q=3  plot;
forecast lead=12 interval=year id=time out=results;
run;
quit;
ods output close;

data pcenasi;
set pcenasi;
Region_Name="Asia";
Sub_region_Name="Central Asia";
run;

data cenasi ;
set results;
Region_Name="Asia";
Sub_region_Name="Central Asia";
where time=mdy(1,1,2021) or time=mdy(1,1,2025) or time=mdy(1,1,2030);
run;
```


Betran AP, *et al. BMJ Global Health* 2021; 6:e005671. doi: 10.1136/bmjgh-2021-005671

```
/*Eastern Asia*/
ods output Forecasts=peasasi;
proc arima data=csinmiregsubdev;
identify var=csinter(2) minic scan esacf  stationarity=(adf=(5,6,7,8));
where Sub_region_Name="Eastern Asia";
estimate p=4 q=0 plot;
forecast lead=12 interval=year id=time out=results;
run;
quit;
ods output close;

data peasasi;
set peasasi;
Region_Name="Asia";
Sub_region_Name="Eastern Asia";
run;

data easasi ;
set results;
Region_Name="Asia";
Sub_region_Name="Eastern Asia";
where time=mdy(1,1,2021) or time=mdy(1,1,2025) or time=mdy(1,1,2030);
run;

/*South-eastern Asia*/
ods output Forecasts=psoueasasi;
proc arima data=csinmiregsubdev;
identify var=csinter(3) minic scan esacf  stationarity=(adf=(5,6,7,8));
where Sub_region_Name="South-eastern Asia";
estimate p=2 q=0  plot;
forecast lead=12 interval=year id=time out=results;
run;
quit;
ods output close;

data psoueasasi;
set psoueasasi;
Region_Name="Asia";
Sub_region_Name="South-eastern Asia";
run;

data soueasasi ;
set results;
Region_Name="Asia";
Sub_region_Name="South-eastern Asia";
where time=mdy(1,1,2021) or time=mdy(1,1,2025) or time=mdy(1,1,2030);
run;

/*Southern Asia*/
ods output Forecasts=psouasi;
proc arima data=csinmiregsubdev;
identify var=csinter(2) minic  scan esacf  stationarity=(adf=(5,6,7,8));
where Sub_region_Name="Southern Asia";
estimate p=2 q=1  plot;
```

```
forecast lead=12 interval=year id=time out=results;
run;
quit;
ods output close;

data psouasi;
set psouasi;
Region_Name="Asia";
Sub_region_Name="Southern Asia";
run;

data souasi ;
set results;
Region_Name="Asia";
Sub_region_Name="Southern Asia";
where time=mdy(1,1,2021) or time=mdy(1,1,2025) or time=mdy(1,1,2030);
run;

/*Western Asia*/
ods output Forecasts=pwesasi;
proc arima data=csinmiregsubdev;
identify var=csinter(5) nlag=6 minic scan esacf  stationarity=(adf=(5,6,7,8));
where Sub_region_Name="Western Asia";

estimate p=0 q=1  plot;
forecast lead=12 interval=year id=time out=results;
run;
quit;
ods output close;

data pwesasi;
set pwesasi;
Region_Name="Asia";
Sub_region_Name="Western Asia";
run;

data wesasi ;
set results;
Region_Name="Asia";
Sub_region_Name="Western Asia";
where time=mdy(1,1,2021) or time=mdy(1,1,2025) or time=mdy(1,1,2030);
run;

/*Eastern Europe*/
ods output Forecasts=peaseur;
proc arima data=csinmiregsubdev;
identify var=csinter(2) minic scan esacf nlag=6 stationarity=(adf=(5,6,7,8));
where Sub_region_Name="Eastern Europe";
estimate p=0 q=2  plot;
forecast lead=12 interval=year id=time out=results;
run;
quit;
ods output close;

data peaseur;
set peaseur;
```

```
Region_Name="Europe";
Sub_region_Name="Eastern Europe";
run;

data easeur ;
set results;
Region_Name="Europe";
Sub_region_Name="Eastern Europe";
where time=mdy(1,1,2021) or time=mdy(1,1,2025) or time=mdy(1,1,2030);
run;

/*Northern Europe*/
/* After any differencing is performed, at
    least six (6) observations are required by the ARIMA procedure for the identification step.
*/
ods output Forecasts=pnoreur;
proc arima data=csinmiregsubdev;
identify var=csinter(1,1) nlag=6 minic scan esacf  stationarity=(adf=(1,2,3));
where Sub_region_Name="Northern Europe" and 2005<=year<=2018;
estimate p=0 q=0  plot;
forecast lead=12 interval=year id=time out=results;
run;
quit;
ods output close;

data pnoreur;
set pnoreur;
Region_Name="Europe";
Sub_region_Name="Northern Europe";
run;

data noreur ;
set results;
Region_Name="Europe";
Sub_region_Name="Northern Europe";
where time=mdy(1,1,2021) or time=mdy(1,1,2025) or time=mdy(1,1,2030);
run;

/*Southern Europe*/
ods output Forecasts=psoueur;
proc arima data=csinmiregsubdev;
identify var=csinter(3) nlag=6 minic scan esacf  stationarity=(adf=(5,6,7,8));
where Sub_region_Name="Southern Europe";
estimate p=2 q=0  plot;
forecast lead=12 interval=year id=time out=results;
run;
quit;
ods output close;

data psoueur;
set psoueur;
Region_Name="Europe";
Sub_region_Name="Southern Europe";
run;

data soueur ;
```

```
set results;
Region_Name="Europe";
Sub_region_Name="Southern Europe";
where time=mdy(1,1,2021) or time=mdy(1,1,2025) or time=mdy(1,1,2030);
run;

/*Western Europe*/
ods output Forecasts=pweseur;
proc arima data=csinmiregsubdev;
identify var=csinter(3) nlag=6 minic scan esacf  stationarity=(adf=(5,6,7,8));
where Sub_region_Name="Western Europe";
estimate p=2 q=0  plot;
forecast lead=12 interval=year id=time out=results;
run;
quit;
ods output close;

data pweseur;
set pweseur;
Region_Name="Europe";
Sub_region_Name="Western Europe";
run;

data weseur ;
set results;
Region_Name="Europe";
Sub_region_Name="Western Europe";
where time=mdy(1,1,2021) or time=mdy(1,1,2025) or time=mdy(1,1,2030);
run;

/*Australia and New Zealand*/
ods output Forecasts=pausoce;
proc arima data=csinmiregsubdev;
identify var=csinter(1) nlag=6 minic scan esacf  stationarity=(adf=(1,2,3));
where Sub_region_Name="Australia and New Zealand" and 1990<=year<=2018;
estimate p=1 q=0  plot;
forecast lead=12 interval=year id=time out=results;
run;
quit;
ods output close;

data pausoce;
set pausoce;
Region_Name="Oceania";
Sub_region_Name="Australia and New Zealand";
run;

data ausoce ;
set results;
Region_Name="Oceania";
Sub_region_Name="Australia and New Zealand";
where time=mdy(1,1,2021) or time=mdy(1,1,2025) or time=mdy(1,1,2030);
run;

data subregionarima;
set Norafr Subafr Latame Norame Cenasi Easasi Soueasasi Souasi Wesasi Easeur Noreur Soueur Weseur Ausoce;
```

```
run;
```

4. Supplementary tables and other results

Supplementary table 1: Caesarean section rates in countries categorised according to United Nations geographical grouping in 2018[a] using imputed data

Region/subregion	Estimated CS rate (%, 95% CI)	Range (min-max, %)	Coverage of estimates (%)
**Africa** (n=44)	10.1 (5.6, 14.5)	1.3-60.4	89.8
Northern Africa (n=5)	36.0 (5.2, 66.8)	12.2-60.4	97.9
Sub-Saharan Africa (n=39)	5.7 (4.0, 7.4)	1.3-53.9	88.6
**Asia** (n=40)	26.4 (21.6, 31.2)	4.3-62.7	96.9
Central Asia (n=5)	11.9 (6.4, 17.4)	5.4-17.5	100.0
Eastern Asia (n=5)	46.3 (35.5, 57.2)	13.0-48.8	100.0
South-eastern Asia (n=8)	18.0 (10.6, 25.4)	4.7-36.6	94.9
Southern Asia (n=7)	18.2 (12.9, 23.5)	4.3-41.3	96.6
Western Asia (n=15)	35.1 (24.4, 45.8)	7.9-62.7	91.7
**Europe** (n=38)	29.5 (27.0, 32.1)	14.9-44.0	98.9
Eastern Europe (n=10)	29.8 (24.1, 35.5)	18.8-44.0	100.0
Northern Europe (n=10)	25.9 (21.9, 29.9)	15.9-34.3	100.0
Southern Europe (n=11)	35.7 (31.1, 40.4)	22.6-42.9	93.3
Western Europe (n=7)	27.6 (20.4, 34.9)	14.9-36.8	100.0
**Americas** (n=25)	41.3 (35.8, 46.8)	6.8-63.4	93.8
Latin America and the Caribbean (n=23)	45.8 (39.7, 51.8)	6.8-63.4	91.1
Northern America (n=2)	31.8 (28.5, 35.1)	31.0-31.9	100.0
**Oceania** (n=7)	22.3 (6.5, 38.1)	0.0-36.6	96.5
Australia and New Zealand (n=2)	35.1 (-7.3, 77.6)	27.6-36.6	100.0
Melanesia, Micronesia, and Polynesia (n=5)	3.6 (-0.5, 7.7)	0.0-28.5	91.9
**World total** (n=154)	23.4 (20.6, 26.1)	**0.0-63.4**	**94.5**
More developed countries (n=45)	29.8 (27.9, 31.8)	14.9-58.3	99.4
Less developed countries (n=70)	27.2 (22.9, 31.5)	0.0-63.4	94.6
Least developed countries (n=39)	8.9 (6.1, 11.7)	1.3-31.8	91.9

[a] Countries with the latest CS rate record available in 2010 or later were included.

Supplementary table 2: Number of observed caesarean section rate data points and sampling period for projections.

Region/subregion	2010-2018		2000-2018		1990-2018		Sampling period
	Data points (N)	Proportion (%)	Data points (N)	Proportion (%)	Data points (N)	Proportion (%)	
**Africa**							
Northern Africa	14	31.1	29	30.5	41	28.3	1990-2018
Sub-Saharan Africa	95	24.5	222	27.2	310	24.9	1990-2018
**Asia**							
Central Asia	34	75.6	84	88.4	128	88.3	2000-2018
Eastern Asia	12	26.7	27	28.4	37	25.5	1990-2018
South-eastern Asia	18	25.0	43	28.3	62	26.7	1990-2018
Southern Asia	25	34.7	57	37.5	67	28.9	1990-2018
Western Asia	81	56.3	164	53.9	223	48.1	1990-2018
**Europe**							
Eastern Europe	56	62.2	150	78.9	234	80.7	1990-2018
Northern Europe	85	94.4	185	97.4	282	97.2	2005-2018
Southern Europe	71	60.7	170	68.8	243	64.5	1990-2018
Western Europe	54	75.0	115	75.7	158	68.1	1990-2018
**Americas**							
Latin America and the Caribbean	86	41.5	203	46.5	288	43.2	1990-2018
Northern America	15	83.3	35	92.1	51	87.9	2005-2018
**Oceania**							
Australia and New Zealand	16	88.9	36	94.7	46	79.3	1990-2018
**World total**							
More developed countries	311	72.0	721	79.1	1056	75.9	1990-2018
Less developed countries	272	39.8	627	43.4	878	39.8	1990-2018
Least developed countries	85	24.2	196	26.5	260	23.0	1990-2018

Supplementary table 3: Predicted caesarean section rates and numbers using autoregressive integrated moving-average models globaly, by region and sub-region.

Region/subregion [a]	2021		2025		2030	
	CS rate (%, 95% CI)	Number of CS (95% CI)	CS rate (%, 95% CI)	Number of CS (95% CI)	CS rate (%, 95% CI)	Number of CS (95% CI)
**Africa** (n=48)	10.4 (9.6, 11.3)	4366667 (4018732, 4714603)	11.0 (9.5, 12.5)	4820849 (4162233, 5479465)	12.0 (10.1, 13.9)	5579213 (4682901, 6475526)
Northern Africa (n=5)	39.0 (35.1, 42.8)	2140126 (1928253, 2351999)	42.1 (34.3, 49.9)	2285559 (1860664, 2710454)	48.1 (37.4, 58.8)	2648794 (2059522, 3238067)
Sub-Saharan Africa (n=43)	6.1 (5.7, 6.5)	2226542 (2090480, 2362604)	6.6 (6.0, 7.2)	2535290 (2301569, 2769011)	7.1 (6.4, 7.9)	2930419 (2623380, 3237459)
**Asia (n=42)**	28.5 (25.6, 31.4)	20251726 (18182738, 22320715)	30.6 (25.8, 35.5)	21098704 (17767704, 24429703)	35.2 (29.6, 40.8)	23448681 (19698915, 27198447)
Central Asia (n=5)	11.9 (10.5, 13.4)	176552 (154551, 198554)	12.4 (7.6, 17.2)	172422 (105479, 239365)	13.3 (2.0, 24.6)	179322 (27261, 331382)
Eastern Asia (n=5)	50.6 (44.9, 56.4)	8674013 (7686903, 9661124)	54.7 (45.2, 64.2)	8763514 (7243904, 10283123)	63.4 (52.9, 74.0)	9614950 (8015153, 11214747)
South-eastern Asia (n=8)	20.2 (19.1, 21.3)	2128241 (2014104, 2242379)	22.4 (20.7, 24.1)	2309804 (2133707, 2485900)	25.3 (23.2, 27.4)	2542985 (2331166, 2754803)
Southern Asia (n=8)	19.4 (17.0, 21.7)	7069469 (6210630, 7928309)	20.9 (16.8, 24.9)	7446897 (6001887, 8891907)	24.2 (19.4, 28.9)	8353151 (6719198, 9987103)
Western Asia (n=16)	40.5 (38.9, 42.1)	2203450 (2116551, 2290349)	44.1 (41.8, 46.3)	2406067 (2282727, 2529408)	50.2 (47.4, 52.9)	2758274 (2606137, 2910412)
**Europe** (n=38)	31.3 (28.5, 34.1)	2334577 (2123342, 2545812)	33.2 (28.5, 38.0)	2380762 (2042680, 2718844)	36.5 (29.7, 43.3)	2509184 (2044674, 2973693)
Eastern Europe (n=10)	31.2 (27.8, 34.6)	954184 (849860, 1058508)	34.3 (29.9, 38.7)	968045 (844397, 1091694)	38.3 (32.9, 43.6)	993099 (854021, 1132177)
Northern Europe (n=10)	26.4 (24.8, 28.1)	328468 (307637, 349300)	27.0 (21.7, 32.3)	330610 (265760, 395460)	27.6 (16.2, 39.1)	331373 (194448, 468298)
Southern Europe (n=11)	39.1 (35.8, 42.3)	454396 (416561, 492231)	41.7 (35.4, 48.0)	471877 (400917, 542838)	47.0 (38.8, 55.3)	527454 (434850, 620058)
Western Europe (n=7)	30.0 (27.5, 32.4)	597528 (549284, 645773)	30.7 (26.7, 34.7)	610229 (531606, 688852)	33.5 (28.7, 38.4)	657258 (561355, 753161)

**Americas** (n=25)	42.6 (40.0, 45.2)	6117950 (5745393, 6490506)	44.6 (39.8, 49.4)	6322345 (5642561, 7002130)	47.2 (39.6, 54.9)	6537574 (5478009, 7597139)
Latin America and the Caribbean (n=23)	47.5 (44.6, 50.5)	4626678 (4339223, 4914133)	50.5 (46.0, 55.0)	4772742 (4346811, 5198673)	54.3 (48.3, 60.2)	4929455 (4392984, 5465927)
Northern America (n=2)	32.2 (30.4, 34)	1491272 (1406171, 1576373)	32.8 (27.4, 38.2)	1549603 (1295750, 1803457)	33.8 (22.8, 44.8)	1608119 (1085025, 2131213)
**Oceania** (n=6)	36.4 (34.3, 38.5)	148196 (139587, 156806)	39.2 (34.8, 43.7)	160148 (141941, 178355)	42.3 (35.6, 48.9)	172754 (145525, 199984)
Australia and New Zealand (n=2)	38.5 (36.3, 40.7)	146375 (138006, 154745)	41.6 (37.0, 46.3)	158302 (140565, 176038)	45.0 (38.1, 52.0)	170911 (144559, 197262)
**World total** (n=159)	24.6 (22.3, 26.8)	33219117 (30209793, 36228441)	25.9 (22.1, 29.6)	34782808 (29757119, 39808497)	28.5 (23.9, 33.1)	38247407 (32050024, 44444789)
More developed countries (n=45)	31.4 (29.5, 33.3)	4280118 (4020425, 4539811)	33.7 (30.4, 37.1)	4521314 (4070733, 4971896)	36.6 (31.7, 41.4)	4802717 (4167930, 5437504)
Less developed countries (n=75)	30.2 (28.5, 31.8)	27602787 (26117568, 29088006)	33.7 (30.5, 36.8)	30206101 (27357034, 33055169)	36.5 (32.7, 40.3)	32197073 (28821362, 35572785)
Least developed countries (n=39)	9.0 (7.9, 10.1)	2692984 (2358436, 3027533)	10.1 (8.5, 11.8)	3177239 (2654085, 3700394)	11.8 (9.7, 13.8)	3879770 (3196387, 4563152)

[a] Countries categorized according to the UN geographical grouping.

Supplementary table 4: Projections of CS rates, number of births, number of CS and estimated CS-related mortality by geographic subregion and year (2021-2030).

Sub-region	Year	Projected CS rate (95% CI)	Projected number of live births	Estimated number of CS
Northern Africa	2021	39% (35,1% -42,8%)	5493805	2140126
Northern Africa	2022	38,7% (33,6% -43,8%)	5471991	2116814
Northern Africa	2023	39,9% (33,5% -46,2%)	5453542	2174780
Northern Africa	2024	42% (35% -49,1%)	5439749	2285742
Northern Africa	2025	42,1% (34,3% -49,9%)	5432088	2285559
Northern Africa	2026	42,9% (34,3% -51,6%)	5431758	2331379
Northern Africa	2027	45,1% (35,9% -54,3%)	5438879	2452970
Northern Africa	2028	45,5% (35,8% -55,2%)	5453251	2480283
Northern Africa	2029	46% (35,8% -56,3%)	5474665	2520490
Northern Africa	2030	48,1% (37,4% -58,8%)	5503036	2648794
Sub-Saharan Africa	2021	6,1% (5,7% -6,5%)	36385972	2226542
Sub-Saharan Africa	2022	6,3% (5,8% -6,7%)	36895590	2307859
Sub-Saharan Africa	2023	6,2% (5,7% -6,8%)	37408921	2332648
Sub-Saharan Africa	2024	6,5% (5,9% -7%)	37925355	2449514
Sub-Saharan Africa	2025	6,6% (6% -7,2%)	38444633	2535290
Sub-Saharan Africa	2026	6,6% (5,9% -7,2%)	38966568	2562085
Sub-Saharan Africa	2027	6,8% (6,2% -7,4%)	39490156	2684667
Sub-Saharan Africa	2028	6,9% (6,2% -7,6%)	40013835	2774638
Sub-Saharan Africa	2029	6,9% (6,2% -7,7%)	40535843	2802903
Sub-Saharan Africa	2030	7,1% (6,4% -7,9%)	41054567	2930419
Latin America and the Caribbean	2021	47,5% (44,6% -50,5%)	9737161	4626678
Latin America and the Caribbean	2022	48,2% (44,8% -51,6%)	9665677	4661043
Latin America and the Caribbean	2023	49% (45,2% -52,8%)	9592749	4702668
Latin America and the Caribbean	2024	49,7% (45,6% -53,9%)	9519067	4733841
Latin America and the Caribbean	2025	50,5% (46% -55%)	9445247	4772742
Latin America and the Caribbean	2026	51,2% (46,4% -56,1%)	9371720	4801842
Latin America and the Caribbean	2027	52% (46,9% -57,2%)	9298733	4838884
Latin America and the Caribbean	2028	52,7% (47,4% -58,1%)	9226566	4866557
Latin America and the Caribbean	2029	53,5% (47,9% -59,2%)	9155608	4902423

Betran AP, *et al. BMJ Global Health* 2021; 6:e005671. doi: 10.1136/bmjgh-2021-005671

Latin America and the Caribbean	2030	54,3% (48,3% -60,2%)	9086131	4929455
Central Asia	2021	11,9% (10,5% -13,4%)	1477934	176552
Central Asia	2022	12% (10% -14,1%)	1454371	175011
Central Asia	2023	12,1% (9,3% -15%)	1431382	173736
Central Asia	2024	12,3% (8,5% -16%)	1409933	172836
Central Asia	2025	12,4% (7,6% -17,2%)	1390968	172422
Central Asia	2026	12,5% (6,6% -18,5%)	1375170	172582
Central Asia	2027	12,7% (5,5% -19,9%)	1362619	173332
Central Asia	2028	12,9% (4,4% -21,4%)	1353329	174684
Central Asia	2029	13,1% (3,3% -23%)	1347428	176669
Central Asia	2030	13,3% (2% -24,6%)	1345047	179322
Eastern Asia	2021	50,6% (44,9% -56,4%)	17137965	8674013
Eastern Asia	2022	51,6% (44,6% -58,6%)	16829607	8684255
Eastern Asia	2023	52,2% (43,9% -60,5%)	16537276	8632114
Eastern Asia	2024	53,2% (44,3% -62%)	16267175	8650289
Eastern Asia	2025	54,7% (45,2% -64,2%)	16023617	8763514
Eastern Asia	2026	56,1% (46,4% -65,8%)	15807736	8870838
Eastern Asia	2027	57,9% (47,9% -67,9%)	15615765	9043046
Eastern Asia	2028	59,8% (49,7% -69,9%)	15444396	9240229
Eastern Asia	2029	61,7% (51,3% -72,1%)	15293024	9440314
Eastern Asia	2030	63,4% (52,9% -74%)	15161556	9614950
South-eastern Asia	2021	20,2% (19,1% -21,3%)	10518858	2128241
South-eastern Asia	2022	20,7% (19,3% -22,1%)	10472606	2168692
South-eastern Asia	2023	21,2% (19,7% -22,7%)	10423749	2211757
South-eastern Asia	2024	21,9% (20,4% -23,4%)	10372703	2270525
South-eastern Asia	2025	22,4% (20,7% -24,1%)	10320133	2309804
South-eastern Asia	2026	22,9% (21,1% -24,7%)	10266892	2351334
South-eastern Asia	2027	23,6% (21,7% -25,4%)	10214096	2408126
South-eastern Asia	2028	24,1% (22,1% -26,1%)	10162794	2445958
South-eastern Asia	2029	24,6% (22,5% -26,7%)	10113759	2486587
South-eastern Asia	2030	25,3% (23,2% -27,4%)	10067702	2542985
Southern Asia	2021	19,4% (17% -21,7%)	36475904	7069469
Southern Asia	2022	20,4% (17,7% -23%)	36285073	7385423

Southern Asia	2023	20% (16,7% -23,4%)	36087251	7229930
Southern Asia	2024	21,1% (17,5% -24,7%)	35884547	7573092
Southern Asia	2025	20,9% (16,8% -24,9%)	35677725	7446897
Southern Asia	2026	22% (17,8% -26,2%)	35466219	7809541
Southern Asia	2027	21,9% (17,4% -26,3%)	35248654	7703434
Southern Asia	2028	23,1% (18,5% -27,6%)	35024542	8075370
Southern Asia	2029	22,9% (18,2% -27,6%)	34794873	7980140
Southern Asia	2030	24,2% (19,4% -28,9%)	34560468	8353151
Western Asia	2021	40,5% (38,9% -42,1%)	5439007	2203450
Western Asia	2022	41,4% (39,8% -43%)	5443660	2255857
Western Asia	2023	41,7% (40,1% -43,3%)	5448364	2273582
Western Asia	2024	42,4% (40,3% -44,4%)	5453323	2310084
Western Asia	2025	44,1% (41,8% -46,3%)	5458753	2406067
Western Asia	2026	46,6% (44,3% -48,9%)	5464845	2546973
Western Asia	2027	47,5% (45,3% -49,8%)	5471697	2600948
Western Asia	2028	47,8% (45,6% -50,1%)	5479385	2620468
Western Asia	2029	48,5% (45,9% -51,1%)	5487998	2659240
Western Asia	2030	50,2% (47,4% -52,9%)	5497665	2758274

Supplementary figure 1: The top 10 countries with the maximum absolute or relative increase in caesarean section rate from 1990 to 2018 and separately for each of the three periods studied (1990-2000, 2000-2010 and 2010-2018).

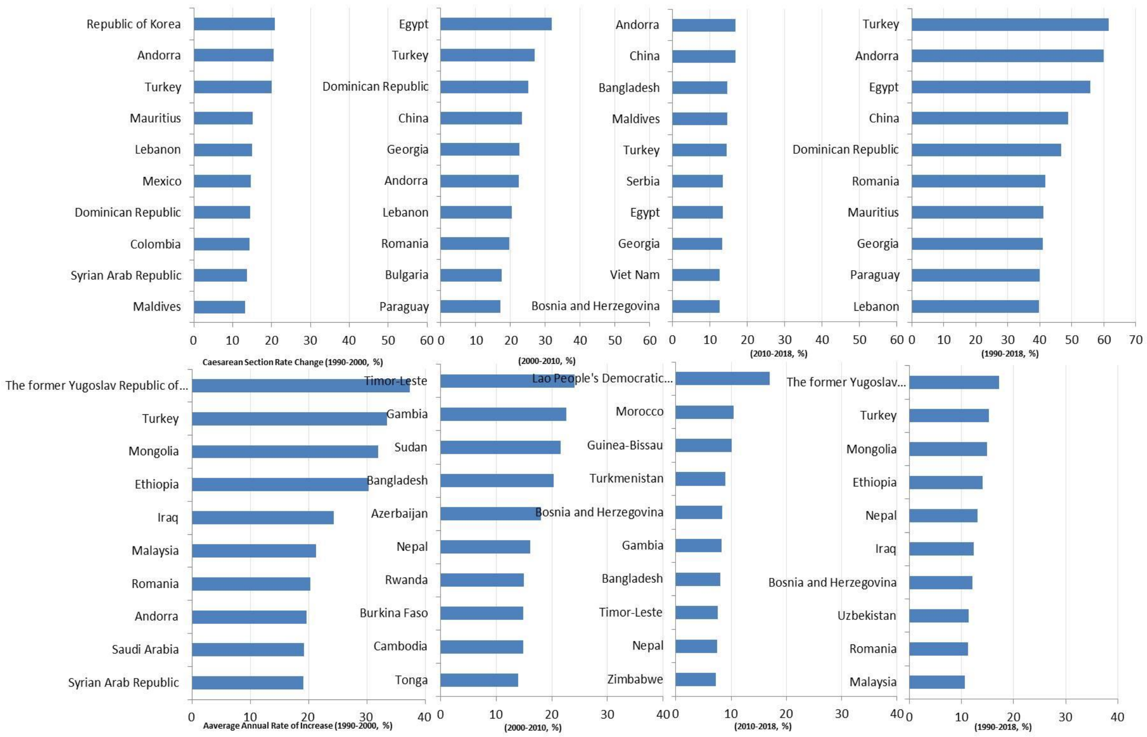

5. Data points included in the analysis by country and year and source of data

Supplementary table 5: Data points included in the analysis by country and year and source of data.

ISO Code	Country	Coverage start year	Coverage end year	Caesarean section rate (%)	References
AFG	Afghanistan	2005	2010	5.0	Afghanistan Mortality Survey 2010. Calverton: Afghan Public Health Institute, Ministry of Public Health (APHI/MoPH) [Afghanistan], Central Statistics Organization (CSO) [Afghanistan], ICF Macro, Indian Institute of Health Management Research (IIHMR) [India], and World Health Organization Regional Office for the Eastern Mediterranean (WHO/EMRO) [Egypt]; 2011.
AFG	Afghanistan	2008	2011	3.6	Central Statistics Organisation (CSO), UNICEF. Afghanistan Multiple Indicator Cluster Survey 2010-2011: Final Report. Kabul: Central Statistics Organisation (CSO), UNICEF; 2012.
AFG	Afghanistan	2010	2015	2.7	Central Statistics Organization (CSO), Ministry of Public Health (MoPH), ICF. Afghanistan Demographic and Health Survey 2015. Kabul, Afghanistan: Central Statistics Organization; 2017.
AFG	Afghanistan	2016	2018	6.6	Afghanistan Health Survey 2018. April 2019. Amsterdam: KIT Royal Tropical Institute; 2018.
ALB	Albania	1992	1992	9.3	European Health for All Database (HFA-DB) [online database]. World Health Organization (WHO) Regional Office for Europe; 2012 (http://data.euro.who.int/hfadb, accessed 20 August 2012).
ALB	Albania	1993	1993	9.8	European Health for All Database (HFA-DB) [online database]. World Health Organization (WHO) Regional Office for Europe; 2012 (http://data.euro.who.int/hfadb, accessed 20 August 2012).

ISO Code	Country	Coverage start year	Coverage end year	Caesarean section rate (%)	References
ALB	Albania	1994	1994	7.8	European Health for All Database (HFA-DB) [online database]. World Health Organization (WHO) Regional Office for Europe; 2012 (http://data.euro.who.int/hfadb, accessed 20 August 2012).
ALB	Albania	1995	1995	8.4	European Health for All Database (HFA-DB) [online database]. World Health Organization (WHO) Regional Office for Europe; 2012 (http://data.euro.who.int/hfadb, accessed 20 August 2012).
ALB	Albania	1996	1996	8.6	European Health for All Database (HFA-DB) [online database]. World Health Organization (WHO) Regional Office for Europe; 2012 (http://data.euro.who.int/hfadb, accessed 20 August 2012).
ALB	Albania	1997	1997	10.0	European Health for All Database (HFA-DB) [online database]. World Health Organization (WHO) Regional Office for Europe; 2012 (http://data.euro.who.int/hfadb, accessed 20 August 2012).
ALB	Albania	1998	1998	10.1	European Health for All Database (HFA-DB) [online database]. World Health Organization (WHO) Regional Office for Europe; 2012 (http://data.euro.who.int/hfadb, accessed 20 August 2012).
ALB	Albania	2001	2001	13.1	European Health for All Database (HFA-DB) [online database]. World Health Organization (WHO) Regional Office for Europe; 2012 (http://data.euro.who.int/hfadb, accessed 20 August 2012).
ALB	Albania	1997	2002	13.4	Institute of Public Health, Albania Ministry of Health, Institute of Statistics, DRH/CDC, United States Agency for International Development (USAID), United Nations Population Fund, Albania (UNFPA), United Nations Children's Fund (UNICEF). Reproductive Health Survey Albania, 2002. Final Report. Atlanta: DHR/CDC; 2005.
ALB	Albania	2002	2002	16.5	European Health for All Database (HFA-DB) [online database]. World Health Organization (WHO) Regional Office for Europe; 2012 (http://data.euro.who.int/hfadb, accessed 20 August 2012).

ISO Code	Country	Coverage start year	Coverage end year	Caesarean section rate (%)	References
ALB	Albania	2003	2003	15.1	European Health for All Database (HFA-DB) [online database]. World Health Organization (WHO) Regional Office for Europe; 2012 (http://data.euro.who.int/hfadb, accessed 20 August 2012).
ALB	Albania	2004	2004	18.0	European Health for All Database (HFA-DB) [online database]. World Health Organization (WHO) Regional Office for Europe; 2012 (http://data.euro.who.int/hfadb, accessed 20 August 2012).
ALB	Albania	2005	2005	20.3	European Health for All Database (HFA-DB) [online database]. World Health Organization (WHO) Regional Office for Europe; 2012 (http://data.euro.who.int/hfadb, accessed 20 August 2012).
ALB	Albania	2007	2007	25.6	European Health for All Database (HFA-DB) [online database]. World Health Organization (WHO) Regional Office for Europe; 2012 (http://data.euro.who.int/hfadb, accessed 20 August 2012).
ALB	Albania	2008	2008	22.7	European Health for All Database (HFA-DB) [online database]. World Health Organization (WHO) Regional Office for Europe; 2012 (http://data.euro.who.int/hfadb, accessed 20 August 2012).
ALB	Albania	2003	2009	18.7	Institute of Statistics, Institute of Public Health [Albania], ICF Macro. Albania Demographic and Health Survey 2008-09. Tirana, Albania: Institute of Statistics, Institute of Public Health, ICF Macro; 2010.
ALB	Albania	2009	2009	28.1	European Health for All Database (HFA-DB) [online database]. World Health Organization (WHO) Regional Office for Europe; 2012 (http://data.euro.who.int/hfadb, accessed 20 August 2012).
ALB	Albania	2010	2010	29.5	European Health for All Database (HFA-DB) [online database]. World Health Organization (WHO) Regional Office for Europe; 2016 (http://data.euro.who.int/hfadb, accessed 8 August 2016).

ISO Code	Country	Coverage start year	Coverage end year	Caesarean section rate (%)	References
ALB	Albania	2011	2011	30.0	European Health for All Database (HFA-DB) [online database]. World Health Organization (WHO) Regional Office for Europe; 2016 (http://data.euro.who.int/hfadb, accessed 8 August 2016).
ALB	Albania	2012	2012	32.1	European Health for All Database (HFA-DB) [online database]. World Health Organization (WHO) Regional Office for Europe; 2016 (http://data.euro.who.int/hfadb, accessed 8 August 2016).
ALB	Albania	2013	2013	32.3	European Health for All Database (HFA-DB) [online database]. World Health Organization (WHO) Regional Office for Europe; 2016 (http://data.euro.who.int/hfadb, accessed 8 August 2016).
ALB	Albania	2012	2018	31	Albania Demographic and Health Survey 2017-18. Tirana: Institute of Statistics, Institute of Public Health, ICF; 2018.
DZA	Algeria	1987	1992	6.3	Enquête Algérienne sur la Santé de la Mère et de l'Enfant. Rapport Principal. Alger: Ministère de la Santé et de la Population, Office National des Statistique, Ligue des Etats Arabes; 1993 (in French).
DZA	Algeria	2010	2013	16.0	Enquête par Grappes à Indicateurs Multiples (MICS) [Multiple Indicator Cluster Survey (MICS)] République Algérienne Démocratique et Populaire 2012-2013. Rapport final. Algérie: Ministère de la Santé, de la Population et de la Réforme Hospitalière, Fonds des Nations unies pour l'enfance (UNICEF), Fonds des Nations Unies pour la Population (FNUAP/UNFPA); 2015 (in French).
AND	Andorra	1996	1996	17.6	European Health for All Database (HFA-DB) [online database]. World Health Organization (WHO) Regional Office for Europe; 2012 (http://data.euro.who.int/hfadb, accessed 20 August 2012).
AND	Andorra	1997	1997	19.5	European Health for All Database (HFA-DB) [online database]. World Health Organization (WHO) Regional Office for Europe; 2012 (http://data.euro.who.int/hfadb, accessed 20 August 2012).

ISO Code	Country	Coverage start year	Coverage end year	Caesarean section rate (%)	References
AND	Andorra	1998	1998	20.1	European Health for All Database (HFA-DB) [online database]. World Health Organization (WHO) Regional Office for Europe; 2012 (http://data.euro.who.int/hfadb, accessed 20 August 2012).
AND	Andorra	1999	1999	23.7	European Health for All Database (HFA-DB) [online database]. World Health Organization (WHO) Regional Office for Europe; 2012 (http://data.euro.who.int/hfadb, accessed 20 August 2012).
AGO	Angola	2010	2016	3.7	Instituto Nacional de Estatística (INE), Ministério da Saúde (MINSA), Ministério do Planeamento e do Desenvolvimento Territorial (MINPLAN), ICF. Inquérito de Indicadores Múltiplos e de Saúde em Angola 2015-2016. Luanda, Angola e Rockville, Maryland, EUA: INE, MINSA, MINPLAN, ICF; 2017 (in Portuguese).
ARG	Argentina	1999	2005	33.0	Encuesta Nacional de Nutrición y Salud (ENNyS). Documento de Resultados 2007. Buenos Aires: Ministerio de Salud, Plan Federal de Salud [Argentina]; 2007 (in Spanish).
ARG	Argentina	2005	2005	22.9	Sistema Informático Perinatal de la República Argentina. Anuario 2005 de Información Perinatal. Buenos Aires: Ministerio de Salud, Dirección Nacional de Maternidad e Infancia [Argentina]; 2006 (in Spanish).
ARG	Argentina	2006	2006	22.7	Sistema Informático Perinatal de la República Argentina. Anuario 2006 de Información Perinatal. Buenos Aires: Ministerio de Salud, Dirección Nacional de Maternidad e Infancia [Argentina]; 2007 (in Spanish).
ARG	Argentina	2011	2011	29.1	Karolinski A, Mercer R, Salgado P, Ocampo C, Bolzán A, Nieto R et al. Primer informe nacional de relevamiento epidemiológico SIP-Gestión: Desarrollo e implementación a escala nacional de un sistema de información en salud de la mujer y perinatal en Argentina. Buenos Aires: Organización Panamericana de la Salud (OPS); Ministerio de Salud de la Nación [Argentina]; 2013 (in Spanish).
ARM	Armenia	1990	1990	3.4	European Health for All Database (HFA-DB) [online database]. World Health Organization (WHO) Regional Office for Europe; 2012 (http://data.euro.who.int/hfadb, accessed 20 August 2012).

ISO Code	Country	Coverage start year	Coverage end year	Caesarean section rate (%)	References
ARM	Armenia	1991	1991	3.6	European Health for All Database (HFA-DB) [online database]. World Health Organization (WHO) Regional Office for Europe; 2012 (http://data.euro.who.int/hfadb, accessed 20 August 2012).
ARM	Armenia	1992	1992	3.7	European Health for All Database (HFA-DB) [online database]. World Health Organization (WHO) Regional Office for Europe; 2012 (http://data.euro.who.int/hfadb, accessed 20 August 2012).
ARM	Armenia	1993	1993	3.8	European Health for All Database (HFA-DB) [online database]. World Health Organization (WHO) Regional Office for Europe; 2012 (http://data.euro.who.int/hfadb, accessed 20 August 2012).
ARM	Armenia	1994	1994	3.7	European Health for All Database (HFA-DB) [online database]. World Health Organization (WHO) Regional Office for Europe; 2012 (http://data.euro.who.int/hfadb, accessed 20 August 2012).
ARM	Armenia	1995	1995	4.3	European Health for All Database (HFA-DB) [online database]. World Health Organization (WHO) Regional Office for Europe; 2012 (http://data.euro.who.int/hfadb, accessed 20 August 2012).
ARM	Armenia	1996	1996	4.8	European Health for All Database (HFA-DB) [online database]. World Health Organization (WHO) Regional Office for Europe; 2012 (http://data.euro.who.int/hfadb, accessed 20 August 2012).
ARM	Armenia	1999	1999	6.6	European Health for All Database (HFA-DB) [online database]. World Health Organization (WHO) Regional Office for Europe; 2012 (http://data.euro.who.int/hfadb, accessed 20 August 2012).
ARM	Armenia	2000	2000	7.4	European Health for All Database (HFA-DB) [online database]. World Health Organization (WHO) Regional Office for Europe; 2012 (http://data.euro.who.int/hfadb, accessed 20 August 2012).

ISO Code	Country	Coverage start year	Coverage end year	Caesarean section rate (%)	References
ARM	Armenia	1995	2000	6.6	National Statistical Service [Armenia], Ministry of Health [Armenia], ORC Macro. Armenia Demographic and Health Survey 2000. Calverton, Maryland: National Statistical Service, Ministry of Health, ORC Macro; 2001.
ARM	Armenia	2001	2001	7.9	European Health for All Database (HFA-DB) [online database]. World Health Organization (WHO) Regional Office for Europe; 2012 (http://data.euro.who.int/hfadb, accessed 20 August 2012).
ARM	Armenia	2004	2004	9.6	European Health for All Database (HFA-DB) [online database]. World Health Organization (WHO) Regional Office for Europe; 2012 (http://data.euro.who.int/hfadb, accessed 20 August 2012).
ARM	Armenia	2005	2005	11.3	European Health for All Database (HFA-DB) [online database]. World Health Organization (WHO) Regional Office for Europe; 2012 (http://data.euro.who.int/hfadb, accessed 20 August 2012).
ARM	Armenia	2000	2005	9.0	National Statistical Service [Armenia], Ministry of Health [Armenia], ORC Macro. Armenia Demographic and Health Survey 2005. Calverton, Maryland: National Statistical Service, Ministry of Health, ORC Macro; 2006.
ARM	Armenia	2006	2006	11.3	European Health for All Database (HFA-DB) [online database]. World Health Organization (WHO) Regional Office for Europe; 2012 (http://data.euro.who.int/hfadb, accessed 20 August 2012).
ARM	Armenia	2009	2009	17.7	European Health for All Database (HFA-DB) [online database]. World Health Organization (WHO) Regional Office for Europe; 2012 (http://data.euro.who.int/hfadb, accessed 20 August 2012).
ARM	Armenia	2005	2010	12.5	National Statistical Service [Armenia], Ministry of Health [Armenia], ICF International. Armenia Demographic and Health Survey 2010. Calverton, Maryland: National Statistical Service, Ministry of Health, ICF International; 2012.

ISO Code	Country	Coverage start year	Coverage end year	Caesarean section rate (%)	References
ARM	Armenia	2010	2010	18.8	European Health for All Database (HFA-DB) [online database]. World Health Organization (WHO) Regional Office for Europe; 2016 (http://data.euro.who.int/hfadb, accessed 8 August 2016).
ARM	Armenia	2011	2011	21.7	European Health for All Database (HFA-DB) [online database]. World Health Organization (WHO) Regional Office for Europe; 2016 (http://data.euro.who.int/hfadb, accessed 8 August 2016).
ARM	Armenia	2012	2012	22.5	European Health for All Database (HFA-DB) [online database]. World Health Organization (WHO) Regional Office for Europe; 2016 (http://data.euro.who.int/hfadb, accessed 8 August 2016).
ARM	Armenia	2014	2014	25.9	European Health for All Database (HFA-DB) [online database]. World Health Organization (WHO) Regional Office for Europe; 2016 (http://data.euro.who.int/hfadb, accessed 8 August 2016).
ARM	Armenia	2015	2015	27.5	European Health Information Gateway. European Health for All database (HFA-DB). Caesarean sections per 1000 live births [online database]. World Health Organization (WHO) Regional Office for Europe; 2018 (https://gateway.euro.who.int/en/indicators/hfa_596-7060-caesarean-sections-per-1000-live-births/, accessed 14 February 2019).
ARM	Armenia	2010	2016	18.0	National Statistical Service [Armenia], Ministry of Health [Armenia], ICF. Armenia Demographic and Health Survey 2015-16. Rockville, Maryland, USA: National Statistical Service, Ministry of Health, ICF; 2017.
AUS	Australia	1991	1991	18.0	Lancaster P, Huang J, Pedisich E. Australia's mothers and babies 1991. Perinatal Statistics Series No. 1. Sydney: Australian Institute of Health and Welfare (AIHW) National Perinatal Statistics Unit; 1994.
AUS	Australia	1992	1992	18.3	Lancaster P, Huang J, Pedisich E. Australia's mothers and babies 1992. Perinatal Statistics Series No. 2. Sydney: Australian Institute of Health and Welfare (AIHW) National Perinatal Statistics Unit; 1995.

ISO Code	Country	Coverage start year	Coverage end year	Caesarean section rate (%)	References
AUS	Australia	1993	1993	19.0	Lancaster P, Huang J, Lin M. Australia's mothers and babies 1993. Perinatal Statistics Series No. 3. Sydney: Australian Institute of Health and Welfare (AIHW) National Perinatal Statistics Unit; 1996.
AUS	Australia	1994	1994	19.4	Day P, Lancaster P, Huang J. Australia's mothers and babies 1994. Perinatal Statistics Series No. 5. Sydney: Australian Institute of Health and Welfare (AIHW) National Perinatal Statistics Unit; 1997.
AUS	Australia	1995	1995	19.3	Day P, Lancaster P, Huang J. Australia's mothers and babies 1995. Perinatal Statistics Series No. 6. Sydney: Australian Institute of Health and Welfare (AIHW) National Perinatal Statistics Unit; 1997.
AUS	Australia	1996	1996	19.5	Day P, Sullivan EA, Lancaster P. Australia's mothers and babies 1996. Perinatal Statistics Series No. 7. AIHW Cat. No. PER 4. Sydney: Australian Institute of Health and Welfare (AIHW) National Perinatal Statistics Unit; 1999.
AUS	Australia	1997	1997	20.3	Day P, Sullivan EA, Ford J, Lancaster P. Australia's mothers and babies 1997. Perinatal Statistics Series no.9. AIHW Cat. No. PER 12. Sydney: Australian Institute of Health and Welfare (AIHW) National Perinatal Statistics Unit; 1999.
AUS	Australia	1998	1998	21.1	Nassar N, Sullivan EA, Lancaster P, Day P. Australia's mothers and babies 1998. Perinatal Statistics Series no. 10 AIHW Cat. No. PER 15. Sydney: Australian Institute of Health and Welfare (AIHW) National Perinatal Statistics Unit; 2000.
AUS	Australia	1999	1999	21.9	Nassar N, Sullivan EA. Australia’s mothers and babies 1999. Perinatal Statistics Series no. 11. AIHW Cat. No. PER 19. Sydney, Australia: Australian Institute of Health and Welfare (AIHW) National Perinatal Statistics Unit; 2001.
AUS	Australia	2000	2000	23.3	Australia’s mothers and babies 2000. Perinatal Statistics Series no. 12. AIHW Cat. No. PER 21. Canberra: Australian Institute of Health and Welfare National Perinatal Statistics Unit (AIHW NPSU); 2003.

Betran AP, *et al. BMJ Global Health* 2021; 6:e005671. doi: 10.1136/bmjgh-2021-005671

ISO Code	Country	Coverage start year	Coverage end year	Caesarean section rate (%)	References
AUS	Australia	2001	2001	25.4	Laws PJ, Sullivan EA. Australia's mothers and babies 2001. Perinatal Statistics Series No. 13. AIHW Cat. No. PER 25. Sydney: Australian Institute of Health and Welfare (AIHW) National Perinatal Statistics Unit; 2004.
AUS	Australia	2002	2002	27.0	Laws PJ, Sullivan EA. Australia's mothers and babies 2002. Perinatal Statistics Series No. 15. AIHW Cat. No. PER 28. Sydney: Australian Institute of Health and Welfare (AIHW) National Perinatal Statistics Unit; 2004.
AUS	Australia	2003	2003	28.5	Laws PJ, Sullivan EA. Australia's mothers and babies 2003. Perinatal Statistics Series No. 16. AIHW Cat. No. PER 29. Sydney: Australian Institute of Health and Welfare (AIHW) National Perinatal Statistics Unit; 2005.
AUS	Australia	2004	2004	29.4	Laws PJ, Grayson N, Sullivan EA. Australia's mothers and babies 2004. Perinatal statistics series no. 18. AIHW cat. no. PER 34. Sydney: Australian Institute of Health and Welfare (AIHW) National Perinatal Statistics Unit; 2006.
AUS	Australia	2005	2005	30.3	Laws PJ, Abeywardana S, Walker J, Sullivan EA. Australia's mothers and babies 2005. Perinatal statistics series no. 20. Cat. no. PER 40. Sydney: Australian Institute of Health and Welfare (AIHW) National Perinatal Statistics Unit; 2007.
AUS	Australia	2006	2006	30.8	Laws PJ, Hilder L. Australia's mothers and babies 2006. Perinatal statistics series no. 22. Cat. no. PER 46. Sydney: Australian Institute of Health and Welfare (AIHW) National Perinatal Statistics Unit; 2008.
AUS	Australia	2007	2007	30.9	Laws P, Sullivan EA. Australia's mothers and babies 2007. Perinatal statistics series no. 23. Cat. no. PER 48. Sydney: Australian Institute of Health and Welfare (AIHW) National Perinatal Statistics Unit; 2009.
AUS	Australia	2008	2008	31.1	Laws PJ, Li Z, Sullivan EA. Australia's mothers and babies 2008. Perinatal statistics series no. 24. Cat. No. PER 50. Canberra: Australian Institute of Health and Welfare (AIHW); 2010.

ISO Code	Country	Coverage start year	Coverage end year	Caesarean section rate (%)	References
AUS	Australia	2009	2009	31.5	Li Z, McNally L, Hilder L, Sullivan EA. Australia's mothers and babies 2009. Perinatal statistics series no. 25. Cat. no. PER 52. Sydney: Australian Institute of Health and Welfare (AIHW) National Perinatal Epidemiology and Statistics Unit; 2011.
AUS	Australia	2010	2010	31.6	Li Z, Zeki R, Hilder L, Sullivan EA. Australia's mothers and babies 2010. Perinatal statistics series no. 27. Cat. no. PER 57. Canberra: Australian Institute of Health and Welfare (AIHW) National Perinatal Epidemiology and Statistics Unit; 2012.
AUS	Australia	2011	2011	32.3	Li Z, Zeki R, Hilder L, Sullivan EA. Australia's mothers and babies 2011. Perinatal statistics series no. 28. Cat. no. PER 59. Canberra: Australian Institute of Health and Welfare (AIHW) National Perinatal Epidemiology and Statistics Unit; 2013.
AUS	Australia	2012	2012	32.4	Hilder L, Zhichao Z, Parker M, Jahan S, Chambers GM. Australia's mothers and babies 2012. Perinatal statistics series no. 30. Cat no. PER 69. Canberra: Australian Institute of Health and Welfare (AIHW); 2014.
AUS	Australia	2013	2013	33.0	Australia's mothers and babies 2013—in brief. Perinatal statistics series no. 31. Cat no. PER 72. Canberra: Australian Institute of Health and Welfare (AIHW); 2015.
AUS	Australia	2014	2014	33.1	Australia's mothers and babies 2014—in brief. Perinatal statistics series no. 32. Cat no. PER 87. Canberra: Australian Institute of Health and Welfare (AIHW); 2016.
AUS	Australia	2015	2015	33.3	Australia's mothers and babies 2015—in brief. Perinatal statistics series no. 33. Cat no. PER 91. Canberra: Australian Institute of Health and Welfare (AIHW); 2017.
AUS	Australia	2016	2016	33.8	Australia's mothers and babies 2016 - in brief. Perinatal statistics series no. 34. Cat no. PER 97. Canberra: Australian Institute of Health and Welfare (AIHW); 2018.

ISO Code	Country	Coverage start year	Coverage end year	Caesarean section rate (%)	References
AUS	Australia	2017	2017	34.6	Australia's mothers and babies 2017 - in brief. Perinatal statistics series no. 35. Cat no. PER 100. Canberra: Australian Institute of Health and Welfare (AIHW); 2019.
AUT	Austria	1995	1995	12.4	European Health for All Database (HFA-DB) [online database]. World Health Organization (WHO) Regional Office for Europe; 2012 (http://data.euro.who.int/hfadb, accessed 20 August 2012).
AUT	Austria	1996	1996	13.1	European Health for All Database (HFA-DB) [online database]. World Health Organization (WHO) Regional Office for Europe; 2012 (http://data.euro.who.int/hfadb, accessed 20 August 2012).
AUT	Austria	1997	1997	13.9	European Health for All Database (HFA-DB) [online database]. World Health Organization (WHO) Regional Office for Europe; 2012 (http://data.euro.who.int/hfadb, accessed 20 August 2012).
AUT	Austria	1998	1998	14.6	European Health for All Database (HFA-DB) [online database]. World Health Organization (WHO) Regional Office for Europe; 2012 (http://data.euro.who.int/hfadb, accessed 20 August 2012).
AUT	Austria	1999	1999	16.4	European Health for All Database (HFA-DB) [online database]. World Health Organization (WHO) Regional Office for Europe; 2012 (http://data.euro.who.int/hfadb, accessed 20 August 2012).
AUT	Austria	2000	2000	17.2	Vital statistics. Births since 2000 by selected characteristics [website]. Vienna: Statistics Austria; 2011 (http://www.statistik.at/web_en/statisticspopulation-births-index.html, accessed 19 May 2011).
AUT	Austria	2001	2001	18.9	Vital statistics. Births since 2000 by selected characteristics [website]. Vienna: Statistics Austria; 2011 (http://www.statistik.at/web_en/statisticspopulation-births-index.html, accessed 19 May 2011).

ISO Code	Country	Coverage start year	Coverage end year	Caesarean section rate (%)	References
AUT	Austria	2002	2002	20.6	Vital statistics. Births since 2000 by selected characteristics [website]. Vienna: Statistics Austria; 2011 (http://www.statistik.at/web_en/statisticspopulation-births-index.html, accessed 19 May 2011).
AUT	Austria	2003	2003	22.1	Vital statistics. Births since 2003 by selected characteristics [website]. Vienna: Statistics Austria; 2014 (http://www.statistik.at/web_en/statisticspopulation-births-index.html, accessed 28 May 2014).
AUT	Austria	2004	2004	23.6	Vital statistics. Births since 2003 by selected characteristics [website]. Vienna: Statistics Austria; 2014 (http://www.statistik.at/web_en/statisticspopulation-births-index.html, accessed 28 May 2014).
AUT	Austria	2005	2005	24.4	Vital statistics. Births since 2003 by selected characteristics [website]. Vienna: Statistics Austria; 2014 (http://www.statistik.at/web_en/statisticspopulation-births-index.html, accessed 28 May 2014).
AUT	Austria	2006	2006	25.8	Vital statistics. Births since 2003 by selected characteristics [website]. Vienna: Statistics Austria; 2014 (http://www.statistik.at/web_en/statisticspopulation-births-index.html, accessed 28 May 2014).
AUT	Austria	2007	2007	27.1	Vital statistics. Births since 2003 by selected characteristics [website]. Vienna: Statistics Austria; 2014 (http://www.statistik.at/web_en/statisticspopulation-births-index.html, accessed 28 May 2014).
AUT	Austria	2008	2008	28.0	Vital statistics. Births since 2003 by selected characteristics [website]. Vienna: Statistics Austria; 2014 (http://www.statistik.at/web_en/statisticspopulation-births-index.html, accessed 28 May 2014).
AUT	Austria	2009	2009	28.8	Vital statistics. Births since 2003 by selected characteristics [website]. Vienna: Statistics Austria; 2014 (http://www.statistik.at/web_en/statisticspopulation-births-index.html, accessed 28 May 2014).

ISO Code	Country	Coverage start year	Coverage end year	Caesarean section rate (%)	References
AUT	Austria	2010	2010	28.9	Vital statistics. Births since 2003 by selected characteristics [website]. Vienna: Statistics Austria; 2014 (http://www.statistik.at/web_en/statisticspopulation-births-index.html, accessed 28 May 2014).
AUT	Austria	2011	2011	29.0	Vital statistics. Births since 2003 by selected characteristics [website]. Vienna: Statistics Austria; 2014 (http://www.statistik.at/web_en/statisticspopulation-births-index.html, accessed 28 May 2014).
AUT	Austria	2012	2012	28.8	European Health for All Database (HFA-DB) [online database]. World Health Organization (WHO) Regional Office for Europe; 2016 (http://data.euro.who.int/hfadb, accessed 8 August 2016).
AUT	Austria	2013	2013	29.3	Vital statistics. Births since 2003 by selected characteristics [website]. Vienna: Statistics Austria; 2014 (http://www.statistik.at/web_en/statisticspopulation-births-index.html, accessed 28 May 2014).
AUT	Austria	2014	2014	29.3	Vital statistics. Births since 2003 by selected characteristics [website]. Vienna: Statistics Austria; 2014 (http://www.statistik.at/web_en/statisticspopulation-births-index.html, accessed 28 May 2014).
AUT	Austria	2014	2014	29.3	European Health for All Database (HFA-DB) [online database]. World Health Organization (WHO) Regional Office for Europe; 2016 (http://data.euro.who.int/hfadb, accessed 8 August 2016).
AUT	Austria	2015	2015	29.7	Vital statistics. Live births (with place of birth in Austria) by medical characteristics since 2006 [website]. Vienna: Statistics Austria; 2017 (http://www.statistik.at/web_en/statistics/PeopleSociety/health/medical_and_sociomedical_characteristics_of_live_birth/110639.html, accessed 27 October 2017).
AUT	Austria	2016	2016	29.5	Vital statistics. Live births (with place of birth in Austria) by medical characteristics since 2006 [website]. Vienna: Statistics Austria; 2017 (http://www.statistik.at/web_en/statistics/PeopleSociety/health/medical_and_sociomedical_characteristics_of_live_birth/110639.html, accessed 27 October 2017).

ISO Code	Country	Coverage start year	Coverage end year	Caesarean section rate (%)	References
AUT	Austria	2017	2017	29.6	Vital statistics. Live births (with place of birth in Austria) by medical characteristics since 2007 [website]. Vienna: Statistics Austria; 2018 (http://www.statistik.at/web_en/statistics/PeopleSociety/health/medical_and_sociomedical_characteristics_of_live_birth/110639.html, accessed 11 September 2018).
AUT	Austria	2018	2018	29.4	Vital statistics. Live births (with place of birth in Austria) by medical characteristics since 2008 [website]. Vienna: Statistics Austria; 2019 (http://www.statistik.at/web_en/statistics/PeopleSociety/health/medical_and_sociomedical_characteristics_of_live_birth/110639.html, accessed 10 February 2020).
AZE	Azerbaijan	1990	1990	1.4	European Health for All Database (HFA-DB) [online database]. World Health Organization (WHO) Regional Office for Europe; 2012 (http://data.euro.who.int/hfadb, accessed 20 August 2012).
AZE	Azerbaijan	1991	1991	1.6	European Health for All Database (HFA-DB) [online database]. World Health Organization (WHO) Regional Office for Europe; 2012 (http://data.euro.who.int/hfadb, accessed 20 August 2012).
AZE	Azerbaijan	1992	1992	1.7	European Health for All Database (HFA-DB) [online database]. World Health Organization (WHO) Regional Office for Europe; 2012 (http://data.euro.who.int/hfadb, accessed 20 August 2012).
AZE	Azerbaijan	1993	1993	1.7	European Health for All Database (HFA-DB) [online database]. World Health Organization (WHO) Regional Office for Europe; 2012 (http://data.euro.who.int/hfadb, accessed 20 August 2012).
AZE	Azerbaijan	1994	1994	1.8	European Health for All Database (HFA-DB) [online database]. World Health Organization (WHO) Regional Office for Europe; 2012 (http://data.euro.who.int/hfadb, accessed 20 August 2012).
AZE	Azerbaijan	1995	1995	1.9	European Health for All Database (HFA-DB) [online database]. World Health Organization (WHO) Regional Office for Europe; 2012 (http://data.euro.who.int/hfadb, accessed 20 August 2012).

ISO Code	Country	Coverage start year	Coverage end year	Caesarean section rate (%)	References
AZE	Azerbaijan	1996	1996	2.0	European Health for All Database (HFA-DB) [online database]. World Health Organization (WHO) Regional Office for Europe; 2012 (http://data.euro.who.int/hfadb, accessed 20 August 2012).
AZE	Azerbaijan	1997	1997	2.0	European Health for All Database (HFA-DB) [online database]. World Health Organization (WHO) Regional Office for Europe; 2012 (http://data.euro.who.int/hfadb, accessed 20 August 2012).
AZE	Azerbaijan	2000	2000	2.6	European Health for All Database (HFA-DB) [online database]. World Health Organization (WHO) Regional Office for Europe; 2012 (http://data.euro.who.int/hfadb, accessed 20 August 2012).
AZE	Azerbaijan	2001	2001	3.1	European Health for All Database (HFA-DB) [online database]. World Health Organization (WHO) Regional Office for Europe; 2012 (http://data.euro.who.int/hfadb, accessed 20 August 2012).
AZE	Azerbaijan	1996	2001	2.6	Ministry of Health [Azerbaijan], Division of Reproductive Health - Centers for Disease Control and Prevention (DRH/CDC). Reproductive Health Survey Azerbaijan, 2001. Final Report. Atlanta: CDC; 2003.
AZE	Azerbaijan	2002	2002	3.6	European Health for All Database (HFA-DB) [online database]. World Health Organization (WHO) Regional Office for Europe; 2012 (http://data.euro.who.int/hfadb, accessed 20 August 2012).
AZE	Azerbaijan	2004	2004	4.7	European Health for All Database (HFA-DB) [online database]. World Health Organization (WHO) Regional Office for Europe; 2012 (http://data.euro.who.int/hfadb, accessed 20 August 2012).
AZE	Azerbaijan	2005	2005	5.3	European Health for All Database (HFA-DB) [online database]. World Health Organization (WHO) Regional Office for Europe; 2012 (http://data.euro.who.int/hfadb, accessed 20 August 2012).

ISO Code	Country	Coverage start year	Coverage end year	Caesarean section rate (%)	References
AZE	Azerbaijan	2006	2006	6.1	European Health for All Database (HFA-DB) [online database]. World Health Organization (WHO) Regional Office for Europe; 2012 (http://data.euro.who.int/hfadb, accessed 20 August 2012).
AZE	Azerbaijan	2001	2006	4.7	State Statistical Committee (SSC) [Azerbaijan], Macro International Inc. Azerbaijan Demographic and Health Survey 2006. Calverton, Maryland, USA: State Statistical Committee, Macro International Inc.; 2008.
AZE	Azerbaijan	2007	2007	7.6	European Health for All Database (HFA-DB) [online database]. World Health Organization (WHO) Regional Office for Europe; 2012 (http://data.euro.who.int/hfadb, accessed 20 August 2012).
AZE	Azerbaijan	2010	2010	13.7	European Health for All Database (HFA-DB) [online database]. World Health Organization (WHO) Regional Office for Europe; 2016 (http://data.euro.who.int/hfadb, accessed 8 August 2016).
AZE	Azerbaijan	2006	2011	20.0	Public Health and Reforms Centre (PHRC) [Azerbaijan]. Demographic and Health Survey, Azerbaijan, 2011. Baku: Ministry of Health (MoH) [Azerbaijan]; 2013.
AZE	Azerbaijan	2011	2011	14.7	European Health for All Database (HFA-DB) [online database]. World Health Organization (WHO) Regional Office for Europe; 2014 (http://data.euro.who.int/hfadb, accessed 15 December 2014).
AZE	Azerbaijan	2012	2012	20.5	Healthcare, social security and housing conditions in Azerbaijan. Baku: State Statistical Committee of the Republic of Azerbaijan; 2013.
AZE	Azerbaijan	2013	2013	17.0	European Health for All Database (HFA-DB) [online database]. World Health Organization (WHO) Regional Office for Europe; 2016 (http://data.euro.who.int/hfadb, accessed 30 June 2016).

ISO Code	Country	Coverage start year	Coverage end year	Caesarean section rate (%)	References
AZE	Azerbaijan	2014	2014	18.3	European Health for All Database (HFA-DB) [online database]. World Health Organization (WHO) Regional Office for Europe; 2016 (http://data.euro.who.int/hfadb, accessed 8 August 2016).
AZE	Azerbaijan	2015	2015	27.6	European Health for All Database (HFA-DB) [online database]. World Health Organization (WHO) Regional Office for Europe; 2016 (http://data.euro.who.int/hfadb, accessed 8 August 2016).
AZE	Azerbaijan	2017	2017	33.5	European Health Information Gateway. European Health for All database (HFA-DB). Caesarean sections per 1000 live births [online database]. World Health Organization (WHO) Regional Office for Europe; 2019 (https://gateway.euro.who.int/en/indicators/hfa_596-7060-caesarean-sections-per-1000-live-births/, accessed 5 March 2020).
BHR	Bahrain	1992	1995	16.0	Tawfeeq N, Farid SM. Bahrain Family Health Survey 1995. Principal Report. Manama: Ministry of Health, Bahrain; 2000.
BHR	Bahrain	2004	2004	16.2	Health Statistics of 2008. Bahrain: Ministry of Health; 2009.
BHR	Bahrain	2005	2005	19.8	Health Statistics of 2008. Bahrain: Ministry of Health; 2009.
BHR	Bahrain	2006	2006	20.9	Health Statistics of 2010. Bahrain: Ministry of Health; 2011.
BHR	Bahrain	2007	2007	23.4	Health Statistics of 2010. Bahrain: Ministry of Health; 2011.

ISO Code	Country	Coverage start year	Coverage end year	Caesarean section rate (%)	References
BHR	Bahrain	2008	2008	23.9	Health Statistics of 2010. Bahrain: Ministry of Health; 2011.
BHR	Bahrain	2009	2009	23.6	Health Statistics of 2010. Bahrain: Ministry of Health; 2011.
BHR	Bahrain	2010	2010	26.8	Health Statistics of 2010. Bahrain: Ministry of Health; 2011.
BHR	Bahrain	2011	2011	30.0	Health Statistics of 2011. Bahrain: Ministry of Health; 2012.
BHR	Bahrain	2012	2012	26.0	Health Statistics of 2012. Bahrain: Ministry of Health; 2013.
BHR	Bahrain	2013	2013	24.0	Health Statistics of 2015. Bahrain: Ministry of Health; 2016.
BHR	Bahrain	2014	2014	27.0	Health Statistics of 2015. Bahrain: Ministry of Health; 2016.
BHR	Bahrain	2015	2015	28.0	Health Statistics of 2015. Bahrain: Ministry of Health; 2016.

ISO Code	Country	Coverage start year	Coverage end year	Caesarean section rate (%)	References
BHR	Bahrain	2016	2016	28.0	Health Statistics of 2016. Bahrain: Ministry of Health; 2017.
BHR	Bahrain	2017	2017	29.0	Health Statistics of 2017. Bahrain: Ministry of Health; 2018.
BGD	Bangladesh	1994	2000	2.4	National Institute of Population Research and Training (NIPORT), Mitra and Associates (MA), ORC Macro (ORCM). Bangladesh Demographic and Health Survey 1999-2000. Dhaka, Bangladesh and Calverton, Maryland [USA]: National Institute of Population Research and Training, Mitra and Associates, ORC Macro; 2001.
BGD	Bangladesh	1998	2001	2.7	Bangladesh Maternal Health Services and Maternal Mortality Survey 2001. Dhaka: National Institute of Population Research and Training - NIPORT/Bangladesh, ORC Macro, Johns Hopkins University, Centre for Health and Population Research - icddr,b/Bangladesh; 2003.
BGD	Bangladesh	1999	2004	3.5	National Institute of Population Research and Training (NIPORT), Mitra and Associates, ORC Macro. Bangladesh Demographic and Health Survey 2004. Dhaka, Bangladesh and Calverton, Maryland [USA]: National Institute of Population Research and Training, Mitra and Associates, ORC Macro; 2005.
BGD	Bangladesh	2002	2007	7.5	National Institute of Population Research and Training (NIPORT), Mitra and Associates, Macro International. Bangladesh Demographic and Health Survey 2007. Dhaka, Bangladesh and Calverton, Maryland, USA: National Institute of Population Research and Training, Mitra and Associates, Macro International; 2009.
BGD	Bangladesh	2005	2010	12.2	Bangladesh Maternal Mortality and Health Care Survey 2010. Dhaka: National Institute of Population Research and Training (NIPORT), MEASURE Evaluation, icddr,b.; 2012.
BGD	Bangladesh	2008	2011	17.1	National Institute of Population Research and Training (NIPORT), Mitra and Associates, ICF International. Bangladesh Demographic and Health Survey 2011. Dhaka, Bangladesh and Calverton, Maryland, USA: NIPORT, Mitra and Associates, ICF International; 2013.

ISO Code	Country	Coverage start year	Coverage end year	Caesarean section rate (%)	References
BGD	Bangladesh	2010	2013	19.1	Bangladesh Multiple Indicator Cluster Survey 2012-2013, Progotir Pathey: Final Report. Dhaka, Bangladesh: Bangladesh Bureau of Statistics (BBS), UNICEF Bangladesh; 2014.
BGD	Bangladesh	2013	2016	30.7	Bangladesh Maternal Mortality and Health Care Survey 2016: Final Report. Dhaka and Chapel Hill: National Institute of Population Research and Training (NIPORT), International Centre for Diarrhoeal Disease Research, Bangladesh (icddr,b), MEASURE Evaluation; 2019.
BGD	Bangladesh	2014	2018	32.7	Bangladesh Demographic and Health Survey 2017-18: Key Indicators. Dhaka, Rockville, maryland: National Institute of Population Research and Training (NIPORT), ICF; 2019.
BRB	Barbados	2010	2012	21.3	Barbados Statistical Service, 'Barbados Multiple Indicator Cluster Survey 2012: Final Report'. BSS, Bridgetown, Barbados: Barbados Statistical Service, United Nations Children's Fund (UNICEF), United Nations Population Fund (UNFPA), United Nations Entity for Gender Equality and the Empowerment of Women (UN Women); 2014.
BLR	Belarus	1993	1993	9.2	European Health for All Database (HFA-DB) [online database]. World Health Organization (WHO) Regional Office for Europe; 2012 (http://data.euro.who.int/hfadb, accessed 20 August 2012).
BLR	Belarus	1994	1994	9.7	European Health for All Database (HFA-DB) [online database]. World Health Organization (WHO) Regional Office for Europe; 2012 (http://data.euro.who.int/hfadb, accessed 20 August 2012).
BLR	Belarus	1995	1995	11.0	European Health for All Database (HFA-DB) [online database]. World Health Organization (WHO) Regional Office for Europe; 2012 (http://data.euro.who.int/hfadb, accessed 20 August 2012).
BLR	Belarus	1996	1996	12.0	European Health for All Database (HFA-DB) [online database]. World Health Organization (WHO) Regional Office for Europe; 2012 (http://data.euro.who.int/hfadb, accessed 20 August 2012).

ISO Code	Country	Coverage start year	Coverage end year	Caesarean section rate (%)	References
BLR	Belarus	1997	1997	13.9	European Health for All Database (HFA-DB) [online database]. World Health Organization (WHO) Regional Office for Europe; 2012 (http://data.euro.who.int/hfadb, accessed 20 August 2012).
BLR	Belarus	1998	1998	15.2	European Health for All Database (HFA-DB) [online database]. World Health Organization (WHO) Regional Office for Europe; 2012 (http://data.euro.who.int/hfadb, accessed 20 August 2012).
BLR	Belarus	1999	1999	15.6	European Health for All Database (HFA-DB) [online database]. World Health Organization (WHO) Regional Office for Europe; 2012 (http://data.euro.who.int/hfadb, accessed 20 August 2012).
BLR	Belarus	2000	2000	16.7	European Health for All Database (HFA-DB) [online database]. World Health Organization (WHO) Regional Office for Europe; 2012 (http://data.euro.who.int/hfadb, accessed 20 August 2012).
BLR	Belarus	2001	2001	16.4	European Health for All Database (HFA-DB) [online database]. World Health Organization (WHO) Regional Office for Europe; 2012 (http://data.euro.who.int/hfadb, accessed 20 August 2012).
BLR	Belarus	2002	2002	17.4	European Health for All Database (HFA-DB) [online database]. World Health Organization (WHO) Regional Office for Europe; 2012 (http://data.euro.who.int/hfadb, accessed 20 August 2012).
BLR	Belarus	2003	2003	17.8	European Health for All Database (HFA-DB) [online database]. World Health Organization (WHO) Regional Office for Europe; 2012 (http://data.euro.who.int/hfadb, accessed 20 August 2012).
BLR	Belarus	2004	2004	18.3	European Health for All Database (HFA-DB) [online database]. World Health Organization (WHO) Regional Office for Europe; 2012 (http://data.euro.who.int/hfadb, accessed 20 August 2012).

Betran AP, *et al. BMJ Global Health* 2021; 6:e005671. doi: 10.1136/bmjgh-2021-005671

ISO Code	Country	Coverage start year	Coverage end year	Caesarean section rate (%)	References
BLR	Belarus	2005	2005	18.9	Public Health in the Republic of Belarus. An Official Statistics Collection, 2011. Minsk: Public Health Ministry of the Republic of Belarus. Division of Methodology and Analysis of Medical Statistics; 2012.
BLR	Belarus	2006	2006	19.5	Public Health in the Republic of Belarus. An Official Statistics Collection, 2011. Minsk: Public Health Ministry of the Republic of Belarus. Division of Methodology and Analysis of Medical Statistics; 2012.
BLR	Belarus	2007	2007	20.5	Public Health in the Republic of Belarus. An Official Statistics Collection, 2011. Minsk: Public Health Ministry of the Republic of Belarus. Division of Methodology and Analysis of Medical Statistics; 2012.
BLR	Belarus	2008	2008	21.0	Public Health in the Republic of Belarus. An Official Statistics Collection, 2011. Minsk: Public Health Ministry of the Republic of Belarus. Division of Methodology and Analysis of Medical Statistics; 2012.
BLR	Belarus	2009	2009	22.0	Public Health in the Republic of Belarus. An Official Statistics Collection, 2011. Minsk: Public Health Ministry of the Republic of Belarus. Division of Methodology and Analysis of Medical Statistics; 2012.
BLR	Belarus	2010	2010	23.0	Public Health in the Republic of Belarus. An Official Statistics Collection, 2011. Minsk: Public Health Ministry of the Republic of Belarus. Division of Methodology and Analysis of Medical Statistics; 2012.
BLR	Belarus	2011	2011	24.0	European Health for All Database (HFA-DB) [online database]. World Health Organization (WHO) Regional Office for Europe; 2016 (http://data.euro.who.int/hfadb, accessed 8 August 2016).
BLR	Belarus	2012	2012	25.3	European Health for All Database (HFA-DB) [online database]. World Health Organization (WHO) Regional Office for Europe; 2016 (http://data.euro.who.int/hfadb, accessed 8 August 2016).

ISO Code	Country	Coverage start year	Coverage end year	Caesarean section rate (%)	References
BLR	Belarus	2013	2013	26.6	European Health for All Database (HFA-DB) [online database]. World Health Organization (WHO) Regional Office for Europe; 2016 (http://data.euro.who.int/hfadb, accessed 8 August 2016).
BLR	Belarus	2014	2014	27.1	European Health for All Database (HFA-DB) [online database]. World Health Organization (WHO) Regional Office for Europe; 2016 (http://data.euro.who.int/hfadb, accessed 8 August 2016).
BLR	Belarus	2017	2017	29.6	European Health Information Gateway. European Health for All database (HFA-DB). Caesarean sections per 1000 live births [online database]. World Health Organization (WHO) Regional Office for Europe; 2019 (https://gateway.euro.who.int/en/indicators/hfa_596-7060-caesarean-sections-per-1000-live-births/, accessed 5 March 2020).
BEL	Belgium	1990	1990	10.9	Standardized Procedures for Mortality Analysis (SPMA). Number and proportion of live births by delivery for all Belgium, Belgium 1990-2008 [website]. Brussels: Scientific Institute of Public Health; 2009 (https://www.wiv-isp.be/epidemio/spma/, accessed 23 August 2012).
BEL	Belgium	1991	1991	11.5	Standardized Procedures for Mortality Analysis (SPMA). Number and proportion of live births by delivery for all Belgium, Belgium 1990-2008 [website]. Brussels: Scientific Institute of Public Health; 2009 (https://www.wiv-isp.be/epidemio/spma/, accessed 23 August 2012).
BEL	Belgium	1992	1992	11.9	Standardized Procedures for Mortality Analysis (SPMA). Number and proportion of live births by delivery for all Belgium, Belgium 1990-2008 [website]. Brussels: Scientific Institute of Public Health; 2009 (https://www.wiv-isp.be/epidemio/spma/, accessed 23 August 2012).
BEL	Belgium	1993	1993	12.9	Standardized Procedures for Mortality Analysis (SPMA). Number and proportion of live births by delivery for all Belgium, Belgium 1990-2008 [website]. Brussels: Scientific Institute of Public Health; 2009 (https://www.wiv-isp.be/epidemio/spma/, accessed 23 August 2012).
BEL	Belgium	1994	1994	12.9	Standardized Procedures for Mortality Analysis (SPMA). Number and proportion of live births by delivery for all Belgium, Belgium 1990-2008 [website]. Brussels: Scientific Institute of Public Health; 2009 (https://www.wiv-isp.be/epidemio/spma/, accessed 23 August 2012).

ISO Code	Country	Coverage start year	Coverage end year	Caesarean section rate (%)	References
BEL	Belgium	1995	1995	13.4	Standardized Procedures for Mortality Analysis (SPMA). Number and proportion of live births by delivery for all Belgium, Belgium 1990-2008 [website]. Brussels: Scientific Institute of Public Health; 2009 (https://www.wiv-isp.be/epidemio/spma/, accessed 23 August 2012).
BEL	Belgium	1996	1996	14.0	Standardized Procedures for Mortality Analysis (SPMA). Number and proportion of live births by delivery for all Belgium, Belgium 1990-2008 [website]. Brussels: Scientific Institute of Public Health; 2009 (https://www.wiv-isp.be/epidemio/spma/, accessed 23 August 2012).
BEL	Belgium	1997	1997	14.5	Standardized Procedures for Mortality Analysis (SPMA). Number and proportion of live births by delivery for all Belgium, Belgium 1990-2008 [website]. Brussels: Scientific Institute of Public Health; 2009 (https://www.wiv-isp.be/epidemio/spma/, accessed 23 August 2012).
BEL	Belgium	1998	1998	15.4	Standardized Procedures for Mortality Analysis (SPMA). Number and proportion of live births by delivery for all Belgium, Belgium 1990-2008 [website]. Brussels: Scientific Institute of Public Health; 2009 (https://www.wiv-isp.be/epidemio/spma/, accessed 23 August 2012).
BEL	Belgium	1999	1999	16.0	Standardized Procedures for Mortality Analysis (SPMA). Number and proportion of live births by delivery for all Belgium, Belgium 1990-2008 [website]. Brussels: Scientific Institute of Public Health; 2009 (https://www.wiv-isp.be/epidemio/spma/, accessed 23 August 2012).
BEL	Belgium	2000	2000	16.9	Standardized Procedures for Mortality Analysis (SPMA). Number and proportion of live births by delivery for all Belgium, Belgium 1990-2008 [website]. Brussels: Scientific Institute of Public Health; 2009 (https://www.wiv-isp.be/epidemio/spma/, accessed 23 August 2012).
BEL	Belgium	2001	2001	17.6	Standardized Procedures for Mortality Analysis (SPMA). Number and proportion of live births by delivery for all Belgium, Belgium 1990-2008 [website]. Brussels: Scientific Institute of Public Health; 2009 (https://www.wiv-isp.be/epidemio/spma/, accessed 23 August 2012).
BEL	Belgium	2002	2002	18.3	Standardized Procedures for Mortality Analysis (SPMA). Number and proportion of live births by delivery for all Belgium, Belgium 1990-2008 [website]. Brussels: Scientific Institute of Public Health; 2009 (https://www.wiv-isp.be/epidemio/spma/, accessed 23 August 2012).

Betran AP, *et al. BMJ Global Health* 2021; 6:e005671. doi: 10.1136/bmjgh-2021-005671

ISO Code	Country	Coverage start year	Coverage end year	Caesarean section rate (%)	References
BEL	Belgium	2003	2003	18.9	Standardized Procedures for Mortality Analysis (SPMA). Number and proportion of live births by delivery for all Belgium, Belgium 1990-2008 [website]. Brussels: Scientific Institute of Public Health; 2009 (https://www.wiv-isp.be/epidemio/spma/, accessed 23 August 2012).
BEL	Belgium	2004	2004	18.9	Standardized Procedures for Mortality Analysis (SPMA). Number and proportion of live births by delivery for all Belgium, Belgium 1990-2008 [website]. Brussels: Scientific Institute of Public Health; 2009 (https://www.wiv-isp.be/epidemio/spma/, accessed 23 August 2012).
BEL	Belgium	2005	2005	19.5	Standardized Procedures for Mortality Analysis (SPMA). Number and proportion of live births by delivery for all Belgium, Belgium 1990-2008 [website]. Brussels: Scientific Institute of Public Health; 2009 (https://www.wiv-isp.be/epidemio/spma/, accessed 23 August 2012).
BEL	Belgium	2006	2006	20.0	Standardized Procedures for Mortality Analysis (SPMA). Number and proportion of live births by delivery for all Belgium, Belgium 1990-2008 [website]. Brussels: Scientific Institute of Public Health; 2009 (https://www.wiv-isp.be/epidemio/spma/, accessed 23 August 2012).
BEL	Belgium	2007	2007	19.9	Standardized Procedures for Mortality Analysis (SPMA). Number and proportion of live births by delivery for all Belgium, Belgium 1990-2008 [website]. Brussels: Scientific Institute of Public Health; 2009 (https://www.wiv-isp.be/epidemio/spma/, accessed 23 August 2012).
BEL	Belgium	2008	2008	20.3	Standardized Procedures for Mortality Analysis (SPMA). Number and proportion of live births by delivery for all Belgium, Belgium 1990-2008 [website]. Brussels: Scientific Institute of Public Health; 2009 (https://www.wiv-isp.be/epidemio/spma/, accessed 23 August 2012).
BEL	Belgium	2009	2009	19.8	Standardized Procedures for Mortality Analysis (SPMA). Number and proportion of live births by delivery for all Belgium, Belgium 2008-2009 [website]. Brussels: Scientific Institute of Public Health; 2010 (https://www.wiv-isp.be/epidemio/spma/, accessed 10 October 2013).
BEL	Belgium	2010	2010	19.6	Standardized Procedures for Mortality Analysis (SPMA). Number of births by delivery for all Belgium, Belgium 2010 [website]. Brussels: Scientific Institute of Public Health; 2011 (https://www.wiv-isp.be/epidemio/spma/, accessed 17 December 2014).

Betran AP, *et al. BMJ Global Health* 2021; 6:e005671. doi: 10.1136/bmjgh-2021-005671

ISO Code	Country	Coverage start year	Coverage end year	Caesarean section rate (%)	References
BEL	Belgium	2011	2011	20.1	Standardized Procedures for Mortality Analysis (SPMA). Number of births by delivery for all Belgium, Belgium 2011 [website]. Brussels: Scientific Institute of Public Health; 2012 (https://www.wiv-isp.be/epidemio/spma/, accessed 30 October 2017).
BEL	Belgium	2011	2011	20.1	European Health for All Database (HFA-DB) [online database]. World Health Organization (WHO) Regional Office for Europe; 2016 (http://data.euro.who.int/hfadb, accessed 8 August 2016).
BEL	Belgium	2012	2012	20.6	Standardized Procedures for Mortality Analysis (SPMA). Number of births by delivery for all Belgium, Belgium 2012 [website]. Brussels: Scientific Institute of Public Health; 2013 (https://www.wiv-isp.be/epidemio/spma/, accessed 30 October 2017).
BEL	Belgium	2013	2013	21.2	Standardized Procedures for Mortality Analysis (SPMA). Number of births by delivery for all Belgium, Belgium 2013 [website]. Brussels: Scientific Institute of Public Health; 2014 (https://www.wiv-isp.be/epidemio/spma/, accessed 30 October 2017).
BEL	Belgium	2015	2015	21.3	European Perinatal Health Report. Core indicators of the health and care of pregnant women and babies in Europe in 2015. Euro-Peristat Project; 2018.
BLZ	Belize	1994	1999	9.1	1999 Belize Family Health Survey Females. Belize: Central Statistical Office; 2001.
BLZ	Belize	2009	2011	28.1	Belize Multiple Indicator Cluster Survey 2011. Final Report. Belize: The Statistical Institute of Belize (SIB), United Nations Children’s Fund (UNICEF); 2012.
BLZ	Belize	2013	2016	34.2	Statistical Institute of Belize, UNICEF Belize. Belize Multiple Indicator Cluster Survey, 2015-2016, Final Report. Belmopan, Belize: Statistical Institute of Belize, UNICEF Belize; 2017.

ISO Code	Country	Coverage start year	Coverage end year	Caesarean section rate (%)	References
BEN	Benin	1991	1996	2.2	Kodjogbé, N, Mboup G, Tossou J, de Souza L, Gandaho T, Guédémé A et al. (Institut National de la Statistique et de l'Analyse Économique, Macro International Inc.). Enquête Démographique et de Santé, République de Bénin 1996. Calverton, Maryland USA: Institut National de la Statistique et de l'Analyse Économique et Macro International Inc.; 1997 (in French).
BEN	Benin	1996	2001	3.3	Institut National de la Statistique et de l'Analyse Économique (INSAE), ORC Macro. Enquête Démographique et de Santé au Bénin 2001. Calverton, Maryland, USA: Institut National de la Statistique et de l'Analyse Économique, ORC Macro; 2002 (in French).
BEN	Benin	2001	2006	3.6	Institut National de la Statistique et de l'Analyse Économique (INSAE) [Bénin], Macro International Inc. Enquête Démographique et de Santé (EDSB-III) - Bénin 2006. Calverton, Maryland, USA: Institut National de la Statistique et de l'Analyse Économique, Macro International Inc.; 2007 (in French).
BEN	Benin	2005	2012	5.4	Institut National de la Statistique et de l'Analyse Économique (INSAE), ICF International. Enquête Démographique et de Santé du Bénin 2011-2012. Calverton, Maryland, USA: INSAE, ICF International; 2013 (in French).
BEN	Benin	2012	2014	5.3	Institut national de la statistique et de l'analyse économique (INSAE), Bénin, Fonds des Nations Unies pour l'Enfance (UNICEF). Enquête par grappes à indicateurs multiples 2014, Rapport final. Cotonou, Bénin: Institut national de la statistique et de l'analyse économique; 2015 (in French).
BEN	Benin	2012	2018	5.1	Enquête Démographique et de Santé au Bénin, 2017-2018. Cotonou, Rockville, Maryland: Institut National de la Statistique et de l'Analyse Économique (INSAE), ICF; 2018 (in French).
BTN	Bhutan	2008	2010	12.4	National Statistics Bureau, Bhutan, United Nations Children's Fund (UNICEF), United Nations Population Fund (UNFPA). Bhutan Multiple Indicator Survey 2010. Thrimphu, Bhutan: National Statistics Bureau; 2011.
BOL	Bolivia (Plurinational State of)	1995	1998	14.7	Gutiérrez Sardán M, Ochoa LH, Gómez Vargas A (Instituto Nacional de Estadística - INE [Bolivia], Macro International). Bolivia Encuesta Nacional de Demografía y Salud 1998. Calverton, Maryland: Instituto Nacional de Estadística - INE [Bolivia], Macro International; 1998 (in Spanish).

ISO Code	Country	Coverage start year	Coverage end year	Caesarean section rate (%)	References
BOL	Bolivia (Plurinational State of)	1998	2003	14.6	Gutiérrez Sardán M, Ochoa LH, Castillo Guerra W, ORC Macro. Bolivia Encuesta Nacional de Demografía y Salud 2003. Calverton, Maryland: Instituto Nacional de Estadística [Bolivia], ORC Macro; 2004 (in Spanish).
BOL	Bolivia (Plurinational State of)	2003	2008	18.6	Ramiro C, Ochoa LH, Macro International. Bolivia Encuesta Nacional de Demografía y Salud - ENDSA - 2008. Calverton, Maryland: Ministerio de Salud y Deportes and Macro International; 2009 (in Spanish).
BOL	Bolivia (Plurinational State of)	2007	2012	26.7	Encuesta de Evaluación de Salud y Nutrición 2012: Informe de Resultados. La Paz: Unidad de Análisis de Políticas Sociales y Económicas (UDAPE), Ministerio de Salud, Estado Plurinacional de Bolivia, Comité Técnico – Consejo Nacional de Alimentación y Nutrición (CT-CONAN); 2014 (in Spanish).
BOL	Bolivia (Plurinational State of)	2011	2016	33.4	Encuesta de Demografía y Salud - EDSA 2016. Bolivia: Indicadores Priorizados. La Paz: Ministerio de Salud, Instituto Nacional de Estadística (INE); 2017 (in Spanish).
BIH	Bosnia and Herzegovina	2009	2009	17.5	European Health for All Database (HFA-DB) [online database]. World Health Organization (WHO) Regional Office for Europe; 2012 (http://data.euro.who.int/hfadb, accessed 20 August 2012).
BIH	Bosnia and Herzegovina	2009	2012	13.9	The Agency for Statistics of Bosnia and Herzegovina, the Federal Ministry of Health, the Ministry of Health and Social Welfare of the Republic of Srpska, the Institute for Public Health of the Federation of Bosnia and Herzegovina, UNICEF. Bosnia and Herzegovina Multiple Indicator Cluster Survey (MICS) 2011–2012, Final Report. Sarajevo: UNICEF; 2013.
BIH	Bosnia and Herzegovina	2013	2013	24.1	European Health for All Database (HFA-DB) [online database]. World Health Organization (WHO) Regional Office for Europe; 2016 (http://data.euro.who.int/hfadb, accessed 8 August 2016).
BIH	Bosnia and Herzegovina	2014	2014	24.0	European Health for All Database (HFA-DB) [online database]. World Health Organization (WHO) Regional Office for Europe; 2016 (http://data.euro.who.int/hfadb, accessed 8 August 2016).

ISO Code	Country	Coverage start year	Coverage end year	Caesarean section rate (%)	References
BIH	Bosnia and Herzegovina	2015	2015	24.9	European Health Information Gateway. European Health for All database (HFA-DB). Caesarean sections per 1000 live births [online database]. World Health Organization (WHO) Regional Office for Europe; 2018 (https://gateway.euro.who.int/en/indicators/hfa_596-7060-caesarean-sections-per-1000-live-births/, accessed 14 February 2019).
BIH	Bosnia and Herzegovina	2017	2017	26.7	European Health Information Gateway. European Health for All database (HFA-DB). Caesarean sections per 1000 live births [online database]. World Health Organization (WHO) Regional Office for Europe; 2019 (https://gateway.euro.who.int/en/indicators/hfa_596-7060-caesarean-sections-per-1000-live-births/, accessed 5 March 2020).
BRA	Brazil	1995	1995	39.6	DATASUS [online database]. Brazil: MS/SVS/DASIS - Sistema de Informações sobre Nacidos Vivos – SINASC. Nascim p/ocorrênc por Região segundo Tipo de parto. Período: 1995; 2012 (http://tabnet.datasus.gov.br/cgi/tabcgi.exe?sinasc/cnv/nvuf.def, accessed 24 August 2012, in Portuguese).
BRA	Brazil	1996	1996	40.2	DATASUS [online database]. Brazil: MS/SVS/DASIS - Sistema de Informações sobre Nacidos Vivos – SINASC. Nascim p/ocorrênc por Região segundo Tipo de parto. Período: 1996; 2012 (http://tabnet.datasus.gov.br/cgi/tabcgi.exe?sinasc/cnv/nvuf.def, accessed 24 August 2012, in Portuguese).
BRA	Brazil	1991	1996	36.4	Sociedade Civil Bem-Estar Familiar no Brazil, BEMFAM, Programa de Pesquisas de Demografia e Saúde (DHS) Macro International Inc. Brazil Pesquisa Nacional Sobre Demografia e Saúde 1996. Calverton, Maryland, USA: Macro International Inc.; 1997 (in Portuguese).
BRA	Brazil	1997	1997	39.8	DATASUS [online database]. Brazil: MS/SVS/DASIS - Sistema de Informações sobre Nacidos Vivos – SINASC. Nascim p/ocorrênc por Região segundo Tipo de parto. Período: 1997; 2012 (http://tabnet.datasus.gov.br/cgi/tabcgi.exe?sinasc/cnv/nvuf.def, accessed 24 August 2012, in Portuguese).
BRA	Brazil	1998	1998	38.1	DATASUS [online database]. Brazil: MS/SVS/DASIS - Sistema de Informações sobre Nacidos Vivos – SINASC. Nascim p/ocorrênc por Região segundo Tipo de parto. Período: 1998; 2012 (http://tabnet.datasus.gov.br/cgi/tabcgi.exe?sinasc/cnv/nvuf.def, accessed 24 August 2012, in Portuguese).
BRA	Brazil	1999	1999	36.9	DATASUS [online database]. Brazil: MS/SVS/DASIS - Sistema de Informações sobre Nacidos Vivos – SINASC. Nascim p/ocorrênc por Região segundo Tipo de parto. Período: 1999; 2012 (http://tabnet.datasus.gov.br/cgi/tabcgi.exe?sinasc/cnv/nvuf.def, accessed 24 August 2012, in Portuguese).

ISO Code	Country	Coverage start year	Coverage end year	Caesarean section rate (%)	References
BRA	Brazil	2000	2000	37.8	DATASUS [online database]. Brazil: MS/SVS/DASIS - Sistema de Informações sobre Nacidos Vivos – SINASC. Nascim p/ocorrênc por Região segundo Tipo de parto. Período: 2000; 2012 (http://tabnet.datasus.gov.br/cgi/tabcgi.exe?sinasc/cnv/nvuf.def, accessed 24 August 2012, in Portuguese).
BRA	Brazil	2001	2001	38.1	DATASUS [online database]. Brazil: MS/SVS/DASIS - Sistema de Informações sobre Nacidos Vivos – SINASC. Nascim p/ocorrênc por Região segundo Tipo de parto. Período: 2001; 2012 (http://tabnet.datasus.gov.br/cgi/tabcgi.exe?sinasc/cnv/nvuf.def, accessed 24 August 2012, in Portuguese).
BRA	Brazil	2002	2002	38.6	DATASUS [online database]. Brazil: MS/SVS/DASIS - Sistema de Informações sobre Nacidos Vivos – SINASC. Nascim p/ocorrênc por Região segundo Tipo de parto. Período: 2002; 2012 (http://tabnet.datasus.gov.br/cgi/tabcgi.exe?sinasc/cnv/nvuf.def, accessed 24 August 2012, in Portuguese).
BRA	Brazil	2003	2003	40.0	DATASUS [online database]. Brazil: MS/SVS/DASIS - Sistema de Informações sobre Nacidos Vivos – SINASC. Nascim p/ocorrênc por Região segundo Tipo de parto. Período: 2003; 2012 (http://tabnet.datasus.gov.br/cgi/tabcgi.exe?sinasc/cnv/nvuf.def, accessed 24 August 2012, in Portuguese).
BRA	Brazil	2004	2004	41.8	DATASUS [online database]. Brazil: MS/SVS/DASIS - Sistema de Informações sobre Nacidos Vivos – SINASC. Nascim p/ocorrênc por Região segundo Tipo de parto. Período: 2004; 2012 (http://tabnet.datasus.gov.br/cgi/tabcgi.exe?sinasc/cnv/nvuf.def, accessed 24 August 2012, in Portuguese).
BRA	Brazil	2005	2005	43.2	DATASUS [online database]. Brazil: MS/SVS/DASIS - Sistema de Informações sobre Nacidos Vivos – SINASC. Nascim p/ocorrênc por Região segundo Tipo de parto. Período: 2005; 2012 (http://tabnet.datasus.gov.br/cgi/tabcgi.exe?sinasc/cnv/nvuf.def, accessed 24 August 2012, in Portuguese).
BRA	Brazil	2006	2006	45.0	DATASUS [online database]. Brazil: MS/SVS/DASIS - Sistema de Informações sobre Nacidos Vivos – SINASC. Nascim p/ocorrênc por Região segundo Tipo de parto. Período: 2006; 2012 (http://tabnet.datasus.gov.br/cgi/tabcgi.exe?sinasc/cnv/nvuf.def, accessed 24 August 2012, in Portuguese).
BRA	Brazil	2007	2007	46.5	DATASUS [online database]. Brazil: MS/SVS/DASIS - Sistema de Informações sobre Nacidos Vivos – SINASC. Nascim p/ocorrênc por Região segundo Tipo de parto. Período: 2007; 2012 (http://tabnet.datasus.gov.br/cgi/tabcgi.exe?sinasc/cnv/nvuf.def, accessed 24 August 2012, in Portuguese).

ISO Code	Country	Coverage start year	Coverage end year	Caesarean section rate (%)	References
BRA	Brazil	2008	2008	48.4	DATASUS [online database]. Brazil: MS/SVS/DASIS - Sistema de Informações sobre Nacidos Vivos – SINASC. Nascim p/ocorrênc por Região segundo Tipo de parto. Período: 2008; 2012 (http://tabnet.datasus.gov.br/cgi/tabcgi.exe?sinasc/cnv/nvuf.def, accessed 24 August 2012, in Portuguese).
BRA	Brazil	2009	2009	50.0	DATASUS [online database]. Brazil: MS/SVS/DASIS - Sistema de Informações sobre Nacidos Vivos – SINASC. Nascim p/ocorrênc por Região segundo Tipo de parto. Período: 2009; 2012 (http://tabnet.datasus.gov.br/cgi/tabcgi.exe?sinasc/cnv/nvuf.def, accessed 24 August 2012, in Portuguese).
BRA	Brazil	2010	2010	52.3	DATASUS [online database]. Brazil: MS/SVS/DASIS - Sistema de Informações sobre Nacidos Vivos – SINASC. Nascim p/ocorrênc por Região segundo Tipo de parto. Período: 2010; 2012 (http://tabnet.datasus.gov.br/cgi/tabcgi.exe?sinasc/cnv/nvuf.def, accessed 24 August 2012, in Portuguese).
BRA	Brazil	2011	2011	53.7	DATASUS [online database]. Brazil: MS/SVS/DASIS - Sistema de Informações sobre Nacidos Vivos – SINASC. Nascim p/resid.mãe por Tipo de parto segundo Região. Período: 2011; 2013 (http://tabnet.datasus.gov.br/cgi/tabcgi.exe?sinasc/cnv/nvuf.def, accessed 11 October 2013, in Portuguese).
BRA	Brazil	2012	2012	55.6	DATASUS [online database]. Brazil: MS/SVS/DASIS - Sistema de Informações sobre Nacidos Vivos – SINASC. Nascim p/resid.mãe por Tipo de parto segundo Região. Período: 2012; 2014 (http://tabnet.datasus.gov.br/cgi/tabcgi.exe?sinasc/cnv/nvuf.def, accessed 18 December 2014, in Portuguese).
BRA	Brazil	2013	2013	56.6	DATASUS [online database]. Brazil: MS/SVS/DASIS - Sistema de Informações sobre Nacidos Vivos – SINASC. Nascim p/ocorrênc por Tipo de parto Segundo Região. Período: 2013; 2016 (http://tabnet.datasus.gov.br/cgi/deftohtm.exe? sinasc/cnv/nvuf.def, accessed 8 July 2016, in Portuguese).
BRA	Brazil	2014	2014	57.0	DATASUS [online database]. Brazil: MS/SVS/DASIS - Sistema de Informações sobre Nacidos Vivos – SINASC. Nascim p/ocorrênc por Tipo de parto Segundo Região. Período: 2014; 2016 (http://tabnet.datasus.gov.br/cgi/deftohtm.exe? sinasc/cnv/nvuf.def, accessed 8 July 2016, in Portuguese).
BRA	Brazil	2015	2015	55.5	DATASUS [online database]. Brazil: MS/SVS/DASIS - Sistema de Informações sobre Nacidos Vivos – SINASC. Nascim p/ocorrênc por Tipo de parto Segundo Região. Período: 2015; 2017 (http://tabnet.datasus.gov.br/cgi/deftohtm.exe? sinasc/cnv/nvuf.def, accessed 2017, in Portuguese).

ISO Code	Country	Coverage start year	Coverage end year	Caesarean section rate (%)	References
BRA	Brazil	2016	2016	55.4	DATASUS [online database]. Brazil: MS/SVS/DASIS - Sistema de Informações sobre Nacidos Vivos – SINASC. Nascim p/ocorrênc por Tipo de parto Segundo Região. Período: 2016; 2019 (http://tabnet.datasus.gov.br/cgi/tabcgi.exe?sinasc/cnv/nvuf.def, accessed 12 February 2019, in Portuguese).
BRA	Brazil	2017	2017	55.7	DATASUS [online database]. Brazil: MS/SVS/DASIS - Sistema de Informações sobre Nacidos Vivos – SINASC. Nascim p/ocorrênc por Tipo de parto Segundo Região. Período: 2017; 2020 (http://tabnet.datasus.gov.br/cgi/tabcgi.exe?sinasc/cnv/nvuf.def, accessed 20 February 2020, in Portuguese).
BGR	Bulgaria	1990	1990	7.3	European Health for All Database (HFA-DB) [online database]. World Health Organization (WHO) Regional Office for Europe; 2012 (http://data.euro.who.int/hfadb, accessed 20 August 2012).
BGR	Bulgaria	1991	1991	7.1	European Health for All Database (HFA-DB) [online database]. World Health Organization (WHO) Regional Office for Europe; 2012 (http://data.euro.who.int/hfadb, accessed 20 August 2012).
BGR	Bulgaria	1992	1992	7.8	European Health for All Database (HFA-DB) [online database]. World Health Organization (WHO) Regional Office for Europe; 2012 (http://data.euro.who.int/hfadb, accessed 20 August 2012).
BGR	Bulgaria	1993	1993	8.5	European Health for All Database (HFA-DB) [online database]. World Health Organization (WHO) Regional Office for Europe; 2012 (http://data.euro.who.int/hfadb, accessed 20 August 2012).
BGR	Bulgaria	1994	1994	9.2	European Health for All Database (HFA-DB) [online database]. World Health Organization (WHO) Regional Office for Europe; 2012 (http://data.euro.who.int/hfadb, accessed 20 August 2012).
BGR	Bulgaria	1995	1995	10.0	European Health for All Database (HFA-DB) [online database]. World Health Organization (WHO) Regional Office for Europe; 2012 (http://data.euro.who.int/hfadb, accessed 20 August 2012).

ISO Code	Country	Coverage start year	Coverage end year	Caesarean section rate (%)	References
BGR	Bulgaria	1996	1996	10.3	European Health for All Database (HFA-DB) [online database]. World Health Organization (WHO) Regional Office for Europe; 2012 (http://data.euro.who.int/hfadb, accessed 20 August 2012).
BGR	Bulgaria	1997	1997	11.0	European Health for All Database (HFA-DB) [online database]. World Health Organization (WHO) Regional Office for Europe; 2012 (http://data.euro.who.int/hfadb, accessed 20 August 2012).
BGR	Bulgaria	1998	1998	12.4	European Health for All Database (HFA-DB) [online database]. World Health Organization (WHO) Regional Office for Europe; 2012 (http://data.euro.who.int/hfadb, accessed 20 August 2012).
BGR	Bulgaria	1999	1999	13.1	European Health for All Database (HFA-DB) [online database]. World Health Organization (WHO) Regional Office for Europe; 2012 (http://data.euro.who.int/hfadb, accessed 20 August 2012).
BGR	Bulgaria	2000	2000	13.5	European Health for All Database (HFA-DB) [online database]. World Health Organization (WHO) Regional Office for Europe; 2012 (http://data.euro.who.int/hfadb, accessed 20 August 2012).
BGR	Bulgaria	2001	2001	14.7	European Health for All Database (HFA-DB) [online database]. World Health Organization (WHO) Regional Office for Europe; 2012 (http://data.euro.who.int/hfadb, accessed 20 August 2012).
BGR	Bulgaria	2002	2002	16.8	European Health for All Database (HFA-DB) [online database]. World Health Organization (WHO) Regional Office for Europe; 2012 (http://data.euro.who.int/hfadb, accessed 20 August 2012).
BGR	Bulgaria	2003	2003	17.5	European Health for All Database (HFA-DB) [online database]. World Health Organization (WHO) Regional Office for Europe; 2012 (http://data.euro.who.int/hfadb, accessed 20 August 2012).

ISO Code	Country	Coverage start year	Coverage end year	Caesarean section rate (%)	References
BGR	Bulgaria	2004	2004	19.2	European Health for All Database (HFA-DB) [online database]. World Health Organization (WHO) Regional Office for Europe; 2012 (http://data.euro.who.int/hfadb, accessed 20 August 2012).
BGR	Bulgaria	2005	2005	22.0	European Health for All Database (HFA-DB) [online database]. World Health Organization (WHO) Regional Office for Europe; 2012 (http://data.euro.who.int/hfadb, accessed 20 August 2012).
BGR	Bulgaria	2006	2006	23.6	European Health for All Database (HFA-DB) [online database]. World Health Organization (WHO) Regional Office for Europe; 2012 (http://data.euro.who.int/hfadb, accessed 20 August 2012).
BGR	Bulgaria	2007	2007	26.8	European Health for All Database (HFA-DB) [online database]. World Health Organization (WHO) Regional Office for Europe; 2012 (http://data.euro.who.int/hfadb, accessed 20 August 2012).
BGR	Bulgaria	2008	2008	28.4	European Health for All Database (HFA-DB) [online database]. World Health Organization (WHO) Regional Office for Europe; 2012 (http://data.euro.who.int/hfadb, accessed 20 August 2012).
BGR	Bulgaria	2009	2009	32.8	European Health for All Database (HFA-DB) [online database]. World Health Organization (WHO) Regional Office for Europe; 2012 (http://data.euro.who.int/hfadb, accessed 20 August 2012).
BGR	Bulgaria	2010	2010	31.0	European Health for All Database (HFA-DB) [online database]. World Health Organization (WHO) Regional Office for Europe; 2016 (http://data.euro.who.int/hfadb, accessed 8 August 2016).
BGR	Bulgaria	2011	2011	33.1	European Health for All Database (HFA-DB) [online database]. World Health Organization (WHO) Regional Office for Europe; 2016 (http://data.euro.who.int/hfadb, accessed 8 August 2016).

ISO Code	Country	Coverage start year	Coverage end year	Caesarean section rate (%)	References
BGR	Bulgaria	2012	2012	33.4	European Health for All Database (HFA-DB) [online database]. World Health Organization (WHO) Regional Office for Europe; 2016 (http://data.euro.who.int/hfadb, accessed 8 August 2016).
BGR	Bulgaria	2013	2013	36.0	European Health for All Database (HFA-DB) [online database]. World Health Organization (WHO) Regional Office for Europe; 2016 (http://data.euro.who.int/hfadb, accessed 8 August 2016).
BGR	Bulgaria	2014	2014	39.1	European Health for All Database (HFA-DB) [online database]. World Health Organization (WHO) Regional Office for Europe; 2016 (http://data.euro.who.int/hfadb, accessed 8 August 2016).
BGR	Bulgaria	2015	2015	43.0	European Perinatal Health Report. Core indicators of the health and care of pregnant women and babies in Europe in 2015. Euro-Peristat Project; 2018.
BFA	Burkina Faso	1988	1993	1.3	Institut National de la Statistique et de la Démographie, Macro International Inc. Enquête Démographique et de Santé Burkina Faso 1993. Calverton, Maryland USA: Macro International Inc.; 1994 (in French).
BFA	Burkina Faso	1993	1999	1.1	Institut National de la Statistique et de la Démographie, Macro International Inc. Enquête Démographique et de Santé, Burkina Faso 1998-1999. Calverton, Maryland, USA: Macro International Inc.; 2000 (in French).
BFA	Burkina Faso	1998	2003	0.7	Institut National de la Statistique et de la Démographie (INSD), ORC Macro. Enquête Démographique et de Santé du Burkina Faso 2003. Calverton, Maryland, USA: INSD; ORC Macro; 2004 (in French).
BFA	Burkina Faso	2005	2010	1.9	Institut National de la Statistique et de la Démographie (INSD), ICF International. Enquête Démographique et de Santé et à Indicateurs Multiples du Burkina Faso 2010. Calverton, Maryland, USA: INSD, ICF International; 2012 (in French).

ISO Code	Country	Coverage start year	Coverage end year	Caesarean section rate (%)	References
BFA	Burkina Faso	2010	2015	3.7	Rapport du Module Démographie et Santé (MDS) de L'Enquête Multisectorielle Continue (EMC). Version en relecture. Burkina Faso: Institut National de la Statistique et de la Démographie (INSD); 2016 (in French).
BDI	Burundi	2005	2010	4.0	Institut de Statistiques et d'Études Économiques du Burundi (ISTEEBU), Ministère de la Santé Publique et de la Lutte contre le Sida [Burundi] (MSPLS), ICF International. Enquête Démographique et de Santé Burundi 2010. Bujumbura, Burundi: ISTEEBU, MSPLS, ICF International; 2012 (in French).
BDI	Burundi	2011	2017	5.0	Ministère à la Présidence chargé de la Bonne Gouvernance et du Plan [Burundi] (MPBGP), Ministère de la Santé Publique et de la Lutte contre le Sida [Burundi] (MSPLS), Institut de Statistiques et d'Études Économiques du Burundi (ISTEEBU), ICF. Troisième Enquête Démographique et de Santé. Bujumbura, Burundi : ISTEEBU, MSPLS, ICF; 2017 (in French).
CPV	Cabo Verde	1993	1998	6.0	Inquérito Demográfico e de Saúde Reprodutiva IDSR 1998. Cabo Verde Relatório Final. Praia: Instituto Nacional de Estatística (INE) [Cabo Verde], CDC; 2000 (in Portuguese).
CPV	Cabo Verde	2000	2005	10.7	Instituto Nacional de Estatística (INE) [Cabo Verde], Ministério da Saúde, Macro International. Segundo Inquérito Demográfico e de Saúde Reprodutiva, Cabo Verde, IDSR-II, 2005. Calverton, Maryland, USA: INE; 2008 (in Portuguese).
KHM	Cambodia	1995	2000	0.8	National Institute of Statistics, Directorate General for Health [Cambodia], ORC Macro. Cambodia Demographic and Health Survey 2000. Phnom Penh, Cambodia, and Calverton, Maryland USA: National Institute of Statistics, Directorate General for Health, ORC Macro; 2001.
KHM	Cambodia	2000	2005	1.8	National Institute of Public Health, National Institute of Statistics [Cambodia], ORC Macro. Cambodia Demographic and Health Survey 2005. Phnom Penh, Cambodia and Calverton, Maryland, USA: National Institute of Public Health, National Institute of Statistics, ORC Macro; 2006.
KHM	Cambodia	2005	2010	3.0	National Institute of Statistics, Directorate General for Health, ICF Macro. Cambodia Demographic and Health Survey 2010. Phnom Penh, Cambodia and Calverton, Maryland, USA: National Institute of Statistics, Directorate General for Health, ICF Macro; 2011.

ISO Code	Country	Coverage start year	Coverage end year	Caesarean section rate (%)	References
KHM	Cambodia	2009	2014	6.3	National Institute of Statistics, Directorate General for Health, ICF International. Cambodia Demographic and Health Survey 2014. Phnom Penh, Cambodia, and Rockville, Maryland, USA: National Institute of Statistics, Directorate General for Health, ICF International; 2015.
CMR	Cameroon	1993	1998	2.5	Fotso M, Ndonou R, Libité PR, Tsafack M, Wakou R, Ghapoutsa A et al. (Bureau Central des Recensements et des Études de Population et Macro International Inc.). Enquête Démographique et de Santé, Cameroun 1998. Calverton, Maryland, USA: Bureau Central des Recensements et des Études de Population, Macro International Inc.; 1999 (in French).
CMR	Cameroon	1999	2004	2.0	Institut National de la Statistique (INS) et ORC Macro. Enquête Démographique et de Santé du Cameroun 2004. Calverton, Maryland, USA: INS et ORC Macro; 2005 (in French).
CMR	Cameroon	2006	2011	3.8	Institut National de la Statistique (INS) et ICF. International. Enquête Démographique et de Santé et à Indicateurs Multiples du Cameroun 2011. Calverton, Maryland, USA: INS et ICF International; 2012 (in French.
CMR	Cameroon	2012	2014	2.4	Institut National de la Statistique, UNICEF. Enquête par grappes à indicateurs multiples (MICS5), 2014, Rapport Final. Yaoundé, Cameroun, Institut National de la Statistique; 2015 (in French).
CAN	Canada	1994	1994	17.8	Canadian Perinatal Health Report 2000. Ottawa: Minister of Public Works and Government Services Canada; 2000.
CAN	Canada	1995	1995	17.6	Canadian Perinatal Health Report 2008 Edition. Ottawa: Public Health Agency of Canada; 2008.
CAN	Canada	1996	1996	18.2	Canadian Perinatal Health Report 2008 Edition. Ottawa: Public Health Agency of Canada; 2008.

ISO Code	Country	Coverage start year	Coverage end year	Caesarean section rate (%)	References
CAN	Canada	1997	1997	18.5	Canadian Perinatal Health Report 2008 Edition. Ottawa: Public Health Agency of Canada; 2008.
CAN	Canada	1998	1998	19.0	Canadian Perinatal Health Report 2008 Edition. Ottawa: Public Health Agency of Canada; 2008.
CAN	Canada	1999	1999	19.7	Canadian Perinatal Health Report 2008 Edition. Ottawa: Public Health Agency of Canada; 2008.
CAN	Canada	2000	2000	21.9	Perinatal Health Indicators for Canada 2011. Ottawa: Public Health Agency of Canada; 2012.
CAN	Canada	2001	2001	23.4	Perinatal Health Indicators for Canada 2011. Ottawa: Public Health Agency of Canada; 2012.
CAN	Canada	2002	2002	24.5	Perinatal Health Indicators for Canada 2011. Ottawa: Public Health Agency of Canada; 2012.
CAN	Canada	2003	2003	25.8	Perinatal Health Indicators for Canada 2011. Ottawa: Public Health Agency of Canada; 2012.
CAN	Canada	2004	2004	26.5	Perinatal Health Indicators for Canada 2011. Ottawa: Public Health Agency of Canada; 2012.

Betran AP, *et al. BMJ Global Health* 2021; 6:e005671. doi: 10.1136/bmjgh-2021-005671

ISO Code	Country	Coverage start year	Coverage end year	Caesarean section rate (%)	References
CAN	Canada	2005	2005	27.3	Perinatal Health Indicators for Canada 2011. Ottawa: Public Health Agency of Canada; 2012.
CAN	Canada	2006	2006	27.3	Perinatal Health Indicators for Canada 2011. Ottawa: Public Health Agency of Canada; 2012.
CAN	Canada	2007	2007	27.7	Perinatal Health Indicators for Canada 2011. Ottawa: Public Health Agency of Canada; 2012.
CAN	Canada	2008	2008	28.0	Perinatal Health Indicators for Canada 2011. Ottawa: Public Health Agency of Canada; 2012.
CAN	Canada	2009	2009	27.8	Perinatal Health Indicators for Canada 2011. Ottawa: Public Health Agency of Canada; 2012.
CAN	Canada	2010	2011	26.9	Health Indicators 2012. Ottawa: Canadian Institute for Health Information (CIHI); 2012.
CAN	Canada	2011	2012	27.1	Health Indicators 2013. Ottawa: Canadian Institute for Health Information (CIHI); 2013.
CAN	Canada	2014	2014	27.5	Health Indicators Interactive Tool. Health System Performance. 2014. Caesarean Section-2014-Percent [database]. Ottawa: Canadian Institute for Health Information (CIHI); 2016 (http://yourhealthsystem.cihi.ca/epub/SearchServlet, accessed on 30 June 2016).

ISO Code	Country	Coverage start year	Coverage end year	Caesarean section rate (%)	References
CAN	Canada	2015	2015	27.9	Health Indicators Interactive Tool. Health System Performance. 2015. Caesarean Section-2015-Percent [database]. Ottawa: Canadian Institute for Health Information (CIHI); 2017 (http://yourhealthsystem.cihi.ca/epub/SearchServlet, accessed on 22 December 2017).
CAN	Canada	2016	2016	28.2	Health Indicators Interactive Tool. Health System Performance. 2016. Caesarean Section-2016-Percent [database]. Ottawa: Canadian Institute for Health Information (CIHI); 2019 (http://yourhealthsystem.cihi.ca/epub/SearchServlet, accessed on 12 February 2019).
CAN	Canada	2017	2017	28.8	Health Indicators Interactive Tool. Health System Performance. 2016. Caesarean Section-2017-Percent [database]. Ottawa: Canadian Institute for Health Information (CIHI); 2020 (http://yourhealthsystem.cihi.ca/epub/SearchServlet, accessed on 20 February 2020).
CAF	Central African Republic	1991	1995	1.9	Ndamobissi R, Mboup G, Nguélébé EO. Enquête Démographique et de Santé, République Centrafricaine 1994-95. Calverton, Maryland, USA: Direction des Statistiques Démographiques et Sociales et Macro International Inc.; 1995 (in French).
CAF	Central African Republic	2008	2010	4.5	ICASEES, Fonds des Nations Unies pour l'Enfance (UNICEF). Enquête par grappes à indicateurs multiples MICS, RCA 2010 Rapport final. Bangui: RCA ICASEES; 2012 (in French).
TCD	Chad	1991	1997	0.5	Ouagadjio B, Nodjimadji K, Ngoniri JN, Ngakoutou N, Ignégongba K, Tokindang JS et al. (Bureau Central du Recensement, Macro International Inc.) Enquête Démographique et de Santé, Tchad 1996-1997. Calverton, Maryland, USA: Bureau Central du Recensement, Macro International Inc.; 1998 (in French).
TCD	Chad	1999	2004	0.4	Ouagadjio B, Nodjimadji K, Bagamla T, Madnodji R, Tokindang JS, Ngakoutou N et al. (INSEED, ORC Macro). Enquête Démographique et de Santé Tchad 2004. Calverton, Maryland, USA: INSEED, ORC Macro; 2005 (in French).
TCD	Chad	2008	2010	1.5	Enquête par grappes à indicateurs multiples Tchad 2010 - Rapport Final. Tchad: Ministère du Plan, de l'Economie et de la Coopération Internationale. Institut National de la Statistique, des Études Économiques et Démographiques (INSEED), Fonds des Nations Unies pour la population (FNUAP/UNFPA), Fonds des Nations Unies pour l'Enfance (UNICEF); 2011 (in French).

ISO Code	Country	Coverage start year	Coverage end year	Caesarean section rate (%)	References
TCD	Chad	2009	2015	1.4	Institut National de la Statistique, des Études Économiques et Démographiques (INSEED), Ministère de la Santé Publique (MSP), ICF International. Enquête Démographique et de Santé et à Indicateurs Multiples (EDS-MICS 2014-2015). Rockville, Maryland, USA : INSEED, MSP, ICF International; 2016 (in French).
CHL	Chile	1990	1990	30.6	Murray SF, Serani Pradenas F. Cesarean Birth Trends in Chile, 1986 to 1994. Birth. 1997; 24:4:258-63.
CHL	Chile	1991	1991	33.1	Murray SF, Serani Pradenas F. Cesarean Birth Trends in Chile, 1986 to 1994. Birth. 1997; 24:4:258-63.
CHL	Chile	1992	1992	34.8	Murray SF, Serani Pradenas F. Cesarean Birth Trends in Chile, 1986 to 1994. Birth. 1997; 24:4:258-63.
CHL	Chile	1993	1993	36.0	Murray SF, Serani Pradenas F. Cesarean Birth Trends in Chile, 1986 to 1994. Birth. 1997; 24:4:258-63.
CHL	Chile	1994	1994	37.2	Murray SF, Serani Pradenas F. Cesarean Birth Trends in Chile, 1986 to 1994. Birth. 1997; 24:4:258-63.
CHL	Chile	2001	2001	30.7	Guzmán E. Perfil epidemiológico de la cesárea en Chile en la década 2000-2010 [Epidemiological profile of caesarean section in Chile in the decade 2000-2010]. Medwave. 2012;12(3):e5331. Doi: 10.5867/medwave.2012.03.5331 (in Spanish).
CHL	Chile	2002	2002	30.7	Guzmán E. Perfil epidemiológico de la cesárea en Chile en la década 2000-2010 [Epidemiological profile of caesarean section in Chile in the decade 2000-2010]. Medwave. 2012;12(3):e5331. Doi: 10.5867/medwave.2012.03.5331 (in Spanish).

ISO Code	Country	Coverage start year	Coverage end year	Caesarean section rate (%)	References
CHL	Chile	2003	2003	30.9	Guzmán E. Perfil epidemiológico de la cesárea en Chile en la década 2000-2010 [Epidemiological profile of caesarean section in Chile in the decade 2000-2010]. Medwave. 2012;12(3):e5331. Doi: 10.5867/medwave.2012.03.5331 (in Spanish).
CHL	Chile	2004	2004	31.6	Guzmán E. Perfil epidemiológico de la cesárea en Chile en la década 2000-2010 [Epidemiological profile of caesarean section in Chile in the decade 2000-2010]. Medwave. 2012;12(3):e5331. Doi: 10.5867/medwave.2012.03.5331 (in Spanish).
CHL	Chile	2005	2005	32.4	Guzmán E. Perfil epidemiológico de la cesárea en Chile en la década 2000-2010 [Epidemiological profile of caesarean section in Chile in the decade 2000-2010]. Medwave. 2012;12(3):e5331. Doi: 10.5867/medwave.2012.03.5331 (in Spanish).
CHL	Chile	2006	2006	33.7	Guzmán E. Perfil epidemiológico de la cesárea en Chile en la década 2000-2010 [Epidemiological profile of caesarean section in Chile in the decade 2000-2010]. Medwave. 2012;12(3):e5331. Doi: 10.5867/medwave.2012.03.5331 (in Spanish).
CHL	Chile	2007	2007	34.7	Guzmán E. Perfil epidemiológico de la cesárea en Chile en la década 2000-2010 [Epidemiological profile of caesarean section in Chile in the decade 2000-2010]. Medwave. 2012;12(3):e5331. Doi: 10.5867/medwave.2012.03.5331 (in Spanish).
CHL	Chile	2008	2008	35.7	Guzmán E. Perfil epidemiológico de la cesárea en Chile en la década 2000-2010 [Epidemiological profile of caesarean section in Chile in the decade 2000-2010]. Medwave. 2012;12(3):e5331. Doi: 10.5867/medwave.2012.03.5331 (in Spanish).
CHL	Chile	2009	2009	36.4	Guzmán E. Perfil epidemiológico de la cesárea en Chile en la década 2000-2010 [Epidemiological profile of caesarean section in Chile in the decade 2000-2010]. Medwave. 2012;12(3):e5331. Doi: 10.5867/medwave.2012.03.5331 (in Spanish).
CHL	Chile	2010	2010	37.0	Guzmán E. Perfil epidemiológico de la cesárea en Chile en la década 2000-2010 [Epidemiological profile of caesarean section in Chile in the decade 2000-2010]. Medwave. 2012;12(3):e5331. Doi: 10.5867/medwave.2012.03.5331 (in Spanish).

ISO Code	Country	Coverage start year	Coverage end year	Caesarean section rate (%)	References
CHL	Chile	2012	2012	49.6	Segunda ronda encuesta longitudinal de la primera infancia. Informe resultados encuesta. Santiago: Centro Micro Datos, Departamento de Economía, Universidad de Chile; 2013 (in Spanish).
CHN	China	2003	2003	11.0	Xu, K. [e-mail, 3 March 2010].
CHN	China	2008	2008	28.8	Li H-T, Luo S, Trasande L, Hellerstein S, Kang C, Li J-X et al. Geographic Variations and Temporal Trends in Cesarean Delivery Rates in China, 2008-2014. JAMA. 2017; 317(1):69-76. doi: 10.1001/jama.2016.18663.
CHN	China	2009	2009	30.6	Li H-T, Luo S, Trasande L, Hellerstein S, Kang C, Li J-X et al. Geographic Variations and Temporal Trends in Cesarean Delivery Rates in China, 2008-2014. JAMA. 2017; 317(1):69-76. doi: 10.1001/jama.2016.18663.
CHN	China	2010	2010	31.9	Li H-T, Luo S, Trasande L, Hellerstein S, Kang C, Li J-X et al. Geographic Variations and Temporal Trends in Cesarean Delivery Rates in China, 2008-2014. JAMA. 2017; 317(1):69-76. doi: 10.1001/jama.2016.18663.
CHN	China	2011	2011	33.1	Li H-T, Luo S, Trasande L, Hellerstein S, Kang C, Li J-X et al. Geographic Variations and Temporal Trends in Cesarean Delivery Rates in China, 2008-2014. JAMA. 2017; 317(1):69-76. doi: 10.1001/jama.2016.18663.
CHN	China	2012	2012	34.0	Li H-T, Luo S, Trasande L, Hellerstein S, Kang C, Li J-X et al. Geographic Variations and Temporal Trends in Cesarean Delivery Rates in China, 2008-2014. JAMA. 2017; 317(1):69-76. doi: 10.1001/jama.2016.18663.
CHN	China	2013	2013	34.6	Li H-T, Luo S, Trasande L, Hellerstein S, Kang C, Li J-X et al. Geographic Variations and Temporal Trends in Cesarean Delivery Rates in China, 2008-2014. JAMA. 2017; 317(1):69-76. doi: 10.1001/jama.2016.18663.

ISO Code	Country	Coverage start year	Coverage end year	Caesarean section rate (%)	References
CHN	China	2014	2014	34.9	Li H-T, Luo S, Trasande L, Hellerstein S, Kang C, Li J-X et al. Geographic Variations and Temporal Trends in Cesarean Delivery Rates in China, 2008-2014. JAMA. 2017; 317(1):69-76. doi: 10.1001/jama.2016.18663.
COL	Colombia	1990	1995	16.9	Profamilia, Macro International Inc. Encuesta Nacional de Demografía y Salud 1995. Bogotá, Colombia: Profamilia, Macro International; 1995 (in Spanish).
COL	Colombia	1999	1999	26.1	DANE [online database]. Estadísticas Vitales – Nacimientos por tipo de parto, según departamento de residencia de la madre y multiplicidad del embarazo. 1999. Bogotá D.C.: Departamento Administrativo Nacional de Estadística (DANE); 2012 (http://www.dane.gov.co/index.php?option=com_content&view=article&id=786&Itemid=119, accessed 29 August 2012, in Spanish).
COL	Colombia	2000	2000	26.9	DANE [online database]. Estadísticas Vitales – Nacimientos por tipo de parto, según departamento de residencia de la madre y multiplicidad del embarazo. 2000. Bogotá D.C.: Departamento Administrativo Nacional de Estadística (DANE); 2012 (http://www.dane.gov.co/index.php?option=com_content&view=article&id=786&Itemid=119, accessed 29 August 2012, in Spanish).
COL	Colombia	1995	2000	23.6	Profamilia, Macro International Inc. Salud Sexual y Reproductiva en Colombia. Resultados Encuesta Nacional de Demografía y Salud 2000. Bogotá, Colombia: Profamilia; 2000 (in Spanish).
COL	Colombia	2001	2001	27.3	DANE [online database]. Estadísticas Vitales – Nacimientos por tipo de parto, según departamento de residencia de la madre y multiplicidad del embarazo. 2001. Bogotá D.C.: Departamento Administrativo Nacional de Estadística (DANE); 2012 (http://www.dane.gov.co/index.php?option=com_content&view=article&id=786&Itemid=119, accessed 29 August 2012, in Spanish).
COL	Colombia	2004	2004	29.4	DANE [online database]. Estadísticas Vitales – Nacimientos por tipo de parto, según departamento de residencia de la madre y multiplicidad del embarazo. 2004. Bogotá D.C.: Departamento Administrativo Nacional de Estadística (DANE); 2012 (http://www.dane.gov.co/index.php?option=com_content&view=article&id=786&Itemid=119, accessed 29 August 2012, in Spanish).
COL	Colombia	2005	2005	31.3	DANE [online database]. Estadísticas Vitales – Nacimientos por tipo de parto, según departamento de residencia de la madre y multiplicidad del embarazo. 2005. Bogotá D.C.: Departamento Administrativo Nacional de Estadística (DANE); 2012 (http://www.dane.gov.co/index.php?option=com_content&view=article&id=786&Itemid=119, accessed 29 August 2012, in Spanish).

ISO Code	Country	Coverage start year	Coverage end year	Caesarean section rate (%)	References
COL	Colombia	2000	2005	26.7	Ojeda G (Profamilia), Ordonez M (Profamilia), Ochoa LH (Macro International Inc.). Colombia Salud Sexual y Reproductiva: Resultados Encuesta Nacional de Demografía y Salud 2005. Bogotá, Colombia: Profamilia; 2005 (in Spanish).
COL	Colombia	2006	2006	33.2	DANE [online database]. Estadísticas Vitales – Nacimientos por tipo de parto, según departamento de residencia de la madre y multiplicidad del embarazo. 2006. Bogotá D.C.: Departamento Administrativo Nacional de Estadística (DANE); 2012 (http://www.dane.gov.co/index.php?option=com_content&view=article&id=786&Itemid=119, accessed 29 August 2012, in Spanish).
COL	Colombia	2009	2009	39.0	DANE [online database]. Estadísticas Vitales – Nacimientos por tipo de parto, según departamento de residencia de la madre y multiplicidad del embarazo. 2009. Bogotá D.C.: Departamento Administrativo Nacional de Estadística (DANE); 2012 (http://www.dane.gov.co/index.php?option=com_content&view=article&id=786&Itemid=119, accessed 29 August 2012, in Spanish).
COL	Colombia	2010	2010	40.1	DANE [online database]. Estadísticas Vitales – Nacimientos por tipo de parto, según departamento de residencia de la madre y multiplicidad del embarazo. 2010. Bogotá D.C.: Departamento Administrativo Nacional de Estadística (DANE); 2012 (http://www.dane.gov.co/index.php?option=com_content&view=article&id=786&Itemid=119, accessed 29 August 2012, in Spanish).
COL	Colombia	2005	2010	34.4	Ojeda G, Ordonez M, Ochoa LH (Profamilia), ICF Macro. Colombia Encuesta Nacional de Demografía y Salud 2010. Bogotá, Colombia: Profamilia; 2011 (in Spanish).
COL	Colombia	2011	2011	42.8	DANE [online database]. Estadísticas Vitales – Nacimientos por tipo de parto, según departamento de residencia de la madre y multiplicidad del embarazo. 2011. Bogotá D.C.: Departamento Administrativo Nacional de Estadística (DANE); 2012 (http://www.dane.gov.co/index.php?option=com_content&view=article&id=786&Itemid=119, accessed 29 August 2012, in Spanish).
COL	Colombia	2014	2014	46.2	DANE [online database]. Estadísticas Vitales – Nacimientos por tipo de parto, según departamento de residencia de la madre y multiplicidad del embarazo. 2014. Bogotá D.C.: Departamento Administrativo Nacional de Estadística (DANE); 2016 (http://www.dane.gov.com, accessed 11 July 2016, in Spanish).
COL	Colombia	2015	2015	46.4	DANE [online database]. Estadísticas Vitales – Nacimientos por tipo de parto, según departamento de residencia de la madre y multiplicidad del embarazo. 2015. Bogotá D.C.: Departamento Administrativo Nacional de Estadística (DANE); 2017 (http://www.dane.gov.com, accessed 31 October 2017, in Spanish).

ISO Code	Country	Coverage start year	Coverage end year	Caesarean section rate (%)	References
COL	Colombia	2016	2016	45.8	DANE [online database]. Estadísticas Vitales – Nacimientos por tipo de parto, según departamento de residencia de la madre y multiplicidad del embarazo. 2016. Bogotá D.C.: Departamento Administrativo Nacional de Estadística (DANE); 2017 (http://www.dane.gov.com, accessed 31 October 2017, in Spanish).
COL	Colombia	2010	2015	43.2	Ministerio de Salud y Protección Social, Profamilia. Colombia Encuesta Nacional de Demográfia y Salud 2015. Bogotá, Colombia: Profamilia; 2017 (in Spanish).
COL	Colombia	2017	2017	45.5	DANE [online database]. Estadísticas Vitales – Nacimientos por tipo de parto, según departamento de residencia de la madre y multiplicidad del embarazo. 2017. Bogotá D.C.: Departamento Administrativo Nacional de Estadística (DANE); 2019 (http://www.dane.gov.com, accessed 12 February 2019, in Spanish).
COL	Colombia	2018	2018	44.4	DANE [online database]. Estadísticas Vitales - Nacimientos 2018. Cifras definitivas 2018. Cuadro 10. Nacimientos por tipo de parto, según departamento de residencia de la madre y multiplicidad del embarazo. Bogotá D.C.: Departamento Administrativo Nacional de Estadística (DANE); 2019 (https://www.dane.gov.co/index.php/estadisticas-por-tema/salud/nacimientos-y-defunciones/nacimientos/nacimientos-2018, accessed 20 February 2020, in Spanish).
COM	Comoros	1993	1996	5.3	Mondoha KA, Schoemaker J, Barrère M. Enquête Démographique et de Santé, Comores 1996. Calverton, Maryland: Centre National de Documentation et de Recherche Scientifique et Macro International Inc.; 1997 (in French).
COM	Comoros	2007	2012	9.6	Direction Générale de la Statistique et de la Prospective (DGSP), ICF International. Enquête Démographique et de Santé et à Indicateurs Multiples aux Comores 2012. Rockville, MD 20850 USA : DGSP, ICF International; 2014 (in French).
COG	Congo	2000	2005	3.2	Centre National de la Statistique et des Études Économiques (CNSEE) et ORC Macro. Enquête Démographique et de Santé du Congo 2005. Calverton, Maryland, USA: CNSEE et ORC Macro; 2006 (in French). DHS 2005.
COG	Congo	2006	2012	5.8	Centre Nationale de la Statistique et des Études Économiques (CNSEE) [Congo], ICF International. Enquête Démographique et de Santé du Congo (EDSC-II) 2011-2012. Calverton, Maryland, USA: CNSEE, ICF International; 2013 (in French).

ISO Code	Country	Coverage start year	Coverage end year	Caesarean section rate (%)	References
COG	Congo	2012	2015	4.9	Institut National de la Statistique (INS), Fonds des Nations Unies pour l'Enfance (UNICEF). Enquête par grappes à indicateurs multiples, MICS5 Congo 2014-2015, Rapport Final. Brazzaville, Congo: INS, UNICEF; 2015 (in French).
CRI	Costa Rica	1990	1990	19.7	Estadísticas de Salud. Cuadro No 71. Partos, Nacimientos y porcentajes de parturientas con Atención Prenatal y con Cesárea; Nacimientos con bajo e Insuficiente peso al nacer, C. C. S. S., 1980-2012 [website]. San José: Caja Costarricense del Seguro Social; 2012 (http://www.ccss.sa.cr/estadisticas_salud_docs, accessed 29 August 2012, in Spanish).
CRI	Costa Rica	1991	1991	19.7	Estadísticas de Salud. Cuadro No 71. Partos, Nacimientos y porcentajes de parturientas con Atención Prenatal y con Cesárea; Nacimientos con bajo e Insuficiente peso al nacer, C. C. S. S., 1980-2012 [website]. San José: Caja Costarricense del Seguro Social; 2012 (http://www.ccss.sa.cr/estadisticas_salud_docs, accessed 29 August 2012, in Spanish).
CRI	Costa Rica	1992	1992	20.4	Estadísticas de Salud. Cuadro No 71. Partos, Nacimientos y porcentajes de parturientas con Atención Prenatal y con Cesárea; Nacimientos con bajo e Insuficiente peso al nacer, C. C. S. S., 1980-2012 [website]. San José: Caja Costarricense del Seguro Social; 2012 (http://www.ccss.sa.cr/estadisticas_salud_docs, accessed 29 August 2012, in Spanish).
CRI	Costa Rica	1993	1993	20.9	Estadísticas de Salud. Cuadro No 71. Partos, Nacimientos y porcentajes de parturientas con Atención Prenatal y con Cesárea; Nacimientos con bajo e Insuficiente peso al nacer, C. C. S. S., 1980-2012 [website]. San José: Caja Costarricense del Seguro Social; 2012 (http://www.ccss.sa.cr/estadisticas_salud_docs, accessed 29 August 2012, in Spanish).
CRI	Costa Rica	1994	1994	20.6	Estadísticas de Salud. Cuadro No 71. Partos, Nacimientos y porcentajes de parturientas con Atención Prenatal y con Cesárea; Nacimientos con bajo e Insuficiente peso al nacer, C. C. S. S., 1980-2012 [website]. San José: Caja Costarricense del Seguro Social; 2012 (http://www.ccss.sa.cr/estadisticas_salud_docs, accessed 29 August 2012, in Spanish).
CRI	Costa Rica	1995	1995	20.7	Estadísticas de Salud. Cuadro No 71. Partos, Nacimientos y porcentajes de parturientas con Atención Prenatal y con Cesárea; Nacimientos con bajo e Insuficiente peso al nacer, C. C. S. S., 1980-2012 [website]. San José: Caja Costarricense del Seguro Social; 2012 (http://www.ccss.sa.cr/estadisticas_salud_docs, accessed 29 August 2012, in Spanish).
CRI	Costa Rica	1996	1996	20.9	Estadísticas de Salud. Cuadro No 71. Partos, Nacimientos y porcentajes de parturientas con Atención Prenatal y con Cesárea; Nacimientos con bajo e Insuficiente peso al nacer, C. C. S. S., 1980-2012 [website]. San José: Caja Costarricense del Seguro Social; 2012 (http://www.ccss.sa.cr/estadisticas_salud_docs, accessed 29 August 2012, in Spanish).

Betran AP, *et al. BMJ Global Health* 2021; 6:e005671. doi: 10.1136/bmjgh-2021-005671

ISO Code	Country	Coverage start year	Coverage end year	Caesarean section rate (%)	References
CRI	Costa Rica	1997	1997	21.0	Estadísticas de Salud. Cuadro No 71. Partos, Nacimientos y porcentajes de parturientas con Atención Prenatal y con Cesárea; Nacimientos con bajo e Insuficiente peso al nacer, C. C. S. S., 1980-2012 [website]. San José: Caja Costarricense del Seguro Social; 2012 (http://www.ccss.sa.cr/estadisticas_salud_docs, accessed 29 August 2012, in Spanish).
CRI	Costa Rica	1998	1998	20.8	Estadísticas de Salud. Cuadro No 71. Partos, Nacimientos y porcentajes de parturientas con Atención Prenatal y con Cesárea; Nacimientos con bajo e Insuficiente peso al nacer, C. C. S. S., 1980-2012 [website]. San José: Caja Costarricense del Seguro Social; 2012 (http://www.ccss.sa.cr/estadisticas_salud_docs, accessed 29 August 2012, in Spanish).
CRI	Costa Rica	1999	1999	21.7	Estadísticas de Salud. Cuadro No 71. Partos, Nacimientos y porcentajes de parturientas con Atención Prenatal y con Cesárea; Nacimientos con bajo e Insuficiente peso al nacer, C. C. S. S., 1980-2012 [website]. San José: Caja Costarricense del Seguro Social; 2012 (http://www.ccss.sa.cr/estadisticas_salud_docs, accessed 29 August 2012, in Spanish).
CRI	Costa Rica	2000	2000	21.3	Estadísticas de Salud. Cuadro No 71. Partos, Nacimientos y porcentajes de parturientas con Atención Prenatal y con Cesárea; Nacimientos con bajo e Insuficiente peso al nacer, C. C. S. S., 1980-2012 [website]. San José: Caja Costarricense del Seguro Social; 2012 (http://www.ccss.sa.cr/estadisticas_salud_docs, accessed 29 August 2012, in Spanish).
CRI	Costa Rica	2001	2001	22.2	Estadísticas de Salud. Cuadro No 71. Partos, Nacimientos y porcentajes de parturientas con Atención Prenatal y con Cesárea; Nacimientos con bajo e Insuficiente peso al nacer, C. C. S. S., 1980-2012 [website]. San José: Caja Costarricense del Seguro Social; 2012 (http://www.ccss.sa.cr/estadisticas_salud_docs, accessed 29 August 2012, in Spanish).
CRI	Costa Rica	2002	2002	22.0	Estadísticas de Salud. Cuadro No 71. Partos, Nacimientos y porcentajes de parturientas con Atención Prenatal y con Cesárea; Nacimientos con bajo e Insuficiente peso al nacer, C. C. S. S., 1980-2012 [website]. San José: Caja Costarricense del Seguro Social; 2012 (http://www.ccss.sa.cr/estadisticas_salud_docs, accessed 29 August 2012, in Spanish).
CRI	Costa Rica	2003	2003	22.0	Estadísticas de Salud. Cuadro No 71. Partos, Nacimientos y porcentajes de parturientas con Atención Prenatal y con Cesárea; Nacimientos con bajo e Insuficiente peso al nacer, C. C. S. S., 1980-2012 [website]. San José: Caja Costarricense del Seguro Social; 2012 (http://www.ccss.sa.cr/estadisticas_salud_docs, accessed 29 August 2012, in Spanish).
CRI	Costa Rica	2004	2004	21.8	Estadísticas de Salud. Cuadro No 71. Partos, Nacimientos y porcentajes de parturientas con Atención Prenatal y con Cesárea; Nacimientos con bajo e Insuficiente peso al nacer, C. C. S. S., 1980-2012 [website]. San José: Caja Costarricense del Seguro Social; 2012 (http://www.ccss.sa.cr/estadisticas_salud_docs, accessed 29 August 2012, in Spanish).

ISO Code	Country	Coverage start year	Coverage end year	Caesarean section rate (%)	References
CRI	Costa Rica	2005	2005	21.6	Estadísticas de Salud. Cuadro No 71. Partos, Nacimientos y porcentajes de parturientas con Atención Prenatal y con Cesárea; Nacimientos con bajo e Insuficiente peso al nacer, C. C. S. S., 1980-2012 [website]. San José: Caja Costarricense del Seguro Social; 2012 (http://www.ccss.sa.cr/estadisticas_salud_docs, accessed 29 August 2012, in Spanish).
CRI	Costa Rica	2006	2006	22.1	Estadísticas de Salud. Cuadro No 71. Partos, Nacimientos y porcentajes de parturientas con Atención Prenatal y con Cesárea; Nacimientos con bajo e Insuficiente peso al nacer, C. C. S. S., 1980-2012 [website]. San José: Caja Costarricense del Seguro Social; 2012 (http://www.ccss.sa.cr/estadisticas_salud_docs, accessed 29 August 2012, in Spanish).
CRI	Costa Rica	2007	2007	21.4	Estadísticas de Salud. Cuadro No 71. Partos, Nacimientos y porcentajes de parturientas con Atención Prenatal y con Cesárea; Nacimientos con bajo e Insuficiente peso al nacer, C. C. S. S., 1980-2012 [website]. San José: Caja Costarricense del Seguro Social; 2012 (http://www.ccss.sa.cr/estadisticas_salud_docs, accessed 29 August 2012, in Spanish).
CRI	Costa Rica	2008	2008	20.5	Estadísticas de Salud. Cuadro No 71. Partos, Nacimientos y porcentajes de parturientas con Atención Prenatal y con Cesárea; Nacimientos con bajo e Insuficiente peso al nacer, C. C. S. S., 1980-2012 [website]. San José: Caja Costarricense del Seguro Social; 2012 (http://www.ccss.sa.cr/estadisticas_salud_docs, accessed 29 August 2012, in Spanish).
CRI	Costa Rica	2009	2009	20.4	Estadísticas de Salud. Cuadro No 71. Partos, Nacimientos y porcentajes de parturientas con Atención Prenatal y con Cesárea; Nacimientos con bajo e Insuficiente peso al nacer, C. C. S. S., 1980-2012 [website]. San José: Caja Costarricense del Seguro Social; 2012 (http://www.ccss.sa.cr/estadisticas_salud_docs, accessed 29 August 2012, in Spanish).
CRI	Costa Rica	2009	2011	26.2	Encuesta de Indicadores Múltiples por Conglomerados 2011: Monitoreo de la Situación de la Niñez y las Mujeres, Costa Rica. San José: Ministerio de Salud [Costa Rica], Fondo de las Naciones Unidas para la Infancia (UNICEF); 2013 (in Spanish).
CRI	Costa Rica	2011	2011	20.5	Estadísticas de Salud. Cuadro No 71. Partos, Nacimientos y porcentajes de parturientas con Atención Prenatal y con Cesárea; Nacimientos con bajo e Insuficiente peso al nacer, C. C. S. S., 1980-2012 [website]. San José: Caja Costarricense del Seguro Social; 2012 (http://www.ccss.sa.cr/estadisticas_salud_docs, accessed 29 August 2012, in Spanish).
CRI	Costa Rica	2012	2012	21.6	Estadísticas de Salud. Cuadro No 43. Partos, Porcentaje con cesárea, abortos, nacimientos, porcentaje de defunciones fetales y defunciones maternas, segun red de servicios y centro de salud. C. C. S. S., 2012 [website]. San José: Caja Costarricense del Seguro Social; 2013 (http://www.ccss.sa.cr/estadisticas_salud_docs, accessed 14 October 2013, in Spanish).

ISO Code	Country	Coverage start year	Coverage end year	Caesarean section rate (%)	References
CRI	Costa Rica	2013	2013	21.9	Estadísticas de Salud. Cuadro No 43. Partos, Porcentaje con cesárea, abortos, nacimientos, porcentaje de defunciones fetales y defunciones maternas, segun red de servicios y centro de salud. C. C. S. S., 2013 [website]. San José: Caja Costarricense del Seguro Social; 2014 (http://www.ccss.sa.cr/estadisticas_salud_docs, accessed 19 December 2014, in Spanish).
CRI	Costa Rica	2014	2014	23.5	Estadísticas de Salud. Cuadro No 43. Partos, Porcentaje con cesárea, abortos, nacimientos, porcentaje de defunciones fetales y defunciones maternas, segun red de servicios y centro de salud. C. C. S. S., 2014 [website]. San José: Caja Costarricense del Seguro Social; 2016 (http://www.ccss.sa.cr/arc/estadisticas/salud/80/an_est_8788b_2014.zip, accessed 12 July 2016, in Spanish).
CRI	Costa Rica	2015	2015	23.8	Anuario Estadístico 2017. Cuadro No 71. Partos, nacimientos y porcentajes de parturientas con atencion prenatal y con cesárea; naciminetos con bajo e insuficiente peso al nacer, C.C.S.S., 1980 - 2017 [website]. San José: Caja Costarricense del Seguro Social; 2019 (https://www.ccss.sa.cr/est_salud, accessed 19 February 2019, in Spanish).
CRI	Costa Rica	2016	2016	23.9	Anuario Estadístico 2017. Cuadro No 71. Partos, nacimientos y porcentajes de parturientas con atencion prenatal y con cesárea; naciminetos con bajo e insuficiente peso al nacer, C.C.S.S., 1980 - 2017 [website]. San José: Caja Costarricense del Seguro Social; 2019 (https://www.ccss.sa.cr/est_salud, accessed 19 February 2019, in Spanish).
CRI	Costa Rica	2017	2017	24.3	Anuario Estadístico 2017. Cuadro No 71. Partos, nacimientos y porcentajes de parturientas con atencion prenatal y con cesárea; naciminetos con bajo e insuficiente peso al nacer, C.C.S.S., 1980 - 2017 [website]. San José: Caja Costarricense del Seguro Social; 2019 (https://www.ccss.sa.cr/est_salud, accessed 19 February 2019, in Spanish).
CIV	Côte d'Ivoire	1991	1994	1.8	Sombo, N'Cho, Lucien Kouassi, Albert Kouamé Koffi, Juan Schoemaker, Monique Barrère, Bernard Barrère, Prosper Poukouta (Institut National de la Statistique [Côte d'Ivoire], Macro International). Enquête Démographique et de Santé, Côte d'Ivoire 1994. Calverton, Maryland, USA: Institut National de la Statistique [Côte d'Ivoire], Macro International; 1995 (in French).
CIV	Côte d'Ivoire	1993	1999	2.5	Institut National de la Statistique [Côte d'Ivoire] et ORC Macro. Enquête Démographique et de Santé, Côte d'Ivoire 1998-1999. Calverton, Maryland USA : Institut National de la Statistique et ORC Macro; 2001 (in French).
CIV	Côte d'Ivoire	2000	2005	6.4	Institut National de la Statistique (INS) et Ministère de la Lutte contre le Sida [Côte d'Ivoire] et ORC Macro. Enquête sur les Indicateurs du Sida, Côte d'Ivoire 2005. Calverton, Maryland, USA: INS et ORC Macro; 2006 (in French). DHS 2005.

ISO Code	Country	Coverage start year	Coverage end year	Caesarean section rate (%)	References
CIV	Côte d'Ivoire	2006	2012	2.7	Institut National de la Statistique (INS), ICF International. Enquête Démographique et de Santé et à Indicateurs Multiples de Côte d'Ivoire 2011-2012. Calverton, Maryland, USA: INS et ICF International; 2012 (in French).
CIV	Côte d'Ivoire	2014	2016	3.3	Enquête par grappes à indicateurs multiples - Côte d'Ivoire 2016 (MICS 2016). Abidjan: Ministère du Plan et du Développement [Côte d'Ivoire], Institut National de la Statistique (INS) [Côte d'Ivoire], Fonds des Nations Unies pour l'Enfance (UNICEF), le Fonds mondial [the Global Fund], Contrat de Désendettement et de Développement, Fonds des Nations unies pour la population (FNUAP/UNFPA); 2017 (in French).
HRV	Croatia	1990	1990	9.3	Dražančić A, Rodin U, Filipović-Grčić B. Perinatalna Zaštita U Hrvatskoj. Jučer, danas, sutra [Perinatal Care in Croatia. Yesterday, today, tomorrow]. Liječ Vjesn. 2007;129:87–99.
HRV	Croatia	1991	1991	8.8	Dražančić A, Rodin U, Filipović-Grčić B. Perinatalna Zaštita U Hrvatskoj. Jučer, danas, sutra [Perinatal Care in Croatia. Yesterday, today, tomorrow]. Liječ Vjesn. 2007;129:87–99.
HRV	Croatia	1992	1992	7.7	Dražančić A, Rodin U, Filipović-Grčić B. Perinatalna Zaštita U Hrvatskoj. Jučer, danas, sutra [Perinatal Care in Croatia. Yesterday, today, tomorrow]. Liječ Vjesn. 2007;129:87–99.
HRV	Croatia	1993	1993	8.3	Dražančić A, Rodin U, Filipović-Grčić B. Perinatalna Zaštita U Hrvatskoj. Jučer, danas, sutra [Perinatal Care in Croatia. Yesterday, today, tomorrow]. Liječ Vjesn. 2007;129:87–99.
HRV	Croatia	1994	1994	8.6	Dražančić A, Rodin U, Filipović-Grčić B. Perinatalna Zaštita U Hrvatskoj. Jučer, danas, sutra [Perinatal Care in Croatia. Yesterday, today, tomorrow]. Liječ Vjesn. 2007;129:87–99.
HRV	Croatia	1995	1995	9.2	Dražančić A, Rodin U, Filipović-Grčić B. Perinatalna Zaštita U Hrvatskoj. Jučer, danas, sutra [Perinatal Care in Croatia. Yesterday, today, tomorrow]. Liječ Vjesn. 2007;129:87–99.

ISO Code	Country	Coverage start year	Coverage end year	Caesarean section rate (%)	References
HRV	Croatia	1996	1996	9.3	Dražančić A, Rodin U, Filipović-Grčić B. Perinatalna Zaštita U Hrvatskoj. Jučer, danas, sutra [Perinatal Care in Croatia. Yesterday, today, tomorrow]. Liječ Vjesn. 2007;129:87–99.
HRV	Croatia	1997	1997	9.9	Dražančić A, Rodin U, Filipović-Grčić B. Perinatalna Zaštita U Hrvatskoj. Jučer, danas, sutra [Perinatal Care in Croatia. Yesterday, today, tomorrow]. Liječ Vjesn. 2007;129:87–99.
HRV	Croatia	1998	1998	10.8	Dražančić A, Rodin U, Filipović-Grčić B. Perinatalna Zaštita U Hrvatskoj. Jučer, danas, sutra [Perinatal Care in Croatia. Yesterday, today, tomorrow]. Liječ Vjesn. 2007;129:87–99.
HRV	Croatia	1999	1999	11.5	Dražančić A, Rodin U, Filipović-Grčić B. Perinatalna Zaštita U Hrvatskoj. Jučer, danas, sutra [Perinatal Care in Croatia. Yesterday, today, tomorrow]. Liječ Vjesn. 2007;129:87–99.
HRV	Croatia	2000	2000	12.4	Dražančić A, Rodin U, Filipović-Grčić B. Perinatalna Zaštita U Hrvatskoj. Jučer, danas, sutra [Perinatal Care in Croatia. Yesterday, today, tomorrow]. Liječ Vjesn. 2007;129:87–99.
HRV	Croatia	2001	2001	13.8	Dražančić A, Rodin U, Filipović-Grčić B. Perinatalna Zaštita U Hrvatskoj. Jučer, danas, sutra [Perinatal Care in Croatia. Yesterday, today, tomorrow]. Liječ Vjesn. 2007;129:87–99.
HRV	Croatia	2002	2002	14.8	Dražančić A, Rodin U, Filipović-Grčić B. Perinatalna Zaštita U Hrvatskoj. Jučer, danas, sutra [Perinatal Care in Croatia. Yesterday, today, tomorrow]. Liječ Vjesn. 2007;129:87–99.
HRV	Croatia	2003	2003	15.1	Dražančić A, Rodin U, Filipović-Grčić B. Perinatalna Zaštita U Hrvatskoj. Jučer, danas, sutra [Perinatal Care in Croatia. Yesterday, today, tomorrow]. Liječ Vjesn. 2007;129:87–99.

Betran AP, *et al. BMJ Global Health* 2021; 6:e005671. doi: 10.1136/bmjgh-2021-005671

ISO Code	Country	Coverage start year	Coverage end year	Caesarean section rate (%)	References
HRV	Croatia	2004	2004	15.5	Dražančić A, Rodin U, Filipović-Grčić B. Perinatalna Zaštita U Hrvatskoj. Jučer, danas, sutra [Perinatal Care in Croatia. Yesterday, today, tomorrow]. Liječ Vjesn. 2007;129:87–99.
HRV	Croatia	2005	2005	16.0	European Health for All Database (HFA-DB) [online database]. World Health Organization (WHO) Regional Office for Europe; 2012 (http://data.euro.who.int/hfadb, accessed 20 August 2012).
HRV	Croatia	2006	2006	16.2	European Health for All Database (HFA-DB) [online database]. World Health Organization (WHO) Regional Office for Europe; 2012 (http://data.euro.who.int/hfadb, accessed 20 August 2012).
HRV	Croatia	2007	2007	16.4	European Health for All Database (HFA-DB) [online database]. World Health Organization (WHO) Regional Office for Europe; 2012 (http://data.euro.who.int/hfadb, accessed 20 August 2012).
HRV	Croatia	2008	2008	17.1	European Health for All Database (HFA-DB) [online database]. World Health Organization (WHO) Regional Office for Europe; 2012 (http://data.euro.who.int/hfadb, accessed 20 August 2012).
HRV	Croatia	2009	2009	17.5	European Health for All Database (HFA-DB) [online database]. World Health Organization (WHO) Regional Office for Europe; 2012 (http://data.euro.who.int/hfadb, accessed 20 August 2012).
HRV	Croatia	2010	2010	18.7	European Health for All Database (HFA-DB) [online database]. World Health Organization (WHO) Regional Office for Europe; 2012 (http://data.euro.who.int/hfadb, accessed 20 August 2012).
HRV	Croatia	2012	2012	18.8	Croatian Health Service Yearbook 2012. Zagreb: Croatian National Institute of Public Health; 2013.

ISO Code	Country	Coverage start year	Coverage end year	Caesarean section rate (%)	References
HRV	Croatia	2013	2013	19.1	Croatian Health Service Yearbook 2013. Zagreb: Croatian National Institute of Public Health; 2014.
HRV	Croatia	2014	2014	19.9	Croatian Health Service Yearbook 2014. Zagreb: Croatian Institute of Public Health; 2015.
HRV	Croatia	2015	2015	20.9	Croatian Health Statistics Yearbook 2015. Zagreb: Croatian Institute of Public Health; 2016.
HRV	Croatia	2016	2016	23.0	Croatian Health Statistics Yearbook 2016. Zagreb: Croatian National Institute of Public Health; 2017.
CUB	Cuba	1990	1990	19.4	Vázquez Cabrera J. Cesárea. Análisis crítico y recomendaciones para disminuir su morbilidad. La Habana: Editorial Ciencias Médicas; 2010 (in Spanish).
CUB	Cuba	1991	1991	20.1	Vázquez Cabrera J. Cesárea. Análisis crítico y recomendaciones para disminuir su morbilidad. La Habana: Editorial Ciencias Médicas; 2010 (in Spanish).
CUB	Cuba	1992	1992	19.7	Vázquez Cabrera J. Cesárea. Análisis crítico y recomendaciones para disminuir su morbilidad. La Habana: Editorial Ciencias Médicas; 2010 (in Spanish).
CUB	Cuba	1993	1993	19.4	Vázquez Cabrera J. Cesárea. Análisis crítico y recomendaciones para disminuir su morbilidad. La Habana: Editorial Ciencias Médicas; 2010 (in Spanish).

ISO Code	Country	Coverage start year	Coverage end year	Caesarean section rate (%)	References
CUB	Cuba	1994	1994	20.5	Vázquez Cabrera J. Cesárea. Análisis crítico y recomendaciones para disminuir su morbilidad. La Habana: Editorial Ciencias Médicas; 2010 (in Spanish).
CUB	Cuba	1995	1995	22.3	Vázquez Cabrera J. Cesárea. Análisis crítico y recomendaciones para disminuir su morbilidad. La Habana: Editorial Ciencias Médicas; 2010 (in Spanish).
CUB	Cuba	1996	1996	15.6	Vázquez Cabrera J. Cesárea. Análisis crítico y recomendaciones para disminuir su morbilidad. La Habana: Editorial Ciencias Médicas; 2010 (in Spanish).
CUB	Cuba	1997	1997	23.0	Vázquez Cabrera J. Cesárea. Análisis crítico y recomendaciones para disminuir su morbilidad. La Habana: Editorial Ciencias Médicas; 2010 (in Spanish).
CUB	Cuba	1998	1998	24.1	Vázquez Cabrera J. Cesárea. Análisis crítico y recomendaciones para disminuir su morbilidad. La Habana: Editorial Ciencias Médicas; 2010 (in Spanish).
CUB	Cuba	1999	1999	25.0	Vázquez Cabrera J. Cesárea. Análisis crítico y recomendaciones para disminuir su morbilidad. La Habana: Editorial Ciencias Médicas; 2010 (in Spanish).
CUB	Cuba	2000	2000	26.4	Vázquez Cabrera J. Cesárea. Análisis crítico y recomendaciones para disminuir su morbilidad. La Habana: Editorial Ciencias Médicas; 2010 (in Spanish).
CUB	Cuba	2001	2001	26.5	Vázquez Cabrera J. Cesárea. Análisis crítico y recomendaciones para disminuir su morbilidad. La Habana: Editorial Ciencias Médicas; 2010 (in Spanish).

Betran AP, *et al. BMJ Global Health* 2021; 6:e005671. doi: 10.1136/bmjgh-2021-005671

ISO Code	Country	Coverage start year	Coverage end year	Caesarean section rate (%)	References
CUB	Cuba	2002	2002	28.1	Vázquez Cabrera J. Cesárea. Análisis crítico y recomendaciones para disminuir su morbilidad. La Habana: Editorial Ciencias Médicas; 2010 (in Spanish).
CUB	Cuba	2003	2003	31.5	Vázquez Cabrera J. Cesárea. Análisis crítico y recomendaciones para disminuir su morbilidad. La Habana: Editorial Ciencias Médicas; 2010 (in Spanish).
CUB	Cuba	2004	2004	34.5	Vázquez Cabrera J. Cesárea. Análisis crítico y recomendaciones para disminuir su morbilidad. La Habana: Editorial Ciencias Médicas; 2010 (in Spanish).
CUB	Cuba	2012	2014	40.4	Dirección de Registros Médicos y Estadísticas de Salud, Ministerio de Salud Pública, Fondo de las Naciones Unidas para la Infancia (UNICEF). Encuesta de Indicadores Múltiples por Conglomerados. Cuba, 2014. Informe final. La Habana, Cuba: Dirección de Registros Médicos y Estadísticas de Salud, Ministerio de Salud Pública, UNICEF; 2015 (in Spanish).
CYP	Cyprus	2007	2007	50.9	Perinatal Health Survey 2007. Nicosia: Statistical Service of Cyprus (CYSTAT); 2010 (in Greek).
CYP	Cyprus	2008	2008	53.4	Cyprus Public and Private Maternity Units. Perinatal Health Indicators for the Year 2014. Nicosia: Ministry of Health of the Republic of Cyprus; 2016.
CYP	Cyprus	2009	2009	52.2	Cyprus Public and Private Maternity Units. Perinatal Health Indicators for the Year 2014. Nicosia: Ministry of Health of the Republic of Cyprus; 2016.
CYP	Cyprus	2010	2010	53.7	Cyprus Public and Private Maternity Units. Perinatal Health Indicators for the Year 2014. Nicosia: Ministry of Health of the Republic of Cyprus; 2016.

ISO Code	Country	Coverage start year	Coverage end year	Caesarean section rate (%)	References
CYP	Cyprus	2011	2011	53.3	Cyprus Public and Private Maternity Units. Perinatal Health Indicators for the Year 2014. Nicosia: Ministry of Health of the Republic of Cyprus; 2016.
CYP	Cyprus	2012	2012	52.5	Cyprus Public and Private Maternity Units. Perinatal Health Indicators for the Year 2014. Nicosia: Ministry of Health of the Republic of Cyprus; 2016.
CYP	Cyprus	2013.0	2013.0	55.3	Cyprus Public and Private Maternity Units. Perinatal Health Indicators for the Year 2014. Nicosia: Ministry of Health of the Republic of Cyprus; 2016.
CYP	Cyprus	2014	2014	56.9	Cyprus Public and Private Maternity Units. Perinatal Health Indicators for the Year 2014. Nicosia: Ministry of Health of the Republic of Cyprus; 2016.
CYP	Cyprus	2015	2015	56.9	Cyprus Public and Private Maternity Units. Important Perinatal Health Indicators for the Year 2015. Nicosia: Ministry of Health of the Republic of Cyprus; 2017.
CYP	Cyprus	2016	2016	55.3	Perinatal health report 2018. Important Perinatal Health Indicators. Cyprus Public and Private Maternity Units 2014-2016. Nicosia: Ministry of Health of the Republic of Cyprus; 2018.
CZE	Czechia	1990	1990	7.6	European Health for All Database (HFA-DB) [online database]. World Health Organization (WHO) Regional Office for Europe; 2012 (http://data.euro.who.int/hfadb, accessed 20 August 2012).
CZE	Czechia	1991	1991	8.4	European Health for All Database (HFA-DB) [online database]. World Health Organization (WHO) Regional Office for Europe; 2012 (http://data.euro.who.int/hfadb, accessed 20 August 2012).

Betran AP, *et al. BMJ Global Health* 2021; 6:e005671. doi: 10.1136/bmjgh-2021-005671

ISO Code	Country	Coverage start year	Coverage end year	Caesarean section rate (%)	References
CZE	Czechia	1993	1993	9.1	European Health for All Database (HFA-DB) [online database]. World Health Organization (WHO) Regional Office for Europe; 2012 (http://data.euro.who.int/hfadb, accessed 20 August 2012).
CZE	Czechia	1994	1994	10.1	European Health for All Database (HFA-DB) [online database]. World Health Organization (WHO) Regional Office for Europe; 2012 (http://data.euro.who.int/hfadb, accessed 20 August 2012).
CZE	Czechia	1995	1995	11.2	European Health for All Database (HFA-DB) [online database]. World Health Organization (WHO) Regional Office for Europe; 2012 (http://data.euro.who.int/hfadb, accessed 20 August 2012).
CZE	Czechia	1996	1996	11.6	European Health for All Database (HFA-DB) [online database]. World Health Organization (WHO) Regional Office for Europe; 2012 (http://data.euro.who.int/hfadb, accessed 20 August 2012).
CZE	Czechia	1997	1997	11.8	European Health for All Database (HFA-DB) [online database]. World Health Organization (WHO) Regional Office for Europe; 2012 (http://data.euro.who.int/hfadb, accessed 20 August 2012).
CZE	Czechia	1998	1998	12.3	European Health for All Database (HFA-DB) [online database]. World Health Organization (WHO) Regional Office for Europe; 2012 (http://data.euro.who.int/hfadb, accessed 20 August 2012).
CZE	Czechia	1999	1999	12.6	Rodička a novorozenec 1999 [Mother and Newborn 1999]. Prague: Ústav zdravotnických informací a statistiky ČR [Institute of Health Information and Statistics of the Czech Republic]; 2001 (in Czech).
CZE	Czechia	2000	2000	13.8	Rodička a novorozenec 2000 [Mother and Newborn 2000]. Prague: Ústav zdravotnických informací a statistiky ČR [Institute of Health Information and Statistics of the Czech Republic]; 2001 (in Czech).

ISO Code	Country	Coverage start year	Coverage end year	Caesarean section rate (%)	References
CZE	Czechia	2001	2001	14.1	Rodička a novorozenec 2001 [Mother and Newborn 2001]. Prague: Ústav zdravotnických informací a statistiky ČR [Institute of Health Information and Statistics of the Czech Republic]; 2002 (in Czech).
CZE	Czechia	2002	2002	14.9	Rodička a novorozenec 2002 [Mother and Newborn 2002]. Prague: Ústav zdravotnických informací a statistiky ČR [Institute of Health Information and Statistics of the Czech Republic]; 2004 (in Czech).
CZE	Czechia	2003	2003	16.3	Rodička a novorozenec 2003 [Mother and Newborn 2003]. Prague: Ústav zdravotnických informací a statistiky ČR [Institute of Health Information and Statistics of the Czech Republic]; 2004 (in Czech).
CZE	Czechia	2004	2004	17.2	Rodička a novorozenec 2004 [Mother and Newborn 2004]. Prague: Ústav zdravotnických informací a statistiky ČR [Institute of Health Information and Statistics of the Czech Republic]; 2005 (in Czech).
CZE	Czechia	2005	2005	18.3	Rodička a novorozenec 2005 [Mother and Newborn 2005]. Prague: Ústav zdravotnických informací a statistiky ČR [Institute of Health Information and Statistics of the Czech Republic]; 2006 (in Czech).
CZE	Czechia	2006	2006	19.6	Rodička a novorozenec 2006 [Mother and Newborn 2006]. Prague: Ústav zdravotnických informací a statistiky ČR [Institute of Health Information and Statistics of the Czech Republic]; 2007 (in Czech).
CZE	Czechia	2007	2007	21.1	Rodička a novorozenec 2007 [Mother and Newborn 2007]. Prague: Ústav zdravotnických informací a statistiky ČR [Institute of Health Information and Statistics of the Czech Republic]; 2008 (in Czech).
CZE	Czechia	2008	2008	21.9	Rodička a novorozenec 2008 [Mother and Newborn 2008]. Prague: Ústav zdravotnických informací a statistiky ČR [Institute of Health Information and Statistics of the Czech Republic]; 2009 (in Czech).

ISO Code	Country	Coverage start year	Coverage end year	Caesarean section rate (%)	References
CZE	Czechia	2009	2009	22.7	Rodička a novorozenec 2009 [Mother and Newborn 2009]. Prague: Ústav zdravotnických informací a statistiky ČR [Institute of Health Information and Statistics of the Czech Republic]; 2009 (in Czech).
CZE	Czechia	2010	2010	24.1	Rodička a novorozenec 2010 [Mother and Newborn 2010]. Prague: Ústav zdravotnických informací a statistiky ČR [Institute of Health Information and Statistics of the Czech Republic]; 2011 (in Czech).
CZE	Czechia	2011	2011	24.7	Rodička a novorozenec 2011 [Mother and Newborn 2011]. Prague: Ústav zdravotnických informací a statistiky ČR [Institute of Health Information and Statistics of the Czech Republic]; 2012 (in Czech).
CZE	Czechia	2012	2012	25.9	Rodička a novorozenec 2012 [Mother and Newborn 2012]. Prague: Ústav zdravotnických informací a statistiky ČR [Institute of Health Information and Statistics of the Czech Republic]; 2013 (in Czech).
CZE	Czechia	2013	2013	26.4	Rodička a novorozenec 2013 [Mother and Newborn 2013]. Prague: Ústav zdravotnických informací a statistiky ČR [Institute of Health Information and Statistics of the Czech Republic]; 2014 (in Czech).
CZE	Czechia	2014	2014	26.9	Rodička a novorozenec 2014-2015 [Mother and Newborn 2014-2015]. Prague: Ústav zdravotnických informací a statistiky ČR [Institute of Health Information and Statistics of the Czech Republic]; 2017 (in Czech).
CZE	Czechia	2015	2015	26.9	Rodička a novorozenec 2014-2015 [Mother and Newborn 2014-2015]. Prague: Ústav zdravotnických informací a statistiky ČR [Institute of Health Information and Statistics of the Czech Republic]; 2017 (in Czech).
PRK	Democratic People's Republic of Korea	2007	2009	12.5	Central Bureau of Statistics (CBS), United Nations Children's Fund (UNICEF). DPR Korea Multiple Indicator Cluster Survey 2009, Final Report. Pyongyang, DPR Korea: Central Bureau of Statistics (CBS), UNICEF; 2010.

ISO Code	Country	Coverage start year	Coverage end year	Caesarean section rate (%)	References
PRK	Democratic People's Republic of Korea	2015	2017	12.9	DPR Korea Multiple Indicator Cluster Survey 2017, Survey Findings Report. Pyongyang: Central Bureau of Statistics of the DPR Korea, United Nations Children's Fund (UNICEF); 2017.
COD	Democratic Republic of the Congo	2002	2007	4.0	Ministère du Plan, Macro International. Enquête Démographique et de Santé, République Démocratique du Congo 2007. Calverton, Maryland, U.S.A. : Ministère du Plan, Macro International; 2008 (in French).
COD	Democratic Republic of the Congo	2008	2010	7.2	Institut National de la Statistique, Fonds des Nations Unies pour l'Enfance (UNICEF). Enquête par Grappes à Indicateurs Multiples en République Démocratique du Congo (MICS-RDC 2010), Rapport Final. République Démocratique du Congo; 2011 (in French).
COD	Democratic Republic of the Congo	2008	2014	5.1	Ministère du Plan et Suivi de la Mise en œuvre de la Révolution de la Modernité (MPSMRM), Ministère de la Santé Publique (MSP), ICF International. Enquête Démographique et de Santé en République Démocratique du Congo 2013-2014. Rockville, Maryland, USA: MPSMRM, MSP, ICF International; 2014 (in French).
DNK	Denmark	1990	1990	12.5	Sundhedsstyrelsen [National Board of Health, Denmark]. Nye tal fra Sundhedsstyrelsen - Fødselsregisteret 1973-2003, 2004:23 [New figures from the National Board of Health, Denmark - Birth Registry 1973-2003, 2004:23]. Copenhagen: Sundhedsstyrelsen; 2004 (in Danish).
DNK	Denmark	1991	1991	12.4	Sundhedsstyrelsen [National Board of Health, Denmark]. Nye tal fra Sundhedsstyrelsen - Fødselsregisteret 1973-2003, 2004:23 [New figures from the National Board of Health, Denmark - Birth Registry 1973-2003, 2004:23]. Copenhagen: Sundhedsstyrelsen; 2004 (in Danish).
DNK	Denmark	1992	1992	12.3	Sundhedsstyrelsen [National Board of Health, Denmark]. Nye tal fra Sundhedsstyrelsen - Fødselsregisteret 1973-2003, 2004:23 [New figures from the National Board of Health, Denmark - Birth Registry 1973-2003, 2004:23]. Copenhagen: Sundhedsstyrelsen; 2004 (in Danish).
DNK	Denmark	1993	1993	12.7	Sundhedsstyrelsen [National Board of Health, Denmark]. Nye tal fra Sundhedsstyrelsen - Fødselsregisteret 1973-2003, 2004:23 [New figures from the National Board of Health, Denmark - Birth Registry 1973-2003, 2004:23]. Copenhagen: Sundhedsstyrelsen; 2004 (in Danish).

Betran AP, *et al. BMJ Global Health* 2021; 6:e005671. doi: 10.1136/bmjgh-2021-005671

ISO Code	Country	Coverage start year	Coverage end year	Caesarean section rate (%)	References
DNK	Denmark	1994	1994	12.7	Sundhedsstyrelsen [National Board of Health, Denmark]. Nye tal fra Sundhedsstyrelsen - Fødselsregisteret 1973-2003, 2004:23 [New figures from the National Board of Health, Denmark - Birth Registry 1973-2003, 2004:23]. Copenhagen: Sundhedsstyrelsen; 2004 (in Danish).
DNK	Denmark	1995	1995	12.6	Sundhedsstyrelsen [National Board of Health, Denmark]. Nye tal fra Sundhedsstyrelsen - Fødselsregisteret 1973-2003, 2004:23 [New figures from the National Board of Health, Denmark - Birth Registry 1973-2003, 2004:23]. Copenhagen: Sundhedsstyrelsen; 2004 (in Danish).
DNK	Denmark	1996	1996	12.9	Sundhedsstyrelsen [National Board of Health, Denmark]. Nye tal fra Sundhedsstyrelsen - Fødselsregisteret 1973-2003, 2004:23 [New figures from the National Board of Health, Denmark - Birth Registry 1973-2003, 2004:23]. Copenhagen: Sundhedsstyrelsen; 2004 (in Danish).
DNK	Denmark	1997	1997	13.6	Sundhedsstyrelsen [National Board of Health, Denmark].Nye tal fra Sundhedsstyrelsen - Fødselsregisteret 1997-2001, 2003:12 [New figures from the National Board of Health, Denmark - Birth Registry 1997-2001, 2003:12]. Copenhagen: Sundhedsstyrelsen; 2003 (in Danish).
DNK	Denmark	1998	1998	14.4	Sundhedsstyrelsen [National Board of Health, Denmark].Nye tal fra Sundhedsstyrelsen - Fødselsregisteret 1997-2001, 2003:12 [New figures from the National Board of Health, Denmark - Birth Registry 1997-2001, 2003:12]. Copenhagen: Sundhedsstyrelsen; 2003 (in Danish).
DNK	Denmark	1999	1999	15.0	Sundhedsstyrelsen [National Board of Health, Denmark].Nye tal fra Sundhedsstyrelsen - Fødselsregisteret 1997-2001, 2003:12 [New figures from the National Board of Health, Denmark - Birth Registry 1997-2001, 2003:12]. Copenhagen: Sundhedsstyrelsen; 2003 (in Danish).
DNK	Denmark	2000	2000	16.0	Sundhedsstyrelsen [National Board of Health, Denmark].Nye tal fra Sundhedsstyrelsen - Fødselsregisteret 1997-2001, 2003:12 [New figures from the National Board of Health, Denmark - Birth Registry 1997-2001, 2003:12]. Copenhagen: Sundhedsstyrelsen; 2003 (in Danish).
DNK	Denmark	2001	2001	17.8	Sundhedsstyrelsen [National Board of Health, Denmark].Nye tal fra Sundhedsstyrelsen - Fødselsregisteret 1997-2001, 2003:12 [New figures from the National Board of Health, Denmark - Birth Registry 1997-2001, 2003:12]. Copenhagen: Sundhedsstyrelsen; 2003 (in Danish).

Betran AP, *et al. BMJ Global Health* 2021; 6:e005671. doi: 10.1136/bmjgh-2021-005671

ISO Code	Country	Coverage start year	Coverage end year	Caesarean section rate (%)	References
DNK	Denmark	2002	2002	18.5	Sundhedsstyrelsen [National Board of Health, Denmark]. Nye tal fra Sundhedsstyrelsen - Fødselsregisteret 2003, 2004:4 [New figures from the National Board of Health, Denmark - Birth Registry 2003, 2004:4]. Copenhagen: Sundhedsstyrelsen; 2004 (in Danish).
DNK	Denmark	2003	2003	19.8	Sundhedsstyrelsen [National Board of Health, Denmark]. Nye tal fra Sundhedsstyrelsen - Fødselsregisteret 2004, 2005:4 [New figures from the National Board of Health, Denmark - Birth Registry 2004, 2005:4]. Copenhagen: Sundhedsstyrelsen; 2005 (in Danish).
DNK	Denmark	2004	2004	20.7	Sundhedsstyrelsen [National Board of Health, Denmark]. Nye tal fra Sundhedsstyrelsen - Fødselsregisteret 2004, 2005:4 [New figures from the National Board of Health, Denmark - Birth Registry 2004, 2005:4]. Copenhagen: Sundhedsstyrelsen; 2005 (in Danish).
DNK	Denmark	2006	2006	21.0	Sundhedsstyrelsen [National Board of Health, Denmark]. Nye tal fra Sundhedsstyrelsen - Fødselsregisteret 2007, 2008:1 [New figures from the National Board of Health, Denmark - Birth Registry 2007, 2008:1]. Copenhagen: Sundhedsstyrelsen; 2008 (in Danish).
DNK	Denmark	2007	2007	21.9	Sundhedsstyrelsen [National Board of Health, Denmark]. Nye tal fra Sundhedsstyrelsen - Fødselsregisteret 2007, 2008:1 [New figures from the National Board of Health, Denmark - Birth Registry 2007, 2008:1]. Copenhagen: Sundhedsstyrelsen; 2008 (in Danish).
DNK	Denmark	2008	2008	20.6	Sundhedsstyrelsen [National Board of Health, Denmark].Nye tal fra Sundhedsstyrelsen - Fødselsregisteret 1. Halvår 2008, 2008:8 [New figures from the National Board of Health, Denmark - Birth Registry 1. Half year 2008, 2008:8]. Copenhagen: Sundhedsstyrelsen; 2008 (in Danish).
DNK	Denmark	2009	2009	21.0	Sundhedsstyrelsen [National Board of Health, Denmark]. Fødselsstatistikken 2011 [Birth Statistics 2011]. Copenhagen: Sundhedsstyrelsen; 2012 (in Danish).
DNK	Denmark	2010	2010	21.2	Sundhedsstyrelsen [National Board of Health, Denmark]. Fødselsstatistikken 2011 [Birth Statistics 2011]. Copenhagen: Sundhedsstyrelsen; 2012 (in Danish).

ISO Code	Country	Coverage start year	Coverage end year	Caesarean section rate (%)	References
DNK	Denmark	2011	2011	21.8	Sundhedsstyrelsen [National Board of Health, Denmark]. Fødselsstatistikken 2011 [Birth Statistics 2011]. Copenhagen: Sundhedsstyrelsen; 2012 (in Danish).
DNK	Denmark	2012	2012	21.2	European Health for All Database (HFA-DB) [online database]. World Health Organization (WHO) Regional Office for Europe; 2016 (http://data.euro.who.int/hfadb, accessed 8 August 2016).
DNK	Denmark	2012	2012	21.2	Statens Serum Institut. Fødselsstatistikken. Tal og analyser 2012 [Birth Statistics. Numbers and analysis 2012]. Copenhagen: Statens Serum Institut; 2013 (in Danish).
DNK	Denmark	2015	2015	21.6	European Health Information Gateway. European Health for All database (HFA-DB). Caesarean sections per 1000 live births [online database]. World Health Organization (WHO) Regional Office for Europe; 2018 (https://gateway.euro.who.int/en/indicators/hfa_596-7060-caesarean-sections-per-1000-live-births/, accessed 14 February 2019).
DNK	Denmark	2013	2013	22.0	Det Medicinske Fødselsregister (MFR), MFR-kuben, Institutional deliveries [online database]. Copenhagen: Sundhedsdatastyrelsen [National Board of Health Data, Denmark]; 2018 (http://www.esundhed.dk/sundhedsregistre/MFR/Sider/MFR06A.aspx, accessed 7 January 2018, in Danish).
DNK	Denmark	2014	2014	21.2	Det Medicinske Fødselsregister (MFR), MFR-kuben, Institutional deliveries [online database]. Copenhagen: Sundhedsdatastyrelsen [National Board of Health Data, Denmark]; 2018 (http://www.esundhed.dk/sundhedsregistre/MFR/Sider/MFR06A.aspx, accessed 7 January 2018, in Danish).
DNK	Denmark	2005	2005	20.5	Det Medicinske Fødselsregister (MFR), MFR-kuben, Institutional deliveries [online database]. Copenhagen: Sundhedsdatastyrelsen [National Board of Health Data, Denmark]; 2018 (http://www.esundhed.dk/sundhedsregistre/MFR/Sider/MFR06A.aspx, accessed 7 January 2018, in Danish).
DNK	Denmark	2016	2016	19.5	Det Medicinske Fødselsregister (MFR), MFR-kuben, Institutional deliveries [online database]. Copenhagen: Sundhedsdatastyrelsen [National Board of Health Data, Denmark]; 2018 (http://www.esundhed.dk/sundhedsregistre/MFR/Sider/MFR06A.aspx, accessed 7 January 2018, in Danish).

ISO Code	Country	Coverage start year	Coverage end year	Caesarean section rate (%)	References
DNK	Denmark	2017	2017	19.7	Det Medicinske Fødselsregister (MFR), MFR-kuben, Institutional deliveries [online database]. Copenhagen: Sundhedsdatastyrelsen [National Board of Health Data, Denmark]; 2019 (http://www.esundhed.dk/sundhedsregistre/MFR/Sider/MFR06A.aspx, accessed 12 February 2019, in Danish).
DNK	Denmark	2018	2018	19.1	Det Medicinske Fødselsregister (MFR), MFR-kuben, Institutional deliveries [online database]. Copenhagen: Sundhedsdatastyrelsen [National Board of Health Data, Denmark]; 2020 (http://www.esundhed.dk/sundhedsregistre/MFR/Sider/MFR06A.aspx, accessed 20 February 2020, in Danish).
DJI	Djibouti	1997	2002	6.1	Enquête Djiboutienne sur la Santé de la Famille. PAPFAM - Rapport Final. Djibouti: Gouvernement de Djibouti, Ligue des États Arabes; 2004 (in French).
DJI	Djibouti	2007	2012	11.0	Deuxième Enquête Djiboutienne sur la Santé de la Famille (EDSF/PAPFAM 2 – 2012). Le Caire: Ministère de la Santé Djibouti, Direction de la Statistique et des Etudes Démographiques Djibouti, Ligue des États Arabes (Projet Pan Arabe sur la Santé de la Famille); 2012 (in French).
DOM	Dominican Republic	1991	1996	25.9	Centro de Estudios Sociales y Demográficos (CESDEM), Asociación Dominicana Pro-Bienestar de la Familia (PROFAMILIA), Oficina Nacional de Planificación (ONAPLAN), Macro International Inc. Encuesta Demográfica y de Salud de la República Dominicana 1996 (ENDESA-96). Columbia, Maryland, USA: Macro International Inc.; 1997 (in Spanish).
DOM	Dominican Republic	1994	1999	27.9	Centro de Estudios Sociales y Demográficos (CESDEM), USAID, Macro International Inc. Encuesta Experimental de Demográfica y de Salud 1999. Calverton, Maryland, USA: Macro International Inc.; 2001 (in Spanish).
DOM	Dominican Republic	1997	2002	31.3	Molina Achécar M, Ramirez N, Polanco JJ, Ochoa LH, Lerebours G, Garcia B (Centro de Estudios Sociales y Demográficos - CESDEM/República Dominicana, ORC Macro). República Dominicana Encuesta Demográfica y de Salud (ENDESA) 2002. Santo Domingo, República Dominicana: Centro de Estudios Sociales y Demográficos - CESDEM/República Dominicana, ORC Macro; 2003 (in Spanish).
DOM	Dominican Republic	2003	2003	31.5	Dominicana en cifras 2008. Santo Domingo: Oficina Nacional de Estadística (ONE); 2008 (in Spanish).

ISO Code	Country	Coverage start year	Coverage end year	Caesarean section rate (%)	References
DOM	Dominican Republic	2006	2006	32.9	Dominicana en cifras 2008. Santo Domingo: Oficina Nacional de Estadística (ONE); 2008 (in Spanish).
DOM	Dominican Republic	2007	2007	34.9	Dominicana en cifras 2008. Santo Domingo: Oficina Nacional de Estadística (ONE); 2008 (in Spanish).
DOM	Dominican Republic	2002	2007	41.9	Centro de Estudios Sociales y Demográficos (CESDEM), Macro International Inc. Encuesta Demográfica y de Salud 2007. Santo Domingo, República Dominicana: CESDEM, Macro International Inc.; 2008 (in Spanish).
DOM	Dominican Republic	2008	2008	36.2	Dominicana en cifras 2011. Santo Domingo: Oficina Nacional de Estadística (ONE); 2011 (in Spanish).
DOM	Dominican Republic	2009	2009	37.5	Anuario de Salud 2009. Santo Domingo: Oficina Nacional de Estadística (ONE); 2009 (in Spanish).
DOM	Dominican Republic	2008	2013	56.4	Centro de Estudios Sociales y Demográficos (CESDEM), ICF International. Encuesta Demográfica y de Salud 2013. Santo Domingo, República Dominicana: CESDEM, ICF International; 2014 (in Spanish).
DOM	Dominican Republic	2012	2012	58.1	Oficina Nacional de Estadísticas, Fondo de las Naciones Unidas para la Infancia (UNICEF). Encuesta Nacional de Hogares de Propósitos Múltiples - Encuesta de Indicadores Múltiples por Conglomerados 2014. Informe final. Santo Domingo, República Dominicana: Oficina Nacional de Estadística; 2016 (in Spanish).
ECU	Ecuador	1989	1994	17.1	Encuesta Demográfica y de Salud Materna e Infantil (ENDEMAIN) Ecuador 94. Informe General. Quito: Centro de Estudios de Población y Paternidad Responsable (CEPAR), Centros para el Control y la Prevención de Enfermedades [Centers for Disease Control and Prevention] (CDC); 1995 (in Spanish).

ISO Code	Country	Coverage start year	Coverage end year	Caesarean section rate (%)	References
ECU	Ecuador	1994	1999	19.9	Encuesta Demográfica y de Salud Materna e Infantil (ENDEMAIN) Ecuador 99. Informe General. Quito: Centro de Estudios de Población y Paternidad Responsable (CEPAR), Centros para el Control y la Prevención de Enfermedades [Centers for Disease Control and Prevention] (CDC); 2001 (in Spanish).
ECU	Ecuador	1999	2004	25.8	Encuesta Demográfica y de Salud Materna e Infantil (ENDEMAIN) Ecuador 2004. Informe Final. Quito: Centro de Estudios de Población y Paternidad Responsable (CEPAR), Centro de Estudios de Población y Desarrollo Social, Centros para el Control y la Prevención de Enfermedades [Centers for Disease Control and Prevention] (CDC); 2005 (in Spanish).
ECU	Ecuador	2006	2006	25.1	Producción Estadística MSP 2006-2014. Quito: Ministerio de Salud del Ecuador; 2015 (in Spanish).
ECU	Ecuador	2007	2007	26.6	Producción Estadística MSP 2006-2014. Quito: Ministerio de Salud del Ecuador; 2015 (in Spanish).
ECU	Ecuador	2008	2008	27.4	Producción Estadística MSP 2006-2014. Quito: Ministerio de Salud del Ecuador; 2015 (in Spanish).
ECU	Ecuador	2009	2009	27.5	Producción Estadística MSP 2006-2014. Quito: Ministerio de Salud del Ecuador; 2015 (in Spanish).
ECU	Ecuador	2010	2010	28.9	Producción Estadística MSP 2006-2014. Quito: Ministerio de Salud del Ecuador; 2015 (in Spanish).
ECU	Ecuador	2011	2011	30.5	Producción Estadística MSP 2006-2014. Quito: Ministerio de Salud del Ecuador; 2015 (in Spanish).

ISO Code	Country	Coverage start year	Coverage end year	Caesarean section rate (%)	References
ECU	Ecuador	2012	2012	38.6	Boletín Nacimientos por cesárea MSP 2012-2015. Quito: Ministerio de Salud Pública del Ecuador; 2016 (in Spanish).
ECU	Ecuador	2013	2013	48.8	Boletín Nacimientos por cesárea MSP 2012-2015. Quito: Ministerio de Salud Pública del Ecuador; 2016 (in Spanish).
ECU	Ecuador	2014	2014	50.6	Boletín Nacimientos por cesárea MSP 2012-2015. Quito: Ministerio de Salud Pública del Ecuador; 2016 (in Spanish).
ECU	Ecuador	2015	2015	49.0	Boletín Nacimientos por cesárea MSP 2012-2015. Quito: Ministerio de Salud Pública del Ecuador; 2016 (in Spanish).
ECU	Ecuador	2016	2016	46.2	Boletín Nacimientos por cesárea MSP 2013-2016. Quito: Ministerio de Salud Pública [Ecuador]; 2018 (in Spanish).
EGY	Egypt	1987	1992	4.6	EI-Zanaty FH, Sayed H AA, Zaky Hassan HM, Way AA (National Population Council [Egypt], Macro International Inc.). Egypt Demographic and Health Survey 1992. Calverton, Maryland [USA]: National Population Council [Egypt], Macro International Inc.; 1993.
EGY	Egypt	1990	1995	6.6	EI-Zanaty F, Hussein EM, Shawky GA, Way AA, Kishor S (National Population Council [Egypt], Macro International Inc.). Egypt Demographic and Health Survey 1995. Calverton, Maryland [USA]: National Population Council [Egypt], Macro International Inc.; 1996.
EGY	Egypt	1995	2000	10.3	El-Zanaty F, Way A (Ministry of Health and Population [Egypt], National Population Council, ORC Macro). Egypt Demographic and Health Survey 2000. Calverton, Maryland [USA]: Ministry of Health and Population [Egypt], National Population Council, ORC Macro; 2001.

ISO Code	Country	Coverage start year	Coverage end year	Caesarean section rate (%)	References
EGY	Egypt	2000	2005	19.9	El-Zanaty F, Way A (Ministry of Health and Population, National Population Council, El-Zanaty and Associates, ORC Macro). Egypt Demographic and Health Survey 2005. Cairo, Egypt: Ministry of Health and Population, National Population Council, El-Zanaty and Associates, ORC Macro; 2006.
EGY	Egypt	2003	2008	27.6	El-Zanaty F, Way A (Ministry of Health, El-Zanaty and Associates, Macro International). Egypt Demographic and Health Survey 2008. Cairo, Egypt: Ministry of Health, El-Zanaty and Associates, Macro International; 2009.
EGY	Egypt	2009	2014	51.8	Ministry of Health and Population [Egypt], El-Zanaty and Associates [Egypt], ICF International. Egypt Demographic and Health Survey 2014. Cairo, Egypt and Rockville, Maryland, USA: Ministry of Health and Population, ICF International; 2015.
SLV	El Salvador	1991	1993	13.4	Asociación Demográfica Salvadoreña (ADS), Centros para el Control y Prevención de Enfermedades (CDC), Agencia de los Estados Unidos para el Desarrollo Internacional (USAID), Ministerio de Salud Pública y Asistencia Social (MSPAS), MIPLAN, Instituto Salvadoreño del Seguro Social (ISSS), Dirección General de Estadística y Censos (DIGESTYC). Encuesta Nacional de Salud Familiar (FESAL) 93. Informe Final. San Salvador: ADS; 1994 (in Spanish).
SLV	El Salvador	1996	1998	15.7	Asociación Demográfica Salvadoreña (ADS), Comité Consultivo Interinstitucional (CCI), Centros para el Control y Prevención de Enfermedades (CDC), Agencia de los Estados Unidos para el Desarrollo Internacional (USAID), Fondo de las Naciones Unidas para la Infancia (UNICEF), Organización Panamericana de la Salud/Instituto de Nutrición de Centro América y Panamá (OPS/INCAP), Fondo de Población de las Naciones Unidas (FNUAP/UNFPA), Ministerio de Salud Pública y Asistencia Social (MSPAS), Ministerio de Relaciones Exteriores (M.RR.EE.), Instituto Salvadoreño del Seguro Social (ISSS), Dirección General de Estadística y Censos (DIGESTYC), Proyecto de Salud Materno Infantil (PROSAMI). Encuesta Nacional de Salud Familiar (FESAL) 98. Informe Final. San Salvador: ADS; 2000 (in Spanish).
SLV	El Salvador	1997	2003	22.0	Asociación Demográfica Salvadoreña (ADS), Comité Consultivo Interinstitucional (CCI), Centros para el Control y Prevención de Enfermedades (CDC), Agencia de los Estados Unidos para el Desarrollo Internacional (USAID). Encuesta Nacional de Salud Familiar (FESAL) 2002/03. Informe Final. San Salvador: ADS; 2004 (in Spanish).
SLV	El Salvador	2007	2007	28.2	Informe de Labores 2011-2012. San Salvador: Ministerio de Salud de El Salvador; 2012 (in Spanish).
SLV	El Salvador	2003	2008	24.9	Asociación Demográfica Salvadoreña (ADS), Comité Consultivo Interinstitucional (CCI), Centros para el Control y Prevención de Enfermedades (CDC), Agencia de los Estados Unidos para el Desarrollo Internacional (USAID). Encuesta Nacional de Salud Familiar (FESAL) 2008. Informe Final. San Salvador: ADS; 2009 (in Spanish).

ISO Code	Country	Coverage start year	Coverage end year	Caesarean section rate (%)	References
SLV	El Salvador	2008	2008	28.1	Informe de Labores 2011-2012. San Salvador: Ministerio de Salud de El Salvador; 2012 (in Spanish).
SLV	El Salvador	2009	2009	29.9	Informe de Labores 2011-2012. San Salvador: Ministerio de Salud de El Salvador; 2012 (in Spanish).
SLV	El Salvador	2010	2010	29.5	Informe de Labores 2011-2012. San Salvador: Ministerio de Salud de El Salvador; 2012 (in Spanish).
SLV	El Salvador	2011	2011	30.4	Informe de Labores 2011-2012. San Salvador: Ministerio de Salud de El Salvador; 2012 (in Spanish).
SLV	El Salvador	2012	2012	28.2	Informe de Labores 2012-2013. San Salvador: Ministerio de Salud de El Salvador; 2013 (in Spanish).
SLV	El Salvador	2014	2014	28.7	Informe de Labores 2016-2017. San Salvador: Ministerio de Salud de El Salvador; 2017 (in Spanish).
SLV	El Salvador	2015	2015	29.9	Informe de Labores 2016-2017. San Salvador: Ministerio de Salud de El Salvador; 2017 (in Spanish).
SLV	El Salvador	2016	2016	31.3	Informe de Labores 2016-2017. San Salvador: Ministerio de Salud de El Salvador; 2017 (in Spanish).

ISO Code	Country	Coverage start year	Coverage end year	Caesarean section rate (%)	References
SLV	El Salvador	2012	2014	32	Ministerio de Salud/Instituto Nacional de Salud, Fondo de las Naciones Unidas para la Infancia (UNICEF). Encuesta Nacional de Salud 2014 - Encuesta de Indicadores Múltiples por Conglomerados 2014, Resultados Principales. San Salvador, El Salvador: Ministerio de Salud e Instituto Nacional de Salud; 2015 (in Spanish).
SLV	El Salvador	2017	2017	29.9	Informe de Labores 2017-2018. San Salvador: Ministerio de Salud de El Salvador; 2018 (in Spanish).
SLV	El Salvador	2018	2018	29.8	Informe de Labores 2018-2019. San Salvador: Ministerio de Salud de El Salvador; 2019 (in Spanish).
GNQ	Equatorial Guinea	2006	2011	6.6	Ministerio de Sanidad y Bienestar Social, Ministerio de Economía, Planificación e Inversiones Públicas, ICF International. Encuesta Demográfica y de Salud (EDSGE-I) 2011. Calverton, Maryland, USA; 2012 (in Spanish).
ERI	Eritrea	1992	1995	1.6	National Statistics Office [Eritrea], Macro International Inc. Eritrea Demographic and Health Survey, 1995. Calverton, Maryland: National Statistics Office, Macro International Inc.; 1997.
ERI	Eritrea	1997	2002	2.7	National Statistics and Evaluation Office (NSEO) [Eritrea], ORC Macro. Eritrea Demographic and Health Survey 2002. Calverton, Maryland, USA: National Statistics and Evaluation Office, ORC Macro; 2003.
ERI	Eritrea	2005	2010	2.8	Eritrea Population and Health Survey 2010. Asmara: National Statistics Office (NSO), Eritrea, Fafo Institute for Applied International Studies (Fafo AIS); 2013.
EST	Estonia	1990	1990	6.3	European Health for All Database (HFA-DB) [online database]. World Health Organization (WHO) Regional Office for Europe; 2012 (http://data.euro.who.int/hfadb, accessed 20 August 2012).

ISO Code	Country	Coverage start year	Coverage end year	Caesarean section rate (%)	References
EST	Estonia	1991	1991	6.3	European Health for All Database (HFA-DB) [online database]. World Health Organization (WHO) Regional Office for Europe; 2012 (http://data.euro.who.int/hfadb, accessed 20 August 2012).
EST	Estonia	1992	1992	6.4	Estonian Medical Birth Registry (EMBR). Mode of delivery, 1992-2013 [website]. Tallinn: National Institute for Health Development [Estonia]; 2014 (http://www.tai.ee/en/r-and-d/registers/estonian-medical-birth-registry-and-estonian-abortion-registry/statistics, accessed 23 October 2014).
EST	Estonia	1993	1993	7.0	Estonian Medical Birth Registry (EMBR). Mode of delivery, 1992-2013 [website]. Tallinn: National Institute for Health Development [Estonia]; 2014 (http://www.tai.ee/en/r-and-d/registers/estonian-medical-birth-registry-and-estonian-abortion-registry/statistics, accessed 23 October 2014).
EST	Estonia	1994	1994	8.8	Estonian Medical Birth Registry (EMBR). Mode of delivery, 1992-2013 [website]. Tallinn: National Institute for Health Development [Estonia]; 2014 (http://www.tai.ee/en/r-and-d/registers/estonian-medical-birth-registry-and-estonian-abortion-registry/statistics, accessed 23 October 2014).
EST	Estonia	1995	1995	9.7	Estonian Medical Birth Registry (EMBR). Mode of delivery, 1992-2013 [website]. Tallinn: National Institute for Health Development [Estonia]; 2014 (http://www.tai.ee/en/r-and-d/registers/estonian-medical-birth-registry-and-estonian-abortion-registry/statistics, accessed 23 October 2014).
EST	Estonia	1996	1996	10.2	Estonian Medical Birth Registry (EMBR). Mode of delivery, 1992-2013 [website]. Tallinn: National Institute for Health Development [Estonia]; 2014 (http://www.tai.ee/en/r-and-d/registers/estonian-medical-birth-registry-and-estonian-abortion-registry/statistics, accessed 23 October 2014).
EST	Estonia	1997	1997	11.3	Estonian Medical Birth Registry (EMBR). Mode of delivery, 1992-2013 [website]. Tallinn: National Institute for Health Development [Estonia]; 2014 (http://www.tai.ee/en/r-and-d/registers/estonian-medical-birth-registry-and-estonian-abortion-registry/statistics, accessed 23 October 2014).
EST	Estonia	1998	1998	13.2	Estonian Medical Birth Registry (EMBR). Mode of delivery, 1992-2013 [website]. Tallinn: National Institute for Health Development [Estonia]; 2014 (http://www.tai.ee/en/r-and-d/registers/estonian-medical-birth-registry-and-estonian-abortion-registry/statistics, accessed 23 October 2014).

ISO Code	Country	Coverage start year	Coverage end year	Caesarean section rate (%)	References
EST	Estonia	1999	1999	14.0	Estonian Medical Birth Registry (EMBR). Mode of delivery, 1992-2013 [website]. Tallinn: National Institute for Health Development [Estonia]; 2014 (http://www.tai.ee/en/r-and-d/registers/estonian-medical-birth-registry-and-estonian-abortion-registry/statistics, accessed 23 October 2014).
EST	Estonia	2000	2000	14.6	Estonian Medical Birth Registry (EMBR). Mode of delivery, 1992-2013 [website]. Tallinn: National Institute for Health Development [Estonia]; 2014 (http://www.tai.ee/en/r-and-d/registers/estonian-medical-birth-registry-and-estonian-abortion-registry/statistics, accessed 23 October 2014).
EST	Estonia	2001	2001	15.6	Estonian Medical Birth Registry (EMBR). Mode of delivery, 1992-2013 [website]. Tallinn: National Institute for Health Development [Estonia]; 2014 (http://www.tai.ee/en/r-and-d/registers/estonian-medical-birth-registry-and-estonian-abortion-registry/statistics, accessed 23 October 2014).
EST	Estonia	2002	2002	15.1	Estonian Medical Birth Registry (EMBR). Mode of delivery, 1992-2013 [website]. Tallinn: National Institute for Health Development [Estonia]; 2014 (http://www.tai.ee/en/r-and-d/registers/estonian-medical-birth-registry-and-estonian-abortion-registry/statistics, accessed 23 October 2014).
EST	Estonia	2003	2003	16.5	Estonian Medical Birth Registry (EMBR). Mode of delivery, 1992-2013 [website]. Tallinn: National Institute for Health Development [Estonia]; 2014 (http://www.tai.ee/en/r-and-d/registers/estonian-medical-birth-registry-and-estonian-abortion-registry/statistics, accessed 23 October 2014).
EST	Estonia	2004	2004	17.3	Estonian Medical Birth Registry (EMBR). Mode of delivery, 1992-2013 [website]. Tallinn: National Institute for Health Development [Estonia]; 2014 (http://www.tai.ee/en/r-and-d/registers/estonian-medical-birth-registry-and-estonian-abortion-registry/statistics, accessed 23 October 2014).
EST	Estonia	2005	2005	19.0	Estonian Medical Birth Registry (EMBR). Mode of delivery, 1992-2013 [website]. Tallinn: National Institute for Health Development [Estonia]; 2014 (http://www.tai.ee/en/r-and-d/registers/estonian-medical-birth-registry-and-estonian-abortion-registry/statistics, accessed 23 October 2014).
EST	Estonia	2006	2006	19.2	Estonian Medical Birth Registry (EMBR). Mode of delivery, 1992-2013 [website]. Tallinn: National Institute for Health Development [Estonia]; 2014 (http://www.tai.ee/en/r-and-d/registers/estonian-medical-birth-registry-and-estonian-abortion-registry/statistics, accessed 23 October 2014).

ISO Code	Country	Coverage start year	Coverage end year	Caesarean section rate (%)	References
EST	Estonia	2007	2007	20.1	Estonian Medical Birth Registry (EMBR). Mode of delivery, 1992-2013 [website]. Tallinn: National Institute for Health Development [Estonia]; 2014 (http://www.tai.ee/en/r-and-d/registers/estonian-medical-birth-registry-and-estonian-abortion-registry/statistics, accessed 23 October 2014).
EST	Estonia	2008	2008	20.2	Estonian Medical Birth Registry (EMBR). Mode of delivery, 1992-2013 [website]. Tallinn: National Institute for Health Development [Estonia]; 2014 (http://www.tai.ee/en/r-and-d/registers/estonian-medical-birth-registry-and-estonian-abortion-registry/statistics, accessed 23 October 2014).
EST	Estonia	2009	2009	20.9	Estonian Medical Birth Registry (EMBR). Mode of delivery, 1992-2013 [website]. Tallinn: National Institute for Health Development [Estonia]; 2014 (http://www.tai.ee/en/r-and-d/registers/estonian-medical-birth-registry-and-estonian-abortion-registry/statistics, accessed 23 October 2014).
EST	Estonia	2010	2010	20.4	Estonian Medical Birth Registry (EMBR). Mode of delivery, 1992-2013 [website]. Tallinn: National Institute for Health Development [Estonia]; 2014 (http://www.tai.ee/en/r-and-d/registers/estonian-medical-birth-registry-and-estonian-abortion-registry/statistics, accessed 23 October 2014).
EST	Estonia	2011	2011	20.2	Estonian Medical Birth Registry (EMBR). Mode of delivery, 1992-2013 [website]. Tallinn: National Institute for Health Development [Estonia]; 2014 (http://www.tai.ee/en/r-and-d/registers/estonian-medical-birth-registry-and-estonian-abortion-registry/statistics, accessed 23 October 2014).
EST	Estonia	2012	2012	19.9	Estonian Medical Birth Registry (EMBR). SR 45: Mode of delivery by country: 2012 [website]. Tallinn: National Institute for Health Development [Estonia]; 2013 (http://www.tai.ee/en/r-and-d/registers/estonian-medical-birth-registry-and-estonian-abortion-registry/statistics, accessed 15 October 2013).
EST	Estonia	2013	2013	19.7	Estonian Medical Birth Registry (EMBR). Mode of delivery, 1992-2013 [website]. Tallinn: National Institute for Health Development [Estonia]; 2014 (http://www.tai.ee/en/r-and-d/registers/estonian-medical-birth-registry-and-estonian-abortion-registry/statistics, accessed 23 October 2014).
EST	Estonia	2014	2014	19.9	Estonian Medical Birth Registry (EMBR). Mode of delivery, 1992-2016 [website]. Tallinn: National Institute for Health Development [Estonia]; 2017 (http://www.tai.ee/en/r-and-d/registers/estonian-medical-birth-registry-and-estonian-abortion-registry/statistics, accessed 2 November 2017).

ISO Code	Country	Coverage start year	Coverage end year	Caesarean section rate (%)	References
EST	Estonia	2015	2015	18.7	Estonian Medical Birth Registry (EMBR). Mode of delivery,1992-2016 [website]. Tallinn: National Institute for Health Development [Estonia]; 2017 (http://www.tai.ee/en/r-and-d/registers/estonian-medical-birth-registry-and-estonian-abortion-registry/statistics, accessed 2 November 2017).
EST	Estonia	2016	2016	20.3	Estonian Medical Birth Registry (EMBR). Mode of delivery, 1992-2016 [website]. Tallinn: National Institute for Health Development [Estonia]; 2017 (http://www.tai.ee/en/r-and-d/registers/estonian-medical-birth-registry-and-estonian-abortion-registry/statistics, accessed 2 November 2017).
EST	Estonia	2017	2017	19.9	Estonian Medical Birth Registry (EMBR). Mode of delivery, 1992-2017 [website]. Tallinn: National Institute for Health Development [Estonia]; 2019 (https://www.tai.ee/en/r-and-d/registers/estonian-medical-birth-registry-and-estonian-abortion-registry/statistical-data-of-embr-and-ear, accessed 13 February 2019).
EST	Estonia	2018	2018	19.1	Health statistics and health research database. SR45: Mode of delivery by health care provider's county [online database]. Tallinn: National Institute for Health Development [Estonia]; 2019 (http://pxweb.tai.ee/PXWeb2015/pxweb/en/01Rahvastik/01Rahvastik__02Synnid/SR45.px/table/tableViewLayout2/?rxid=9de2b5d7-dedf-4184-b3e9-cc2fcae6c610, accessed 20 February 2020).
ETH	Ethiopia	1995	2000	0.7	Central Statistical Authority [Ethiopia], ORC Macro. Ethiopia Demographic and Health Survey 2000. Addis Ababa, Ethiopia and Calverton, Maryland, USA: Central Statistical Authority, ORC Macro; 2001.
ETH	Ethiopia	2000	2005	1.0	Central Statistical Agency, ORC Macro. Ethiopia Demographic and Health Survey 2005. Addis Ababa, Ethiopia and Calverton, Maryland, USA: Central Statistical Agency, ORC Macro; 2006.
ETH	Ethiopia	2005	2011	1.5	Central Statistical Agency [Ethiopia], ICF International. Ethiopia Demographic and Health Survey 2011. Addis Ababa, Ethiopia and Calverton, Maryland, USA: Central Statistical Agency, ICF International; 2012.
ETH	Ethiopia	2009	2014	2.1	Ethiopia Mini Demographic and Health Survey 2014. Addis Ababa: Central Statistical Agency [Ethiopia]; 2014.

ISO Code	Country	Coverage start year	Coverage end year	Caesarean section rate (%)	References
ETH	Ethiopia	2011	2016	1.9	Central Statistical Agency (CSA) [Ethiopia], ICF. Ethiopia Demographic and Health Survey 2016. Addis Ababa, Ethiopia, and Rockville, Maryland, USA: CSA , ICF; 2016.
FIN	Finland	1990	1990	13.7	Synnyttäjät, synnytykset ja vastasyntyneet 2002 - Parturients, births and newborn infants 2002. Statistical Summary 24/2003. Helsinki: National Research and Development Centre for Welfare and Health; 2003.
FIN	Finland	1991	1991	14.3	Synnyttäjät, synnytykset ja vastasyntyneet 2000 - tiedonantajapalaute 16/2001 - Parturients, births and newborn infants 2000 - data supplier feedback 16/2001. Helsinki: STAKES/Stakes Information, Welfare and Health care Statistics; 2001.
FIN	Finland	1992	1992	14.6	Perinataalitilasto - synnyttäjät, synnytykset ja vastasyntyneet 2010 - Perinatal statistics: parturients, deliveries and newborns 2010. Statistical report 27/2011. Helsinki: Terveyden ja Hyvinvoinnin Laitos/National Institute for Health and Welfare (THL), Finland; 2011.
FIN	Finland	1993	1993	14.7	Synnytykset ja vastasyntyneet 2009 - Births and Newborns 2009. Statistical report 26/2010. Helsinki: Terveyden ja Hyvinvoinnin Laitos/National Institute for Health and Welfare (THL), Finland; 2010.
FIN	Finland	1994	1994	15.6	Synnyttäjät, synnytykset ja vastasyntyneet 2004 - Parturients, births and newborns 2004. Statistical Summary 21/2005. Helsinki: National Research and Development Centre for Welfare and Health; 2005.
FIN	Finland	1995	1995	15.8	Synnytykset ja vastasyntyneet 2009 - Births and Newborns 2009. Statistical report 26/2010. Helsinki: Terveyden ja Hyvinvoinnin Laitos/National Institute for Health and Welfare (THL), Finland; 2010.
FIN	Finland	1996	1996	15.9	Perinataalitilasto - synnyttäjät, synnytykset ja vastasyntyneet 2010 - Perinatal statistics: parturients, deliveries and newborns 2010. Statistical report 27/2011. Helsinki: Terveyden ja Hyvinvoinnin Laitos/National Institute for Health and Welfare (THL), Finland; 2011.

ISO Code	Country	Coverage start year	Coverage end year	Caesarean section rate (%)	References
FIN	Finland	1997	1997	15.8	Synnytykset ja vastasyntyneet 2009 - Births and Newborns 2009. Statistical report 26/2010. Helsinki: Terveyden ja Hyvinvoinnin Laitos/National Institute for Health and Welfare (THL), Finland; 2010.
FIN	Finland	1998	1998	15.6	Perinataalitilasto - synnyttäjät, synnytykset ja vastasyntyneet 2010 - Perinatal statistics: parturients, deliveries and newborns 2010. Statistical report 27/2011. Helsinki: Terveyden ja Hyvinvoinnin Laitos/National Institute for Health and Welfare (THL), Finland; 2011.
FIN	Finland	1999	1999	16.0	Synnytykset ja vastasyntyneet 2009 - Births and Newborns 2009. Statistical report 26/2010. Helsinki: Terveyden ja Hyvinvoinnin Laitos/National Institute for Health and Welfare (THL), Finland; 2010.
FIN	Finland	2000	2000	16.0	Perinataalitilasto - synnyttäjät, synnytykset ja vastasyntyneet 2010 - Perinatal statistics: parturients, deliveries and newborns 2010. Statistical report 27/2011. Helsinki: Terveyden ja Hyvinvoinnin Laitos/National Institute for Health and Welfare (THL), Finland; 2011.
FIN	Finland	2001	2001	16.7	Synnytykset ja vastasyntyneet 2009 - Births and Newborns 2009. Statistical report 26/2010. Helsinki: Terveyden ja Hyvinvoinnin Laitos/National Institute for Health and Welfare (THL), Finland; 2010.
FIN	Finland	2002	2002	16.6	Perinataalitilasto - synnyttäjät, synnytykset ja vastasyntyneet 2010 - Perinatal statistics: parturients, deliveries and newborns 2010. Statistical report 27/2011. Helsinki: Terveyden ja Hyvinvoinnin Laitos/National Institute for Health and Welfare (THL), Finland; 2011.
FIN	Finland	2003	2003	16.4	Synnytykset ja vastasyntyneet 2009 - Births and Newborns 2009. Statistical report 26/2010. Helsinki: Terveyden ja Hyvinvoinnin Laitos/National Institute for Health and Welfare (THL), Finland; 2010.
FIN	Finland	2004	2004	16.6	Perinataalitilasto - synnyttäjät, synnytykset ja vastasyntyneet 2010 - Perinatal statistics: parturients, deliveries and newborns 2010. Statistical report 27/2011. Helsinki: Terveyden ja Hyvinvoinnin Laitos/National Institute for Health and Welfare (THL), Finland; 2011.

ISO Code	Country	Coverage start year	Coverage end year	Caesarean section rate (%)	References
FIN	Finland	2006	2006	16.3	Perinataalitilasto - synnyttäjät, synnytykset ja vastasyntyneet 2010 - Perinatal statistics: parturients, deliveries and newborns 2010. Statistical report 27/2011. Helsinki: Terveyden ja Hyvinvoinnin Laitos/National Institute for Health and Welfare (THL), Finland; 2011.
FIN	Finland	2007	2007	16.5	Perinataalitilasto - synnyttäjät, synnytykset ja vastasyntyneet 2010 - Perinatal statistics: parturients, deliveries and newborns 2010. Statistical report 27/2011. Helsinki: Terveyden ja Hyvinvoinnin Laitos/National Institute for Health and Welfare (THL), Finland; 2011.
FIN	Finland	2008	2008	16.7	Perinataalitilasto - synnyttäjät, synnytykset ja vastasyntyneet 2010 - Perinatal statistics: parturients, deliveries and newborns 2010. Statistical report 27/2011. Helsinki: Terveyden ja Hyvinvoinnin Laitos/National Institute for Health and Welfare (THL), Finland; 2011.
FIN	Finland	2009	2009	15.9	Perinataalitilasto - synnyttäjät, synnytykset ja vastasyntyneet 2010 - Perinatal statistics: parturients, deliveries and newborns 2010. Statistical report 27/2011. Helsinki: Terveyden ja Hyvinvoinnin Laitos/National Institute for Health and Welfare (THL), Finland; 2011.
FIN	Finland	2010	2010	16.3	Perinataalitilasto - synnyttäjät, synnytykset ja vastasyntyneet 2010 - Perinatal statistics: parturients, deliveries and newborns 2010. Statistical report 27/2011. Helsinki: Terveyden ja Hyvinvoinnin Laitos/National Institute for Health and Welfare (THL), Finland; 2011.
FIN	Finland	2011	2011	16.3	Perinataalitilasto - synnyttäjät, synnytykset ja vastasyntyneet 2011 - Perinatal statistics: parturients, deliveries and newborns 2011. Statistical report 20/2012. Helsinki: Terveyden ja Hyvinvoinnin Laitos/National Institute for Health and Welfare (THL), Finland; 2012.
FIN	Finland	2012	2012	16.3	Perinataalitilasto - synnyttäjät, synnytykset ja vastasyntyneet 2012 - Perinatal statistics: parturients, deliveries and newborns 2012. Statistical report 24/2013. Helsinki: Terveyden ja Hyvinvoinnin Laitos/National Institute for Health and Welfare (THL), Finland; 2013.
FIN	Finland	2013	2013	16.3	Perinataalitilasto - synnyttäjät, synnytykset ja vastasyntyneet 2013 - Perinatal statistics: parturients, deliveries and newborns 2013. Statistical report 23/2014. Helsinki: Terveyden ja Hyvinvoinnin Laitos/National Institute for Health and Welfare (THL), Finland; 2014.

ISO Code	Country	Coverage start year	Coverage end year	Caesarean section rate (%)	References
FIN	Finland	2014	2014	16.1	Perinataalitilasto - synnyttäjät, synnytykset ja vastasyntyneet 2014 - Perinatal statistics: parturients, deliveries and newborns 2014. Statistical report 19/2015. Helsinki: Terveyden ja Hyvinvoinnin Laitos/National Institute for Health and Welfare (THL), Finland; 2015.
FIN	Finland	2005	2005	15.9	Perinataalitilasto - synnyttäjät, synnytykset ja vastasyntyneet 2015 - Perinatal statistics: parturients, deliveries and newborns 2015. Statistical report 16/2016. Helsinki: Terveyden ja Hyvinvoinnin Laitos/National Institute for Health and Welfare (THL), Finland; 2016.
FIN	Finland	2016	2016	16.4	Perinataalitilasto - synnyttäjät, synnytykset ja vastasyntyneet 2016 [Perinatal statistics: parturients, deliveries and newborns 2016]. [Statistical report] 37/2017. Helsinki: Terveyden ja Hyvinvoinnin Laitos/National Institute for Health and Welfare (THL), Finland; 2017 (in Finnish).
FIN	Finland	2015	2015	16.0	Perinataalitilasto - synnyttäjät, synnytykset ja vastasyntyneet 2017 [Perinatal statistics: parturients, deliveries and newborns 2017]. Tilastoraportti [Statistical report] 38/2018. Helsinki: Terveyden ja Hyvinvoinnin Laitos/National Institute for Health and Welfare (THL) [Finland]; 2018.
FIN	Finland	2017	2017	16.7	Perinataalitilasto - synnyttäjät, synnytykset ja vastasyntyneet 2017 [Perinatal statistics: parturients, deliveries and newborns 2017]. Tilastoraportti [Statistical report] 38/2018. Helsinki: Terveyden ja Hyvinvoinnin Laitos/National Institute for Health and Welfare (THL) [Finland]; 2018.
FIN	Finland	2018	2018	16.7	Perinataalitilasto - synnyttäjät, synnytykset ja vastasyntyneet 2018 [Perinatal statistics: parturients, deliveries and newborns 2018]. Tilastoraportti [Statistical report] 49/2019. Helsinki: Terveyden ja Hyvinvoinnin Laitos/National Institute for Health and Welfare (THL) [Finland]; 2019.
FRA	France	1990	1990	13.9	European Health for All Database (HFA-DB) [online database]. World Health Organization (WHO) Regional Office for Europe; 2012 (http://data.euro.who.int/hfadb, accessed 20 August 2012).
FRA	France	1991	1991	12.7	European Health for All Database (HFA-DB) [online database]. World Health Organization (WHO) Regional Office for Europe; 2012 (http://data.euro.who.int/hfadb, accessed 20 August 2012).

ISO Code	Country	Coverage start year	Coverage end year	Caesarean section rate (%)	References
FRA	France	1992	1992	14.5	European Health for All Database (HFA-DB) [online database]. World Health Organization (WHO) Regional Office for Europe; 2012 (http://data.euro.who.int/hfadb, accessed 20 August 2012).
FRA	France	1993	1993	15.5	European Health for All Database (HFA-DB) [online database]. World Health Organization (WHO) Regional Office for Europe; 2012 (http://data.euro.who.int/hfadb, accessed 20 August 2012).
FRA	France	1994	1994	15.1	European Health for All Database (HFA-DB) [online database]. World Health Organization (WHO) Regional Office for Europe; 2012 (http://data.euro.who.int/hfadb, accessed 20 August 2012).
FRA	France	1995	1995	15.0	European Health for All Database (HFA-DB) [online database]. World Health Organization (WHO) Regional Office for Europe; 2012 (http://data.euro.who.int/hfadb, accessed 20 August 2012).
FRA	France	1996	1996	15.4	European Health for All Database (HFA-DB) [online database]. World Health Organization (WHO) Regional Office for Europe; 2012 (http://data.euro.who.int/hfadb, accessed 20 August 2012).
FRA	France	1997	1997	16.0	European Health for All Database (HFA-DB) [online database]. World Health Organization (WHO) Regional Office for Europe; 2012 (http://data.euro.who.int/hfadb, accessed 20 August 2012).
FRA	France	1998	1998	15.7	European Health for All Database (HFA-DB) [online database]. World Health Organization (WHO) Regional Office for Europe; 2012 (http://data.euro.who.int/hfadb, accessed 20 August 2012).
FRA	France	1999	1999	16.1	European Health for All Database (HFA-DB) [online database]. World Health Organization (WHO) Regional Office for Europe; 2012 (http://data.euro.who.int/hfadb, accessed 20 August 2012).

ISO Code	Country	Coverage start year	Coverage end year	Caesarean section rate (%)	References
FRA	France	2000	2000	17.2	European Health for All Database (HFA-DB) [online database]. World Health Organization (WHO) Regional Office for Europe; 2012 (http://data.euro.who.int/hfadb, accessed 20 August 2012).
FRA	France	2001	2001	17.9	European Health for All Database (HFA-DB) [online database]. World Health Organization (WHO) Regional Office for Europe; 2012 (http://data.euro.who.int/hfadb, accessed 20 August 2012).
FRA	France	2002	2002	18.7	European Health for All Database (HFA-DB) [online database]. World Health Organization (WHO) Regional Office for Europe; 2012 (http://data.euro.who.int/hfadb, accessed 20 August 2012).
FRA	France	2003	2003	18.8	European Health for All Database (HFA-DB) [online database]. World Health Organization (WHO) Regional Office for Europe; 2012 (http://data.euro.who.int/hfadb, accessed 20 August 2012).
FRA	France	2004	2004	19.2	Maternité – les accouchements en 2004 [website]. Paris: Institut National de la Statistique et des Études Économiques (INSEE) [National Institute of Statistics and Economic Studies]; 2005 (http://insee.fr/fr/themes/document.asp?reg_id=2&ref_id=14086&page=dossiers, accessed 21 October 2012, in French).
FRA	France	2010	2010	21.0	European Health for All Database (HFA-DB) [online database]. World Health Organization (WHO) Regional Office for Europe; 2016 (http://data.euro.who.int/hfadb, accessed 8 August 2016).
FRA	France	2010	2010	21.0	Blondel B, Kermarrec M, editors (Institut National de la santé et de la recherche médicale/INSERM). Enquête nationale périnatale 2010. Les naissances en 2010 et leur évolution depuis 2003. Paris: INSERM, Direction générale de la sante (DGS), Direction de la recherche, des études, de l'évaluation et des statistiques, Ministère de l'Emploi et de la solidarité (DREES); 2011 (in French).
FRA	France	2011	2011	21.0	European Health for All Database (HFA-DB) [online database]. World Health Organization (WHO) Regional Office for Europe; 2016 (http://data.euro.who.int/hfadb, accessed 8 August 2016).

ISO Code	Country	Coverage start year	Coverage end year	Caesarean section rate (%)	References
FRA	France	2012	2012	20.8	European Health for All Database (HFA-DB) [online database]. World Health Organization (WHO) Regional Office for Europe; 2016 (http://data.euro.who.int/hfadb, accessed 8 August 2016).
FRA	France	2013	2013	20.8	European Health for All Database (HFA-DB) [online database]. World Health Organization (WHO) Regional Office for Europe; 2016 (http://data.euro.who.int/hfadb, accessed 8 August 2016).
FRA	France	2014	2014	20.8	European Health for All Database (HFA-DB) [online database]. World Health Organization (WHO) Regional Office for Europe; 2016 (http://data.euro.who.int/hfadb, accessed 8 August 2016).
FRA	France	2016	2016	19.6	Enquête nationale périnatale Rapport 2016. Les naissances et les établissements. Situation et évaluation depuis 2010. Paris: Institut National de la santé et de la recherche médicale (INSERM), Direction de la recherche, des études, de l’évaluation et des statistiques, Ministère de l'Emploi et de la solidarité (DREES); 2017 (in French).
GAB	Gabon	1995	2000	5.6	Direction Générale de la Statistique et des Études Économiques (DGSEE) [Gabon], ORC Macro. Enquête Démographique et de Santé Gabon 2000. Calverton, Maryland: Direction Générale de la Statistique et des Études Économiques, Fonds des Nations Unies pour la population (FNUAP/UNFPA), ORC Macro; 2001 (in French).
GAB	Gabon	2007	2012	10.0	Direction Générale de la Statistique (DGS), ICF International. Enquête Démographique et de Santé du Gabon 2012. Calverton, Maryland, Libreville, Gabon: DGS, ICF International; 2013 (in French).
GMB	Gambia	2008	2010	2.5	The Gambia Bureau of Statistics (GBOS). The Gambia Multiple Indicator Cluster Survey 2010, Final Report. Banjul, The Gambia: The Gambia Bureau of Statistics (GBOS); 2012.
GMB	Gambia	2008	2013	2.0	The Gambia Bureau of Statistics (GBOS), ICF International. The Gambia Demographic and Health Survey 2013. Banjul, The Gambia, and Rockville, Maryland, USA: GBOS, ICF International; 2014.

ISO Code	Country	Coverage start year	Coverage end year	Caesarean section rate (%)	References
GMB	Gambia	2016	2018	3.7	The Gambia Multiple Indicator Cluster Survey 2018, Survey Findings Report. Banjul: The Gambia Bureau of Statistics (GBOS); 2019.
GEO	Georgia	1990	1990	3.8	European Health for All Database (HFA-DB) [online database]. World Health Organization (WHO) Regional Office for Europe; 2012 (http://data.euro.who.int/hfadb, accessed 20 August 2012).
GEO	Georgia	1991	1991	4.6	European Health for All Database (HFA-DB) [online database]. World Health Organization (WHO) Regional Office for Europe; 2012 (http://data.euro.who.int/hfadb, accessed 20 August 2012).
GEO	Georgia	1992	1992	5.1	European Health for All Database (HFA-DB) [online database]. World Health Organization (WHO) Regional Office for Europe; 2012 (http://data.euro.who.int/hfadb, accessed 20 August 2012).
GEO	Georgia	1993	1993	4.6	European Health for All Database (HFA-DB) [online database]. World Health Organization (WHO) Regional Office for Europe; 2012 (http://data.euro.who.int/hfadb, accessed 20 August 2012).
GEO	Georgia	1994	1994	5.5	European Health for All Database (HFA-DB) [online database]. World Health Organization (WHO) Regional Office for Europe; 2012 (http://data.euro.who.int/hfadb, accessed 20 August 2012).
GEO	Georgia	1995	1995	5.8	European Health for All Database (HFA-DB) [online database]. World Health Organization (WHO) Regional Office for Europe; 2012 (http://data.euro.who.int/hfadb, accessed 20 August 2012).
GEO	Georgia	1996	1996	6.7	European Health for All Database (HFA-DB) [online database]. World Health Organization (WHO) Regional Office for Europe; 2012 (http://data.euro.who.int/hfadb, accessed 20 August 2012).

ISO Code	Country	Coverage start year	Coverage end year	Caesarean section rate (%)	References
GEO	Georgia	1998	1998	7.9	European Health for All Database (HFA-DB) [online database]. World Health Organization (WHO) Regional Office for Europe; 2012 (http://data.euro.who.int/hfadb, accessed 20 August 2012).
GEO	Georgia	1999	1999	7.2	European Health for All Database (HFA-DB) [online database]. World Health Organization (WHO) Regional Office for Europe; 2012 (http://data.euro.who.int/hfadb, accessed 20 August 2012).
GEO	Georgia	1994	2000	6.4	National Center for Disease Control (NCDC) [Georgia], Ministry of Health and Social Affairs (MOH&SA) [Georgia], Center for Medical Statistics and Information (CMSI) [Georgia], State Department of Statistics (SDS) [Georgia], Division of Reproductive Health, Centers for Disease Control and Prevention (DRH/CDC), United Nations Population Fund (UNFPA), United Nations Children's Fund (UNICEF), United States Agency for International Development (USAID), United Nations High Commissioner for Refugees (UNHCR), American International Health Alliance, Inc. (AIHA). Reproductive Health Survey Georgia 1999. Final Report. Atlanta, GA: CDC; 2001.
GEO	Georgia	2000	2000	8.7	European Health for All Database (HFA-DB) [online database]. World Health Organization (WHO) Regional Office for Europe; 2012 (http://data.euro.who.int/hfadb, accessed 20 August 2012).
GEO	Georgia	2001	2001	10.0	European Health for All Database (HFA-DB) [online database]. World Health Organization (WHO) Regional Office for Europe; 2012 (http://data.euro.who.int/hfadb, accessed 20 August 2012).
GEO	Georgia	2004	2004	16.8	Health and Health Care, Georgia, 2005 Statistics. Tbilisi: Ministry of Labour, Health and Social Affairs of Georgia, National Centre for Disease Control and Medical Statistics; 2006.
GEO	Georgia	2000	2005	13.0	National Center for Disease Control (NCDC) [Georgia], Ministry of Health and Social Affairs (MOH&SA) [Georgia], Department of Statistics [Georgia], Division of Reproductive Health, Centers for Disease Control and Prevention (DRH/CDC), United Nations Population Fund (UNFPA), United States Agency for International Development (USAID). Reproductive Health Survey Georgia 2005. Final Report. Atlanta, GA: CDC; 2007.
GEO	Georgia	2005	2005	19.3	Health and Health Care, Georgia, 2005 Statistics. Tbilisi: Ministry of Labour, Health and Social Affairs of Georgia, National Centre for Disease Control and Medical Statistics; 2006.

ISO Code	Country	Coverage start year	Coverage end year	Caesarean section rate (%)	References
GEO	Georgia	2006	2006	20.8	Health and Health Care, Statistical Yearbook 2007 Georgia. Tbilisi: Ministry of Labour, Health and Social Affairs of Georgia, National Centre for Disease Control and Public Health; 2008.
GEO	Georgia	2009	2009	28.7	Health Care, Statistical Yearbook 2010 Georgia. Tbilisi: Ministry of Labour, Health and Social Affairs of Georgia, National Centre for Disease Control and Public Health; 2011.
GEO	Georgia	2005	2010	23.9	Reproductive Health Survey Georgia 2010 Final Report. Tbilisi: National Center for Disease Control and Public Health (NCDC) [Georgia], Centers for Disease Control and Prevention (DRH/CDC), United Nations Population Fund (UNFPA), United States Agency for International Development (USAID), United Nations Children's Fund (UNICEF); 2012.
GEO	Georgia	2010	2010	31.4	Health Care, Statistical Yearbook 2010 Georgia. Tbilisi: Ministry of Labour, Health and Social Affairs of Georgia, National Centre for Disease Control and Public Health; 2011.
GEO	Georgia	2011	2011	35.1	Health Care, Statistical Yearbook 2011 Georgia. Tbilisi: Ministry of Labour, Health and Social Affairs of Georgia, National Centre for Disease Control and Public Health; 2012.
GEO	Georgia	2012	2012	36.7	European Health for All Database (HFA-DB) [online database]. World Health Organization (WHO) Regional Office for Europe; 2016 (http://data.euro.who.int/hfadb, accessed 8 August 2016).
GEO	Georgia	2013	2013	37.3	Health Care, Statistical Yearbook 2013 Georgia. Tbilisi: Ministry of Labour, Health and Social Affairs of Georgia, National Centre for Disease Control and Public Health; 2014.
GEO	Georgia	2014	2014	38.9	Health Care, Statistical Yearbook 2014 Georgia. Tbilisi: Ministry of Labour, Health and Social Affairs of Georgia, National Centre for Disease Control and Public Health; 2015.

ISO Code	Country	Coverage start year	Coverage end year	Caesarean section rate (%)	References
GEO	Georgia	2015	2015	41.4	Health Care, Statistical Yearbook 2015 Georgia. Tbilisi: Ministry of Labour, Health and Social Affairs of Georgia, National Centre for Disease Control and Public Health; 2016.
GEO	Georgia	2016	2016	43.7	Health Care, Statistical Yearbook 2016 Georgia. Tbilisi: Ministry of Labour, Health and Social Affairs of Georgia, National Centre for Disease Control and Public Health; 2017.
GEO	Georgia	2016	2018	46.6	Georgia Multiple Indicator Cluster Survey 2018, Survey Findings Report. Tbilisi: National Statistics Office of Georgia, United Nations Children's Fund (UNICEF); 2019.
DEU	Germany	1991	1991	15.3	Krankenhausentbindungen in Deutschland: Jahre 1991 bis 2016 [website]. Wiesbaden: Statistisches Bundesamt (DESTATIS) [Federal Statistical Office, Germany]; 2017 (https://www.destatis.de/DE/ZahlenFakten/GesellschaftStaat, accessed 9 November 2017, in German).
DEU	Germany	1992	1992	16.2	Krankenhausentbindungen in Deutschland: Jahre 1991 bis 2016 [website]. Wiesbaden: Statistisches Bundesamt (DESTATIS) [Federal Statistical Office, Germany]; 2017 (https://www.destatis.de/DE/ZahlenFakten/GesellschaftStaat, accessed 9 November 2017, in German).
DEU	Germany	1993	1993	16.9	Krankenhausentbindungen in Deutschland: Jahre 1991 bis 2016 [website]. Wiesbaden: Statistisches Bundesamt (DESTATIS) [Federal Statistical Office, Germany]; 2017 (https://www.destatis.de/DE/ZahlenFakten/GesellschaftStaat, accessed 9 November 2017, in German).
DEU	Germany	1994	1994	17.3	Krankenhausentbindungen in Deutschland: Jahre 1991 bis 2016 [website]. Wiesbaden: Statistisches Bundesamt (DESTATIS) [Federal Statistical Office, Germany]; 2017 (https://www.destatis.de/DE/ZahlenFakten/GesellschaftStaat, accessed 9 November 2017, in German).
DEU	Germany	1995	1995	17.6	Krankenhausentbindungen in Deutschland: Jahre 1991 bis 2016 [website]. Wiesbaden: Statistisches Bundesamt (DESTATIS) [Federal Statistical Office, Germany]; 2017 (https://www.destatis.de/DE/ZahlenFakten/GesellschaftStaat, accessed 9 November 2017, in German).

ISO Code	Country	Coverage start year	Coverage end year	Caesarean section rate (%)	References
DEU	Germany	1996	1996	18.0	Krankenhausentbindungen in Deutschland: Jahre 1991 bis 2016 [website]. Wiesbaden: Statistisches Bundesamt (DESTATIS) [Federal Statistical Office, Germany]; 2017 (https://www.destatis.de/DE/ZahlenFakten/GesellschaftStaat, accessed 9 November 2017, in German).
DEU	Germany	1997	1997	18.5	Krankenhausentbindungen in Deutschland: Jahre 1991 bis 2016 [website]. Wiesbaden: Statistisches Bundesamt (DESTATIS) [Federal Statistical Office, Germany]; 2017 (https://www.destatis.de/DE/ZahlenFakten/GesellschaftStaat, accessed 9 November 2017, in German).
DEU	Germany	1998	1998	19.5	Krankenhausentbindungen in Deutschland: Jahre 1991 bis 2016 [website]. Wiesbaden: Statistisches Bundesamt (DESTATIS) [Federal Statistical Office, Germany]; 2017 (https://www.destatis.de/DE/ZahlenFakten/GesellschaftStaat, accessed 9 November 2017, in German).
DEU	Germany	1999	1999	20.3	Krankenhausentbindungen in Deutschland: Jahre 1991 bis 2016 [website]. Wiesbaden: Statistisches Bundesamt (DESTATIS) [Federal Statistical Office, Germany]; 2017 (https://www.destatis.de/DE/ZahlenFakten/GesellschaftStaat, accessed 9 November 2017, in German).
DEU	Germany	2000	2000	21.5	Krankenhausentbindungen in Deutschland: Jahre 1991 bis 2016 [website]. Wiesbaden: Statistisches Bundesamt (DESTATIS) [Federal Statistical Office, Germany]; 2017 (https://www.destatis.de/DE/ZahlenFakten/GesellschaftStaat, accessed 9 November 2017, in German).
DEU	Germany	2001	2001	22.6	Krankenhausentbindungen in Deutschland: Jahre 1991 bis 2016 [website]. Wiesbaden: Statistisches Bundesamt (DESTATIS) [Federal Statistical Office, Germany]; 2017 (https://www.destatis.de/DE/ZahlenFakten/GesellschaftStaat, accessed 9 November 2017, in German).
DEU	Germany	2002	2002	24.4	Krankenhausentbindungen in Deutschland: Jahre 1991 bis 2016 [website]. Wiesbaden: Statistisches Bundesamt (DESTATIS) [Federal Statistical Office, Germany]; 2017 (https://www.destatis.de/DE/ZahlenFakten/GesellschaftStaat, accessed 9 November 2017, in German).
DEU	Germany	2003	2003	25.5	Krankenhausentbindungen in Deutschland: Jahre 1991 bis 2016 [website]. Wiesbaden: Statistisches Bundesamt (DESTATIS) [Federal Statistical Office, Germany]; 2017 (https://www.destatis.de/DE/ZahlenFakten/GesellschaftStaat, accessed 9 November 2017, in German).

ISO Code	Country	Coverage start year	Coverage end year	Caesarean section rate (%)	References
DEU	Germany	2004	2004	26.8	Krankenhausentbindungen in Deutschland: Jahre 1991 bis 2016 [website]. Wiesbaden: Statistisches Bundesamt (DESTATIS) [Federal Statistical Office, Germany]; 2017 (https://www.destatis.de/DE/ZahlenFakten/GesellschaftStaat, accessed 9 November 2017, in German).
DEU	Germany	2005	2005	27.6	Krankenhausentbindungen in Deutschland: Jahre 1991 bis 2016 [website]. Wiesbaden: Statistisches Bundesamt (DESTATIS) [Federal Statistical Office, Germany]; 2017 (https://www.destatis.de/DE/ZahlenFakten/GesellschaftStaat, accessed 9 November 2017, in German).
DEU	Germany	2006	2006	28.6	Krankenhausentbindungen in Deutschland: Jahre 1991 bis 2016 [website]. Wiesbaden: Statistisches Bundesamt (DESTATIS) [Federal Statistical Office, Germany]; 2017 (https://www.destatis.de/DE/ZahlenFakten/GesellschaftStaat, accessed 9 November 2017, in German).
DEU	Germany	2007	2007	29.3	Krankenhausentbindungen in Deutschland: Jahre 1991 bis 2016 [website]. Wiesbaden: Statistisches Bundesamt (DESTATIS) [Federal Statistical Office, Germany]; 2017 (https://www.destatis.de/DE/ZahlenFakten/GesellschaftStaat, accessed 9 November 2017, in German).
DEU	Germany	2008	2008	30.2	Krankenhausentbindungen in Deutschland: Jahre 1991 bis 2016 [website]. Wiesbaden: Statistisches Bundesamt (DESTATIS) [Federal Statistical Office, Germany]; 2017 (https://www.destatis.de/DE/ZahlenFakten/GesellschaftStaat, accessed 9 November 2017, in German).
DEU	Germany	2009	2009	31.3	Krankenhausentbindungen in Deutschland: Jahre 1991 bis 2016 [website]. Wiesbaden: Statistisches Bundesamt (DESTATIS) [Federal Statistical Office, Germany]; 2017 (https://www.destatis.de/DE/ZahlenFakten/GesellschaftStaat, accessed 9 November 2017, in German).
DEU	Germany	2010	2010	31.9	Krankenhausentbindungen in Deutschland: Jahre 1991 bis 2016 [website]. Wiesbaden: Statistisches Bundesamt (DESTATIS) [Federal Statistical Office, Germany]; 2017 (https://www.destatis.de/DE/ZahlenFakten/GesellschaftStaat, accessed 9 November 2017, in German).
DEU	Germany	2011	2011	32.2	Krankenhausentbindungen in Deutschland: Jahre 1991 bis 2016 [website]. Wiesbaden: Statistisches Bundesamt (DESTATIS) [Federal Statistical Office, Germany]; 2017 (https://www.destatis.de/DE/ZahlenFakten/GesellschaftStaat, accessed 9 November 2017, in German).

ISO Code	Country	Coverage start year	Coverage end year	Caesarean section rate (%)	References
DEU	Germany	2012	2012	31.9	Krankenhausentbindungen in Deutschland: Jahre 1991 bis 2016 [website]. Wiesbaden: Statistisches Bundesamt (DESTATIS) [Federal Statistical Office, Germany]; 2017 (https://www.destatis.de/DE/ZahlenFakten/GesellschaftStaat, accessed 9 November 2017, in German).
DEU	Germany	2013	2013	31.8	Krankenhausentbindungen in Deutschland: Jahre 1991 bis 2016 [website]. Wiesbaden: Statistisches Bundesamt (DESTATIS) [Federal Statistical Office, Germany]; 2017 (https://www.destatis.de/DE/ZahlenFakten/GesellschaftStaat, accessed 9 November 2017, in German).
DEU	Germany	2014	2014	31.8	Krankenhausentbindungen in Deutschland: Jahre 1991 bis 2016 [website]. Wiesbaden: Statistisches Bundesamt (DESTATIS) [Federal Statistical Office, Germany]; 2017 (https://www.destatis.de/DE/ZahlenFakten/GesellschaftStaat, accessed 9 November 2017, in German).
DEU	Germany	2015	2015	31.1	Krankenhausentbindungen in Deutschland: Jahre 1991 bis 2016 [website]. Wiesbaden: Statistisches Bundesamt (DESTATIS) [Federal Statistical Office, Germany]; 2017 (https://www.destatis.de/DE/ZahlenFakten/GesellschaftStaat, accessed 9 November 2017, in German).
DEU	Germany	2016	2016	30.5	Krankenhausentbindungen in Deutschland: Jahre 1991 bis 2016 [website]. Wiesbaden: Statistisches Bundesamt (DESTATIS) [Federal Statistical Office, Germany]; 2017 (https://www.destatis.de/DE/ZahlenFakten/GesellschaftStaat, accessed 9 November 2017, in German).
DEU	Germany	2017	2017	30.5	Hospital deliveries in Germany from 1991 to 2017 [website]. Wiesbaden: Statistisches Bundesamt (DESTATIS) [Federal Statistical Office, Germany]; 2019 (https://www.destatis.de/EN/FactsFigures/SocietyState/Health/Hospitals/Tables/HospitalsDeliveriesYears.html, accessed 13 February 2019).
GHA	Ghana	1990	1993	4.4	Ghana Statistical Service (GSS), Macro International Inc. (MI). Ghana Demographic and Health Survey 1993. Calverton, Maryland: GSS, MI; 1994.
GHA	Ghana	1993	1998	4.0	Ghana Statistical Service (GSS), Macro International Inc. (MI). Ghana Demographic and Health Survey 1998. Calverton, Maryland: GSS, MI; 1999.

ISO Code	Country	Coverage start year	Coverage end year	Caesarean section rate (%)	References
GHA	Ghana	1998	2003	3.7	Ghana Statistical Service (GSS), Noguchi Memorial Institute for Medical Research (NMIMR), ORC Macro. Ghana Demographic and Health Survey 2003. Calverton, Maryland: GSS, NMIMR, ORC Macro; 2004.
GHA	Ghana	2002	2007	6.5	Ghana Statistical Service (GSS), Ghana Health Service (GHS), Macro International. Ghana Maternal Health Survey 2007. Calverton, Maryland, USA: GSS, GHS, Macro International; 2009.
GHA	Ghana	2003	2008	6.9	Ghana Statistical Service (GSS), Ghana Health Service (GHS), ICF Macro. Ghana Demographic and Health Survey 2008. Accra, Ghana: GSS, GHS, ICF Macro; 2009.
GHA	Ghana	2009	2011	11.4	Ghana Statistical Service, United Nations Children's Fund (UNICEF). Ghana Multiple Indicator Cluster Survey with an Enhanced Malaria Module and Biomarker, 2011, Final Report. Accra, Ghana: Ghana Statistical Service, United Nations Children's Fund (UNICEF); 2011.
GHA	Ghana	2009	2014	12.8	Ghana Statistical Service (GSS), Ghana Health Service (GHS), ICF International. Ghana Demographic and Health Survey 2014. Rockville, Maryland, USA: GSS, GHS, ICF International; 2015.
GHA	Ghana	2012	2017	16.0	Ghana Maternal Health Survey 2017. Accra, Ghana: Ghana Statistical Service (GSS), Ghana Health Service (GHS), ICF; 2018.
GTM	Guatemala	1990	1995	8.2	Instituto Nacional de Estadística (INE), Macro International Inc. Guatemala Encuesta Nacional de Salud Materno Infantil 1995. Calverton, Maryland, USA: Macro International Inc.; 1996 (in Spanish).
GTM	Guatemala	1993	1999	10.8	Instituto Nacional de Estadística (INE), Macro International Inc. Guatemala Encuesta Nacional de Salud Materno Infantil 1998-1999. Calverton, Maryland, USA: Macro International Inc.; 1999 (in Spanish).

ISO Code	Country	Coverage start year	Coverage end year	Caesarean section rate (%)	References
GTM	Guatemala	1997	2002	11.4	Encuesta Nacional de Salud Materno Infantil (ENSMI) 2002. Ciudad de Guatemala: Ministerio de Salud Pública y Asistencia Social (MSPAS) [Guatemala], Instituto Nacional de Estadística (INE) [Guatemala], Centros de Control y Prevención de Enfermedades (CDC); 2003 (in Spanish).
GTM	Guatemala	2003	2009	16.3	Encuesta Nacional de Salud Materno Infantil (ENSMI) 2008-2009. Ciudad de Guatemala: Ministerio de Salud Pública y Asistencia Social (MSPAS) [Guatemala], Instituto Nacional de Estadística (INE) [Guatemala], Centros de Control y Prevención de Enfermedades (CDC); 2010 (in Spanish).
GTM	Guatemala	2009	2015	26.3	Encuesta Nacional de Salud Materno Infantil (ENSMI) 2014-2015. Informe Final. Ciudad de Guatemala: Ministerio de Salud Pública y Asistencia Social (MSPAS) [Guatemala], Instituto Nacional de Estadística (INE) [Guatemala], ICF International; 2015 (in Spanish).
GIN	Guinea	1994	1999	2.0	Direction Nationale de la Statistique [Guinée], Macro International Inc. Enquête Démographique et de Santé, Guinée 1999. Calverton, Maryland USA: Direction Nationale de la Statistique, Macro International Inc.; 2000 (in French).
GIN	Guinea	2000	2005	1.7	Direction Nationale de la Statistique (DNS) [Guinée], ORC Macro. Enquête Démographique et de Santé, Guinée 2005. Calverton, Maryland, USA: DNS, ORC Macro; 2006 (in French).
GIN	Guinea	2002	2007	2.4	Enquête nationale sur l'état nutritionnel et le suivi des principaux indicateurs de survie de l'enfant. Rapport provisoire. Guinée : Ministère de l'Economie des Finances et du Plan, Direction Nationale de la Statistique [Guinée], Fonds des Nations Unies pour l'enfance (UNICEF), PAM; 2008 (in French).
GIN	Guinea	2007	2012	2.4	Guinée. Enquête Démographique et de Santé et à Indicateurs Multiples (EDS-MICS 2012). Calverton, Maryland: Institut National de la Statistique [Guinée], MEASURE DHS, ICF international; 2013 (in French).
GIN	Guinea	2014	2016	3.1	Institut National de la Statistique [Guinée], Fonds des Nations Unies pour l'Enfance (UNICEF), USAID, le Fonds mondial [the Global Fund], Fonds des Nations Unies pour la Population (FNUAP/UNFPA), Programme des Nations Unies pour le développement (PNUD/UNDP). Enquête par grappes à indicateurs multiples (MICS 2016), Rapport final. Conakry: Institut National de la Statistique [Guinée]; 2017 (in French).

ISO Code	Country	Coverage start year	Coverage end year	Caesarean section rate (%)	References
GIN	Guinea	2013	2018	2.7	Enquête Démographique et de Santé en Guinée 2018. Conakry, Rockville, Maryland: Institut National de la Statistique (INS), ICF; 2018 (in French).
GNB	Guinea-Bissau	2008	2010	2.3	Ministério da Economia, do Plano e Integração Regional – Direcção Geral do Plano, Fundo das Nações Unidas para a Infância (UNICEF). Inquérito aos Indicadores Múltiplos, Inquérito Demográfico de Saúde Reprodutiva 2010. Relatório Final. Guiné-Bissau: Ministério da Economia, do Plano e Integração Regional – Direcção Geral do Plano; 2011 (in Portuguese).
GNB	Guinea-Bissau	2012	2014	3.9	Ministério da Economia e Finanças, Direcção Geral do Plano Instituto Nacional de Estatística (INE), Fundo das Nações Unidas para a Infância (UNICEF). Inquérito aos Indicadores Múltiplos (MICS) 2014, Principais Resultados. Guiné-Bissau: Ministério da Economia e Finanças, Direcção Geral do Plano Instituto Nacional de Estatística (INE); 2015 (in Portuguese).
GUY	Guyana	2004	2009	13.3	Ministry of Health (MOH), Bureau of Statistics (BOS), ICF Macro. Guyana Demographic and Health Survey 2009. Georgetown, Guyana: MOH, BOS, ICF Macro; 2010.
GUY	Guyana	2012	2014	16.9	Bureau of Statistics, Ministry of Health, United Nations Children's Fund (UNICEF). Guyana Multiple Indicator Cluster Survey 2014. Georgetown, Guyana: Bureau of Statistics, Ministry of Health, UNICEF; 2015.
HTI	Haiti	1989	1995	1.6	Cayemittes M, Rival A, Barrère B, Lerebours G, Amédée Gédéon M (Institut Hattien de l'Enfance, Macro International Inc.). Enquête Mortalité, Morbidité et Utilisation des Services (EMMUS-II), Haïti 1994/95. Calverton, Maryland USA: Institut Hattien de l'Enfance, Macro International Inc.; 1995 (in French).
HTI	Haiti	1995	2000	1.6	Cayemittes M, Placide MF, Barrère B, Mariko S, Sévère B (Ministère de la Santé Publique et de la Population, Institut Haïtien de l'Enfance, ORC Macro). Enquête Mortalité, Morbidité et Utilisation des Services, Haïti 2000. Calverton, Maryland, USA: Ministère de la Santé Publique et de la Population, Institut Haïtien de l'Enfance, ORC Macro; 2001 (in French).
HTI	Haiti	2000	2006	3.0	Cayemittes M, Placide MF, Mariko S, Barrère B, Sévère B, Alexandre C (Ministère de la Santé Publique et de la Population, Institut Haïtien de l'Enfance, Macro International Inc.). Enquête Mortalité, Morbidité et Utilisation des Services, Haïti, 2005-2006. Calverton, Maryland, USA: Ministère de la Santé Publique et de la Population, Institut Haïtien de l'Enfance, Macro International Inc.; 2007 (in French).

ISO Code	Country	Coverage start year	Coverage end year	Caesarean section rate (%)	References
HTI	Haiti	2007	2012	5.5	Cayemittes M, Busangu MF, de Dieu Bizimana J, Barrère B, Sévère B, Cayemittes V, Charles E (MSPP, IHE, ICF International). Enquête Mortalité, Morbidité et Utilisation des Services, Haïti, 2012. Calverton, Maryland, USA : MSPP, IHE, ICF International; 2013 (in French).
HTI	Haiti	2013	2013	5.4	Rapport Statistique 2013. République d'Haïti, Ministère de la Santé Publique et de la Population, Unité d'Études et de Programmation; 2014 (in French).
HTI	Haiti	2011	2012	5.4	Enquête Mortalité, Morbidité et Utilisation des Services (EMMUS-VI 2016-2017). Pétion-Ville, Haïti, Rockville, Maryland, USA: Institut Haïtien de l'Enfance (IHE), ICF; 2018 (in French).
HND	Honduras	1987	1992	6.4	Encuesta Nacional de Epidemiología y Salud Familiar 1991/92 (ENESF-91/92). Honduras: Ministerio de Salud Pública, Agencia de los Estados Unidos para el Desarrollo Internacional (USAID); 1993 (in Spanish).
HND	Honduras	1991	1996	6.3	Encuesta Nacional de Epidemiología y Salud Familiar 1996 (ENESF-96). Informe Final. Honduras: Secretaría de Salud de Honduras (SS), la Asociación Hondureña de Planificación de Familia (ASHONPLAFA), División de Salud Reproductiva de los Centros para el Control y Prevención de Enfermedades (CDC), Agencia de los Estados Unidos para el Desarrollo Internacional (USAID); 1997 (in Spanish).
HND	Honduras	1996	2001	7.9	Encuesta Nacional de Epidemiología y Salud Familiar (ENESF-2001) y de la Encuesta Nacional de Salud Masculina (ENSM-2001). Informe Final. Honduras: Secretaría de Salud de Honduras (SS), la Asociación Hondureña de Planificación de Familia (ASHONPLAFA), Agencia de los Estados Unidos para el Desarrollo Internacional (USAID); 2002 (in Spanish).
HND	Honduras	2000	2006	13.0	Secretaría de Salud [Honduras], Instituto Nacional de Estadística (INE), Macro International. Encuesta Nacional de Demografía y Salud 2005-2006 (ENDESA). Tegucigalpa, Honduras: SS, INE, Macro International; 2006 (in Spanish).
HND	Honduras	2006	2012	18.6	Secretaría de Salud [Honduras], Instituto Nacional de Estadística (INE), ICF International. ENDESA. Encuesta Nacional de Demografía y Salud 2011-2012. Tegucigalpa, Honduras: SS, INE, ICF International; 2013 (in Spanish).

ISO Code	Country	Coverage start year	Coverage end year	Caesarean section rate (%)	References
HUN	Hungary	1994	1994	12.6	European Health for All Database (HFA-DB) [online database]. World Health Organization (WHO) Regional Office for Europe; 2012 (http://data.euro.who.int/hfadb, accessed 20 August 2012).
HUN	Hungary	1995	1995	13.6	European Health for All Database (HFA-DB) [online database]. World Health Organization (WHO) Regional Office for Europe; 2012 (http://data.euro.who.int/hfadb, accessed 20 August 2012).
HUN	Hungary	1996	1996	14.6	European Health for All Database (HFA-DB) [online database]. World Health Organization (WHO) Regional Office for Europe; 2012 (http://data.euro.who.int/hfadb, accessed 20 August 2012).
HUN	Hungary	1997	1997	15.7	European Health for All Database (HFA-DB) [online database]. World Health Organization (WHO) Regional Office for Europe; 2012 (http://data.euro.who.int/hfadb, accessed 20 August 2012).
HUN	Hungary	1998	1998	17.0	European Health for All Database (HFA-DB) [online database]. World Health Organization (WHO) Regional Office for Europe; 2012 (http://data.euro.who.int/hfadb, accessed 20 August 2012).
HUN	Hungary	1999	1999	18.7	European Health for All Database (HFA-DB) [online database]. World Health Organization (WHO) Regional Office for Europe; 2012 (http://data.euro.who.int/hfadb, accessed 20 August 2012).
HUN	Hungary	2000	2000	20.1	European Health for All Database (HFA-DB) [online database]. World Health Organization (WHO) Regional Office for Europe; 2012 (http://data.euro.who.int/hfadb, accessed 20 August 2012).
HUN	Hungary	2001	2001	21.2	European Health for All Database (HFA-DB) [online database]. World Health Organization (WHO) Regional Office for Europe; 2012 (http://data.euro.who.int/hfadb, accessed 20 August 2012).

ISO Code	Country	Coverage start year	Coverage end year	Caesarean section rate (%)	References
HUN	Hungary	2002	2002	23.2	European Health for All Database (HFA-DB) [online database]. World Health Organization (WHO) Regional Office for Europe; 2012 (http://data.euro.who.int/hfadb, accessed 20 August 2012).
HUN	Hungary	2003	2003	24.7	European Health for All Database (HFA-DB) [online database]. World Health Organization (WHO) Regional Office for Europe; 2012 (http://data.euro.who.int/hfadb, accessed 20 August 2012).
HUN	Hungary	2004	2004	25.7	European Health for All Database (HFA-DB) [online database]. World Health Organization (WHO) Regional Office for Europe; 2012 (http://data.euro.who.int/hfadb, accessed 20 August 2012).
HUN	Hungary	2005	2005	27.9	European Health for All Database (HFA-DB) [online database]. World Health Organization (WHO) Regional Office for Europe; 2012 (http://data.euro.who.int/hfadb, accessed 20 August 2012).
HUN	Hungary	2006	2006	28.1	European Health for All Database (HFA-DB) [online database]. World Health Organization (WHO) Regional Office for Europe; 2012 (http://data.euro.who.int/hfadb, accessed 20 August 2012).
HUN	Hungary	2007	2007	28.6	European Health for All Database (HFA-DB) [online database]. World Health Organization (WHO) Regional Office for Europe; 2012 (http://data.euro.who.int/hfadb, accessed 20 August 2012).
HUN	Hungary	2008	2008	29.9	European Health for All Database (HFA-DB) [online database]. World Health Organization (WHO) Regional Office for Europe; 2012 (http://data.euro.who.int/hfadb, accessed 20 August 2012).
HUN	Hungary	2009	2009	31.3	European Health for All Database (HFA-DB) [online database]. World Health Organization (WHO) Regional Office for Europe; 2012 (http://data.euro.who.int/hfadb, accessed 20 August 2012).

ISO Code	Country	Coverage start year	Coverage end year	Caesarean section rate (%)	References
HUN	Hungary	2010	2010	32.5	European Health for All Database (HFA-DB) [online database]. World Health Organization (WHO) Regional Office for Europe; 2016 (http://data.euro.who.int/hfadb, accessed 8 August 2016).
HUN	Hungary	2011	2011	33.2	European Health for All Database (HFA-DB) [online database]. World Health Organization (WHO) Regional Office for Europe; 2016 (http://data.euro.who.int/hfadb, accessed 8 August 2016).
HUN	Hungary	2012	2012	34.0	European Health for All Database (HFA-DB) [online database]. World Health Organization (WHO) Regional Office for Europe; 2016 (http://data.euro.who.int/hfadb, accessed 8 August 2016).
HUN	Hungary	2013	2013	35.3	European Health for All Database (HFA-DB) [online database]. World Health Organization (WHO) Regional Office for Europe; 2016 (http://data.euro.who.int/hfadb, accessed 8 August 2016).
HUN	Hungary	2014	2014	36.4	European Health for All Database (HFA-DB) [online database]. World Health Organization (WHO) Regional Office for Europe; 2016 (http://data.euro.who.int/hfadb, accessed 8 August 2016).
HUN	Hungary	2015	2015	39.0	European Perinatal Health Report. Core indicators of the health and care of pregnant women and babies in Europe in 2015. Euro-Peristat Project; 2018.
ISL	Iceland	1990	1990	11.9	Pohjoismaiset perinataalitilastot 2008 - Perinatal statistics in the Nordic countries 2008. Statistical Report 14/2010. Helsinki: Terveyden ja Hyvinvoinnin Laitos/National Institute for Health and Welfare (THL) [Finland]; 2010.
ISL	Iceland	1991	1991	11.6	Pohjoismaiset perinataalitilastot 2008 - Perinatal statistics in the Nordic countries 2008. Statistical Report 14/2010. Helsinki: Terveyden ja Hyvinvoinnin Laitos/National Institute for Health and Welfare (THL) [Finland]; 2010.

ISO Code	Country	Coverage start year	Coverage end year	Caesarean section rate (%)	References
ISL	Iceland	1992	1992	13.6	Pohjoismaiset perinataalitilastot 2008 - Perinatal statistics in the Nordic countries 2008. Statistical Report 14/2010. Helsinki: Terveyden ja Hyvinvoinnin Laitos/National Institute for Health and Welfare (THL) [Finland]; 2010.
ISL	Iceland	1993	1993	13.2	Pohjoismaiset perinataalitilastot 2008 - Perinatal statistics in the Nordic countries 2008. Statistical Report 14/2010. Helsinki: Terveyden ja Hyvinvoinnin Laitos/National Institute for Health and Welfare (THL) [Finland]; 2010.
ISL	Iceland	1994	1994	14.0	Pohjoismaiset perinataalitilastot 2008 - Perinatal statistics in the Nordic countries 2008. Statistical Report 14/2010. Helsinki: Terveyden ja Hyvinvoinnin Laitos/National Institute for Health and Welfare (THL) [Finland]; 2010.
ISL	Iceland	1995	1995	14.4	Pohjoismaiset perinataalitilastot 2008 - Perinatal statistics in the Nordic countries 2008. Statistical Report 14/2010. Helsinki: Terveyden ja Hyvinvoinnin Laitos/National Institute for Health and Welfare (THL) [Finland]; 2010.
ISL	Iceland	1996	1996	15.3	Pohjoismaiset perinataalitilastot 2008 - Perinatal statistics in the Nordic countries 2008. Statistical Report 14/2010. Helsinki: Terveyden ja Hyvinvoinnin Laitos/National Institute for Health and Welfare (THL) [Finland]; 2010.
ISL	Iceland	1997	1997	16.6	Pohjoismaiset perinataalitilastot 2008 - Perinatal statistics in the Nordic countries 2008. Statistical Report 14/2010. Helsinki: Terveyden ja Hyvinvoinnin Laitos/National Institute for Health and Welfare (THL) [Finland]; 2010.
ISL	Iceland	1998	1998	16.2	Pohjoismaiset perinataalitilastot 2008 - Perinatal statistics in the Nordic countries 2008. Statistical Report 14/2010. Helsinki: Terveyden ja Hyvinvoinnin Laitos/National Institute for Health and Welfare (THL) [Finland]; 2010.
ISL	Iceland	1999	1999	17.6	Pohjoismaiset perinataalitilastot 2008 - Perinatal statistics in the Nordic countries 2008. Statistical Report 14/2010. Helsinki: Terveyden ja Hyvinvoinnin Laitos/National Institute for Health and Welfare (THL) [Finland]; 2010.

ISO Code	Country	Coverage start year	Coverage end year	Caesarean section rate (%)	References
ISL	Iceland	2000	2000	17.9	Pohjoismaiset perinataalitilastot 2008 - Perinatal statistics in the Nordic countries 2008. Statistical Report 14/2010. Helsinki: Terveyden ja Hyvinvoinnin Laitos/National Institute for Health and Welfare (THL) [Finland]; 2010.
ISL	Iceland	2001	2001	17.0	Pohjoismaiset perinataalitilastot 2008 - Perinatal statistics in the Nordic countries 2008. Statistical Report 14/2010. Helsinki: Terveyden ja Hyvinvoinnin Laitos/National Institute for Health and Welfare (THL) [Finland]; 2010.
ISL	Iceland	2002	2002	17.7	Pohjoismaiset perinataalitilastot 2008 - Perinatal statistics in the Nordic countries 2008. Statistical Report 14/2010. Helsinki: Terveyden ja Hyvinvoinnin Laitos/National Institute for Health and Welfare (THL) [Finland]; 2010.
ISL	Iceland	2003	2003	18.2	Pohjoismaiset perinataalitilastot 2008 - Perinatal statistics in the Nordic countries 2008. Statistical Report 14/2010. Helsinki: Terveyden ja Hyvinvoinnin Laitos/National Institute for Health and Welfare (THL) [Finland]; 2010.
ISL	Iceland	2004	2004	16.6	Pohjoismaiset perinataalitilastot 2008 - Perinatal statistics in the Nordic countries 2008. Statistical Report 14/2010. Helsinki: Terveyden ja Hyvinvoinnin Laitos/National Institute for Health and Welfare (THL) [Finland]; 2010.
ISL	Iceland	2005	2005	15.8	Pohjoismaiset perinataalitilastot 2008 - Perinatal statistics in the Nordic countries 2008. Statistical Report 14/2010. Helsinki: Terveyden ja Hyvinvoinnin Laitos/National Institute for Health and Welfare (THL) [Finland]; 2010.
ISL	Iceland	2006	2006	17.5	Pohjoismaiset perinataalitilastot 2008 - Perinatal statistics in the Nordic countries 2008. Statistical Report 14/2010. Helsinki: Terveyden ja Hyvinvoinnin Laitos/National Institute for Health and Welfare (THL) [Finland]; 2010.
ISL	Iceland	2007	2007	17.2	Pohjoismaiset perinataalitilastot 2008 - Perinatal statistics in the Nordic countries 2008. Statistical Report 14/2010. Helsinki: Terveyden ja Hyvinvoinnin Laitos/National Institute for Health and Welfare (THL) [Finland]; 2010.

ISO Code	Country	Coverage start year	Coverage end year	Caesarean section rate (%)	References
ISL	Iceland	2008	2008	17.2	Hospital Statistics and Financial Accounts 2009. Reykjavík: Landspítali [Landspítali University Hospital], Office of Finance and Information Services; 2010.
ISL	Iceland	2009	2009	17.6	Hospital Statistics and Financial Accounts 2009. Reykjavík: Landspítali [Landspítali University Hospital], Office of Finance and Information Services; 2010.
ISL	Iceland	2010	2010	16.0	Hospital Statistics and Financial Accounts 2011. Reykjavík: Landspítali [Landspítali University Hospital], Office of Finance and Information Services; 2012.
ISL	Iceland	2011	2011	16.6	Hospital Statistics and Financial Accounts 2011. Reykjavík: Landspítali [Landspítali University Hospital], Office of Finance and Information Services; 2012.
ISL	Iceland	2012	2012	17.0	Hospital Statistics and Accounts 2012. Reykjavík: Landspítali [Landspítali University Hospital], Division of Finance and Information; 2013.
ISL	Iceland	2013	2013	17.5	Hospital Statistics and Accounts 2013. Reykjavík: Landspítali [Landspítali University Hospital], Division of Finance and Information; 2014.
ISL	Iceland	2014	2014	17.1	Hospital Statistics and Accounts 2014. Reykjavík: Landspítali [Landspítali University Hospital], Division of Finance and Information; 2015.
ISL	Iceland	2015	2015	17.8	Hospital Statistics and Accounts 2015. Reykjavík: Landspítali [Landspítali University Hospital], Division of Finance and Information; 2016.

ISO Code	Country	Coverage start year	Coverage end year	Caesarean section rate (%)	References
ISL	Iceland	2016	2016	18.3	Hospital Statistics and Accounts 2016. Reykjavík: Landspítali [Landspítali University Hospital], Division of Finance and Information; 2017.
ISL	Iceland	2017	2017	18.2	Hospital Statistics and Accounts 2017. Reykjavík: Landspítali [Landspítali University Hospital], Division of Finance and Information; 2018.
ISL	Iceland	2018	2018	17.2	Hospital Statistics and Accounts 2018. Reykjavík: Landspítali [Landspítali University Hospital], Division of Finance and Information; 2019.
IND	India	1987	1993	2.5	International Institute for Population Sciences (IIPS). National Family Health Survey (MCH and Family Planning), India 1992-93. Bombay, India: IIPS; 1995.
IND	India	1995	1999	7.1	International Institute for Population Sciences (IIPS), ORC Macro. National Family Health Survey (NFHS-2), 1998–99: India. Mumbai, India: IIPS; 2000.
IND	India	1999	2004	8.0	District Level Household and Facility Survey (DLHS-2), 2002-04: India. Mumbai: International Institute for Population Sciences (IIPS) [India]; 2006.
IND	India	2000	2006	8.5	International Institute for Population Sciences (IIPS), Macro International. National Family Health Survey (NFHS-3), 2005–06: India: Volume I. Mumbai: IIPS; 2007.
IND	India	2004	2008	8.2	District Level Household and Facility Survey (DLHS-3), 2007-08: India. Mumbai: International Institute for Population Sciences (IIPS) [India]; 2010.

ISO Code	Country	Coverage start year	Coverage end year	Caesarean section rate (%)	References
IND	India	2010	2016	17.2	National Family Health Survey (NFHS-4) 2015-16: India. Mumbai: International Institute for Population Sciences (IIPS), ICFP [India]; 2017.
IDN	Indonesia	1986	1991	1.3	Central Bureau of Statistics, National Family Planning Coordinating Board, Ministry of Health, Macro International Inc. Indonesia Demographic and Health Survey 1991. Columbia, Maryland, USA: Macro International Inc.; 1992.
IDN	Indonesia	1989	1994	2.5	Central Bureau of Statistics (CBS) [Indonesia], State Ministry of Population/National Family Planning Coordinating Board (NFPCB), Ministry of Health (MOH), Macro International Inc. (MI). Indonesia Demographic and Health Survey 1994. Calverton, Maryland: CBS, MI; 1995.
IDN	Indonesia	1992	1997	4.3	Central Bureau of Statistics (CBS) [Indonesia], State Ministry of Population/National Family Planning Coordinating Board (NFPCB), Ministry of Health (MOH), Macro International Inc. (MI). Indonesia Demographic and Health Survey 1997. Calverton, Maryland: CBS, MI; 1998.
IDN	Indonesia	1997	2003	4.1	Badan Pusat Statistik-Statistics Indonesia (BPS), ORC Macro. Indonesia Demographic and Health Survey 2002-2003. Calverton, Maryland, USA: BPS, ORC Macro; 2003.
IDN	Indonesia	2002	2007	6.8	Statistics Indonesia (Badan Pusat Statistik-BPS), Macro International. Indonesia Demographic and Health Survey 2007. Calverton, Maryland, USA: BPS, Macro International; 2008.
IDN	Indonesia	2007	2012	12.3	Statistics Indonesia (Badan Pusat Statistik—BPS), National Population and Family Planning Board (BKKBN), Kementerian Kesehatan (Kemenkes—MOH),ICF International. Indonesia Demographic and Health Survey 2012. Jakarta, Indonesia: BPS, BKKBN, Kemenkes, ICF International; 2013.
IRN	Iran (Islamic Republic of)	1998	2000	35.0	Ahmad-Nia S, Delavar B, Eini-Zinab H, Kazemipour S, Mehryar AH, Naghavi M. Caesarean section in the Islamic Republic of Iran: prevalence and some sociodemographic correlates. East Mediterr Health J. 2009;15(6):1389-98.

ISO Code	Country	Coverage start year	Coverage end year	Caesarean section rate (%)	References
IRN	Iran (Islamic Republic of)	2003	2005	40.0	Yazdizadeh B, Nedjat S, Mohammad K, Rashidian A, Changizi N, Majdzadeh R. Cesarean section rate in Iran, multidimensional approaches for behavioral change of providers: a qualitative study. BMC Health Serv Res. 2011;11:159. doi: 10.1186/1472-6963-11-159.
IRN	Iran (Islamic Republic of)	2008	2010	45.6	Rashidian A, Khosravi A, Khabiri R, Khodayari-Moez E, Elahi E, Arab M, Radaie Z. Islamic Republic of Iran’s Multiple Indicator Demographic and Health Survey (IrMIDHS) 2010. Tehran: Ministry of Health and Medical Education; 2012 (in Persian).
IRQ	Iraq	2004	2006	20.6	Iraq Multiple Indicator Cluster Survey 2006, Final Report. Baghdad: Central Organization for Statistics and Information Technology, Kurdistan Regional Statistics Office, Ministry of Health [Iraq], United Nations Children's Fund (UNICEF); 2007.
IRQ	Iraq	2009	2011	22.2	Iraq Multiple Indicator Cluster Survey 2011. Final Report. Baghdad: Central Statistical Organization (CSO), Kurdistan Regional Statistics Office (KRSO), United Nations Children’s Fund (UNICEF); 2012.
IRQ	Iraq	2008	2008	18.0	Shabila NP. Rates and trends in cesarean sections between 2008 and 2012 in Iraq. BMC Pregnancy Childbirth. 2017;17:22. doi: 10.1186/s12884-016-1211-6.
IRQ	Iraq	2012	2012	24.4	Shabila NP. Rates and trends in cesarean sections between 2008 and 2012 in Iraq. BMC Pregnancy Childbirth. 2017;17:22. doi: 10.1186/s12884-016-1211-6.
IRQ	Iraq	2016	2018	33.2	Iraq 2018 Multiple Indicator Cluster Survey (MICS6). Survey Findings Report. Baghdad: Central Statistical Organization (CSO), United Nations Children’s Fund (UNICEF); 2019.
IRL	Ireland	1990	1990	10.6	Report on Perinatal Statistics for 1999. Dublin: The Economic and Social Research Institute (ESRI); 2002.

ISO Code	Country	Coverage start year	Coverage end year	Caesarean section rate (%)	References
IRL	Ireland	1991	1991	11.8	Report on Perinatal Statistics for 1999. Dublin: The Economic and Social Research Institute (ESRI); 2002.
IRL	Ireland	1992	1992	12.2	Report on Perinatal Statistics for 1999. Dublin: The Economic and Social Research Institute (ESRI); 2002.
IRL	Ireland	1993	1993	13.0	Report on Perinatal Statistics for 1999. Dublin: The Economic and Social Research Institute (ESRI); 2002.
IRL	Ireland	1995	1995	13.4	European Health for All Database (HFA-DB) [online database]. World Health Organization (WHO) Regional Office for Europe; 2012 (http://data.euro.who.int/hfadb, accessed 20 August 2012).
IRL	Ireland	1996	1996	14.5	European Health for All Database (HFA-DB) [online database]. World Health Organization (WHO) Regional Office for Europe; 2012 (http://data.euro.who.int/hfadb, accessed 20 August 2012).
IRL	Ireland	1997	1997	15.3	European Health for All Database (HFA-DB) [online database]. World Health Organization (WHO) Regional Office for Europe; 2012 (http://data.euro.who.int/hfadb, accessed 20 August 2012).
IRL	Ireland	1998	1998	17.5	European Health for All Database (HFA-DB) [online database]. World Health Organization (WHO) Regional Office for Europe; 2012 (http://data.euro.who.int/hfadb, accessed 20 August 2012).
IRL	Ireland	1999	1999	20.0	Report on Perinatal Statistics for 1999. Dublin: The Economic and Social Research Institute (ESRI); 2002.

Betran AP, *et al. BMJ Global Health* 2021; 6:e005671. doi: 10.1136/bmjgh-2021-005671

ISO Code	Country	Coverage start year	Coverage end year	Caesarean section rate (%)	References
IRL	Ireland	2000	2000	20.9	Report on Perinatal Statistics for 2000. Dublin: The Economic and Social Research Institute (ESRI); 2004.
IRL	Ireland	2001	2001	21.5	Report on Perinatal Statistics for 2001. Dublin: The Economic and Social Research Institute (ESRI); 2005.
IRL	Ireland	2002	2002	21.9	Report on Perinatal Statistics for 2002. Dublin: The Economic and Social Research Institute (ESRI); 2005.
IRL	Ireland	2003	2003	23.6	Report on Perinatal Statistics for 2003. Dublin: Health Policy and Information Division, the Economic and Social Research Institute (ESRI); 2006.
IRL	Ireland	2004	2004	24.6	Report on Perinatal Statistics for 2004. Dublin: Health Policy and Information Division, the Economic and Social Research Institute (ESRI); 2007.
IRL	Ireland	2005	2005	25.3	Perinatal Statistics Report 2005. Dublin: Health Research and Information Division, the Economic and Social Research Institute (ESRI); 2008.
IRL	Ireland	2006	2006	24.8	Perinatal Statistics Report 2006. Dublin: Health Research and Information Division, the Economic and Social Research Institute (ESRI); 2008.
IRL	Ireland	2007	2007	25.5	Perinatal Statistics Report 2007. Dublin: Health Research and Information Division, the Economic and Social Research Institute (ESRI); 2009.

ISO Code	Country	Coverage start year	Coverage end year	Caesarean section rate (%)	References
IRL	Ireland	2008	2008	25.9	Perinatal Statistics Report 2008. Dublin: Health Research and Information Division, the Economic and Social Research Institute (ESRI); 2010.
IRL	Ireland	2009	2009	26.2	Perinatal Statistics Report 2009. Dublin: Health Research and Information Division, the Economic and Social Research Institute (ESRI); 2011.
IRL	Ireland	2010	2010	26.3	Perinatal Statistics Report 2010. Dublin: Health Research and Information Division, the Economic and Social Research Institute (ESRI); 2012.
IRL	Ireland	2011	2011	28.1	Perinatal Statistics Report 2011. Dublin: Health Research and Information Division, the Economic and Social Research Institute (ESRI); 2012.
IRL	Ireland	2012	2012	28.8	Perinatal Statistics Report 2012. Dublin: Health Research and Information Division, the Economic and Social Research Institute (ESRI); 2013.
IRL	Ireland	2013	2013	29.7	Healthcare Pricing Office (HPO), Health Service Executive (HSE). Perinatal Statistics Report 2013. Version 1.0. Dublin: HSE; 2014.
IRL	Ireland	2014	2014	30.4	Healthcare Pricing Office (HPO), Health Service Executive (HSE). Perinatal Statistics Report 2014. Version 1.0. Dublin: HSE; 2016.
IRL	Ireland	2015	2015	31.3	Healthcare Pricing Office (HPO), Health Service Executive (HSE). Perinatal Statistics Report 2015. Version 1.0. Dublin: HSE; 2017.

ISO Code	Country	Coverage start year	Coverage end year	Caesarean section rate (%)	References
IRL	Ireland	2016	2016	32.6	Healthcare Pricing Office (HPO), Health Service Executive (HSE). Perinatal Statistics Report 2016. Version 1.0. Dublin: HSE; 2018.
ISR	Israel	1990	1990	9.9	European Health for All Database (HFA-DB) [online database]. World Health Organization (WHO) Regional Office for Europe; 2012 (http://data.euro.who.int/hfadb, accessed 20 August 2012).
ISR	Israel	1993	1993	10.9	European Health for All Database (HFA-DB) [online database]. World Health Organization (WHO) Regional Office for Europe; 2012 (http://data.euro.who.int/hfadb, accessed 20 August 2012).
ISR	Israel	1994	1994	11.6	European Health for All Database (HFA-DB) [online database]. World Health Organization (WHO) Regional Office for Europe; 2012 (http://data.euro.who.int/hfadb, accessed 20 August 2012).
ISR	Israel	1995	1995	12.2	European Health for All Database (HFA-DB) [online database]. World Health Organization (WHO) Regional Office for Europe; 2012 (http://data.euro.who.int/hfadb, accessed 20 August 2012).
ISR	Israel	1996	1996	12.7	European Health for All Database (HFA-DB) [online database]. World Health Organization (WHO) Regional Office for Europe; 2012 (http://data.euro.who.int/hfadb, accessed 20 August 2012).
ISR	Israel	1997	1997	12.8	European Health for All Database (HFA-DB) [online database]. World Health Organization (WHO) Regional Office for Europe; 2012 (http://data.euro.who.int/hfadb, accessed 20 August 2012).
ISR	Israel	1998	1998	13.4	European Health for All Database (HFA-DB) [online database]. World Health Organization (WHO) Regional Office for Europe; 2012 (http://data.euro.who.int/hfadb, accessed 20 August 2012).

ISO Code	Country	Coverage start year	Coverage end year	Caesarean section rate (%)	References
ISR	Israel	1999	1999	14.6	European Health for All Database (HFA-DB) [online database]. World Health Organization (WHO) Regional Office for Europe; 2012 (http://data.euro.who.int/hfadb, accessed 20 August 2012).
ISR	Israel	2000	2000	15.8	European Health for All Database (HFA-DB) [online database]. World Health Organization (WHO) Regional Office for Europe; 2012 (http://data.euro.who.int/hfadb, accessed 20 August 2012).
ISR	Israel	2001	2001	16.7	European Health for All Database (HFA-DB) [online database]. World Health Organization (WHO) Regional Office for Europe; 2012 (http://data.euro.who.int/hfadb, accessed 20 August 2012).
ISR	Israel	2002	2002	17.3	European Health for All Database (HFA-DB) [online database]. World Health Organization (WHO) Regional Office for Europe; 2012 (http://data.euro.who.int/hfadb, accessed 20 August 2012).
ISR	Israel	2003	2003	17.5	European Health for All Database (HFA-DB) [online database]. World Health Organization (WHO) Regional Office for Europe; 2012 (http://data.euro.who.int/hfadb, accessed 20 August 2012).
ISR	Israel	2004	2004	17.6	European Health for All Database (HFA-DB) [online database]. World Health Organization (WHO) Regional Office for Europe; 2012 (http://data.euro.who.int/hfadb, accessed 20 August 2012).
ISR	Israel	2005	2005	18.6	European Health for All Database (HFA-DB) [online database]. World Health Organization (WHO) Regional Office for Europe; 2012 (http://data.euro.who.int/hfadb, accessed 20 August 2012).
ISR	Israel	2006	2006	18.7	European Health for All Database (HFA-DB) [online database]. World Health Organization (WHO) Regional Office for Europe; 2012 (http://data.euro.who.int/hfadb, accessed 20 August 2012).

ISO Code	Country	Coverage start year	Coverage end year	Caesarean section rate (%)	References
ISR	Israel	2007	2007	18.9	European Health for All Database (HFA-DB) [online database]. World Health Organization (WHO) Regional Office for Europe; 2012 (http://data.euro.who.int/hfadb, accessed 20 August 2012).
ISR	Israel	2008	2008	19.5	European Health for All Database (HFA-DB) [online database]. World Health Organization (WHO) Regional Office for Europe; 2012 (http://data.euro.who.int/hfadb, accessed 20 August 2012).
ISR	Israel	2009	2009	19.2	European Health for All Database (HFA-DB) [online database]. World Health Organization (WHO) Regional Office for Europe; 2012 (http://data.euro.who.int/hfadb, accessed 20 August 2012).
ISR	Israel	2010	2010	17.5	European Health for All Database (HFA-DB) [online database]. World Health Organization (WHO) Regional Office for Europe; 2016 (http://data.euro.who.int/hfadb, accessed 8 August 2016).
ISR	Israel	2011	2011	17.0	European Health for All Database (HFA-DB) [online database]. World Health Organization (WHO) Regional Office for Europe; 2016 (http://data.euro.who.int/hfadb, accessed 8 August 2016).
ISR	Israel	2012	2012	16.4	European Health for All Database (HFA-DB) [online database]. World Health Organization (WHO) Regional Office for Europe; 2016 (http://data.euro.who.int/hfadb, accessed 8 August 2016).
ISR	Israel	2013	2013	15.9	European Health for All Database (HFA-DB) [online database]. World Health Organization (WHO) Regional Office for Europe; 2016 (http://data.euro.who.int/hfadb, accessed 8 August 2016).
ISR	Israel	2014	2014	16.1	European Health for All Database (HFA-DB) [online database]. World Health Organization (WHO) Regional Office for Europe; 2016 (http://data.euro.who.int/hfadb, accessed 8 August 2016).

ISO Code	Country	Coverage start year	Coverage end year	Caesarean section rate (%)	References
ITA	Italy	1990	1990	20.8	European Health for All Database (HFA-DB) [online database]. World Health Organization (WHO) Regional Office for Europe; 2012 (http://data.euro.who.int/hfadb, accessed 20 August 2012).
ITA	Italy	1991	1991	22.6	European Health for All Database (HFA-DB) [online database]. World Health Organization (WHO) Regional Office for Europe; 2012 (http://data.euro.who.int/hfadb, accessed 20 August 2012).
ITA	Italy	1992	1992	22.9	European Health for All Database (HFA-DB) [online database]. World Health Organization (WHO) Regional Office for Europe; 2012 (http://data.euro.who.int/hfadb, accessed 20 August 2012).
ITA	Italy	1993	1993	24.1	European Health for All Database (HFA-DB) [online database]. World Health Organization (WHO) Regional Office for Europe; 2012 (http://data.euro.who.int/hfadb, accessed 20 August 2012).
ITA	Italy	1994	1994	24.8	European Health for All Database (HFA-DB) [online database]. World Health Organization (WHO) Regional Office for Europe; 2012 (http://data.euro.who.int/hfadb, accessed 20 August 2012).
ITA	Italy	1995	1995	26.1	European Health for All Database (HFA-DB) [online database]. World Health Organization (WHO) Regional Office for Europe; 2012 (http://data.euro.who.int/hfadb, accessed 20 August 2012).
ITA	Italy	1996	1996	23.5	European Health for All Database (HFA-DB) [online database]. World Health Organization (WHO) Regional Office for Europe; 2012 (http://data.euro.who.int/hfadb, accessed 20 August 2012).
ITA	Italy	1997	1997	29.4	Demografia in Cifre. La rilevazione delle nascite di fonte stato civile. Anni 1997 e 1998. Tavola 1.1 – Indicatori sintetici regionali – Anno 1997. [website]. Roma: Istituto Nazionale di Statistica [Italian National Institute of Statistics] (ISTAT); 1999 (http://demo.istat.it/altridati/natid1d2/index.html, accessed 12 September 2012, in Italian).

ISO Code	Country	Coverage start year	Coverage end year	Caesarean section rate (%)	References
ITA	Italy	1998	1998	30.3	Demografia in Cifre. La rilevazione delle nascite di fonte stato civile. Anni 1997 e 1998. Tavola 1.1 – Indicatori sintetici regionali – Anno 1998. [website]. Roma: Istituto Nazionale di Statistica [Italian National Institute of Statistics] (ISTAT); 1999 (http://demo.istat.it/altridati/natid1d2/index.html, accessed 12 September 2012, in Italian).
ITA	Italy	1999	1999	31.6	European Health for All Database (HFA-DB) [online database]. World Health Organization (WHO) Regional Office for Europe; 2012 (http://data.euro.who.int/hfadb, accessed 20 August 2012).
ITA	Italy	2000	2000	33.0	European Health for All Database (HFA-DB) [online database]. World Health Organization (WHO) Regional Office for Europe; 2012 (http://data.euro.who.int/hfadb, accessed 20 August 2012).
ITA	Italy	2001	2001	34.5	European Health for All Database (HFA-DB) [online database]. World Health Organization (WHO) Regional Office for Europe; 2012 (http://data.euro.who.int/hfadb, accessed 20 August 2012).
ITA	Italy	2002	2002	35.3	Certificato di assistenza al parto (CeDAP). Analisi dell'evento nascita - Anno 2002. Roma: Direzione Generale del Sistema Informativo – Ufficio di Statistica; 2004 (in Italian).
ITA	Italy	2003	2003	35.8	Certificato di assistenza al parto (CeDAP). Analisi dell'evento nascita - Anno 2003. Roma: Direzione Generale del Sistema Informativo – Ufficio di Statistica; 2005 (in Italian).
ITA	Italy	2004	2004	36.4	Certificato di assistenza al parto (CeDAP). Analisi dell'evento nascita - Anno 2004. Roma: Direzione Generale del Sistema Informativo – Ufficio di Statistica; 2007 (in Italian).
ITA	Italy	2005	2005	37.3	Certificato di assistenza al parto (CeDAP). Analisi dell'evento nascita - Anno 2005. Roma: Direzione Generale del Sistema Informativo – Ufficio di Statistica; 2008 (in Italian).

ISO Code	Country	Coverage start year	Coverage end year	Caesarean section rate (%)	References
ITA	Italy	2006	2006	37.4	Certificato di assistenza al parto (CeDAP). Analisi dell'evento nascita - Anno 2006. Roma: Direzione Generale del Sistema Informativo – Ufficio di Statistica; 2009 (in Italian).
ITA	Italy	2007	2007	37.4	Certificato di assistenza al parto (CeDAP). Analisi dell'evento nascita - Anno 2007. Roma: Direzione Generale del Sistema Informativo – Ufficio di Statistica; 2010 (in Italian).
ITA	Italy	2008	2008	37.8	Certificato di assistenza al parto (CeDAP). Analisi dell'evento nascita - Anno 2008. Roma: Direzione Generale del Sistema Informativo – Ufficio di Statistica; 2011 (in Italian).
ITA	Italy	2009	2009	38.0	Certificato di assistenza al parto (CeDAP). Analisi dell'evento nascita - Anno 2009. Roma: Direzione Generale del Sistema Informativo – Ufficio di Statistica; 2012 (in Italian).
ITA	Italy	2010	2010	37.5	Certificato di assistenza al parto (CeDAP). Analisi dell'evento nascita - Anno 2010. Roma: Direzione Generale del Sistema Informativo e Statistico Sanitario – Ufficio di Statistica; 2013 (in Italian).
ITA	Italy	2011	2011	38.1	European Health for All Database (HFA-DB) [online database]. World Health Organization (WHO) Regional Office for Europe; 2016 (http://data.euro.who.int/hfadb, accessed 8 August 2016).
ITA	Italy	2012	2012	37.3	European Health for All Database (HFA-DB) [online database]. World Health Organization (WHO) Regional Office for Europe; 2016 (http://data.euro.who.int/hfadb, accessed 8 August 2016).
ITA	Italy	2013	2013	35.6	Certificato di assistenza al parto (CeDAP). Analisi dell'evento nascita - Anno 2013. Roma: Direzione Generale della Digitalizzazione, del Sistema Informativo Sanitario e della Statistica – Ufficio di Statistica; 2015 (in Italian).

ISO Code	Country	Coverage start year	Coverage end year	Caesarean section rate (%)	References
ITA	Italy	2014	2014	35.0	Certificato di assistenza al parto (CeDAP). Analisi dell'evento nascita - Anno 2014. Roma: Direzione Generale della Digitalizzazione, del Sistema Informativo Sanitario e della Statistica – Ufficio di Statistica; 2017 (in Italian).
ITA	Italy	2015	2015	34.3	Certificato di assistenza al parto (CeDAP). Analisi dell'evento nascita - Anno 2015. Roma: Direzione Generale della Digitalizzazione, del Sistema Informativo Sanitario e della Statistica – Ufficio di Statistica; 2018 (in Italian).
ITA	Italy	2016	2016	33.7	Certificato di assistenza al parto (CeDAP). Analisi dell'evento nascita - Anno 2016. Roma: Direzione Generale della Digitalizzazione, del Sistema Informativo Sanitario e della Statistica – Ufficio di Statistica; 2019 (in Italian).
JAM	Jamaica	1993	1993	6.6	1993 Contraceptive Prevalence Survey Jamaica. Volume III - Sexual Experience Contraceptive Practice and Fertility. Kingston, Atlanta, GA: National Family Planning Board [Jamaica], Centers for Disease Control and Prevention (CDC); 1994.
JAM	Jamaica	2003	2003	14.2	Annual Report 2007. Kingston: Ministry of Health and Environment - Policy, Planning and Development Division, Planning and Evaluation Branch [Jamaica]; 2009.
JAM	Jamaica	2004	2004	14.2	Annual Report 2007. Kingston: Ministry of Health and Environment - Policy, Planning and Development Division, Planning and Evaluation Branch [Jamaica]; 2009.
JAM	Jamaica	2005	2005	13.8	Annual Report 2007. Kingston: Ministry of Health and Environment - Policy, Planning and Development Division, Planning and Evaluation Branch [Jamaica]; 2009.
JAM	Jamaica	2006	2006	15.3	Annual Report 2007. Kingston: Ministry of Health and Environment - Policy, Planning and Development Division, Planning and Evaluation Branch [Jamaica]; 2009.

ISO Code	Country	Coverage start year	Coverage end year	Caesarean section rate (%)	References
JAM	Jamaica	2007	2007	15.2	Annual Report 2007. Kingston: Ministry of Health and Environment - Policy, Planning and Development Division, Planning and Evaluation Branch [Jamaica]; 2009.
JAM	Jamaica	2009	2011	21.2	Statistical Institute of Jamaica (STATIN), United Nations Children's Fund (UNICEF). Jamaica Multiple Indicator Cluster Survey 2011. Final Report. Kingston, Jamaica: STATIN, UNICEF; 2013.
JPN	Japan	1990	1990	11.2	Summary of Health Care Facilities and Hospital Statistics 2008. [平成２０年 ２００８医療施設(静態・動態)調査・病院報告の概況)]. Tokyo: Ministry of Health, Labour and Welfare [Japan]; 2009 (in Japanese).
JPN	Japan	1993	1993	13.8	Summary of Health Care Facilities and Hospital Statistics 2008. [平成２０年 ２００８医療施設(静態・動態)調査・病院報告の概況)]. Tokyo: Ministry of Health, Labour and Welfare [Japan]; 2009 (in Japanese).
JPN	Japan	1996	1996	14.7	Summary of Health Care Facilities and Hospital Statistics 2008. [平成２０年 ２００８医療施設(静態・動態)調査・病院報告の概況)]. Tokyo: Ministry of Health, Labour and Welfare [Japan]; 2009 (in Japanese).
JPN	Japan	1999	1999	17.4	Summary of Health Care Facilities and Hospital Statistics 2008. [平成２０年 ２００８医療施設(静態・動態)調査・病院報告の概況)]. Tokyo: Ministry of Health, Labour and Welfare [Japan]; 2009 (in Japanese).
JPN	Japan	2002	2002	17.9	Summary of Health Care Facilities and Hospital Statistics 2008. [平成２０年 ２００８医療施設(静態・動態)調査・病院報告の概況)]. Tokyo: Ministry of Health, Labour and Welfare [Japan]; 2009 (in Japanese).
JPN	Japan	2005	2005	21.4	Summary of Health Care Facilities and Hospital Statistics 2008. [平成２０年 ２００８医療施設(静態・動態)調査・病院報告の概況)]. Tokyo: Ministry of Health, Labour and Welfare [Japan]; 2009 (in Japanese).

ISO Code	Country	Coverage start year	Coverage end year	Caesarean section rate (%)	References
JPN	Japan	2008	2008	23.3	Summary of Health Care Facilities and Hospital Statistics 2008. [平成２０年 ２００８医療施設(静態・動態)調査・病院報告の概況)]. Tokyo: Ministry of Health, Labour and Welfare [Japan]; 2009 (in Japanese).
JPN	Japan	2011	2011	19.2	Summary of Health Care Facilities and Hospital Statistics 2011 (平成 23 年（2011）医療施設(静態・動態)調査･病院報告の概況). Tokyo: Ministry of Health, Labour and Welfare [Japan]; 2012 (in Japanese).
JPN	Japan	2014	2014	19.7	Summary of Health Care Facilities and Hospital Statistics 2014 (平成 26 年（2014）医療施設(静態・動態)調査･病院報告の概況). Tokyo: Ministry of Health, Labour and Welfare [Japan]; 2015 (in Japanese).
JOR	Jordan	1985	1990	5.7	Abdel Aziz Zou'bi A, Poedjastoeti S, Ayad M (Department of Statistics [Jordan], Ministry of Health [Jordan], IRD/Macro International). Jordan Population and Family Health Survey 1990. Columbia, Maryland, USA: Department of Statistics [Jordan], Ministry of Health [Jordan], IRD/Macro International; 1992.
JOR	Jordan	1992	1997	10.5	Department of Statistics (DOS) [Jordan], Macro International Inc. (MI). Jordan Population and Family Health Survey 1997. Calverton, Maryland: DOS, MI; 1998.
JOR	Jordan	1997	2002	16.0	Department of Statistics [Jordan], ORC Macro. Jordan Population and Family Health Survey 2002. Calverton, Maryland, USA: Department of Statistics, ORC Macro; 2003.
JOR	Jordan	2002	2007	18.5	Department of Statistics [Jordan], Macro International Inc. Jordan Population and Family Health Survey 2007. Calverton, Maryland, USA: Department of Statistics, Macro International Inc.; 2008.
JOR	Jordan	2007	2012	28.0	Department of Statistics [Jordan], ICF International. Jordan Population and Family Health Survey 2012. Calverton, Maryland, USA: Department of Statistics, ICF International; 2013.

ISO Code	Country	Coverage start year	Coverage end year	Caesarean section rate (%)	References
JOR	Jordan	2013	2018	25.8	Jordan Population and Family and Health Survey 2017-18. Amman, Jordan, and Rockville, Maryland, USA: Department of Statistics (DOS) , ICF; 2019.
KAZ	Kazakhstan	1990	1990	4.6	European Health for All Database (HFA-DB) [online database]. World Health Organization (WHO) Regional Office for Europe; 2012 (http://data.euro.who.int/hfadb, accessed 20 August 2012).
KAZ	Kazakhstan	1991	1991	5.0	European Health for All Database (HFA-DB) [online database]. World Health Organization (WHO) Regional Office for Europe; 2012 (http://data.euro.who.int/hfadb, accessed 20 August 2012).
KAZ	Kazakhstan	1992	1992	5.3	European Health for All Database (HFA-DB) [online database]. World Health Organization (WHO) Regional Office for Europe; 2012 (http://data.euro.who.int/hfadb, accessed 20 August 2012).
KAZ	Kazakhstan	1992	1995	4.6	National Institute of Nutrition [Kazakhstan], Macro International Inc. Kazakhstan Demographic and Health Survey, 1995. Calverton, Maryland: National Institute of Nutrition, Macro International Inc.; 1996.
KAZ	Kazakhstan	1995	1995	5.2	European Health for All Database (HFA-DB) [online database]. World Health Organization (WHO) Regional Office for Europe; 2012 (http://data.euro.who.int/hfadb, accessed 20 August 2012).
KAZ	Kazakhstan	1998	1998	6.5	European Health for All Database (HFA-DB) [online database]. World Health Organization (WHO) Regional Office for Europe; 2012 (http://data.euro.who.int/hfadb, accessed 20 August 2012).
KAZ	Kazakhstan	1994	1999	9.6	Academy of Preventive Medicine [Kazakhstan], Macro International Inc. Kazakhstan Demographic and Health Survey 1999. Calverton, Maryland: Academy of Preventive Medicine, Macro International Inc.; 2000.

ISO Code	Country	Coverage start year	Coverage end year	Caesarean section rate (%)	References
KAZ	Kazakhstan	1999	1999	6.8	European Health for All Database (HFA-DB) [online database]. World Health Organization (WHO) Regional Office for Europe; 2012 (http://data.euro.who.int/hfadb, accessed 20 August 2012).
KAZ	Kazakhstan	2000	2000	6.8	European Health for All Database (HFA-DB) [online database]. World Health Organization (WHO) Regional Office for Europe; 2012 (http://data.euro.who.int/hfadb, accessed 20 August 2012).
KAZ	Kazakhstan	2001	2001	7.4	European Health for All Database (HFA-DB) [online database]. World Health Organization (WHO) Regional Office for Europe; 2012 (http://data.euro.who.int/hfadb, accessed 20 August 2012).
KAZ	Kazakhstan	2002	2002	8.1	European Health for All Database (HFA-DB) [online database]. World Health Organization (WHO) Regional Office for Europe; 2012 (http://data.euro.who.int/hfadb, accessed 20 August 2012).
KAZ	Kazakhstan	2003	2003	9.3	European Health for All Database (HFA-DB) [online database]. World Health Organization (WHO) Regional Office for Europe; 2012 (http://data.euro.who.int/hfadb, accessed 20 August 2012).
KAZ	Kazakhstan	2004	2004	9.4	European Health for All Database (HFA-DB) [online database]. World Health Organization (WHO) Regional Office for Europe; 2012 (http://data.euro.who.int/hfadb, accessed 20 August 2012).
KAZ	Kazakhstan	2005	2005	10.1	European Health for All Database (HFA-DB) [online database]. World Health Organization (WHO) Regional Office for Europe; 2012 (http://data.euro.who.int/hfadb, accessed 20 August 2012).
KAZ	Kazakhstan	2006	2006	10.7	European Health for All Database (HFA-DB) [online database]. World Health Organization (WHO) Regional Office for Europe; 2012 (http://data.euro.who.int/hfadb, accessed 20 August 2012).

ISO Code	Country	Coverage start year	Coverage end year	Caesarean section rate (%)	References
KAZ	Kazakhstan	2007	2007	11.7	European Health for All Database (HFA-DB) [online database]. World Health Organization (WHO) Regional Office for Europe; 2012 (http://data.euro.who.int/hfadb, accessed 20 August 2012).
KAZ	Kazakhstan	2008	2008	12.4	European Health for All Database (HFA-DB) [online database]. World Health Organization (WHO) Regional Office for Europe; 2012 (http://data.euro.who.int/hfadb, accessed 20 August 2012).
KAZ	Kazakhstan	2010	2010	13.5	European Health for All Database (HFA-DB) [online database]. World Health Organization (WHO) Regional Office for Europe; 2016 (http://data.euro.who.int/hfadb, accessed 8 August 2016).
KAZ	Kazakhstan	2008	2010	15.9	The Agency of Statistics, RK, United Nations Children's Fund (UNICEF). Multiple Indicator Cluster Survey (MICS) in the Republic of Kazakhstan, 2010-2011. Final Report. Astana, Kazakhstan: the Agency of Statistics, RK, the Republican State Enterprise Information Computing Center; 2012.
KAZ	Kazakhstan	2011	2011	14.6	European Health for All Database (HFA-DB) [online database]. World Health Organization (WHO) Regional Office for Europe; 2016 (http://data.euro.who.int/hfadb, accessed 8 August 2016).
KAZ	Kazakhstan	2012	2012	15.2	European Health for All Database (HFA-DB) [online database]. World Health Organization (WHO) Regional Office for Europe; 2016 (http://data.euro.who.int/hfadb, accessed 8 August 2016).
KAZ	Kazakhstan	2013	2013	15.1	European Health for All Database (HFA-DB) [online database]. World Health Organization (WHO) Regional Office for Europe; 2016 (http://data.euro.who.int/hfadb, accessed 8 August 2016).
KAZ	Kazakhstan	2015	2015	16.0	European Health for All Database (HFA-DB) [online database]. World Health Organization (WHO) Regional Office for Europe; 2016 (http://data.euro.who.int/hfadb, accessed 8 August 2016).

ISO Code	Country	Coverage start year	Coverage end year	Caesarean section rate (%)	References
KAZ	Kazakhstan	2013	2015	14.8	The Statistics Committee of the Ministry of National Economy of the Republic of Kazakhstan (Statistics Committee of the MNE RK), the United Nations Children's Fund (UNICEF), the United Nations Population Fund (UNFPA). 2015 Kazakhstan Multiple Indicator Cluster Survey, Final Report. Astana, Kazakhstan: The Statistics Committee of the MNE RK, UNICEF, UNFPA; 2016.
KAZ	Kazakhstan	2017	2017	18.0	European Health Information Gateway. European Health for All database (HFA-DB). Caesarean sections per 1000 live births [online database]. World Health Organization (WHO) Regional Office for Europe; 2019 (https://gateway.euro.who.int/en/indicators/hfa_596-7060-caesarean-sections-per-1000-live-births/, accessed 5 March 2020).
KEN	Kenya	1988	1993	5.2	National Council for Population and Development (NCPD), Central Bureau of Statistics (CBS) (Office of the Vice President and Ministry of Planning and National Development [Kenya]), Macro International Inc. (MI). Kenya Demographic and Health Survey 1993. Calverton, Maryland: NCPD, CBS, MI; 1994.
KEN	Kenya	1995	1998	6.8	National Council for Population and Development (NCPD), Central Bureau of Statistics (CBS) (Office of the Vice President and Ministry of Planning and National Development) [Kenya], Macro International Inc. (MI). Kenya Demographic and Health Survey 1998. Calverton, Maryland: NDPD, CBS, and MI; 1999.
KEN	Kenya	1998	2003	4.0	Central Bureau of Statistics (CBS) [Kenya], Ministry of Health (MOH) [Kenya], ORC Macro. Kenya Demographic and Health Survey 2003. Calverton, Maryland: CBS, MOH, ORC Macro; 2004.
KEN	Kenya	2003	2009	6.2	Kenya National Bureau of Statistics (KNBS), ICF Macro. Kenya Demographic and Health Survey 2008-09. Calverton, Maryland: KNBS, ICF Macro; 2010.
KEN	Kenya	2009	2009	8.7	Kenya National Bureau of Statistics, Ministry of Health, National AIDS Control Council, Kenya Medical Research Institute, National Council for Population and Development, Nairobi, Kenya, The DHS Program. Kenya Demographic and Health Survey 2014. Rockville, Maryland, USA: ICF International; 2015.
KIR	Kiribati	2004	2009	9.6	Kiribati National Statistics Office (KNSO), Secretariat of the Pacific Community (SPC) [New Caledonia]. Kiribati Demographic and Health Survey 2009. Noumea: SPC; 2010.

ISO Code	Country	Coverage start year	Coverage end year	Caesarean section rate (%)	References
KWT	Kuwait	1993	1996	11.2	Kuwait Family Health Survey 1996. Preliminary Report. Kuwait: Ministry of Health [Kuwait], Council of Health Ministers of GCC States; 1997.
KGZ	Kyrgyzstan	1990	1990	3.1	European Health for All Database (HFA-DB) [online database]. World Health Organization (WHO) Regional Office for Europe; 2012 (http://data.euro.who.int/hfadb, accessed 20 August 2012).
KGZ	Kyrgyzstan	1991	1991	3.0	European Health for All Database (HFA-DB) [online database]. World Health Organization (WHO) Regional Office for Europe; 2012 (http://data.euro.who.int/hfadb, accessed 20 August 2012).
KGZ	Kyrgyzstan	1992	1992	3.9	European Health for All Database (HFA-DB) [online database]. World Health Organization (WHO) Regional Office for Europe; 2012 (http://data.euro.who.int/hfadb, accessed 20 August 2012).
KGZ	Kyrgyzstan	1993	1993	3.1	European Health for All Database (HFA-DB) [online database]. World Health Organization (WHO) Regional Office for Europe; 2012 (http://data.euro.who.int/hfadb, accessed 20 August 2012).
KGZ	Kyrgyzstan	1994	1994	3.2	European Health for All Database (HFA-DB) [online database]. World Health Organization (WHO) Regional Office for Europe; 2012 (http://data.euro.who.int/hfadb, accessed 20 August 2012).
KGZ	Kyrgyzstan	1994	1997	6.0	Research Institute of Obstetrics and Pediatrics [Kyrgyz Republic], Macro International Inc. Kyrgyz Republic Demographic and Health Survey, 1997. Calverton, Maryland: Research Institute of Obstetrics and Pediatrics, Ministry of Health of the Kyrgyz Republic, Macro International Inc.; 1998.
KGZ	Kyrgyzstan	1997	1997	3.9	European Health for All Database (HFA-DB) [online database]. World Health Organization (WHO) Regional Office for Europe; 2012 (http://data.euro.who.int/hfadb, accessed 20 August 2012).

ISO Code	Country	Coverage start year	Coverage end year	Caesarean section rate (%)	References
KGZ	Kyrgyzstan	1998	1998	3.8	European Health for All Database (HFA-DB) [online database]. World Health Organization (WHO) Regional Office for Europe; 2012 (http://data.euro.who.int/hfadb, accessed 20 August 2012).
KGZ	Kyrgyzstan	1999	1999	4.0	European Health for All Database (HFA-DB) [online database]. World Health Organization (WHO) Regional Office for Europe; 2012 (http://data.euro.who.int/hfadb, accessed 20 August 2012).
KGZ	Kyrgyzstan	2000	2000	3.9	European Health for All Database (HFA-DB) [online database]. World Health Organization (WHO) Regional Office for Europe; 2012 (http://data.euro.who.int/hfadb, accessed 20 August 2012).
KGZ	Kyrgyzstan	2001	2001	4.1	European Health for All Database (HFA-DB) [online database]. World Health Organization (WHO) Regional Office for Europe; 2012 (http://data.euro.who.int/hfadb, accessed 20 August 2012).
KGZ	Kyrgyzstan	2002	2002	4.0	European Health for All Database (HFA-DB) [online database]. World Health Organization (WHO) Regional Office for Europe; 2012 (http://data.euro.who.int/hfadb, accessed 20 August 2012).
KGZ	Kyrgyzstan	2003	2003	4.2	European Health for All Database (HFA-DB) [online database]. World Health Organization (WHO) Regional Office for Europe; 2012 (http://data.euro.who.int/hfadb, accessed 20 August 2012).
KGZ	Kyrgyzstan	2004	2004	5.1	European Health for All Database (HFA-DB) [online database]. World Health Organization (WHO) Regional Office for Europe; 2012 (http://data.euro.who.int/hfadb, accessed 20 August 2012).
KGZ	Kyrgyzstan	2005	2005	5.1	European Health for All Database (HFA-DB) [online database]. World Health Organization (WHO) Regional Office for Europe; 2012 (http://data.euro.who.int/hfadb, accessed 20 August 2012).

ISO Code	Country	Coverage start year	Coverage end year	Caesarean section rate (%)	References
KGZ	Kyrgyzstan	2006	2006	5.1	European Health for All Database (HFA-DB) [online database]. World Health Organization (WHO) Regional Office for Europe; 2012 (http://data.euro.who.int/hfadb, accessed 20 August 2012).
KGZ	Kyrgyzstan	2007	2007	5.8	European Health for All Database (HFA-DB) [online database]. World Health Organization (WHO) Regional Office for Europe; 2012 (http://data.euro.who.int/hfadb, accessed 20 August 2012).
KGZ	Kyrgyzstan	2008	2008	6.4	European Health for All Database (HFA-DB) [online database]. World Health Organization (WHO) Regional Office for Europe; 2012 (http://data.euro.who.int/hfadb, accessed 20 August 2012).
KGZ	Kyrgyzstan	2009	2009	7.3	European Health for All Database (HFA-DB) [online database]. World Health Organization (WHO) Regional Office for Europe; 2012 (http://data.euro.who.int/hfadb, accessed 20 August 2012).
KGZ	Kyrgyzstan	2010	2010	6.9	European Health for All Database (HFA-DB) [online database]. World Health Organization (WHO) Regional Office for Europe; 2016 (http://data.euro.who.int/hfadb, accessed 8 August 2016).
KGZ	Kyrgyzstan	2011	2011	7.0	European Health for All Database (HFA-DB) [online database]. World Health Organization (WHO) Regional Office for Europe; 2016 (http://data.euro.who.int/hfadb, accessed 8 August 2016).
KGZ	Kyrgyzstan	2012	2012	7.7	European Health for All Database (HFA-DB) [online database]. World Health Organization (WHO) Regional Office for Europe; 2016 (http://data.euro.who.int/hfadb, accessed 8 August 2016).
KGZ	Kyrgyzstan	2014	2014	9.8	European Health for All Database (HFA-DB) [online database]. World Health Organization (WHO) Regional Office for Europe; 2016 (http://data.euro.who.int/hfadb, accessed 8 August 2016).

ISO Code	Country	Coverage start year	Coverage end year	Caesarean section rate (%)	References
KGZ	Kyrgyzstan	2012	2014	7.4	National Statistical Committee of the Kyrgyz Republic, United Nations Children's Fund (UNICEF). Kyrgyz Republic Multiple Indicator Cluster Survey 2014, Final Report. Bishkek, Kyrgyzstan: National Statistical Committee of the Kyrgyz Republic, UNICEF; 2016.
KGZ	Kyrgyzstan	2015	2015	9.4	European Health for All Database (HFA-DB) [online database]. World Health Organization (WHO) Regional Office for Europe; 2016 (http://data.euro.who.int/hfadb, accessed 8 August 2016).
KGZ	Kyrgyzstan	2016	2018	8.3	Kyrgyztan Multiple Indicator Cluster Survey 2018, Survey Findings Report. Bishkek: National Statistical Committee of the Kyrgyz Republic, UNICEF; 2019.
LAO	Lao People's Democratic Republic	2006	2007	2.0	Assessment of Skilled Birth Attendance in Lao PDR. Vientiane: Ministry of Health [Lao], United Nations Population Fund (UNFPA); 2008.
LAO	Lao People's Democratic Republic	2009	2010	2.0	National Health Statistic Report FY 2009-2010. Vientiane: Ministry of Health, Department of Planning and Finance [Lao]; 2011.
LAO	Lao People's Democratic Republic	2015	2017	5.8	Lao Statistics Bureau. Lao Social Indicator Survey II 2017, Survey Findings Report. Vientiane, Lao PDR: Lao Statistics Bureau, UNICEF; 2018.
LVA	Latvia	1990	1990	7.0	European Health for All Database (HFA-DB) [online database]. World Health Organization (WHO) Regional Office for Europe; 2012 (http://data.euro.who.int/hfadb, accessed 20 August 2012).
LVA	Latvia	1991	1991	7.8	European Health for All Database (HFA-DB) [online database]. World Health Organization (WHO) Regional Office for Europe; 2012 (http://data.euro.who.int/hfadb, accessed 20 August 2012).

ISO Code	Country	Coverage start year	Coverage end year	Caesarean section rate (%)	References
LVA	Latvia	1992	1992	7.5	European Health for All Database (HFA-DB) [online database]. World Health Organization (WHO) Regional Office for Europe; 2012 (http://data.euro.who.int/hfadb, accessed 20 August 2012).
LVA	Latvia	1993	1993	9.0	European Health for All Database (HFA-DB) [online database]. World Health Organization (WHO) Regional Office for Europe; 2012 (http://data.euro.who.int/hfadb, accessed 20 August 2012).
LVA	Latvia	1994	1994	9.4	European Health for All Database (HFA-DB) [online database]. World Health Organization (WHO) Regional Office for Europe; 2012 (http://data.euro.who.int/hfadb, accessed 20 August 2012).
LVA	Latvia	1995	1995	10.4	European Health for All Database (HFA-DB) [online database]. World Health Organization (WHO) Regional Office for Europe; 2012 (http://data.euro.who.int/hfadb, accessed 20 August 2012).
LVA	Latvia	1996	1996	11.5	European Health for All Database (HFA-DB) [online database]. World Health Organization (WHO) Regional Office for Europe; 2012 (http://data.euro.who.int/hfadb, accessed 20 August 2012).
LVA	Latvia	1997	1997	12.3	European Health for All Database (HFA-DB) [online database]. World Health Organization (WHO) Regional Office for Europe; 2012 (http://data.euro.who.int/hfadb, accessed 20 August 2012).
LVA	Latvia	1998	1998	15.8	European Health for All Database (HFA-DB) [online database]. World Health Organization (WHO) Regional Office for Europe; 2012 (http://data.euro.who.int/hfadb, accessed 20 August 2012).
LVA	Latvia	1999	1999	14.2	European Health for All Database (HFA-DB) [online database]. World Health Organization (WHO) Regional Office for Europe; 2012 (http://data.euro.who.int/hfadb, accessed 20 August 2012).

ISO Code	Country	Coverage start year	Coverage end year	Caesarean section rate (%)	References
LVA	Latvia	2000	2000	15.0	European Health for All Database (HFA-DB) [online database]. World Health Organization (WHO) Regional Office for Europe; 2012 (http://data.euro.who.int/hfadb, accessed 20 August 2012).
LVA	Latvia	2001	2001	16.4	European Health for All Database (HFA-DB) [online database]. World Health Organization (WHO) Regional Office for Europe; 2012 (http://data.euro.who.int/hfadb, accessed 20 August 2012).
LVA	Latvia	2002	2002	17.4	European Health for All Database (HFA-DB) [online database]. World Health Organization (WHO) Regional Office for Europe; 2012 (http://data.euro.who.int/hfadb, accessed 20 August 2012).
LVA	Latvia	2003	2003	19.1	European Health for All Database (HFA-DB) [online database]. World Health Organization (WHO) Regional Office for Europe; 2012 (http://data.euro.who.int/hfadb, accessed 20 August 2012).
LVA	Latvia	2004	2004	19.6	European Health for All Database (HFA-DB) [online database]. World Health Organization (WHO) Regional Office for Europe; 2012 (http://data.euro.who.int/hfadb, accessed 20 August 2012).
LVA	Latvia	2005	2005	20.4	Statistical Yearbook of Health Care in Latvia 12th edition, 2010. Riga: The Centre of Health Economics [Latvia]; 2011.
LVA	Latvia	2006	2006	21.1	Statistical Yearbook of Health Care in Latvia 12th edition, 2010. Riga: The Centre of Health Economics [Latvia]; 2011.
LVA	Latvia	2007	2007	23.1	Statistical Yearbook of Health Care in Latvia 12th edition, 2010. Riga: The Centre of Health Economics [Latvia]; 2011.

ISO Code	Country	Coverage start year	Coverage end year	Caesarean section rate (%)	References
LVA	Latvia	2008	2008	22.7	Statistical Yearbook of Health Care in Latvia 12th edition, 2010. Riga: The Centre of Health Economics [Latvia]; 2011.
LVA	Latvia	2009	2009	23.4	Statistical Yearbook of Health Care in Latvia 12th edition, 2010. Riga: The Centre of Health Economics [Latvia]; 2011.
LVA	Latvia	2010	2010	23.9	Statistical Yearbook of Health Care in Latvia 12th edition, 2010. Riga: The Centre of Health Economics [Latvia]; 2011.
LVA	Latvia	2011	2011	23.6	Statistical Yearbook of Health Care in Latvia 13th edition, 2011. Riga: The Centre for Disease Prevention and Control of Latvia; 2012.
LVA	Latvia	2012	2012	23.0	Statistical Yearbook of Health Care in Latvia 14th edition, 2012. Riga: The Centre for Disease Prevention and Control of Latvia; 2013.
LVA	Latvia	2013	2013	20.9	European Health for All Database (HFA-DB) [online database]. World Health Organization (WHO) Regional Office for Europe; 2016 (http://data.euro.who.int/hfadb, accessed 8 August 2016).
LVA	Latvia	2014	2014	19.9	European Health for All Database (HFA-DB) [online database]. World Health Organization (WHO) Regional Office for Europe; 2016 (http://data.euro.who.int/hfadb, accessed 8 August 2016).
LVA	Latvia	2015	2015	21.5	Statistical Yearbook of Health Care in Latvia 17th edition, 2015. Riga: The Centre for Disease Prevention and Control of Latvia; 2016.

ISO Code	Country	Coverage start year	Coverage end year	Caesarean section rate (%)	References
LVA	Latvia	2016	2016	21.7	Statistical Yearbook of Health Care in Latvia 18th edition, 2016. Riga: The Centre for Disease Prevention and Control of Latvia; 2017.
LVA	Latvia	2017	2017	22.7	Statistical Yearbook of Health Care in Latvia 19th edition, 2017. Riga: The Centre for Disease Prevention and Control of Latvia; 2018.
LBN	Lebanon	1991	1996	15.0	Lebanon Maternal and Child Health Survey - Summary Report. Beirut: Ministry of Public Health [Lebanon], League of Arab States, Pan Arab Project for Child Development; 1998.
LBN	Lebanon	1999	2000	23.1	National Perinatal Survey 1999-2000. Beirut: Ministry of Public Health [Lebanon], United Nations Children's Fund (UNICEF); 2001.
LBN	Lebanon	1999	2004	23.2	Lebanon Family Health Survey 2004. Principal Report. Beirut: Central Administration of Statistics [Lebanon], The Arab League, The Pan Arab Project for Family Health; 2006.
LBN	Lebanon	2011	2011	46.1	Statistical Bulletin 2011. Beirut: Ministry of Public Health [Lebanon]; 2012.
LBN	Lebanon	2012	2012	46.6	Statistical Bulletin 2012. Beirut: Ministry of Public Health [Lebanon]; 2013.
LBN	Lebanon	2013	2013	45.6	Ministry of Public Health [Lebanon]. Statistical Bulletin 2013. Beirut: World Health Organization (WHO) Lebanon Office; 2014.

ISO Code	Country	Coverage start year	Coverage end year	Caesarean section rate (%)	References
LBN	Lebanon	2014	2014	45.6	Ministry of Public Health [Lebanon]. Statistical Bulletin 2014. Beirut: World Health Organization (WHO) Lebanon Office; 2015.
LBN	Lebanon	2015	2015	46.7	Statistical Bulletin 2015. Beirut: Ministry of Public Health [Lebanon]; 2016.
LBN	Lebanon	2016	2016	47.1	Vital Data Observatory (VDO) statistics by Qada and nationality, 2016 [website]. Beirut: Ministry of Public Health [Lebanon]; 2017 (https://moph.gov.lb/en/Pages/8/327/#/en/Pages/8/14246/vital-data-observatory-statistics, accessed 30 November 2017).
LBN	Lebanon	2017	2017	47.3	Vital Data Observatory (VDO) statistics by Qada and nationality, 2017 [website]. Beirut: Ministry of Public Health [Lebanon]; 2019 (https://moph.gov.lb/en/Pages/8/327/#/en/Pages/8/14246/vital-data-observatory-statistics, accessed 13 February 2019).
LBN	Lebanon	2018	2018	47.8	Vital Data Observatory (VDO) statistics by Qada and nationality, 2018 [website]. Beirut: Ministry of Public Health [Lebanon]; 2020 (https://moph.gov.lb/en/Pages/8/327/#/en/Pages/8/14246/vital-data-observatory-statistics, accessed 5 March 2020).
LSO	Lesotho	2003	2003	9.4	Statistical Yearbook 2010 Kingdom of Lesotho. Statistical Reports No 12:2009. Maseru: Bureau of Statistics [Lesotho]; 2010.
LSO	Lesotho	1999	2004	5.1	Ministry of Health and Social Welfare (MOHSW) [Lesotho], Bureau of Statistics (BOS) [Lesotho], ORC Macro. Lesotho Demographic and Health Survey 2004. Calverton, Maryland: MOH, BOS, ORC Macro; 2005.
LSO	Lesotho	2004	2004	9.5	Statistical Yearbook 2010 Kingdom of Lesotho. Statistical Reports No 12:2009. Maseru: Bureau of Statistics [Lesotho]; 2010.

ISO Code	Country	Coverage start year	Coverage end year	Caesarean section rate (%)	References
LSO	Lesotho	2005	2005	9.0	Statistical Yearbook 2010 Kingdom of Lesotho. Statistical Reports No 12:2009. Maseru: Bureau of Statistics [Lesotho]; 2010.
LSO	Lesotho	2004	2009	6.7	Ministry of Health and Social Welfare (MOHSW) [Lesotho], ICF Macro. Lesotho Demographic and Health Survey 2009. Maseru, Lesotho: MOHSW, ICF Macro; 2010.
LSO	Lesotho	2009	2014	9.7	Ministry of Health [Lesotho], ICF International. Lesotho Demographic and Health Survey 2014. Maseru, Lesotho: Ministry of Health, ICF International; 2016.
LSO	Lesotho	2016	2018	17.4	Lesotho Multiple Indicator Cluster Survey 2018, Survey Findings Report. Maseru: Bureau of Statistics [Lesotho]; 2019.
LBR	Liberia	2002	2007	3.5	Liberia Institute of Statistics and Geo-Information Services (LISGIS) [Liberia], Ministry of Health and Social Welfare [Liberia], National AIDS Control Program [Liberia], Macro International Inc. Liberia Demographic and Health Survey 2007. Monrovia, Liberia: Liberia Institute of Statistics and Geo-Information Services (LISGIS), Macro International Inc.; 2008.
LBR	Liberia	2008	2013	3.9	Liberia Institute of Statistics and Geo-Information Services (LISGIS), Ministry of Health and Social Welfare [Liberia], National AIDS Control Program [Liberia], ICF International. Liberia Demographic and Health Survey 2013. Monrovia, Liberia: Liberia Institute of Statistics and Geo- Information Services (LISGIS), ICF International; 2014.
LTU	Lithuania	1992	1992	8.3	European Health for All Database (HFA-DB) [online database]. World Health Organization (WHO) Regional Office for Europe; 2012 (http://data.euro.who.int/hfadb, accessed 20 August 2012).
LTU	Lithuania	1993	1993	9.3	European Health for All Database (HFA-DB) [online database]. World Health Organization (WHO) Regional Office for Europe; 2012 (http://data.euro.who.int/hfadb, accessed 20 August 2012).

ISO Code	Country	Coverage start year	Coverage end year	Caesarean section rate (%)	References
LTU	Lithuania	1994	1994	10.0	European Health for All Database (HFA-DB) [online database]. World Health Organization (WHO) Regional Office for Europe; 2012 (http://data.euro.who.int/hfadb, accessed 20 August 2012).
LTU	Lithuania	1995	1995	9.6	Institute of Hygiene Health Information Centre, Vilnius University Medical Faculty, Vilnius University Clinic of Children's Diseases, Children's Hospital, Affiliate of Vilnius University Hospital Santariskiu Klinikos Centre of Neonatology. Medical data of Births 2010m. Vilnius: Institute of Hygiene Health Information Centre; 2011.
LTU	Lithuania	1996	1996	10.6	Institute of Hygiene Health Information Centre, Vilnius University Medical Faculty, Vilnius University Clinic of Children's Diseases, Children's Hospital, Affiliate of Vilnius University Hospital Santariskiu Klinikos Centre of Neonatology. Medical data of Births 2010m. Vilnius: Institute of Hygiene Health Information Centre; 2011.
LTU	Lithuania	1997	1997	11.2	Institute of Hygiene Health Information Centre, Vilnius University Medical Faculty, Vilnius University Clinic of Children's Diseases, Children's Hospital, Affiliate of Vilnius University Hospital Santariskiu Klinikos Centre of Neonatology. Medical data of Births 2010m. Vilnius: Institute of Hygiene Health Information Centre; 2011.
LTU	Lithuania	1998	1998	11.7	Institute of Hygiene Health Information Centre, Vilnius University Medical Faculty, Vilnius University Clinic of Children's Diseases, Children's Hospital, Affiliate of Vilnius University Hospital Santariskiu Klinikos Centre of Neonatology. Medical data of Births 2010m. Vilnius: Institute of Hygiene Health Information Centre; 2011.
LTU	Lithuania	1999	1999	12.3	Institute of Hygiene Health Information Centre, Vilnius University Medical Faculty, Vilnius University Clinic of Children's Diseases, Children's Hospital, Affiliate of Vilnius University Hospital Santariskiu Klinikos Centre of Neonatology. Medical data of Births 2010m. Vilnius: Institute of Hygiene Health Information Centre; 2011.
LTU	Lithuania	2000	2000	13.0	Institute of Hygiene Health Information Centre, Vilnius University Medical Faculty, Vilnius University Clinic of Children's Diseases, Children's Hospital, Affiliate of Vilnius University Hospital Santariskiu Klinikos Centre of Neonatology. Medical data of Births 2010m. Vilnius: Institute of Hygiene Health Information Centre; 2011.
LTU	Lithuania	2001	2001	13.5	Institute of Hygiene Health Information Centre, Vilnius University Medical Faculty, Vilnius University Clinic of Children's Diseases, Children's Hospital, Affiliate of Vilnius University Hospital Santariskiu Klinikos Centre of Neonatology. Medical data of Births 2010m. Vilnius: Institute of Hygiene Health Information Centre; 2011.

ISO Code	Country	Coverage start year	Coverage end year	Caesarean section rate (%)	References
LTU	Lithuania	2002	2002	15.3	Institute of Hygiene Health Information Centre, Vilnius University Medical Faculty, Vilnius University Clinic of Children's Diseases, Children's Hospital, Affiliate of Vilnius University Hospital Santariskiu Klinikos Centre of Neonatology. Medical data of Births 2010m. Vilnius: Institute of Hygiene Health Information Centre; 2011.
LTU	Lithuania	2003	2003	15.2	Institute of Hygiene Health Information Centre, Vilnius University Medical Faculty, Vilnius University Clinic of Children's Diseases, Children's Hospital, Affiliate of Vilnius University Hospital Santariskiu Klinikos Centre of Neonatology. Medical data of Births 2010m. Vilnius: Institute of Hygiene Health Information Centre; 2011.
LTU	Lithuania	2004	2004	17.3	Institute of Hygiene Health Information Centre, Vilnius University Medical Faculty, Vilnius University Clinic of Children's Diseases, Children's Hospital, Affiliate of Vilnius University Hospital Santariskiu Klinikos Centre of Neonatology. Medical data of Births 2010m. Vilnius: Institute of Hygiene Health Information Centre; 2011.
LTU	Lithuania	2005	2005	19.1	Institute of Hygiene Health Information Centre, Vilnius University Medical Faculty, Vilnius University Clinic of Children's Diseases, Children's Hospital, Affiliate of Vilnius University Hospital Santariskiu Klinikos Centre of Neonatology. Medical data of Births 2010m. Vilnius: Institute of Hygiene Health Information Centre; 2011.
LTU	Lithuania	2006	2006	20.9	Institute of Hygiene Health Information Centre, Vilnius University Medical Faculty, Vilnius University Clinic of Children's Diseases, Children's Hospital, Affiliate of Vilnius University Hospital Santariskiu Klinikos Centre of Neonatology. Medical data of Births 2010m. Vilnius: Institute of Hygiene Health Information Centre; 2011.
LTU	Lithuania	2007	2007	22.9	Institute of Hygiene Health Information Centre, Vilnius University Medical Faculty, Vilnius University Clinic of Children's Diseases, Children's Hospital, Affiliate of Vilnius University Hospital Santariskiu Klinikos Centre of Neonatology. Medical data of Births 2010m. Vilnius: Institute of Hygiene Health Information Centre; 2011.
LTU	Lithuania	2008	2008	23.8	Institute of Hygiene Health Information Centre, Vilnius University Medical Faculty, Vilnius University Clinic of Children's Diseases, Children's Hospital, Affiliate of Vilnius University Hospital Santariskiu Klinikos Centre of Neonatology. Medical data of Births 2010m. Vilnius: Institute of Hygiene Health Information Centre; 2011.
LTU	Lithuania	2009	2009	24.4	Institute of Hygiene Health Information Centre, Vilnius University Medical Faculty, Vilnius University Clinic of Children's Diseases, Children's Hospital, Affiliate of Vilnius University Hospital Santariskiu Klinikos Centre of Neonatology. Medical data of Births 2010m. Vilnius: Institute of Hygiene Health Information Centre; 2011.

ISO Code	Country	Coverage start year	Coverage end year	Caesarean section rate (%)	References
LTU	Lithuania	2010	2010	25.2	Institute of Hygiene Health Information Centre, Vilnius University Medical Faculty, Vilnius University Clinic of Children's Diseases, Children's Hospital, Affiliate of Vilnius University Hospital Santariskiu Klinikos Centre of Neonatology. Medical data of Births 2010m. Vilnius: Institute of Hygiene Health Information Centre; 2011.
LTU	Lithuania	2011	2011	25.0	Institute of Hygiene Health Information Centre, Vilnius University Medical Faculty, Vilnius University Clinic of Children's Diseases, Children's Hospital, Affiliate of Vilnius University Hospital Santariskiu Klinikos Centre of Neonatology. Medical data of Births 2011 m. Vilnius: Institute of Hygiene Health Information Centre; 2012.
LTU	Lithuania	2012	2012	26.5	Institute of Hygiene Health Information Centre, Vilnius University Medical Faculty, Vilnius University, Center of Neonatology. Medical data of Births 2012 m. Vilnius: Institute of Hygiene Health Information Centre; 2013.
LTU	Lithuania	2013	2013	25.7	Institute of Hygiene Health Information Centre, Vilnius University Medical Faculty, Vilnius University, Center of Neonatology. Medical data of Births 2013 m. Vilnius: Institute of Hygiene Health Information Centre; 2014.
LTU	Lithuania	2014	2014	22.0	Institute of Hygiene Health Information Centre, Vilnius University Medical Faculty, Vilnius University, Center of Neonatology. Medical data of Births 2014 m. Vilnius: Institute of Hygiene Health Information Centre; 2015.
LTU	Lithuania	2015	2015	21.9	Institute of Hygiene Health Information Centre, Vilnius University Medical Faculty, Vilnius University, Center of Neonatology. Medical data of Births 2015 m. Vilnius: Institute of Hygiene Health Information Centre; 2016.
LTU	Lithuania	2016	2016	20.6	Medical data of Births 2016. Vilnius: Institute of Hygiene Health Information Centre, Vilnius University Medical Faculty, Vilnius University, Center of Neonatology; 2017.
LTU	Lithuania	2017	2017	20.2	Medical data of Births 2017. Vilnius: Institute of Hygiene Health Information Centre, Vilnius University Medical Faculty, Vilnius University, Center of Neonatology; 2018.

ISO Code	Country	Coverage start year	Coverage end year	Caesarean section rate (%)	References
LUX	Luxembourg	1997	1997	18.1	European Health for All Database (HFA-DB) [online database]. World Health Organization (WHO) Regional Office for Europe; 2012 (http://data.euro.who.int/hfadb, accessed 20 August 2012).
LUX	Luxembourg	1998	1998	17.7	European Health for All Database (HFA-DB) [online database]. World Health Organization (WHO) Regional Office for Europe; 2012 (http://data.euro.who.int/hfadb, accessed 20 August 2012).
LUX	Luxembourg	1999	1999	18.9	European Health for All Database (HFA-DB) [online database]. World Health Organization (WHO) Regional Office for Europe; 2012 (http://data.euro.who.int/hfadb, accessed 20 August 2012).
LUX	Luxembourg	2000	2000	19.8	European Health for All Database (HFA-DB) [online database]. World Health Organization (WHO) Regional Office for Europe; 2012 (http://data.euro.who.int/hfadb, accessed 20 August 2012).
LUX	Luxembourg	2001	2001	22.0	European Health for All Database (HFA-DB) [online database]. World Health Organization (WHO) Regional Office for Europe; 2012 (http://data.euro.who.int/hfadb, accessed 20 August 2012).
LUX	Luxembourg	2002	2002	23.3	European Health for All Database (HFA-DB) [online database]. World Health Organization (WHO) Regional Office for Europe; 2012 (http://data.euro.who.int/hfadb, accessed 20 August 2012).
LUX	Luxembourg	2004	2004	24.0	European Health for All Database (HFA-DB) [online database]. World Health Organization (WHO) Regional Office for Europe; 2012 (http://data.euro.who.int/hfadb, accessed 20 August 2012).
LUX	Luxembourg	2007	2007	29.2	Indications de la césarienne programmée à terme au Luxembourg – Version longue (Version 1.0). Luxembourg: Secrétariat du Conseil Scientifique; 2014 (in French).

ISO Code	Country	Coverage start year	Coverage end year	Caesarean section rate (%)	References
LUX	Luxembourg	2008	2008	29.9	Indications de la césarienne programmée à terme au Luxembourg – Version longue (Version 1.0). Luxembourg: Secrétariat du Conseil Scientifique; 2014 (in French).
LUX	Luxembourg	2009	2009	28.9	Indications de la césarienne programmée à terme au Luxembourg – Version longue (Version 1.0). Luxembourg: Secrétariat du Conseil Scientifique; 2014 (in French).
LUX	Luxembourg	2010	2010	29.1	Indications de la césarienne programmée à terme au Luxembourg – Version longue (Version 1.0). Luxembourg: Secrétariat du Conseil Scientifique; 2014 (in French).
LUX	Luxembourg	2011	2011	31.0	Surveillance de la santé périnatale au Luxembourg. Évolution de 2001 à 2011. Luxembourg: Ministère de la Santé – Centre de Recherche Public de la Santé [Luxembourg]; 2013 (in French).
LUX	Luxembourg	2012	2012	30.8	Surveillance de la santé périnatale au Luxembourg. 2011 – 2012 – 2013. Luxembourg: Ministère de la Santé [Luxembourg], Luxembourg Institute of Health; 2016 (in French).
LUX	Luxembourg	2013	2013	30.5	Surveillance de la santé périnatale au Luxembourg. 2011 – 2012 – 2013. Luxembourg: Ministère de la Santé [Luxembourg], Luxembourg Institute of Health; 2016 (in French).
LUX	Luxembourg	2014	2014	28.8	European Health Information Gateway. European Health for All database (HFA-DB). Caesarean sections per 1000 live births [online database]. World Health Organization (WHO) Regional Office for Europe; 2018 (https://gateway.euro.who.int/en/indicators/hfa_596-7060-caesarean-sections-per-1000-live-births/, accessed 14 February 2019).
LUX	Luxembourg	2015	2015	32.7	European Perinatal Health Report. Core indicators of the health and care of pregnant women and babies in Europe in 2015. Euro-Peristat Project; 2018.

ISO Code	Country	Coverage start year	Coverage end year	Caesarean section rate (%)	References
MDG	Madagascar	1987	1992	1.0	Refeno G, Rabeza V, Mboup G, Schoemaker J (Centre National de Recherches sur l'Environnement [Madagascar], Macro International). Madagascar Enquête Nationale Démographique et Sanitaire 1992. Calverton, Maryland, USA: Centre National de Recherches sur l'Environnement [Madagascar], Macro International; 1994 (in French).
MDG	Madagascar	1992	1997	0.6	Direction de la Démographie et des Statistiques Sociales, Institut National de la Statistique (INSTAT) [Madagascar], Macro International Inc. Enquête Démographique et de Santé, Madagascar 1997. Calverton, Maryland, USA: INSTAT, Macro International Inc.; 1998 (in French).
MDG	Madagascar	1998	2004	1.0	Institut National de la Statistique (INSTAT), ORC Macro. Enquête Démographique et de Santé de Madagascar 2003-2004. Calverton, Maryland, USA: INSTAT, ORC Macro; 2005 (in French).
MDG	Madagascar	2003	2009	1.5	Institut National de la Statistique (INSTAT), ICF Macro. Enquête Démographique et de Santé de Madagascar 2008-2009. Antananarivo, Madagascar: INSTAT, ICF Macro; 2010 (in French).
MDG	Madagascar	2007	2013	1.9	L'Enquête Nationale sur le Suivi des Objectifs du Millénaire pour le Développement à Madagascar 2012-2013 (ENSOMD). Madagascar: l'Institut National de la Statistique [Madagascar], l'Office National de Nutrition (ONN), Fonds des Nations Unies pour l'Enfance (UNICEF), Fonds des Nations Unies pour la Population (FNUAP/UNFPA), Programme des Nations Unies pour le développement (PNUD/UNDP); 2014 (in French).
MDG	Madagascar	2016	2018	2.0	Enquête A Indicateurs Multiples MICS 2018, Snapshot. Madagascar: Institut National De La Statistique (INSTAT) [Madagascar], Fonds des Nations Unies pour l'Enfance (UNICEF); 2019 (in French).
MWI	Malawi	1987	1992	3.4	National Statistical Office, Macro International Inc. Malawi Demographic and Health Survey 1992. Calverton, Maryland USA: Macro International Inc.; 1994.
MWI	Malawi	1995	2000	2.8	National Statistical Office [Malawi], ORC Macro. Malawi Demographic and Health Survey 2000. Zomba, Malawi, Calverton, Maryland, USA: National Statistical Office, ORC Macro; 2001.

ISO Code	Country	Coverage start year	Coverage end year	Caesarean section rate (%)	References
MWI	Malawi	1999	2004	3.1	National Statistical Office (NSO) [Malawi], ORC Macro. Malawi Demographic and Health Survey 2004. Calverton, Maryland, USA: NSO, ORC Macro; 2005.
MWI	Malawi	2005	2010	4.6	National Statistical Office (NSO), ICF Macro. Malawi Demographic and Health Survey 2010. Zomba, Malawi, Calverton, Maryland, USA: NSO, ICF Macro; 2011.
MWI	Malawi	2011	2014	5.1	National Statistical Office, United Nations Children's Fund (UNICEF). Malawi MDG Endline Survey 2014. Zomba, Malawi: National Statistical Office, United Nations Children's Fund (UNICEF); 2015.
MWI	Malawi	2010	2016	6.1	National Statistical Office (NSO) [Malawi], ICF. Malawi Demographic and Health Survey 2015-16. Zomba, Malawi: NSO, ICF; 2017.
MYS	Malaysia	2000	2000	10.5	Ravindran J. Rising Caesarean Section Rates in Public Hospitals in Malaysia 2006. Med J Malaysia. 2008;63(5):434-5.
MYS	Malaysia	2001	2001	11.1	Ravindran J. Rising Caesarean Section Rates in Public Hospitals in Malaysia 2006. Med J Malaysia. 2008;63(5):434-5.
MYS	Malaysia	2006	2006	15.7	Ravindran J. Rising Caesarean Section Rates in Public Hospitals in Malaysia 2006. Med J Malaysia. 2008;63(5):434-5.
MDV	Maldives	2008	2008	34.1	The Maldives Health Statistics 2011. Male': Ministry of Health and Family [Republic of Maldives]; 2012.

ISO Code	Country	Coverage start year	Coverage end year	Caesarean section rate (%)	References
MDV	Maldives	2004	2009	32.4	Ministry of Health and Family (MOHF) [Maldives], ICF Macro. Maldives Demographic and Health Survey 2009. Calverton, Maryland: MOHF, ICF Macro; 2010.
MDV	Maldives	2009	2009	36.7	The Maldives Health Statistics 2011. Male': Ministry of Health and Family [Republic of Maldives]; 2012.
MDV	Maldives	2010	2010	26.6	The Maldives Health Statistics 2011. Male': Ministry of Health and Family [Republic of Maldives]; 2012.
MDV	Maldives	2011	2011	41.1	The Maldives Health Statistics 2012. Male': Ministry of Health [Republic of Maldives]; 2013.
MDV	Maldives	2012	2012	40.8	The Maldives Health Statistics 2013. Male': Ministry of Health [Republic of Maldives]; 2014.
MDV	Maldives	2011	2017	40.0	Maldives Demographic and Health Survey 2016-2017. Malé, Rockville, Maryland: Ministry of Health (MOH) [Maldives], ICF; 208.
MLI	Mali	1992	1996	0.8	Coulibaly S, Dicko F, Traoré SM, Sidibé O, Seroussi M, Barrère B (Cellule de Planification et de Statistique du Ministère de la Santé, Direction Nationale de la Statistique et de l'Informatique, Macro International Inc.). Enquête Démographique et de Santé, Mali 1995-1996. Calverton, Maryland, USA: Cellule de Planification et de Statistique du Ministère de la Santé, Direction Nationale de la Statistique et de l'Informatique, Macro International Inc.; 1996 (in French).
MLI	Mali	1996	2001	1.1	Cellule de Planification et de Statistique du Ministère de la Santé (CPS/MS), Direction Nationale de la Statistique et de l'Informatique (DNSI), ORC Macro. Enquête Démographique et de Santé au Mali 2001. Calverton, Maryland, USA: CPS/MS, DNSI, ORC Macro; 2002 (in French).

ISO Code	Country	Coverage start year	Coverage end year	Caesarean section rate (%)	References
MLI	Mali	2001	2006	1.6	Cellule de Planification et de Statistique du Ministère de la Santé (CPS/MS), Direction Nationale de la Statistique et de l'Informatique du Ministère de l'Économie, de l'Industrie et du Commerce (DNSI/MEIC), Macro International Inc. Enquête Démographique et de Santé du Mali 2006. Calverton, Maryland, USA: CPS/DNSI, Macro International Inc.; 2007 (in French).
MLI	Mali	2007	2013	2.7	Cellule de Planification et de Statistique (CPS/SSDSPF), Institut National de la Statistique (INSTAT/MPATP), INFO-STAT, ICF International. Enquête Démographique et de Santé au Mali 2012-2013. Rockville, Maryland, USA: CPS, INSTAT, INFO-STAT, ICF International; 2014 (in French).
MLI	Mali	2013	2015	2.0	Institut national de la statistique (INSTAT), Fonds des Nations Unies pour l'enfance (UNICEF). Enquête par grappes à indicateurs multiples au Mali (MICS-Mali), 2015, Rapport final. Bamako, Mali: INSTAT; 2016 (in French).
MLI	Mali	2013	2018	2.5	Sixième Enquête Démographique et de Santé au Mali 2018. Bamako, Rockville, Maryland: Institut National de la Statistique (INSTAT), Cellule de Planification et de Statistique Secteur Santé-Développement Social et Promotion de la Famille (CPS/SS-DS-PF), ICF; 2019.
MLT	Malta	1995	1995	16.4	National Obstetric Information System (NOIS). National Birth Statistics in Review: 1995 to 2001. NOIS Annual Report – 2001. Malta: Department of Health Information (DHI); 2002.
MLT	Malta	1996	1996	17.0	National Obstetric Information System (NOIS). National Birth Statistics in Review: 1995 to 2001. NOIS Annual Report – 2001. Malta: Department of Health Information (DHI); 2002.
MLT	Malta	1997	1997	19.0	National Obstetric Information System (NOIS). National Birth Statistics in Review: 1995 to 2001. NOIS Annual Report – 2001. Malta: Department of Health Information (DHI); 2002.
MLT	Malta	1998	1998	21.0	National Obstetric Information System (NOIS). National Birth Statistics in Review: 1995 to 2001. NOIS Annual Report – 2001. Malta: Department of Health Information (DHI); 2002.

ISO Code	Country	Coverage start year	Coverage end year	Caesarean section rate (%)	References
MLT	Malta	1999	1999	22.1	National Obstetric Information System (NOIS). Annual Report – 2008. Malta: Department of Health Information and Research (DHIR); 2009.
MLT	Malta	2000	2000	23.1	National Obstetric Information System (NOIS). Annual Report – 2008. Malta: Department of Health Information and Research (DHIR); 2009.
MLT	Malta	2001	2001	23.6	National Obstetric Information System (NOIS). Annual Report – 2008. Malta: Department of Health Information and Research (DHIR); 2009.
MLT	Malta	2002	2002	23.6	National Obstetric Information System (NOIS). Annual Report – 2008. Malta: Department of Health Information and Research (DHIR); 2009.
MLT	Malta	2003	2003	26.0	National Obstetric Information System (NOIS). Annual Report – 2008. Malta: Department of Health Information and Research (DHIR); 2009.
MLT	Malta	2004	2004	27.3	National Obstetric Information System (NOIS). Annual Report – 2008. Malta: Department of Health Information and Research (DHIR); 2009.
MLT	Malta	2005	2005	30.6	National Obstetric Information System (NOIS). Annual Report – 2008. Malta: Department of Health Information and Research (DHIR); 2009.
MLT	Malta	2006	2006	34.8	National Obstetric Information System (NOIS). Annual Report – 2008. Malta: Department of Health Information and Research (DHIR); 2009.

Betran AP, *et al. BMJ Global Health* 2021; 6:e005671. doi: 10.1136/bmjgh-2021-005671

ISO Code	Country	Coverage start year	Coverage end year	Caesarean section rate (%)	References
MLT	Malta	2007	2007	32.3	National Obstetric Information System (NOIS). Annual Report – 2008. Malta: Department of Health Information and Research (DHIR); 2009.
MLT	Malta	2008	2008	30.4	National Obstetric Information System (NOIS). Annual Report – 2008. Malta: Department of Health Information and Research (DHIR); 2009.
MLT	Malta	2009	2009	29.0	National Obstetric Information System (NOIS). Annual Report – 2009. Malta: Department of Health Information and Research (DHIR); 2010.
MLT	Malta	2010	2010	31.7	National Obstetric Information System (NOIS). Annual Report – 2010. Malta: Department of Health Information and Research (DHIR); 2011.
MLT	Malta	2011	2011	34.0	National Obstetric Information System (NOIS). Annual Report – 2011. Malta: Department of Health Information and Research (DHIR); 2012.
MLT	Malta	2012	2012	33.7	National Obstetric Information System (NOIS). Annual Report – 2012. Malta: Department of Health Information and Research (DHIR); 2013.
MLT	Malta	2013	2013	31.2	National Obstetric Information System (NOIS). Annual Report – 2013. Malta: Directorate for Health Information and Research (DHIR); 2014.
MLT	Malta	2014	2014	32.0	National Obstetric Information System (NOIS). Annual Report – 2014. Malta: Directorate for Health Information and Research (DHIR); 2015.

ISO Code	Country	Coverage start year	Coverage end year	Caesarean section rate (%)	References
MLT	Malta	2015	2015	31.0	National Obstetric Information System (NOIS). Annual Report – 2016. Malta: Directorate for Health Information and Research (DHIR); 2017.
MLT	Malta	2016	2016	30.7	National Obstetric Information System (NOIS). Annual Report – 2016. Malta: Directorate for Health Information and Research (DHIR); 2017.
MLT	Malta	2017	2017	30.9	National Obstetric Information System (NOIS). Annual Report – 2017. Malta: Directorate for Health Information and Research (DHIR); 2018.
MHL	Marshall Islands	2002	2007	9.3	Economic Policy, Planning and Statistics Office (EPPSO) [Marshall Islands], Secretariat of the Pacific Community (SPC), Macro International Inc. Republic of the Marshall Islands Demographic and Health Survey 2007. Noumea: SPC; 2008.
MHL	Marshall Islands	2015	2017	9.7	Republic of the Marshall Islands Ministry of Health and Human Services, RMI Economic, Policy Planning and Statistics Office, UNICEF. Republic of the Marshall Islands Integrated Child Health and Nutrition Survey 2017, Final Report. Majuro: Republic of the Marshall Islands Ministry of Health and Human Services, RMI Economic, Policy Planning and Statistics Office; 2017.
MRT	Mauritania	1995	2001	3.2	Office National de la Statistique (ONS) [Mauritanie],ORC Macro. Enquête Démographique et de Santé Mauritanie 2000-2001. Calverton, Maryland, USA: ONS, ORC Macro; 2001 (in French).
MRT	Mauritania	2009	2011	9.6	Mauritanie Enquête Par Grappes À Indicateurs Multiples MICS 2011. Rapport Final. Nouakchott: Office National De La Statistique [Mauritanie], Fonds des Nations unies pour l'enfance (UNICEF), Fonds des Nations Unies pour la Population (FNUAP/UNFPA); 2014 (in French).
MRT	Mauritania	2013	2015	4.9	Mauritanie Enquête Par Grappes À Indicateurs Multiples MICS 2015. Rapport Final. Nouakchott: Ministère De l'Économie et des Finances, Office National De La Statistique [Mauritanie], Fonds des Nations unies pour l'enfance (UNICEF); 2017 (in French).

ISO Code	Country	Coverage start year	Coverage end year	Caesarean section rate (%)	References
MUS	Mauritius	1990	1990	12.8	2000 Housing and Population Census - Republic of Mauritius. Analysis Report. Volume VI – Health and Quality of Life, Morbidity and Mortality. Port Louis: Central Statistics Office, Ministry of Finance and Economic Development; 2004.
MUS	Mauritius	1991	1991	13.9	2000 Housing and Population Census - Republic of Mauritius. Analysis Report. Volume VI – Health and Quality of Life, Morbidity and Mortality. Port Louis: Central Statistics Office, Ministry of Finance and Economic Development; 2004.
MUS	Mauritius	1992	1992	17.0	2000 Housing and Population Census - Republic of Mauritius. Analysis Report. Volume VI – Health and Quality of Life, Morbidity and Mortality. Port Louis: Central Statistics Office, Ministry of Finance and Economic Development; 2004.
MUS	Mauritius	1993	1993	17.4	2000 Housing and Population Census - Republic of Mauritius. Analysis Report. Volume VI – Health and Quality of Life, Morbidity and Mortality. Port Louis: Central Statistics Office, Ministry of Finance and Economic Development; 2004.
MUS	Mauritius	1994	1994	18.3	2000 Housing and Population Census - Republic of Mauritius. Analysis Report. Volume VI – Health and Quality of Life, Morbidity and Mortality. Port Louis: Central Statistics Office, Ministry of Finance and Economic Development; 2004.
MUS	Mauritius	1995	1995	20.1	2000 Housing and Population Census - Republic of Mauritius. Analysis Report. Volume VI – Health and Quality of Life, Morbidity and Mortality. Port Louis: Central Statistics Office, Ministry of Finance and Economic Development; 2004.
MUS	Mauritius	1996	1996	20.5	2000 Housing and Population Census - Republic of Mauritius. Analysis Report. Volume VI – Health and Quality of Life, Morbidity and Mortality. Port Louis: Central Statistics Office, Ministry of Finance and Economic Development; 2004.
MUS	Mauritius	1997	1997	22.4	2000 Housing and Population Census - Republic of Mauritius. Analysis Report. Volume VI – Health and Quality of Life, Morbidity and Mortality. Port Louis: Central Statistics Office, Ministry of Finance and Economic Development; 2004.

ISO Code	Country	Coverage start year	Coverage end year	Caesarean section rate (%)	References
MUS	Mauritius	1998	1998	22.7	2000 Housing and Population Census - Republic of Mauritius. Analysis Report. Volume VI – Health and Quality of Life, Morbidity and Mortality. Port Louis: Central Statistics Office, Ministry of Finance and Economic Development; 2004.
MUS	Mauritius	1999	1999	25.1	2000 Housing and Population Census - Republic of Mauritius. Analysis Report. Volume VI – Health and Quality of Life, Morbidity and Mortality. Port Louis: Central Statistics Office, Ministry of Finance and Economic Development; 2004.
MUS	Mauritius	2000	2000	28.0	Health Statistics Report 2014. Island of Mauritius and Island of Rodrigues. Port Louis: Health Statistics Unit, Ministry of Health and Quality of Life [Mauritius]; 2015.
MUS	Mauritius	2001	2001	29.8	Health Statistics Report 2014. Island of Mauritius and Island of Rodrigues. Port Louis: Health Statistics Unit, Ministry of Health and Quality of Life [Mauritius]; 2015.
MUS	Mauritius	2002	2002	30.4	Health Statistics Report 2014. Island of Mauritius and Island of Rodrigues. Port Louis: Health Statistics Unit, Ministry of Health and Quality of Life [Mauritius]; 2015.
MUS	Mauritius	2003	2003	31.7	Health Statistics Report 2017. Island of Mauritius and Island of Rodrigues. Port Louis: Health Statistics Unit, Ministry of Health and Quality of Life [Mauritius]; 2018.
MUS	Mauritius	2004	2004	34.7	Health Statistics Report 2017. Island of Mauritius and Island of Rodrigues. Port Louis: Health Statistics Unit, Ministry of Health and Quality of Life [Mauritius]; 2018.
MUS	Mauritius	2005	2005	34.3	Health Statistics Report 2017. Island of Mauritius and Island of Rodrigues. Port Louis: Health Statistics Unit, Ministry of Health and Quality of Life [Mauritius]; 2018.

ISO Code	Country	Coverage start year	Coverage end year	Caesarean section rate (%)	References
MUS	Mauritius	2006	2006	35.2	Health Statistics Report 2017. Island of Mauritius and Island of Rodrigues. Port Louis: Health Statistics Unit, Ministry of Health and Quality of Life [Mauritius]; 2018.
MUS	Mauritius	2007	2007	39.1	Health Statistics Report 2017. Island of Mauritius and Island of Rodrigues. Port Louis: Health Statistics Unit, Ministry of Health and Quality of Life [Mauritius]; 2018.
MUS	Mauritius	2008	2008	40.4	Health Statistics Report 2017. Island of Mauritius and Island of Rodrigues. Port Louis: Health Statistics Unit, Ministry of Health and Quality of Life [Mauritius]; 2018.
MUS	Mauritius	2009	2009	41.6	Health Statistics Report 2017. Island of Mauritius and Island of Rodrigues. Port Louis: Health Statistics Unit, Ministry of Health and Quality of Life [Mauritius]; 2018.
MUS	Mauritius	2010	2010	43.0	Health Statistics Report 2017. Island of Mauritius and Island of Rodrigues. Port Louis: Health Statistics Unit, Ministry of Health and Quality of Life [Mauritius]; 2018.
MUS	Mauritius	2011	2011	43.9	Health Statistics Report 2017. Island of Mauritius and Island of Rodrigues. Port Louis: Health Statistics Unit, Ministry of Health and Quality of Life [Mauritius]; 2018.
MUS	Mauritius	2012	2012	43.8	Health Statistics Report 2017. Island of Mauritius and Island of Rodrigues. Port Louis: Health Statistics Unit, Ministry of Health and Quality of Life [Mauritius]; 2018.
MUS	Mauritius	2013	2013	45.3	Health Statistics Report 2017. Island of Mauritius and Island of Rodrigues. Port Louis: Health Statistics Unit, Ministry of Health and Quality of Life [Mauritius]; 2018.

ISO Code	Country	Coverage start year	Coverage end year	Caesarean section rate (%)	References
MUS	Mauritius	2014	2014	49.2	Health Statistics Report 2017. Island of Mauritius and Island of Rodrigues. Port Louis: Health Statistics Unit, Ministry of Health and Quality of Life [Mauritius]; 2018.
MUS	Mauritius	2015	2015	49.3	Health Statistics Report 2017. Island of Mauritius and Island of Rodrigues. Port Louis: Health Statistics Unit, Ministry of Health and Quality of Life [Mauritius]; 2018.
MUS	Mauritius	2016	2016	48.7	Health Statistics Report 2017. Island of Mauritius and Island of Rodrigues. Port Louis: Health Statistics Unit, Ministry of Health and Quality of Life [Mauritius]; 2018.
MUS	Mauritius	2017	2017	50.7	Health Statistics Report 2017. Island of Mauritius and Island of Rodrigues. Port Louis: Health Statistics Unit, Ministry of Health and Quality of Life [Mauritius]; 2018.
MEX	Mexico	1996	1996	24.1	Síntesis Ejecutiva. Nacimientos por cesárea en México. México D.F.: Secretaria de Salud; 2000 (in Spanish).
MEX	Mexico	1999	1999	35.4	Síntesis Ejecutiva. Nacimientos por cesárea en México. México D.F.: Secretaria de Salud; 2000 (in Spanish).
MEX	Mexico	2002	2002	33.6	Sistema Nacional de Información en Salud - SINAIS. Boletín de Información Estadística. Volumen III. Servicios otorgados y Programas sustantivos. Número 22, Año 2002. México D.F.: Secretaria de Salud [Mexico]; 2003 (in Spanish).
MEX	Mexico	1998	2003	33.2	La salud reproductiva en México. Análisis de la Encuesta Nacional de Salud Reproductiva 2003. México D.F.: Secretaría de Salud [Mexico], Universidad Nacional Autónoma de México - Centro Regional de Investigaciones Multidisciplinarias; 2007 (in Spanish).

ISO Code	Country	Coverage start year	Coverage end year	Caesarean section rate (%)	References
MEX	Mexico	2005	2005	36.1	Sistema Nacional de Información en Salud - SINAIS. Boletín de Información Estadística. Volumen III. Servicios otorgados y Programas sustantivos. Número 25, Año 2005. México D.F.: Secretaria de Salud [Mexico]; 2006 (in Spanish).
MEX	Mexico	2001	2006	37.6	Shamah-Levy T, Villalpando-Hernández S, Rivera-Dommarco JA (Instituto Nacional de Salud Pública [Mexico]). Resultados de Nutrición de la ENSANUT 2006. Cuernavaca: Instituto Nacional de Salud Pública; 2007 (in Spanish).
MEX	Mexico	2004	2009	43.1	Encuesta Nacional de la Dinámica Demográfica 2009. Panorama sociodemográfico de México. Principales resultados. Aguascalientes: Instituto Nacional de Estadística y Geografía (INEGI) [Mexico]; 2011 (in Spanish).
MEX	Mexico	2007	2012	46.2	Gutiérrez JP, Rivera-Dommarco J, Shamah-Levy T, Villalpando-Hernández S, Franco A, Cuevas-Nasu L, Romero-Martínez M, Hernández-Ávila M. (Instituto Nacional de Salud Pública [Mexico]). Encuesta Nacional de Salud y Nutrición 2012. Resultados Nacionales. Cuernavaca: Instituto Nacional de Salud Pública [Mexico]; 2012 (in Spanish).
MEX	Mexico	2009	2014	46.3	Encuesta Nacional de la Dinámica Demográfica ENADID 2014. Principales resultados. Aguascalientes: Instituto Nacional de Estadística y Geografía (INEGI), Consejo Nacional de Población (CONAPO); 2015 (in Spanish).
MEX	Mexico	2013	2015	40.7	Instituto Nacional de Salud Pública, Fondo de las Naciones Unidas para la Infancia (UNICEF) México. Encuesta Nacional de Niños, Niñas y Mujeres 2015 - Encuesta de Indicadores Múltiples por Conglomerados 2015, Informe Final. Ciudad de México, México: Instituto Nacional de Salud Pública, UNICEF México; 2016 (in Spanish).
FSM	Micronesia (Federated States of)	2000	2000	9.6	Statistical Yearbook Federated States of Micronesia 2008. Palikir, Pohnpei: Division of Statistics, Office of Statistics, Budget and Economic Management, Overseas Development Assistance & Compact Management (SBOC) [Federated States of Micronesia]; 2008.
FSM	Micronesia (Federated States of)	2001	2001	11.2	Statistical Yearbook Federated States of Micronesia 2008. Palikir, Pohnpei: Division of Statistics, Office of Statistics, Budget and Economic Management, Overseas Development Assistance & Compact Management (SBOC) [Federated States of Micronesia]; 2008.

ISO Code	Country	Coverage start year	Coverage end year	Caesarean section rate (%)	References
FSM	Micronesia (Federated States of)	2002	2002	10.9	Statistical Yearbook Federated States of Micronesia 2008. Palikir, Pohnpei: Division of Statistics, Office of Statistics, Budget and Economic Management, Overseas Development Assistance & Compact Management (SBOC) [Federated States of Micronesia]; 2008.
FSM	Micronesia (Federated States of)	2003	2003	10.5	Statistical Yearbook Federated States of Micronesia 2008. Palikir, Pohnpei: Division of Statistics, Office of Statistics, Budget and Economic Management, Overseas Development Assistance & Compact Management (SBOC) [Federated States of Micronesia]; 2008.
FSM	Micronesia (Federated States of)	2004	2004	10.5	Statistical Yearbook Federated States of Micronesia 2008. Palikir, Pohnpei: Division of Statistics, Office of Statistics, Budget and Economic Management, Overseas Development Assistance & Compact Management (SBOC) [Federated States of Micronesia]; 2008.
FSM	Micronesia (Federated States of)	2005	2005	8.4	Statistical Yearbook Federated States of Micronesia 2008. Palikir, Pohnpei: Division of Statistics, Office of Statistics, Budget and Economic Management, Overseas Development Assistance & Compact Management (SBOC) [Federated States of Micronesia]; 2008.
FSM	Micronesia (Federated States of)	2006	2006	10.5	Statistical Yearbook Federated States of Micronesia 2008. Palikir, Pohnpei: Division of Statistics, Office of Statistics, Budget and Economic Management, Overseas Development Assistance & Compact Management (SBOC) [Federated States of Micronesia]; 2008.
MCO	Monaco	2012	2012	17.4	European Health for All Database (HFA-DB) [online database]. World Health Organization (WHO) Regional Office for Europe; 2016 (http://data.euro.who.int/hfadb, accessed 8 August 2016).
MCO	Monaco	2015	2015	20.6	European Health for All Database (HFA-DB) [online database]. World Health Organization (WHO) Regional Office for Europe; 2016 (http://data.euro.who.int/hfadb, accessed 8 August 2016).
MCO	Monaco	2016	2016	15.6	European Health Information Gateway. European Health for All database (HFA-DB). Caesarean sections per 1000 live births [online database]. World Health Organization (WHO) Regional Office for Europe; 2018 (https://gateway.euro.who.int/en/indicators/hfa_596-7060-caesarean-sections-per-1000-live-births/, accessed 14 February 2019).

ISO Code	Country	Coverage start year	Coverage end year	Caesarean section rate (%)	References
MNG	Mongolia	1993	1998	5.1	National Statistical Office of Mongolia, Ministry of Health [Mongolia], United Nations Population Fund (UNFPA). Mongolia Reproductive Health Survey 2008. National Report. Ulaanbaatar: National Statistical Office of Mongolia; 2009.
MNG	Mongolia	1998	2003	9.6	National Statistical Office of Mongolia, Ministry of Health [Mongolia], United Nations Population Fund (UNFPA). Mongolia National Report. Reproductive Health Survey 2003. Ulaanbaatar: National Statistical Office of Mongolia; 2004.
MNG	Mongolia	2003	2008	17.4	National Statistical Office of Mongolia, Ministry of Health [Mongolia], United Nations Population Fund (UNFPA). Mongolia Reproductive Health Survey 2008. National Report. Ulaanbaatar: National Statistical Office of Mongolia; 2009.
MNG	Mongolia	2008	2010	21.0	Mongolia "Child Development - 2010" survey. Multiple Indicator Cluster Survey - 4. Final Report. Ulaanbaatar: National Statistical Office of Mongolia, United Nations Children's Fund (UNICEF); 2013.
MNG	Mongolia	2011	2013	23.4	Mongolia Social Indicator Sample Survey - 2013. Multiple Indicator Cluster Survey. Final Report. Ulaanbaatar: National Statistical Office of Mongolia, United Nations Children's Fund (UNICEF), United Nations Population Fund (UNFPA); 2015.
MNG	Mongolia	2016	2018	26.2	Mongolia Social Indicator Sample Survey - 2018. (Multiple Indicator Cluster Survey). Survey Findings Report. Ulaanbaatar: National Statistical Office of Mongolia; 2019.
MNE	Montenegro	1997	1997	9.0	European Health for All Database (HFA-DB) [online database]. World Health Organization (WHO) Regional Office for Europe; 2012 (http://data.euro.who.int/hfadb, accessed 20 August 2012).
MNE	Montenegro	1998	1998	10.2	European Health for All Database (HFA-DB) [online database]. World Health Organization (WHO) Regional Office for Europe; 2012 (http://data.euro.who.int/hfadb, accessed 20 August 2012).

ISO Code	Country	Coverage start year	Coverage end year	Caesarean section rate (%)	References
MNE	Montenegro	1999	1999	10.0	European Health for All Database (HFA-DB) [online database]. World Health Organization (WHO) Regional Office for Europe; 2012 (http://data.euro.who.int/hfadb, accessed 20 August 2012).
MNE	Montenegro	2000	2000	9.9	European Health for All Database (HFA-DB) [online database]. World Health Organization (WHO) Regional Office for Europe; 2012 (http://data.euro.who.int/hfadb, accessed 20 August 2012).
MNE	Montenegro	2001	2001	14.2	European Health for All Database (HFA-DB) [online database]. World Health Organization (WHO) Regional Office for Europe; 2012 (http://data.euro.who.int/hfadb, accessed 20 August 2012).
MNE	Montenegro	2002	2002	12.8	European Health for All Database (HFA-DB) [online database]. World Health Organization (WHO) Regional Office for Europe; 2012 (http://data.euro.who.int/hfadb, accessed 20 August 2012).
MNE	Montenegro	2003	2003	15.1	European Health for All Database (HFA-DB) [online database]. World Health Organization (WHO) Regional Office for Europe; 2012 (http://data.euro.who.int/hfadb, accessed 20 August 2012).
MNE	Montenegro	2007	2007	12.0	Statisticki godisnjak 2007. Health Statistical Yearbook 2007 of Montenegro. Podgorica: Institut Za Javno Zdravlje [Institute of Public Health, Montenegro]; 2008.
MNE	Montenegro	2008	2008	13.1	Statisticki godisnjak 2008. Health Statistical Yearbook 2008 of Montenegro. Podgorica: Institut Za Javno Zdravlje [Institute of Public Health, Montenegro]; 2009.
MNE	Montenegro	2009	2009	19.3	Statisticki godisnjak 2009. Health Statistical Yearbook 2009 of Montenegro. Podgorica: Institut Za Javno Zdravlje [Institute of Public Health, Montenegro]; 2010.

ISO Code	Country	Coverage start year	Coverage end year	Caesarean section rate (%)	References
MNE	Montenegro	2001	2010	23.6	European Health for All Database (HFA-DB) [online database]. World Health Organization (WHO) Regional Office for Europe; 2012 (http://data.euro.who.int/hfadb, accessed 20 August 2012).
MNE	Montenegro	2011	2013	19.9	Statistical Office of Montenegro (MONSTAT), United Nations Children's Fund (UNICEF). 2013 Montenegro Multiple Indicator Cluster Survey and 2013 Montenegro Roma Settlements Multiple Indicator Cluster Survey, Final Report. Podgorica, Montenegro: Statistical Office of Montenegro (MONSTAT), UNICEF; 2014.
MNE	Montenegro	2016	2018	24.4	2018 Montenegro Multiple Indicator Cluster Survey and 2018 Montenegro Roma Settlements Multiple Indicator Cluster Survey, Survey Fndings Report. Podgorica: Statistical Office of Montenegro (MONSTAT), UNICEF; 2019.
MAR	Morocco	1987	1992	2.0	Ministère de la Santé Publique, Macro International Inc. Enquête Nationale sur la Population et la Santé (ENPS-II) 1992. Columbia, Maryland, USA: Macro International Inc.; 1993 (in French).
MAR	Morocco	1990	1995	3.5	Azelmat M, Ayad M, Housni EA. Enquête de Panel sur la Population et la Santé (EPPS) 1995. Calverton, Maryland, USA: Ministère de la Santé Publique, Direction de la Planification et des Ressources Financières, Service des Etudes et de l'Information Sanitaire, Macro International Inc.; 1996 (in French).
MAR	Morocco	1993	1997	3.1	Ministère de la Santé [Morocco], Service des études et de l'information sanitaire (SEIS), Ligue des États Arabes, PAPCHILD (Projet arabe pour la promotion de l'enfance). Enquête Nationale sur la Santé de la Mère et de l 'Enfant (ENSME) 1997. Rabat: Ministère de la Santé [Morocco]; 1999 (in French).
MAR	Morocco	1998	2004	5.4	Ministère de la Santé [Maroc], ORC Macro, Ligue des États Arabes. Enquête sur la Population et la Santé Familiale (EPSF) 2003-2004. Calverton, Maryland, USA: Ministère de la Santé, ORC Macro; 2005 (in French).
MAR	Morocco	2004	2004	3.8	Santé en chiffres 2010. Rabat: Ministère de la Santé - Direction de la Planification et des Ressources Financières/Division de la Planification et des Etudes/Service des Etudes et de l'Information Sanitaire (DPRF/DPE/SEIS); 2011 (in French).

ISO Code	Country	Coverage start year	Coverage end year	Caesarean section rate (%)	References
MAR	Morocco	2005	2005	4.4	Santé en chiffres 2010. Rabat: Ministère de la Santé - Direction de la Planification et des Ressources Financières/Division de la Planification et des Etudes/Service des Etudes et de l'Information Sanitaire (DPRF/DPE/SEIS); 2011 (in French).
MAR	Morocco	2006	2006	4.9	Santé en chiffres 2010. Rabat: Ministère de la Santé - Direction de la Planification et des Ressources Financières/Division de la Planification et des Etudes/Service des Etudes et de l'Information Sanitaire (DPRF/DPE/SEIS); 2011 (in French).
MAR	Morocco	2007	2007	5.6	Santé en chiffres 2010. Rabat: Ministère de la Santé - Direction de la Planification et des Ressources Financières/Division de la Planification et des Etudes/Service des Etudes et de l'Information Sanitaire (DPRF/DPE/SEIS); 2011 (in French).
MAR	Morocco	2010	2010	7.5	Santé en chiffres 2010. Rabat: Ministère de la Santé - Direction de la Planification et des Ressources Financières/Division de la Planification et des Etudes/Service des Etudes et de l'Information Sanitaire (DPRF/DPE/SEIS); 2011 (in French).
MAR	Morocco	2006	2011	16.0	Enquête Nationale sur la Population et la Santé Familiale (ENPSF) 2011. Rabat, Le Caire: Ministère de la Santé [Morocco], Projet PAPFAM, Ligue des États Arabes; 2012 (in French).
MAR	Morocco	2011	2011	12.3	Santé en chiffres 2011. Édition 2012. Rabat: Ministère de la Santé - Direction de la Planification et des Ressources Financières/Division de la Planification et des Etudes/Service des Etudes et de l'Information Sanitaire (DPRF/DPE/SEIS); 2012 (in French).
MAR	Morocco	2012	2012	13.5	Santé en chiffres 2012. Édition 2013. Rabat: Ministère de la Santé - Direction de la Planification et des Ressources Financières/Division de la Planification et des Etudes/Service des Etudes et de l'Information Sanitaire (DPRF/DPE/SEIS); 2013 (in French).
MAR	Morocco	2013	2013	13.9	Santé en chiffres 2013. Édition 2014. Rabat: Ministère de la Santé - Direction de la Planification et des Ressources Financières/Division de la Planification et des Etudes/Service des Etudes et de l'Information Sanitaire (DPRF/DPE/SEIS); 2014 (in French).

ISO Code	Country	Coverage start year	Coverage end year	Caesarean section rate (%)	References
MAR	Morocco	2014	2014	14.2	Santé en chiffres 2014. Édition 2015. Rabat: Ministère de la Santé - Direction de la Planification et des Ressources Financières/Division de la Planification et des Etudes/Service des Etudes et de l'Information Sanitaire (DPRF/DPE/SEIS); 2015 (in French).
MAR	Morocco	2012	2018	21.2	Enquête Nationale sur la Population et la Santé Familiale (ENPSF) 2018. Rapport final. Rabat: Ministère de la Santé [Morocco]; 2019 (in French).
MOZ	Mozambique	1994	1997	2.7	da Costa Gaspar M, Cossa HA, Ribeiro dos Santos C, Manjate RM, Schoemaker J. Moçambique, Inquérito Demográfico e de Saúde, 1997. Calverton, Maryland, USA: Instituto Nacional de Estatística [Mozambique], Macro International; 1998 (in Portuguese).
MOZ	Mozambique	1998	2003	1.9	Instituto Nacional de Estatística [Moçambique], Ministério da Saúde [Moçambique], MEASURE DHS+/ORC Macro. Moçambique Inquérito Demográfico e de Saúde 2003. Calverton, Maryland, USA: Instituto Nacional de Estatística [Moçambique], Ministério da Saúde [Moçambique], MEASURE DHS+/ORC Macro; 2005 (in Portuguese).
MOZ	Mozambique	2006	2011	3.9	Ministerio da Saude (MISAU) [Mozambique], Instituto Nacional de Estatística (INE) [Mozambique], ICF International. Moçambique Inquérito Demográfico e de Saúde 2011. Calverton, Maryland, USA: MISA [Mozambique], INE [Mozambique], ICF International; 2013 (in Portuguese).
MMR	Myanmar	2010	2016	17.1	Ministry of Health and Sports (MoHS), ICF. Myanmar Demographic and Health Survey 2015-16. Nay Pyi Taw, Myanmar, Rockville, Maryland USA: Ministry of Health and Sports, ICF; 2017.
NAM	Namibia	2001	2007	12.7	Ministry of Health and Social Services (MoHSS) [Namibia], Macro International Inc. Namibia Demographic and Health Survey 2006-07. Windhoek, Namibia, Calverton, Maryland, USA: MoHSS, Macro International Inc.; 2008.
NAM	Namibia	2008	2013	14.4	The Namibia Ministry of Health and Social Services (MoHSS), ICF International. The Namibia Demographic and Health Survey 2013. Windhoek, Namibia, Rockville, Maryland, USA: MoHSS, ICF International; 2014.

ISO Code	Country	Coverage start year	Coverage end year	Caesarean section rate (%)	References
NRU	Nauru	2002	2007	7.5	Nauru Bureau of Statistics, Secretariat of the Pacific Community (SPC), Macro International Inc. Nauru 2007 Demographic and Health Survey. Noumea: SPC; 2009.
NPL	Nepal	1993	1996	1.0	Pradha A, Aryal RH, Regmi G, Ban B, Govindasamy P. Nepal Family Health Survey 1996. Kathmandu, Nepal, Calverton, Maryland: Ministry of Health [Nepal], New ERA, Macro International Inc.; 1997.
NPL	Nepal	1996	2001	0.8	Ministry of Health [Nepal], New ERA, ORC Macro. Nepal Demographic and Health Survey 2001. Calverton, Maryland, USA: Family Health Division, Ministry of Health; New ERA; ORC Macro; 2002.
NPL	Nepal	2001	2006	2.7	Ministry of Health and Population (MOHP) [Nepal], New ERA, Macro International Inc. Nepal Demographic and Health Survey 2006. Kathmandu, Nepal: Ministry of Health and Population, New ERA, Macro International Inc.; 2007.
NPL	Nepal	2006	2011	4.6	Ministry of Health and Population (MOHP) [Nepal], New ERA [Nepal], ICF International. Nepal Demographic and Health Survey 2011. Kathmandu, Nepal: MOHP [Nepal], New ERA [Nepal], ICF International; 2012.
NPL	Nepal	2011	2016	9.0	Ministry of Health, Nepal, New ERA, ICF. Nepal Demographic and Health Survey 2016. Kathmandu, Nepal: Ministry of Health, Nepal; 2017.
NLD	Netherlands	1995	1995	9.6	European Health for All Database (HFA-DB) [online database]. World Health Organization (WHO) Regional Office for Europe; 2012 (http://data.euro.who.int/hfadb, accessed 20 August 2012).
NLD	Netherlands	1996	1996	10.1	European Health for All Database (HFA-DB) [online database]. World Health Organization (WHO) Regional Office for Europe; 2012 (http://data.euro.who.int/hfadb, accessed 20 August 2012).

ISO Code	Country	Coverage start year	Coverage end year	Caesarean section rate (%)	References
NLD	Netherlands	1997	1997	10.4	European Health for All Database (HFA-DB) [online database]. World Health Organization (WHO) Regional Office for Europe; 2012 (http://data.euro.who.int/hfadb, accessed 20 August 2012).
NLD	Netherlands	1998	1998	11.1	European Health for All Database (HFA-DB) [online database]. World Health Organization (WHO) Regional Office for Europe; 2012 (http://data.euro.who.int/hfadb, accessed 20 August 2012).
NLD	Netherlands	1999	1999	10.8	Grote Lijnen. 10 jaar Perinatale Registratie Nederland. Utrecht: Stichting Perinatale Registratie Nederland; 2011 (in Dutch).
NLD	Netherlands	2000	2000	13.0	Grote Lijnen. 10 jaar Perinatale Registratie Nederland. Utrecht: Stichting Perinatale Registratie Nederland; 2011 (in Dutch).
NLD	Netherlands	2001	2001	14.7	Grote Lijnen. 10 jaar Perinatale Registratie Nederland. Utrecht: Stichting Perinatale Registratie Nederland; 2011 (in Dutch).
NLD	Netherlands	2002	2002	14.4	Grote Lijnen. 10 jaar Perinatale Registratie Nederland. Utrecht: Stichting Perinatale Registratie Nederland; 2011 (in Dutch).
NLD	Netherlands	2003	2003	14.4	Grote Lijnen. 10 jaar Perinatale Registratie Nederland. Utrecht: Stichting Perinatale Registratie Nederland; 2011 (in Dutch).
NLD	Netherlands	2004	2004	14.6	Grote Lijnen. 10 jaar Perinatale Registratie Nederland. Utrecht: Stichting Perinatale Registratie Nederland; 2011 (in Dutch).

ISO Code	Country	Coverage start year	Coverage end year	Caesarean section rate (%)	References
NLD	Netherlands	2005	2005	14.6	Grote Lijnen. 10 jaar Perinatale Registratie Nederland. Utrecht: Stichting Perinatale Registratie Nederland; 2011 (in Dutch).
NLD	Netherlands	2006	2006	14.7	Grote Lijnen. 10 jaar Perinatale Registratie Nederland. Utrecht: Stichting Perinatale Registratie Nederland; 2011 (in Dutch).
NLD	Netherlands	2007	2007	14.7	Grote Lijnen. 10 jaar Perinatale Registratie Nederland. Utrecht: Stichting Perinatale Registratie Nederland; 2011 (in Dutch).
NLD	Netherlands	2008	2008	15.0	Grote Lijnen. 10 jaar Perinatale Registratie Nederland. Utrecht: Stichting Perinatale Registratie Nederland; 2011 (in Dutch).
NLD	Netherlands	2010	2010	17.0	EURO-PERISTAT Project with SCPE and EUROCAT. European Perinatal Health Report. Health and Care of Pregnant Women and Babies in Europe in 2010; 2013.
NLD	Netherlands	2012	2012	16.3	Perinatale Registratie Nederland. Grote Lijnen 1999-2012. Utrecht: Stichting Perinatale Registratie Nederland; 2013 (in Dutch).
NLD	Netherlands	2013	2013	16.4	Perinatale Zorg in Nederland 2013. Utrecht: Stichting Perinatale Registratie Nederland; 2014 (in Dutch).
NLD	Netherlands	2014	2014	16.5	Perinatale Zorg in Nederland 2014. Utrecht: Perined; 2015 (in Dutch).

ISO Code	Country	Coverage start year	Coverage end year	Caesarean section rate (%)	References
NLD	Netherlands	2015	2015	16.6	Perinatale Zorg in Nederland 2015. Utrecht: Perined; 2016 (in Dutch).
NLD	Netherlands	2016	2016	16.0	Perinatale Zorg in Nederland 2016. Utrecht: Perined; 2018 (in Dutch).
NLD	Netherlands	2017	2017	15.2	Jaarboeken Zorg in Nederland. Jaarboek Zorg 2017 [website]. Utrecht: Perined; 2018 (https://www.perined.nl/producten/publicaties/jaarboeken, accessed 14 February 2019, in Dutch).
NLD	Netherlands	2018	2018	14.9	Jaarboeken Zorg in Nederland. Jaarboek Zorg 2018 [website]. Utrecht: Perined; 2019 (https://www.perined.nl/producten/publicaties/jaarboeken, accessed 5 March 2020, in Dutch).
NZL	New Zealand	1999	1999	20.4	Report on Maternity 1999. Wellington: Ministry of Health [New Zealand]; 2001.
NZL	New Zealand	2000	2000	20.8	Report on Maternity 2000 & 2001. Wellington: Ministry of Health [New Zealand]; 2003.
NZL	New Zealand	2001	2001	21.3	Maternity Factsheet 2001-2010 (provisional). Wellington: Ministry of Health [New Zealand]; 2011.
NZL	New Zealand	2002	2002	21.8	Maternity Factsheet 2001-2010 (provisional). Wellington: Ministry of Health [New Zealand]; 2011.

ISO Code	Country	Coverage start year	Coverage end year	Caesarean section rate (%)	References
NZL	New Zealand	2003	2003	22.1	Maternity Factsheet 2001-2010 (provisional). Wellington: Ministry of Health [New Zealand]; 2011.
NZL	New Zealand	2004	2004	22.4	Maternity Factsheet 2001-2010 (provisional). Wellington: Ministry of Health [New Zealand]; 2011.
NZL	New Zealand	2005	2005	22.8	Maternity Factsheet 2001-2010 (provisional). Wellington: Ministry of Health [New Zealand]; 2011.
NZL	New Zealand	2006	2006	23.9	Maternity Factsheet 2001-2010 (provisional). Wellington: Ministry of Health [New Zealand]; 2011.
NZL	New Zealand	2007	2007	23.2	Maternity Factsheet 2001-2010 (provisional). Wellington: Ministry of Health [New Zealand]; 2011.
NZL	New Zealand	2008	2008	23.2	Maternity Factsheet 2001-2010 (provisional). Wellington: Ministry of Health [New Zealand]; 2011.
NZL	New Zealand	2009	2009	23.7	Maternity Factsheet 2001-2010 (provisional). Wellington: Ministry of Health [New Zealand]; 2011.
NZL	New Zealand	2010	2010	23.6	Maternity Factsheet 2001-2010 (provisional). Wellington: Ministry of Health [New Zealand]; 2011.

Betran AP, *et al. BMJ Global Health* 2021; 6:e005671. doi: 10.1136/bmjgh-2021-005671

ISO Code	Country	Coverage start year	Coverage end year	Caesarean section rate (%)	References
NZL	New Zealand	2011	2011	24.4	Report on Maternity 2014. Accompanying tables: Labour and Birth: type of birth. Table 30: Number and percentage of women giving birth, by type of birth, 2005-2014. Wellington: Ministry of Health [New Zealand]; 2015.
NZL	New Zealand	2012	2012	25.3	Report on Maternity 2014. Accompanying tables: Labour and Birth: type of birth. Table 30: Number and percentage of women giving birth, by type of birth, 2005-2014. Wellington: Ministry of Health [New Zealand]; 2015.
NZL	New Zealand	2013	2013	26.2	Report on Maternity 2014. Accompanying tables: Labour and Birth: type of birth. Table 30: Number and percentage of women giving birth, by type of birth, 2005-2014. Wellington: Ministry of Health [New Zealand]; 2015.
NZL	New Zealand	2014	2014	25.9	Report on Maternity 2014. Accompanying tables: Labour and Birth: type of birth. Table 30: Number and percentage of women giving birth, by type of birth, 2005-2014. Wellington: Ministry of Health [New Zealand]; 2015.
NZL	New Zealand	2015	2015	25.5	Report on Maternity 2015. Accompanying tables: Labour and Birth: type of birth. Table 33: Number and percentage of women giving birth, by type of birth, 2006-2015. Wellington: Ministry of Health [New Zealand]; 2017.
NZL	New Zealand	2016	2016	26.5	Report on Maternity 2017. Accompanying tables: Labour and Birth: type of birth. Table 33: Number and percentage of women giving birth, by type of birth, 2008-2017. Wellington: Ministry of Health [New Zealand]; 2019.
NZL	New Zealand	2017	2017	27.9	Report on Maternity 2017. Accompanying tables: Labour and Birth: type of birth. Table 33: Number and percentage of women giving birth, by type of birth, 2008-2017. Wellington: Ministry of Health [New Zealand]; 2019.
NIC	Nicaragua	1987	1993	13.5	Encuesta sobre Salud Familiar Nicaragua 92-93. Managua: PROFAMILIA, Centers for Disease Control and Prevention (CDC), USAID; 1993 (in Spanish).

ISO Code	Country	Coverage start year	Coverage end year	Caesarean section rate (%)	References
NIC	Nicaragua	1993	1998	15.4	Instituto Nacional de Estadísticas y Censos - INEC [Nicaragua], Macro International. Nicaragua Encuesta Nicaragüense de Demografía y Salud 1998. Calverton, Maryland, USA: INEC [Nicaragua], Macro International; 1999 (in Spanish).
NIC	Nicaragua	1996	2001	14.7	Instituto Nacional de Estadísticas y Censos - INEC [Nicaragua], ORC Macro. Nicaragua Encuesta Nicaragüense de Demografía y Salud 2001. Calverton, Maryland, USA: INEC [Nicaragua], ORC Macro; 2002 (in Spanish).
NIC	Nicaragua	2001	2007	19.6	Encuesta Nicaragüense de Demografía y Salud (ENDESA) 2006/07. Informe Final. Managua: Instituto Nacional de Información de Desarrollo (INIDE), Ministerio de Salud [Nicaragua]; 2008 (in Spanish).
NIC	Nicaragua	2006	2012	29.7	Encuesta Nicaragüense de Demografía y Salud (ENDESA) 2011/12. Informe Preliminar. Managua: Instituto Nacional de Información de Desarrollo (INIDE), Ministerio de Salud [Nicaragua]; 2013 (in Spanish).
NER	Niger	1987	1992	0.9	Kourguéni IA, Garba B, Barrère B (Direction de la Statistique et des Comptes Nationaux [Niger], Macro International). Niger Enquête Démographique et de Santé 1992. Columbia, Maryland, USA: Direction de la Statistique et des Comptes Nationaux [Niger], Macro International; 1993 (in French).
NER	Niger	1995	1998	0.6	Attama S, Seroussi M, Kourguéni IA, Koché H, Barrère B (Care International Niger, Macro International Inc.). Enquête Démographique et de Santé, Niger 1998. Calverton, Maryland, USA: Care International Niger, Macro International Inc.; 1999 (in French).
NER	Niger	2001	2006	1.0	Institut National de la Statistique (INS) [Niger], Macro International Inc. Enquête Démographique et de Santé et à Indicateurs Multiples du Niger 2006. Calverton, Maryland, USA: INS [Niger], Macro International Inc.; 2007 (in French).
NER	Niger	2007	2012	1.4	Institut National de la Statistique (INS) [Niger], ICF International. Enquête Démographique et de Santé et à Indicateurs Multiples du Niger 2012. Calverton, Maryland, USA: INS [Niger], ICF International; 2013 (in French).

ISO Code	Country	Coverage start year	Coverage end year	Caesarean section rate (%)	References
NGA	Nigeria	1985	1990	2.5	Federal Office of Statistics [Nigeria], Institute for Resource Development (IRD)/Macro International. Nigeria Demographic and Health Survey 1990. Columbia, Maryland, USA: Federal Office of Statistics [Nigeria], IRD/Macro International; 1992.
NGA	Nigeria	1996	1999	3.7	National Population Commission [Nigeria]. Nigeria Demographic and Health Survey 1999. Calverton, Maryland: National Population Commission [Nigeria], ORC/Macro; 2000.
NGA	Nigeria	1998	2003	1.7	National Population Commission (NPC) [Nigeria], ORC Macro. Nigeria Demographic and Health Survey 2003. Calverton, Maryland: National Population Commission, ORC Macro; 2004.
NGA	Nigeria	2003	2008	1.8	National Population Commission (NPC) [Nigeria], ICF Macro. Nigeria Demographic and Health Survey 2008. Abuja, Nigeria: NPC [Nigeria], ICF Macro; 2009.
NGA	Nigeria	2008	2013	2.0	National Population Commission (NPC) [Nigeria], ICF International. Nigeria Demographic and Health Survey 2013. Abuja, Nigeria, Rockville, Maryland, USA: NPC, ICF International; 2014.
NGA	Nigeria	2013	2018	2.7	Nigeria Demographic and Health Survey 2018. Abuja, Rockville, Maryland, USA: National Population Commission (NPC) [Nigeria], ICF International; 2019.
NOR	Norway	1990	1990	12.7	Statistikk fra Medisinsk fødselsregister [online database]. Oslo: Folkehelseinstituttet, Medisinsk fødselsregister [Norwegian Institute of Public Health (NIPH), Medical Birth Registry]; 2012 (http://www.fhi.no/eway/default.aspx?pid=233&trg=MainArea_5661&MainArea_5661=56, accessed 24 September 2012, in Norwegian).
NOR	Norway	1991	1991	12.4	Statistikk fra Medisinsk fødselsregister [online database]. Oslo: Folkehelseinstituttet, Medisinsk fødselsregister [Norwegian Institute of Public Health (NIPH), Medical Birth Registry]; 2012 (http://www.fhi.no/eway/default.aspx?pid=233&trg=MainArea_5661&MainArea_5661=56, accessed 24 September 2012, in Norwegian).

ISO Code	Country	Coverage start year	Coverage end year	Caesarean section rate (%)	References
NOR	Norway	1992	1992	12.5	Statistikk fra Medisinsk fødselsregister [online database]. Oslo: Folkehelseinstituttet, Medisinsk fødselsregister [Norwegian Institute of Public Health (NIPH), Medical Birth Registry]; 2012 (http://www.fhi.no/eway/default.aspx?pid=233&trg=MainArea_5661&MainArea_5661=56, accessed 24 September 2012, in Norwegian).
NOR	Norway	1993	1993	12.4	Statistikk fra Medisinsk fødselsregister [online database]. Oslo: Folkehelseinstituttet, Medisinsk fødselsregister [Norwegian Institute of Public Health (NIPH), Medical Birth Registry]; 2012 (http://www.fhi.no/eway/default.aspx?pid=233&trg=MainArea_5661&MainArea_5661=56, accessed 24 September 2012, in Norwegian).
NOR	Norway	1994	1994	12.5	Statistikk fra Medisinsk fødselsregister [online database]. Oslo: Folkehelseinstituttet, Medisinsk fødselsregister [Norwegian Institute of Public Health (NIPH), Medical Birth Registry]; 2012 (http://www.fhi.no/eway/default.aspx?pid=233&trg=MainArea_5661&MainArea_5661=56, accessed 24 September 2012, in Norwegian).
NOR	Norway	1995	1995	12.6	Statistikk fra Medisinsk fødselsregister [online database]. Oslo: Folkehelseinstituttet, Medisinsk fødselsregister [Norwegian Institute of Public Health (NIPH), Medical Birth Registry]; 2012 (http://www.fhi.no/eway/default.aspx?pid=233&trg=MainArea_5661&MainArea_5661=56, accessed 24 September 2012, in Norwegian).
NOR	Norway	1996	1996	12.6	Statistikk fra Medisinsk fødselsregister [online database]. Oslo: Folkehelseinstituttet, Medisinsk fødselsregister [Norwegian Institute of Public Health (NIPH), Medical Birth Registry]; 2012 (http://www.fhi.no/eway/default.aspx?pid=233&trg=MainArea_5661&MainArea_5661=56, accessed 24 September 2012, in Norwegian).
NOR	Norway	1997	1997	12.8	Statistikk fra Medisinsk fødselsregister [online database]. Oslo: Folkehelseinstituttet, Medisinsk fødselsregister [Norwegian Institute of Public Health (NIPH), Medical Birth Registry]; 2012 (http://www.fhi.no/eway/default.aspx?pid=233&trg=MainArea_5661&MainArea_5661=56, accessed 24 September 2012, in Norwegian).
NOR	Norway	1998	1998	13.5	Statistikk fra Medisinsk fødselsregister [online database]. Oslo: Folkehelseinstituttet, Medisinsk fødselsregister [Norwegian Institute of Public Health (NIPH), Medical Birth Registry]; 2012 (http://www.fhi.no/eway/default.aspx?pid=233&trg=MainArea_5661&MainArea_5661=56, accessed 24 September 2012, in Norwegian).
NOR	Norway	1999	1999	13.4	Statistikk fra Medisinsk fødselsregister [online database]. Oslo: Folkehelseinstituttet, Medisinsk fødselsregister [Norwegian Institute of Public Health (NIPH), Medical Birth Registry]; 2012 (http://www.fhi.no/eway/default.aspx?pid=233&trg=MainArea_5661&MainArea_5661=56, accessed 24 September 2012, in Norwegian).

ISO Code	Country	Coverage start year	Coverage end year	Caesarean section rate (%)	References
NOR	Norway	2000	2000	13.5	Statistikk fra Medisinsk fødselsregister [online database]. Oslo: Folkehelseinstituttet, Medisinsk fødselsregister [Norwegian Institute of Public Health (NIPH), Medical Birth Registry]; 2012 (http://www.fhi.no/eway/default.aspx?pid=233&trg=MainArea_5661&MainArea_5661=56, accessed 24 September 2012, in Norwegian).
NOR	Norway	2001	2001	15.5	Statistikk fra Medisinsk fødselsregister [online database]. Oslo: Folkehelseinstituttet, Medisinsk fødselsregister [Norwegian Institute of Public Health (NIPH), Medical Birth Registry]; 2012 (http://www.fhi.no/eway/default.aspx?pid=233&trg=MainArea_5661&MainArea_5661=56, accessed 24 September 2012, in Norwegian).
NOR	Norway	2002	2002	15.8	Statistikk fra Medisinsk fødselsregister [online database]. Oslo: Folkehelseinstituttet, Medisinsk fødselsregister [Norwegian Institute of Public Health (NIPH), Medical Birth Registry]; 2012 (http://www.fhi.no/eway/default.aspx?pid=233&trg=MainArea_5661&MainArea_5661=56, accessed 24 September 2012, in Norwegian).
NOR	Norway	2003	2003	15.9	Statistikk fra Medisinsk fødselsregister [online database]. Oslo: Folkehelseinstituttet, Medisinsk fødselsregister [Norwegian Institute of Public Health (NIPH), Medical Birth Registry]; 2012 (http://www.fhi.no/eway/default.aspx?pid=233&trg=MainArea_5661&MainArea_5661=56, accessed 24 September 2012, in Norwegian).
NOR	Norway	2004	2004	15.6	Statistikk fra Medisinsk fødselsregister [online database]. Oslo: Folkehelseinstituttet, Medisinsk fødselsregister [Norwegian Institute of Public Health (NIPH), Medical Birth Registry]; 2012 (http://www.fhi.no/eway/default.aspx?pid=233&trg=MainArea_5661&MainArea_5661=56, accessed 24 September 2012, in Norwegian).
NOR	Norway	2005	2005	16.6	Statistikk fra Medisinsk fødselsregister [online database]. Oslo: Folkehelseinstituttet, Medisinsk fødselsregister [Norwegian Institute of Public Health (NIPH), Medical Birth Registry]; 2012 (http://www.fhi.no/eway/default.aspx?pid=233&trg=MainArea_5661&MainArea_5661=56, accessed 24 September 2012, in Norwegian).
NOR	Norway	2006	2006	16.4	Statistikk fra Medisinsk fødselsregister [online database]. Oslo: Folkehelseinstituttet, Medisinsk fødselsregister [Norwegian Institute of Public Health (NIPH), Medical Birth Registry]; 2012 (http://www.fhi.no/eway/default.aspx?pid=233&trg=MainArea_5661&MainArea_5661=56, accessed 24 September 2012, in Norwegian).
NOR	Norway	2007	2007	16.7	Statistikk fra Medisinsk fødselsregister [online database]. Oslo: Folkehelseinstituttet, Medisinsk fødselsregister [Norwegian Institute of Public Health (NIPH), Medical Birth Registry]; 2012 (http://www.fhi.no/eway/default.aspx?pid=233&trg=MainArea_5661&MainArea_5661=56, accessed 24 September 2012, in Norwegian).

ISO Code	Country	Coverage start year	Coverage end year	Caesarean section rate (%)	References
NOR	Norway	2008	2008	17.1	Statistikk fra Medisinsk fødselsregister [online database]. Oslo: Folkehelseinstituttet, Medisinsk fødselsregister [Norwegian Institute of Public Health (NIPH), Medical Birth Registry]; 2012 (http://www.fhi.no/eway/default.aspx?pid=233&trg=MainArea_5661&MainArea_5661=56, accessed 24 September 2012, in Norwegian).
NOR	Norway	2009	2009	16.9	Statistikk fra Medisinsk fødselsregister [online database]. Oslo: Folkehelseinstituttet, Medisinsk fødselsregister [Norwegian Institute of Public Health (NIPH), Medical Birth Registry]; 2012 (http://www.fhi.no/eway/default.aspx?pid=233&trg=MainArea_5661&MainArea_5661=56, accessed 24 September 2012, in Norwegian).
NOR	Norway	2010	2010	17.1	Statistikk fra Medisinsk fødselsregister [online database]. Oslo: Folkehelseinstituttet, Medisinsk fødselsregister [Norwegian Institute of Public Health (NIPH), Medical Birth Registry]; 2012 (http://www.fhi.no/eway/default.aspx?pid=233&trg=MainArea_5661&MainArea_5661=56, accessed 24 September 2012, in Norwegian).
NOR	Norway	2011	2011	17.0	Dataset: F10/I2b: Keisersnitt. 2011. [online database]. Oslo: Folkehelseinstituttet, Medisinsk fødselsregister [Norwegian Institute of Public Health (NIPH), Medical Birth Registry]; 2013 (http://www.fhi.no/helseregistre/medisinsk-fodselsregister/statistikk, accessed 28 October 2013, in Norwegian).
NOR	Norway	2013	2013	16.8	Dataset: F10/I2b: Keisersnitt. 2013. [online database]. Oslo: Folkehelseinstituttet, Medisinsk fødselsregister [Norwegian Institute of Public Health (NIPH), Medical Birth Registry]; 2015 (http://www.fhi.no/helseregistre/medisinsk-fodselsregister/statistikk, accessed 13 January 2015, in Norwegian).
NOR	Norway	2014	2014	16.6	Dataset: F10c-2/I2b-2: Keisersnitt utført. 2014. [online database]. Oslo: Folkehelseinstituttet, Medisinsk fødselsregister [Norwegian Institute of Public Health (NIPH), Medical Birth Registry]; 2015 (http://statistikkbank.fhi.no/mfr, in Norwegian).
NOR	Norway	2015	2015	15.9	Dataset: Is4: Inngrep under fødselen. 2015. [online database]. Oslo: Folkehelseinstituttet, Medisinsk fødselsregister [Norwegian Institute of Public Health (NIPH), Medical Birth Registry]; 2016 (http://statistikkbank.fhi.no/mfr, in Norwegian).
NOR	Norway	2016	2016	16.1	Dataset: F10c-2: Keisersnitt utført. 2016. [online database]. Oslo: Folkehelseinstituttet, Medisinsk fødselsregister [Norwegian Institute of Public Health (NIPH), Medical Birth Registry]; 2017 (http://statistikkbank.fhi.no/mfr, in Norwegian).

ISO Code	Country	Coverage start year	Coverage end year	Caesarean section rate (%)	References
NOR	Norway	2017	2017	16.0	Dataset: F10c-2: Keisersnitt utført. 2017. [online database]. Oslo: Folkehelseinstituttet, Medisinsk fødselsregister [Norwegian Institute of Public Health (NIPH), Medical Birth Registry]; 2019 (http://statistikkbank.fhi.no/mfr, accessed 14 February 2019, in Norwegian).
NOR	Norway	2018	2018	15.9	Dataset: F10c-2: Keisersnitt utført. 2018. [online database]. Oslo: Folkehelseinstituttet, Medisinsk fødselsregister [Norwegian Institute of Public Health (NIPH), Medical Birth Registry]; 2020 (http://statistikkbank.fhi.no/mfr, accessed 5 March 2020, in Norwegian).
OMN	Oman	1990	1995	6.6	Jurdi R, Khawaja M. Caesarean section rates in the Arab region: a cross-national study. Health Policy Plan. 2004;19(2):101-10.
OMN	Oman	1995	2000	11.5	National Health Survey, 2000. Volume II - Reproductive Health Study. Muscat: Ministry of Health [Oman], United Nations Children's Fund (UNICEF), United Nations Population Fund (UNFPA); 2001.
OMN	Oman	2003	2003	11.0	Annual Health Report 2003. Muscat: Ministry of Health, Department of Health Information and Statistics, Directorate General of Planning [Oman]; 2004.
OMN	Oman	2004	2004	11.5	Annual Health Report 2004. Muscat: Ministry of Health, Department of Health Information and Statistics, Directorate General of Planning [Oman]; 2005.
OMN	Oman	2005	2005	12.6	Annual Health Report 2005. Muscat: Ministry of Health, Department of Health Information and Statistics, Directorate General of Planning [Oman]; 2006.
OMN	Oman	2006	2006	13.5	Annual Health Report 2006. Muscat: Ministry of Health, Department of Health Information and Statistics, Directorate General of Planning [Oman]; 2007.

ISO Code	Country	Coverage start year	Coverage end year	Caesarean section rate (%)	References
OMN	Oman	2007	2007	14.1	Annual Health Report 2007. Muscat: Ministry of Health, Department of Health Information and Statistics, Directorate General of Planning [Oman]; 2008.
OMN	Oman	2008	2008	14.9	Annual Health Report 2008. Muscat: Ministry of Health, Department of Health Information and Statistics, Directorate General of Planning [Oman]; 2009.
OMN	Oman	2009	2009	15.7	Annual Health Report 2009. Muscat: Ministry of Health, Department of Health Information and Statistics, Directorate General of Planning [Oman]; 2010.
OMN	Oman	2010	2010	16.4	Annual Health Report 2010. Muscat: Ministry of Health, Department of Health Information and Statistics, Directorate General of Planning [Oman]; 2011.
OMN	Oman	2011	2011	17.0	Annual Health Report 2011. Muscat: Ministry of Health, Department of Health Information and Statistics, Directorate General of Planning [Oman]; 2012.
OMN	Oman	2012	2014	19.4	National Centre for Statistics and Information, United Nations Children's Fund (UNICEF). Multiple Indicator Cluster Survey 2014, Final Report. Muscat, Oman: National Centre for Statistics and Information; 2017 (in Arabic).
OMN	Oman	2012	2012	17.5	Annual Health Report 2016. Muscat: Ministry of Health, Department of Health Information and Statistics, Directorate General of Planning [Oman]; 2017.
OMN	Oman	2014	2014	19.3	Annual Health Report 2016. Muscat: Ministry of Health, Department of Health Information and Statistics, Directorate General of Planning [Oman]; 2017.

ISO Code	Country	Coverage start year	Coverage end year	Caesarean section rate (%)	References
OMN	Oman	2015	2015	19.8	Annual Health Report 2016. Muscat: Ministry of Health, Department of Health Information and Statistics, Directorate General of Planning [Oman]; 2017.
OMN	Oman	2016	2016	19.5	Annual Health Report 2016. Muscat: Ministry of Health, Department of Health Information and Statistics, Directorate General of Planning [Oman]; 2017.
OMN	Oman	2017	2017	19.0	Annual Health Report 2017. Muscat: Ministry of Health, Department of Health Information and Statistics, Directorate General of Planning [Oman]; 2018.
OMN	Oman	2018	2018	19.4	Annual Health Report 2018. Muscat: Ministry of Health, Department of Health Information and Statistics, Directorate General of Planning [Oman]; 2019.
PAK	Pakistan	1985	1991	2.7	National Institute of Population Studies (NIPS) [Pakistan], Institute for Resource Development (IRD)/Macro International. Pakistan Demographic and Health Survey 1990/1991. Islamabad, Pakistan: NIPS [Pakistan], IRD/Macro International; 1992.
PAK	Pakistan	2001	2007	7.3	National Institute of Population Studies (NIPS) [Pakistan], Macro International Inc. Pakistan Demographic and Health Survey 2006-07. Islamabad, Pakistan: NIPS, Macro International Inc.; 2008.
PAK	Pakistan	2007	2013	14.1	National Institute of Population Studies (NIPS) [Pakistan], ICF International. Pakistan Demographic and Health Survey 2012-13. Islamabad, Pakistan, Calverton, Maryland, USA: NIPS [Pakistan], ICF International; 2013.
PAK	Pakistan	2013	2018	22.3	National Institute of Population Studies (NIPS) [Pakistan], ICF. Pakistan Demographic and Health Survey 2017-18. Islamabad, Pakistan, and Rockville, Maryland, USA: NIPS, ICF; 2019.

ISO Code	Country	Coverage start year	Coverage end year	Caesarean section rate (%)	References
PAN	Panama	2004	2009	20.2	De León Richardson RG, Martínez García L, Chu V EE, Mendoza Q AI, Chamorro Mojica F, Poveda C et al. Panamá Encuesta Nacional de Salud Sexual y Reproductiva 2009 (ENASSER 2009) - Informe Final. Panamá: Instituto Conmemorativo Gorgas de Estudios de la Salud; 2011 (in Spanish).
PAN	Panama	2011	2013	27.7	Contraloría General de la República, Fondo de las Naciones Unidas para la Infancia (UNICEF). Encuesta de Indicadores Múltiples por Conglomerados de Panamá 2013, Resultados Principales Panamá. Panamá: Contraloría General; 2014 (in Spanish).
PNG	Papua New Guinea	2011	2018	3.0	Papua New Guinea Demographic and Health Survey 2016-18. Oirt Moresby, Rockeville, Maryland: National Statistical Office (NSO) [Papua New Guinea], ICF; 2019.
PRY	Paraguay	1985	1990	13.0	Centro Paraguayo de Estudios de Población, Macro Systems Inc. Encuesta Nacional de Demografía y Salud 1990. Columbia, Maryland, USA: Macro Systems Inc.; 1991 (in Spanish).
PRY	Paraguay	1990	1996	15.3	Encuesta Nacional de Demografía y Salud Reproductiva 1995-1996 (ENDSR-95/96). Asunción: Centro Paraguay de Estudios de Población (CEPEP), Centros para el Control y Prevención de Enfermedades (CDC), Agencia del Gobierno de los Estados Unidos para el Desarrollo Internacional (USAID); 1997 (in Spanish).
PRY	Paraguay	1995	1998	16.5	Encuesta Nacional de Salud Materno Infantil: 1998 (ENSMI-98). Informe Final. Asunción: Centro Paraguay de Estudios de Población (CEPEP), Centros para el Control y Prevención de Enfermedades (CDC), Agencia del Gobierno de los Estados Unidos para el Desarrollo Internacional (USAID); 1999 (in Spanish).
PRY	Paraguay	1999	2004	26.9	Encuesta Nacional de Demografía y Salud Reproductiva (ENDSSR) 2004. Informe Final. Asunción: Centro Paraguayo de Estudios de Población (CEPEP), Agencia del Gobierno de los Estados Unidos para el Desarrollo Internacional (USAID), Centros para el Control y Prevención de Enfermedades (CDC), Fondo de Población de las Naciones Unidas (UNFPA), Federación Internacional de Planificación Familiar (IPPF); 2005 (in Spanish).
PRY	Paraguay	2003	2008	33.1	Encuesta Nacional de Demografía y Salud Reproductiva (ENDSSR) 2008. Informe Final. Asunción: Centro Paraguayo de Estudios de Población (CEPEP), Agencia del Gobierno de los Estados Unidos para el Desarrollo Internacional (USAID), Centros para el Control y Prevención de Enfermedades (CDC), Fondo de las Naciones Unidas para la Infancia (UNICEF), Federación Internacional de Planificación Familiar (IPPF); 2009 (in Spanish).

ISO Code	Country	Coverage start year	Coverage end year	Caesarean section rate (%)	References
PRY	Paraguay	2011	2011	43.6	Indicadores Básicos de Salud 2016. Asunción: Organización Panamericana de la Salud (OPS/PAHO), Organización Mundial de la Salud (OMS/WHO), Ministerio de Salud Pública y Bienestar Social [Paraguay]; 2017 (in Spanish).
PRY	Paraguay	2012	2012	43.8	Indicadores Básicos de Salud 2016. Asunción: Organización Panamericana de la Salud (OPS/PAHO), Organización Mundial de la Salud (OMS/WHO), Ministerio de Salud Pública y Bienestar Social [Paraguay]; 2017 (in Spanish).
PRY	Paraguay	2013	2013	47.8	Indicadores Básicos de Salud 2016. Asunción: Organización Panamericana de la Salud (OPS/PAHO), Organización Mundial de la Salud (OMS/WHO), Ministerio de Salud Pública y Bienestar Social [Paraguay]; 2017 (in Spanish).
PRY	Paraguay	2014	2014	48.3	Indicadores Básicos de Salud 2016. Asunción: Organización Panamericana de la Salud (OPS/PAHO), Organización Mundial de la Salud (OMS/WHO), Ministerio de Salud Pública y Bienestar Social [Paraguay]; 2017 (in Spanish).
PRY	Paraguay	2014	2016	45.9	Encuesta de Indicadores Múltiples por Conglomerados - MICS Paraguay 2016. Fernando de la Mora: Dirección General de Estadística, Encuestas y Censos, Ministerio de Salud Pública Y Bienestar Social, Ministerio de la Secretaria Técnica de Planificación del Desarrollo Económico y Social, Fondo de las Naciones Unidas para la Infancia (UNICEF), Banco Interamericano de Desarrollo (BID), ERSSAN; 2017 (in Spanish).
PRY	Paraguay	2016	2016	48.5	Indicadores Básicos de Salud 2017. Asunción: Organización Panamericana de la Salud (OPS/PAHO), Organización Mundial de la Salud (OMS/WHO), Ministerio de Salud Pública y Bienestar Social [Paraguay]; 2017 (in Spanish).
PER	Peru	1986	1992	10.0	Instituto Nacional de Estadística e Informática (INEI) [Perú], Asociación Benéfica PRISMA, Macro International Inc. Perú Encuesta Demográfica y de Salud Familiar - ENDES 1991-1992. Lima, Perú: INEI [Perú], Asociación Benéfica PRISMA, Macro International Inc.; 1992 (in Spanish).
PER	Peru	1991	1996	8.7	Instituto Nacional de Estadística e Informática (INEI) [Perú], ORC Macro. Perú Encuesta Demográfica y de Salud Familiar - ENDES 1996. Lima, Perú: INEI [Perú], ORC Macro; 1997 (in Spanish).

ISO Code	Country	Coverage start year	Coverage end year	Caesarean section rate (%)	References
PER	Peru	1995	2000	12.7	Instituto Nacional de Estadística e Informática (INEI) [Perú], Macro International. Perú Encuesta Demográfica y de Salud Familiar - ENDES 2000. Lima, Perú: INEI [Perú], Macro International; 2001 (in Spanish).
PER	Peru	1999	2006	15.9	Instituto Nacional de Estadística e Informática (INEI) [Perú], ORC Macro. Perú Encuesta Demográfica y de Salud Familiar - ENDES 2004-2006. Lima, Perú: Instituto Nacional de Estadística e Informática (INEI) [Perú], ORC Macro; 2007 (in Spanish).
PER	Peru	2002	2008	18.4	Instituto Nacional de Estadística e Informática (INEI) [Perú], ORC Macro. Perú Encuesta Demográfica y de Salud Familiar - ENDES 2007-2008. Lima, Perú: Instituto Nacional de Estadística e Informática (INEI) [Perú], ORC Macro; 2009 (in Spanish).
PER	Peru	2005	2009	21.4	Instituto Nacional de Estadística e Informática (INEI) [Perú], ORC Macro. Perú Encuesta Demográfica y de Salud Familiar - ENDES Continua 2009. Lima, Perú: INEI [Perú], ORC Macro; 2010 (in Spanish).
PER	Peru	2005	2010	20.4	Instituto Nacional de Estadística e Informática (INEI) [Perú], ORC Macro. Perú Encuesta Demográfica y de Salud Familiar Continua - ENDES 2010. Lima, Perú: INEI [Perú], ORC Macro; 2011 (in Spanish).
PER	Peru	2006	2011	22.9	Instituto Nacional de Estadística e Informática (INEI) [Perú]. Perú Encuesta Demográfica y de Salud Familiar - ENDES 2011. Lima, Perú: INEI [Perú]; 2012 (in Spanish).
PER	Peru	2007	2012	25.3	Instituto Nacional de Estadística e Informática (INEI) [Perú]. Perú Encuesta Demográfica y de Salud Familiar - ENDES 2012. Lima, Perú: INEI [Perú]; 2013 (in Spanish).
PER	Peru	2009	2014	28.6	Instituto Nacional de Estadística e Informática (INEI) [Perú]. Perú Encuesta Demográfica y de Salud Familiar - ENDES 2014. Lima, Perú: INEI [Perú]; 2015 (in Spanish).

ISO Code	Country	Coverage start year	Coverage end year	Caesarean section rate (%)	References
PER	Peru	2011	2016	31.6	Instituto Nacional de Estadística e Informática (INEI) [Perú]. Perú Encuesta Demográfica y de Salud Familiar - ENDES 2016. Nacional y Regional. Lima, Perú: INEI [Perú]; 2017 (in Spanish).
PER	Peru	2012	2017	34.2	Perú Encuesta Demográfica y de Salud Familiar - ENDES 2017. Lima: Instituto Nacional de Estadística e Informática (INEI) [Perú]; 2018 (in Spanish).
PER	Peru	2013	2018	34.5	Perú: Encuesta Demográfica y de Salud Familiar - ENDES 2018. Lima: Instituto Nacional de Estadística e Informática (INEI) [Perú]; 2019 (in Spanish).
PER	Peru	2014	2019	33.7	Perú: Encuesta Demográfica y de Salud Familiar - ENDES 2019. Resultados Preliminares al 50% de la muestra. Lima: Instituto Nacional de Estadística e Informática (INEI) [Perú]; 2019 (in Spanish).
PHL	Philippines	1989	1993	5.9	National Statistics Office (NSO) [Philippines], Macro International Inc. National Demographic Survey 1993. Calverton, Maryland: (NSO) [Philippines], Macro International Inc.; 1994.
PHL	Philippines	1993	1998	5.7	National Statistics Office (NSO) [Philippines], Department of Health (DOH) [Philippines], Macro International Inc. (MI). National Demographic and Health Survey 1998. Manila: National Statistics Office (NSO) [Philippines], Department of Health (DOH) [Philippines], Macro International Inc. (MI); 1999.
PHL	Philippines	1998	2003	7.3	National Statistics Office (NSO) [Philippines], ORC Macro. National Demographic and Health Survey 2003. Calverton, Maryland: NSO [Philippines], ORC Macro; 2004.
PHL	Philippines	2003	2008	9.5	National Statistics Office (NSO) [Philippines], ICF Macro. National Demographic and Health Survey 2008. Calverton, Maryland: National Statistics Office, ICF Macro; 2009.

ISO Code	Country	Coverage start year	Coverage end year	Caesarean section rate (%)	References
PHL	Philippines	2006	2011	11.1	2011 Family Health Survey (FHS). Final Report. Quezon City: National Statistics Office [Philippines], United States Agency for International Development (USAID); 2012.
PHL	Philippines	2008	2013	9.3	Philippine Statistics Authority (PSA) [Philippines], ICF International. Philippines National Demographic and Health Survey 2013. Manila, Philippines, and Rockville, Maryland, USA: PSA, ICF International; 2014.
PHL	Philippines	2012	2017	12.7	Philippines National Demographic and Health Survey 2017: Final Report. Quezon City, Philippines, and Rockville, Maryland, USA: Philippine Statistics Authority (PSA), ICF; 2018. .
POL	Poland	1994	1994	13.8	European Health for All Database (HFA-DB) [online database]. World Health Organization (WHO) Regional Office for Europe; 2012 (http://data.euro.who.int/hfadb, accessed 20 August 2012).
POL	Poland	1995	1995	15.2	European Health for All Database (HFA-DB) [online database]. World Health Organization (WHO) Regional Office for Europe; 2012 (http://data.euro.who.int/hfadb, accessed 20 August 2012).
POL	Poland	1996	1996	15.6	European Health for All Database (HFA-DB) [online database]. World Health Organization (WHO) Regional Office for Europe; 2012 (http://data.euro.who.int/hfadb, accessed 20 August 2012).
POL	Poland	1997	1997	16.1	European Health for All Database (HFA-DB) [online database]. World Health Organization (WHO) Regional Office for Europe; 2012 (http://data.euro.who.int/hfadb, accessed 20 August 2012).
POL	Poland	2005	2005	27.2	Podstawowe dane z zakresu ochrony zdrowia w 2006 r. [Basic data on health care in 2006]. Warsaw: Główny Urząd Statystyczny [Central Statistical Office, Poland]; 2007 (in Polish).

ISO Code	Country	Coverage start year	Coverage end year	Caesarean section rate (%)	References
POL	Poland	2006	2006	28.6	Podstawowe dane z zakresu ochrony zdrowia w 2006 r. [Basic data on health care in 2006]. Warsaw: Główny Urząd Statystyczny [Central Statistical Office, Poland]; 2007 (in Polish).
POL	Poland	2007	2007	29.5	Podstawowe dane z zakresu ochrony zdrowia w 2007 r. [Basic data on health care in 2007]. Warsaw: Główny Urząd Statystyczny [Central Statistical Office, Poland]; 2008 (in Polish).
POL	Poland	2008	2008	30.3	Basic data on health care in 2008. Warsaw: Central Statistical Office [Poland]; 2009.
POL	Poland	2009	2009	31.9	Basic data on health care in 2009. Warsaw: Central Statistical Office [Poland]; 2010.
POL	Poland	2010	2010	33.7	Health and health care in 2010. Warsaw: Central Statistical Office [Poland]; 2012.
POL	Poland	2011	2011	35.2	Health and health care in 2011. Warsaw: Central Statistical Office [Poland]; 2012.
POL	Poland	2012	2012	31.6	European Health for All Database (HFA-DB) [online database]. World Health Organization (WHO) Regional Office for Europe; 2016 (http://data.euro.who.int/hfadb, accessed 8 August 2016).
POL	Poland	2013	2013	34.6	European Health for All Database (HFA-DB) [online database]. World Health Organization (WHO) Regional Office for Europe; 2016 (http://data.euro.who.int/hfadb, accessed 8 August 2016).

ISO Code	Country	Coverage start year	Coverage end year	Caesarean section rate (%)	References
POL	Poland	2014	2014	35.6	European Health for All Database (HFA-DB) [online database]. World Health Organization (WHO) Regional Office for Europe; 2016 (http://data.euro.who.int/hfadb, accessed 8 August 2016).
PRT	Portugal	1990	1990	18.6	European Health for All Database (HFA-DB) [online database]. World Health Organization (WHO) Regional Office for Europe; 2012 (http://data.euro.who.int/hfadb, accessed 20 August 2012).
PRT	Portugal	1991	1991	20.0	European Health for All Database (HFA-DB) [online database]. World Health Organization (WHO) Regional Office for Europe; 2012 (http://data.euro.who.int/hfadb, accessed 20 August 2012).
PRT	Portugal	1992	1992	21.8	European Health for All Database (HFA-DB) [online database]. World Health Organization (WHO) Regional Office for Europe; 2012 (http://data.euro.who.int/hfadb, accessed 20 August 2012).
PRT	Portugal	1993	1993	22.5	European Health for All Database (HFA-DB) [online database]. World Health Organization (WHO) Regional Office for Europe; 2012 (http://data.euro.who.int/hfadb, accessed 20 August 2012).
PRT	Portugal	1994	1994	23.9	European Health for All Database (HFA-DB) [online database]. World Health Organization (WHO) Regional Office for Europe; 2012 (http://data.euro.who.int/hfadb, accessed 20 August 2012).
PRT	Portugal	1995	1995	24.2	European Health for All Database (HFA-DB) [online database]. World Health Organization (WHO) Regional Office for Europe; 2012 (http://data.euro.who.int/hfadb, accessed 20 August 2012).
PRT	Portugal	1996	1996	24.6	European Health for All Database (HFA-DB) [online database]. World Health Organization (WHO) Regional Office for Europe; 2012 (http://data.euro.who.int/hfadb, accessed 20 August 2012).

ISO Code	Country	Coverage start year	Coverage end year	Caesarean section rate (%)	References
PRT	Portugal	1997	1997	27.4	European Health for All Database (HFA-DB) [online database]. World Health Organization (WHO) Regional Office for Europe; 2012 (http://data.euro.who.int/hfadb, accessed 20 August 2012).
PRT	Portugal	1998	1998	27.5	European Health for All Database (HFA-DB) [online database]. World Health Organization (WHO) Regional Office for Europe; 2012 (http://data.euro.who.int/hfadb, accessed 20 August 2012).
PRT	Portugal	2000	2000	27.7	European Health for All Database (HFA-DB) [online database]. World Health Organization (WHO) Regional Office for Europe; 2012 (http://data.euro.who.int/hfadb, accessed 20 August 2012).
PRT	Portugal	2001	2001	29.5	European Health for All Database (HFA-DB) [online database]. World Health Organization (WHO) Regional Office for Europe; 2012 (http://data.euro.who.int/hfadb, accessed 20 August 2012).
PRT	Portugal	2002	2002	30.2	Caesarean deliveries performed in hospitals (No.) by Geographic localization, Annual Live births (No.) by Sex; annual [online database]. Lisbon: Instituto Nacional de Estadística (INE) [Portugal]; 2012 (http://www.ine.pt, accessed 25 September 2012).
PRT	Portugal	2003	2003	31.9	Caesarean deliveries performed in hospitals (No.) by Geographic localization, Annual Live births (No.) by Sex; annual [online database]. Lisbon: Instituto Nacional de Estadística (INE) [Portugal]; 2012 (http://www.ine.pt, accessed 25 September 2012).
PRT	Portugal	2004	2004	32.4	Caesarean deliveries performed in hospitals (No.) by Geographic localization, Annual Live births (No.) by Sex; annual [online database]. Lisbon: Instituto Nacional de Estadística (INE) [Portugal]; 2012 (http://www.ine.pt, accessed 25 September 2012).
PRT	Portugal	2005	2005	34.0	Caesarean deliveries performed in hospitals (No.) by Geographic localization, Annual Live births (No.) by Sex; annual [online database]. Lisbon: Instituto Nacional de Estadística (INE) [Portugal]; 2012 (http://www.ine.pt, accessed 25 September 2012).

ISO Code	Country	Coverage start year	Coverage end year	Caesarean section rate (%)	References
PRT	Portugal	2006	2006	34.7	Caesarean deliveries performed in hospitals (No.) by Geographic localization, Annual Live births (No.) by Sex; annual [online database]. Lisbon: Instituto Nacional de Estadística (INE) [Portugal]; 2012 (http://www.ine.pt, accessed 25 September 2012).
PRT	Portugal	2007	2007	34.9	Caesarean deliveries performed in hospitals (No.) by Geographic localization, Annual Live births (No.) by Sex; annual [online database]. Lisbon: Instituto Nacional de Estadística (INE) [Portugal]; 2012 (http://www.ine.pt, accessed 25 September 2012).
PRT	Portugal	2008	2008	35.6	Caesarean deliveries performed in hospitals (No.) by Geographic localization, Annual Live births (No.) by Sex; annual [online database]. Lisbon: Instituto Nacional de Estadística (INE) [Portugal]; 2012 (http://www.ine.pt, accessed 25 September 2012).
PRT	Portugal	2009	2009	36.2	Caesarean deliveries performed in hospitals (No.) by Geographic localization, Annual Live births (No.) by Sex; annual [online database]. Lisbon: Instituto Nacional de Estadística (INE) [Portugal]; 2012 (http://www.ine.pt, accessed 25 September 2012).
PRT	Portugal	2010	2010	35.8	Caesarean deliveries performed in hospitals (No.) by Geographic localization, Annual Live births (No.) by Sex; annual [online database]. Lisbon: Instituto Nacional de Estadística (INE) [Portugal]; 2012 (http://www.ine.pt, accessed 25 September 2012).
PRT	Portugal	2011	2011	35.2	Caesarean deliveries performed in hospitals (No.) by Geographic localization, Annual Live births (No.) by Sex; annual [online database]. Lisbon: Instituto Nacional de Estadística (INE) [Portugal]; 2013 (http://www.ine.pt, accessed 29 October 2013).
PRT	Portugal	2013	2013	35.6	Deliveries (No.) in hospitals by Geographic localization (NUTS - 2013) and type of delivery; Annual [online database]. Lisbon: Instituto Nacional de Estadística (INE) [Portugal]; 2019 (https://www.ine.pt/bddXplorer/htdocs/printable.jsp?id=0t3TbYPCjPkrRuDkjgqzV6Bh_61807&lingua_cd=EN, accessed 15 February 2019).
PRT	Portugal	2014	2014	33.5	Deliveries (No.) in hospitals by Geographic localization (NUTS - 2013) and type of delivery; Annual [online database]. Lisbon: Instituto Nacional de Estadística (INE) [Portugal]; 2019 (https://www.ine.pt/bddXplorer/htdocs/printable.jsp?id=0t3TbYPCjPkrRuDkjgqzV6Bh_61807&lingua_cd=EN, accessed 15 February 2019).

ISO Code	Country	Coverage start year	Coverage end year	Caesarean section rate (%)	References
PRT	Portugal	2015	2015	32.9	Deliveries (No.) in hospitals by Geographic localization (NUTS - 2013) and type of delivery; Annual [online database]. Lisbon: Instituto Nacional de Estadística (INE) [Portugal]; 2019 (https://www.ine.pt/bddXplorer/htdocs/printable.jsp?id=0t3TbYPCjPkrRuDkjgqzV6Bh_61807&lingua_cd=EN, accessed 15 February 2019).
PRT	Portugal	2016	2016	33.1	Deliveries (No.) in hospitals by Geographic localization (NUTS - 2013) and type of delivery; Annual [online database]. Lisbon: Instituto Nacional de Estadística (INE) [Portugal]; 2019 (https://www.ine.pt/bddXplorer/htdocs/printable.jsp?id=0t3TbYPCjPkrRuDkjgqzV6Bh_61807&lingua_cd=EN, accessed 15 February 2019).
PRT	Portugal	2017	2017	33.1	Deliveries (No.) in hospitals by Geographic localization (NUTS - 2013) and type of delivery; Annual [online database]. Lisbon: Instituto Nacional de Estadística (INE) [Portugal]; 2019 (https://www.ine.pt/bddXplorer/htdocs/printable.jsp?id=0t3TbYPCjPkrRuDkjgqzV6Bh_61807&lingua_cd=EN, accessed 15 February 2019).
PRT	Portugal	2018	2018	34.1	Deliveries (No.) in hospitals by Geographic localization (NUTS - 2013) and type of delivery; Annual [online database]. Lisbon: Instituto Nacional de Estadística (INE) [Portugal]; 2020 (https://www.ine.pt/xportal/xmain?xpid=INE&xpgid=ine_indicadores&indOcorrCod=0008109&contexto=bd&selTab=tab2, accessed 5 March 2020).
QAT	Qatar	1993	1998	15.9	Jurdi R, Khawaja M. Caesarean section rates in the Arab region: a cross-national study. Health Policy Plan. 2004;19(2):101-10.
QAT	Qatar	2010	2012	19.5	Multiple Indicator Cluster Survey in the State of Qatar 2012. Doha: Ministry of Development Planning and Statistics, Qatar Foundation, Supreme Council of Health, United Nations Children's Fund (UNICEF); 2014.
KOR	Republic of Korea	1987	1991	17.3	The 2003 National Survey on Fertility, Family Health & Welfare in Korea. Sejong City: Korea Institute for Health and Social Affairs; 2004 (in Korean).
KOR	Republic of Korea	1990	1994	31.5	The 2003 National Survey on Fertility, Family Health & Welfare in Korea. Sejong City: Korea Institute for Health and Social Affairs; 2004 (in Korean).

ISO Code	Country	Coverage start year	Coverage end year	Caesarean section rate (%)	References
KOR	Republic of Korea	1995	1997	35.9	The 2003 National Survey on Fertility, Family Health & Welfare in Korea. Sejong City: Korea Institute for Health and Social Affairs; 2004 (in Korean).
KOR	Republic of Korea	1998	2000	37.7	The 2003 National Survey on Fertility, Family Health & Welfare in Korea. Sejong City: Korea Institute for Health and Social Affairs; 2004 (in Korean).
KOR	Republic of Korea	2001	2003	39.2	The 2003 National Survey on Fertility, Family Health & Welfare in Korea. Sejong City: Korea Institute for Health and Social Affairs; 2004 (in Korean).
KOR	Republic of Korea	2004	2006	35.0	The 2009 National Survey on Fertility, Family Health & Welfare in Korea. Sejong City: Korea Institute for Health and Social Affairs; 2009 (in Korean).
KOR	Republic of Korea	2007	2009	36.9	The 2009 National Survey on Fertility, Family Health & Welfare in Korea. Sejong City: Korea Institute for Health and Social Affairs; 2009 (in Korean).
KOR	Republic of Korea	2012	2012	34.3	The 2012 National Survey on Fertility, Family Health & Welfare in Korea. Sejong City: Korea Institute for Health and Social Affairs; 2012 (in Korean).
KOR	Republic of Korea	2013	2015	39.1	The 2015 National Survey on Fertility, Family Health & Welfare in Korea. Sejong City: Korea Institute for Health and Social Affairs; 2015 (in Korean).
MDA	Republic of Moldova	1990	1990	5.3	European Health for All Database (HFA-DB) [online database]. World Health Organization (WHO) Regional Office for Europe; 2012 (http://data.euro.who.int/hfadb, accessed 20 August 2012).

ISO Code	Country	Coverage start year	Coverage end year	Caesarean section rate (%)	References
MDA	Republic of Moldova	1991	1991	5.3	European Health for All Database (HFA-DB) [online database]. World Health Organization (WHO) Regional Office for Europe; 2012 (http://data.euro.who.int/hfadb, accessed 20 August 2012).
MDA	Republic of Moldova	1992	1992	5.0	European Health for All Database (HFA-DB) [online database]. World Health Organization (WHO) Regional Office for Europe; 2012 (http://data.euro.who.int/hfadb, accessed 20 August 2012).
MDA	Republic of Moldova	1993	1993	5.4	European Health for All Database (HFA-DB) [online database]. World Health Organization (WHO) Regional Office for Europe; 2012 (http://data.euro.who.int/hfadb, accessed 20 August 2012).
MDA	Republic of Moldova	1996	1996	6.0	European Health for All Database (HFA-DB) [online database]. World Health Organization (WHO) Regional Office for Europe; 2012 (http://data.euro.who.int/hfadb, accessed 20 August 2012).
MDA	Republic of Moldova	1992	1997	6.2	Moldovan Ministry of Health, Division of Reproductive Health, Centers for Disease Control and Prevention (DRH/CDC), United Nations Population Fund (UNFPA), United States Agency for International Development (USAID), United Nations Children’s Fund (UNICEF). Reproductive Health Survey Moldova, 1997. Final Report. Atlanta, Georgia: CDC; 1998.
MDA	Republic of Moldova	1997	1997	6.8	European Health for All Database (HFA-DB) [online database]. World Health Organization (WHO) Regional Office for Europe; 2012 (http://data.euro.who.int/hfadb, accessed 20 August 2012).
MDA	Republic of Moldova	1998	1998	7.7	European Health for All Database (HFA-DB) [online database]. World Health Organization (WHO) Regional Office for Europe; 2012 (http://data.euro.who.int/hfadb, accessed 20 August 2012).
MDA	Republic of Moldova	1999	1999	5.6	Sănătatea publică in Moldova anul 2000 [Public health in Moldova 2000]. Chișinău: Ministerul Sănătății al Republicii Moldova [Ministry of Health of the Republic of Moldova], Centrul Ştiinţifico-Practic Sănătate Publică şi Management Sanitar [Scientific and Practical Center for Public Health and Sanitary Management, Moldova]; 2001 (in Romanian).

ISO Code	Country	Coverage start year	Coverage end year	Caesarean section rate (%)	References
MDA	Republic of Moldova	2000	2000	6.4	Sănătatea publică in Moldova anul 2000 [Public health in Moldova 2000]. Chișinău: Ministerul Sănătății al Republicii Moldova [Ministry of Health of the Republic of Moldova], Centrul Ştiinţifico-Practic Sănătate Publică şi Management Sanitar [Scientific and Practical Center for Public Health and Sanitary Management, Moldova]; 2001 (in Romanian).
MDA	Republic of Moldova	2001	2001	6.8	Sănătatea publică in Moldova anul 2001 [Public health in Moldova 2001]. Chișinău: Ministerul Sănătății al Republicii Moldova [Ministry of Health of the Republic of Moldova], Centrul Ştiinţifico-Practic Sănătate Publică şi Management Sanitar [Scientific and Practical Center for Public Health and Sanitary Management, Moldova]; 2002 (in Romanian).
MDA	Republic of Moldova	2004	2004	9.3	Sănătatea publică in Moldova anul 2004 [Public health in Moldova 2004]. Chișinău: Ministerul Sănătății al Republicii Moldova [Ministry of Health of the Republic of Moldova], Centrul Ştiinţifico-Practic Sănătate Publică şi Management Sanitar [Scientific and Practical Center for Public Health and Sanitary Management, Moldova]; 2005 (in Romanian).
MDA	Republic of Moldova	2005	2005	10.1	Sănătatea publică in Moldova anul 2005 [Public health in Moldova 2005]. Chișinău: Ministerul Sănătății al Republicii Moldova [Ministry of Health of the Republic of Moldova], Centrul Ştiinţifico-Practic Sănătate Publică şi Management Sanitar [Scientific and Practical Center for Public Health and Sanitary Management, Moldova]; 2006 (in Romanian).
MDA	Republic of Moldova	2000	2005	8.5	National Scientific and Applied Center for Preventive Medicine (NCPM) [Moldova], ORC Macro. Moldova Demographic and Health Survey 2005. Calverton, Maryland: National Scientific and Applied Center for Preventive Medicine of the Ministry of Health and Social Protection, ORC Macro; 2006.
MDA	Republic of Moldova	2006	2006	11.3	Sănătatea publică in Moldova anul 2006 [Public health in Moldova 2006]. Chișinău: Ministerul Sănătății al Republicii Moldova [Ministry of Health of the Republic of Moldova], Centrul Ştiinţifico-Practic Sănătate Publică şi Management Sanitar [Scientific and Practical Center for Public Health and Sanitary Management, Moldova]; 2007 (in Romanian).
MDA	Republic of Moldova	2007	2007	12.1	Sănătatea publică in Moldova anul 2007 [Public health in Moldova 2007]. Chișinău: Ministerul Sănătății al Republicii Moldova [Ministry of Health of the Republic of Moldova], Centrul Național de Management în Sănătate [National Centre for Health Management, Moldova]; 2008 (in Romanian).
MDA	Republic of Moldova	2008	2008	13.6	Sănătatea publică in Moldova anul 2008 [Public health in Moldova 2008]. Chișinău: Ministerul Sănătății al Republicii Moldova [Ministry of Health of the Republic of Moldova], Centrul Național de Management în Sănătate [National Centre for Health Management, Moldova]; 2009 (in Romanian).

ISO Code	Country	Coverage start year	Coverage end year	Caesarean section rate (%)	References
MDA	Republic of Moldova	2009	2009	13.4	Sănătatea publică in Moldova anul 2009 [Public health in Moldova 2009]. Chișinău: Ministerul Sănătății al Republicii Moldova [Ministry of Health of the Republic of Moldova], Centrul Național de Management în Sănătate [National Centre for Health Management, Moldova]; 2010 (in Romanian).
MDA	Republic of Moldova	2010	2010	14.1	Anuarul statistic al sistemului de sănătate din Moldova anul 2010 [Statistical yearbook of the health system in Moldova in 2010]. Chișinău: Ministerul Sănătății al Republicii Moldova [Ministry of Health of the Republic of Moldova], Centrul Național de Management în Sănătate [National Centre for Health Management, Moldova]; 2011 (in Romanian).
MDA	Republic of Moldova	2010	2012	16.2	2012 Republic of Moldova Multiple Indicator Cluster Survey, Final Report. Chișinău, Republic of Moldova: National Centre of Public Health of the Ministry of Health of the Republic of Moldova, United Nations Children's Fund (UNICEF); 2014.
MDA	Republic of Moldova	2012	2012	15.6	Indicatori preliminari în format prescurtat privind sănătatea populaţiei şi activitatea instituţiilor medico-sanitare pe anii 2011-2012. Chișinău: Ministerul Sănătății al Republicii Moldova [Ministry of Health of the Republic of Moldova], Centrul Național de Management în Sănătate [National Centre for Health Management, Moldova]; 2013 (in Romanian).
MDA	Republic of Moldova	2013	2013	17.0	European Health for All Database (HFA-DB) [online database]. World Health Organization (WHO) Regional Office for Europe; 2016 (http://data.euro.who.int/hfadb, accessed 8 August 2016).
MDA	Republic of Moldova	2014	2014	18.4	European Health for All Database (HFA-DB) [online database]. World Health Organization (WHO) Regional Office for Europe; 2016 (http://data.euro.who.int/hfadb, accessed 8 August 2016).
MDA	Republic of Moldova	2015	2015	18.5	European Health Information Gateway. European Health for All database (HFA-DB). Caesarean sections per 1000 live births [online database]. World Health Organization (WHO) Regional Office for Europe; 2018 (https://gateway.euro.who.int/en/indicators/hfa_596-7060-caesarean-sections-per-1000-live-births/, accessed 14 February 2019).
ROU	Romania	1992	1992	7.2	European Health for All Database (HFA-DB) [online database]. World Health Organization (WHO) Regional Office for Europe; 2012 (http://data.euro.who.int/hfadb, accessed 20 August 2012).

ISO Code	Country	Coverage start year	Coverage end year	Caesarean section rate (%)	References
ROU	Romania	1993	1993	8.6	European Health for All Database (HFA-DB) [online database]. World Health Organization (WHO) Regional Office for Europe; 2012 (http://data.euro.who.int/hfadb, accessed 20 August 2012).
ROU	Romania	1994	1994	9.1	European Health for All Database (HFA-DB) [online database]. World Health Organization (WHO) Regional Office for Europe; 2012 (http://data.euro.who.int/hfadb, accessed 20 August 2012).
ROU	Romania	1995	1995	10.1	European Health for All Database (HFA-DB) [online database]. World Health Organization (WHO) Regional Office for Europe; 2012 (http://data.euro.who.int/hfadb, accessed 20 August 2012).
ROU	Romania	1998	1998	12.0	European Health for All Database (HFA-DB) [online database]. World Health Organization (WHO) Regional Office for Europe; 2012 (http://data.euro.who.int/hfadb, accessed 20 August 2012).
ROU	Romania	1994	1999	11.1	Reproductive Health Survey Romania, 1999. Final Report. Atlanta, Georgia: Centers for Disease Control and Prevention (DRH/CDC), Romanian Association of Public Health and Health Management (ARSPMS), United Nations Population Fund (UNFPA), United States Agency for International Development (USAID), United Nations Children's Fund (UNICEF); 2001.
ROU	Romania	1999	1999	12.5	European Health for All Database (HFA-DB) [online database]. World Health Organization (WHO) Regional Office for Europe; 2012 (http://data.euro.who.int/hfadb, accessed 20 August 2012).
ROU	Romania	2000	2000	14.1	European Health for All Database (HFA-DB) [online database]. World Health Organization (WHO) Regional Office for Europe; 2012 (http://data.euro.who.int/hfadb, accessed 20 August 2012).
ROU	Romania	2003	2003	17.8	European Health for All Database (HFA-DB) [online database]. World Health Organization (WHO) Regional Office for Europe; 2012 (http://data.euro.who.int/hfadb, accessed 20 August 2012).

ISO Code	Country	Coverage start year	Coverage end year	Caesarean section rate (%)	References
ROU	Romania	1999	2004	19.1	European Health for All Database (HFA-DB) [online database]. World Health Organization (WHO) Regional Office for Europe; 2012 (http://data.euro.who.int/hfadb, accessed 20 August 2012).
ROU	Romania	2004	2004	18.9	European Health for All Database (HFA-DB) [online database]. World Health Organization (WHO) Regional Office for Europe; 2012 (http://data.euro.who.int/hfadb, accessed 20 August 2012).
ROU	Romania	2005	2005	21.4	European Health for All Database (HFA-DB) [online database]. World Health Organization (WHO) Regional Office for Europe; 2012 (http://data.euro.who.int/hfadb, accessed 20 August 2012).
ROU	Romania	2006	2006	21.5	European Health for All Database (HFA-DB) [online database]. World Health Organization (WHO) Regional Office for Europe; 2012 (http://data.euro.who.int/hfadb, accessed 20 August 2012).
ROU	Romania	2007	2007	23.6	European Health for All Database (HFA-DB) [online database]. World Health Organization (WHO) Regional Office for Europe; 2012 (http://data.euro.who.int/hfadb, accessed 20 August 2012).
ROU	Romania	2008	2008	25.8	European Health for All Database (HFA-DB) [online database]. World Health Organization (WHO) Regional Office for Europe; 2012 (http://data.euro.who.int/hfadb, accessed 20 August 2012).
ROU	Romania	2009	2009	28.6	European Health for All Database (HFA-DB) [online database]. World Health Organization (WHO) Regional Office for Europe; 2012 (http://data.euro.who.int/hfadb, accessed 20 August 2012).
ROU	Romania	2010	2010	33.8	European Health for All Database (HFA-DB) [online database]. World Health Organization (WHO) Regional Office for Europe; 2016 (http://data.euro.who.int/hfadb, accessed 8 August 2016).

ISO Code	Country	Coverage start year	Coverage end year	Caesarean section rate (%)	References
ROU	Romania	2011	2011	36.3	European Health for All Database (HFA-DB) [online database]. World Health Organization (WHO) Regional Office for Europe; 2016 (http://data.euro.who.int/hfadb, accessed 8 August 2016).
ROU	Romania	2012	2012	37.3	European Health for All Database (HFA-DB) [online database]. World Health Organization (WHO) Regional Office for Europe; 2016 (http://data.euro.who.int/hfadb, accessed 8 August 2016).
ROU	Romania	2013	2013	38.3	European Health for All Database (HFA-DB) [online database]. World Health Organization (WHO) Regional Office for Europe; 2016 (http://data.euro.who.int/hfadb, accessed 8 August 2016).
ROU	Romania	2015	2015	46.9	European Perinatal Health Report. Core indicators of the health and care of pregnant women and babies in Europe in 2015. Euro-Peristat Project; 2018.
RUS	Russian Federation	1990	1990	6.7	European Health for All Database (HFA-DB) [online database]. World Health Organization (WHO) Regional Office for Europe; 2012 (http://data.euro.who.int/hfadb, accessed 20 August 2012).
RUS	Russian Federation	1991	1991	7.2	European Health for All Database (HFA-DB) [online database]. World Health Organization (WHO) Regional Office for Europe; 2012 (http://data.euro.who.int/hfadb, accessed 20 August 2012).
RUS	Russian Federation	1992	1992	8.1	European Health for All Database (HFA-DB) [online database]. World Health Organization (WHO) Regional Office for Europe; 2012 (http://data.euro.who.int/hfadb, accessed 20 August 2012).
RUS	Russian Federation	1993	1993	9.0	European Health for All Database (HFA-DB) [online database]. World Health Organization (WHO) Regional Office for Europe; 2012 (http://data.euro.who.int/hfadb, accessed 20 August 2012).

ISO Code	Country	Coverage start year	Coverage end year	Caesarean section rate (%)	References
RUS	Russian Federation	1994	1994	9.4	European Health for All Database (HFA-DB) [online database]. World Health Organization (WHO) Regional Office for Europe; 2012 (http://data.euro.who.int/hfadb, accessed 20 August 2012).
RUS	Russian Federation	1995	1995	9.9	European Health for All Database (HFA-DB) [online database]. World Health Organization (WHO) Regional Office for Europe; 2012 (http://data.euro.who.int/hfadb, accessed 20 August 2012).
RUS	Russian Federation	1996	1996	10.7	European Health for All Database (HFA-DB) [online database]. World Health Organization (WHO) Regional Office for Europe; 2012 (http://data.euro.who.int/hfadb, accessed 20 August 2012).
RUS	Russian Federation	1997	1997	11.8	European Health for All Database (HFA-DB) [online database]. World Health Organization (WHO) Regional Office for Europe; 2012 (http://data.euro.who.int/hfadb, accessed 20 August 2012).
RUS	Russian Federation	1998	1998	12.6	European Health for All Database (HFA-DB) [online database]. World Health Organization (WHO) Regional Office for Europe; 2012 (http://data.euro.who.int/hfadb, accessed 20 August 2012).
RUS	Russian Federation	1999	1999	13.1	European Health for All Database (HFA-DB) [online database]. World Health Organization (WHO) Regional Office for Europe; 2012 (http://data.euro.who.int/hfadb, accessed 20 August 2012).
RUS	Russian Federation	2000	2000	14.0	European Health for All Database (HFA-DB) [online database]. World Health Organization (WHO) Regional Office for Europe; 2012 (http://data.euro.who.int/hfadb, accessed 20 August 2012).
RUS	Russian Federation	2001	2001	14.8	European Health for All Database (HFA-DB) [online database]. World Health Organization (WHO) Regional Office for Europe; 2012 (http://data.euro.who.int/hfadb, accessed 20 August 2012).

ISO Code	Country	Coverage start year	Coverage end year	Caesarean section rate (%)	References
RUS	Russian Federation	2002	2002	15.2	European Health for All Database (HFA-DB) [online database]. World Health Organization (WHO) Regional Office for Europe; 2012 (http://data.euro.who.int/hfadb, accessed 20 August 2012).
RUS	Russian Federation	2003	2003	15.7	European Health for All Database (HFA-DB) [online database]. World Health Organization (WHO) Regional Office for Europe; 2012 (http://data.euro.who.int/hfadb, accessed 20 August 2012).
RUS	Russian Federation	2004	2004	16.2	European Health for All Database (HFA-DB) [online database]. World Health Organization (WHO) Regional Office for Europe; 2012 (http://data.euro.who.int/hfadb, accessed 20 August 2012).
RUS	Russian Federation	2005	2005	17.2	European Health for All Database (HFA-DB) [online database]. World Health Organization (WHO) Regional Office for Europe; 2012 (http://data.euro.who.int/hfadb, accessed 20 August 2012).
RUS	Russian Federation	2006	2006	18.0	European Health for All Database (HFA-DB) [online database]. World Health Organization (WHO) Regional Office for Europe; 2012 (http://data.euro.who.int/hfadb, accessed 20 August 2012).
RUS	Russian Federation	2010	2010	22.1	European Health for All Database (HFA-DB) [online database]. World Health Organization (WHO) Regional Office for Europe; 2016 (http://data.euro.who.int/hfadb, accessed 8 August 2016).
RUS	Russian Federation	2011	2011	22.6	European Health for All Database (HFA-DB) [online database]. World Health Organization (WHO) Regional Office for Europe; 2016 (http://data.euro.who.int/hfadb, accessed 8 August 2016).
RUS	Russian Federation	2006	2011	13.0	Federal State Statistic Service (ROSSTAT), Ministry of Health of the Russian Federation, Information and Publishing Center "Statistics of Russia". Reproductive Health Survey Russia 2011. Full Report. Atlanta: United Nations Population Fund (UNFPA), Division of Reproductive Health, Centers for Disease Control and Prevention (DRH/CDC); 2012 (in Russian).

ISO Code	Country	Coverage start year	Coverage end year	Caesarean section rate (%)	References
RUS	Russian Federation	2013	2013	25.1	European Health Information Gateway. European Health for All database (HFA-DB). Caesarean sections per 1000 live births [online database]. World Health Organization (WHO) Regional Office for Europe; 2018 (https://gateway.euro.who.int/en/indicators/hfa_596-7060-caesarean-sections-per-1000-live-births/, accessed 14 February 2019).
RUS	Russian Federation	2014	2014	26.2	European Health Information Gateway. European Health for All database (HFA-DB). Caesarean sections per 1000 live births [online database]. World Health Organization (WHO) Regional Office for Europe; 2018 (https://gateway.euro.who.int/en/indicators/hfa_596-7060-caesarean-sections-per-1000-live-births/, accessed 14 February 2019).
RUS	Russian Federation	2015	2015	27.0	European Health Information Gateway. European Health for All database (HFA-DB). Caesarean sections per 1000 live births [online database]. World Health Organization (WHO) Regional Office for Europe; 2018 (https://gateway.euro.who.int/en/indicators/hfa_596-7060-caesarean-sections-per-1000-live-births/, accessed 14 February 2019).
RUS	Russian Federation	2017	2017	20.8	European Health Information Gateway. European Health for All database (HFA-DB). Caesarean sections per 1000 live births [online database]. World Health Organization (WHO) Regional Office for Europe; 2019 (https://gateway.euro.who.int/en/indicators/hfa_596-7060-caesarean-sections-per-1000-live-births/, accessed 5 March 2020).
RWA	Rwanda	1987	1992	1.8	Barrère B, Schoemaker J, Barrère M, Habiyakare T, Kabagwira A, Ngendakumana M (Office National de la Population, Macro International). Enquête Démographique et de Santé Rwanda 1992. Kigali, Rwanda: Office National de la Population, Macro International; 1994 (in French).
RWA	Rwanda	1995	2000	2.1	Office National de la Population (ONAPO) [Rwanda], ORC Macro. Enquête Démographique et de Santé, Rwanda 2000. Kigali, Rwanda: Ministère de la Santé, Office National de la Population, ORC Macro; 2001 (in French).
RWA	Rwanda	2000	2005	2.9	Institut National de la Statistique du Rwanda (INSR), ORC Macro. Rwanda Demographic and Health Survey 2005. Calverton, Maryland, USA: INSR, ORC Macro; 2006.
RWA	Rwanda	2005	2010	7.1	National Institute of Statistics of Rwanda (NISR), Ministry of Health (MOH) [Rwanda], ICF International. Rwanda Demographic and Health Survey 2010. Calverton, Maryland, USA: NISR [Rwanda], MOH [Rwanda], ICF International; 2012.

ISO Code	Country	Coverage start year	Coverage end year	Caesarean section rate (%)	References
RWA	Rwanda	2010	2015	13.0	National Institute of Statistics of Rwanda, Ministry of Finance and Economic Planning [Rwanda], Ministry of Health [Rwanda], ICF International. Rwanda Demographic and Health Survey 2014-15. Kigali, Rwanda: National Institute of Statistics of Rwanda, Ministry of Finance and Economic Planning [Rwanda], Ministry of Health [Rwanda], ICF International; 2015.
LCA	Saint Lucia	2010	2012	18.5	Ministry of Social Transformation, Local Government and Community Empowerment, Central Statistics Office, United Nations Children's Fund (UNICEF). Saint Lucia Multiple Indicator Cluster Survey 2012: Final Report. Castries, Saint Lucia: Ministry of Social Transformation, Local Government and Community Empowerment, Central Statistics Office, United Nations Children's Fund (UNICEF); 2014.
WSM	Samoa	2004	2009	12.8	Ministry of Health [Samoa], Bureau of Statistics [Samoa], ICF Macro. Samoa Demographic and Health Survey 2009. Apia, Samoa: Ministry of Health [Samoa]; 2010.
WSM	Samoa	2009	2014	4.6	Census-Surveys and Demography Division, Samoa Bureau of Statistics, Ministry of Health [Samoa], United Nations Population Fund (UNFPA), United Nations Children's Fund (UNICEF), Department of Foreign Affairs and Trade [Australia]. Samoa Demographic and Health Survey 2014. Apia: Samoa Bureau of Statistics; 2015.
SMR	San Marino	2009	2009	31.4	Supplemento al Bollettino di Statistica Anno 2009. Borgo Maggiore: Ufficio Informatica, Tecnologia, Dati e Statistica [San Marino]; 2010 (in Italian).
SMR	San Marino	2010	2010	33.2	Supplemento al Bollettino di Statistica Anno 2010. Borgo Maggiore: Ufficio Informatica, Tecnologia, Dati e Statistica [San Marino]; 2009 (in Italian).
SMR	San Marino	2011	2011	35.4	European Health for All Database (HFA-DB) [online database]. World Health Organization (WHO) Regional Office for Europe; 2016 (http://data.euro.who.int/hfadb, accessed 8 August 2016).
SMR	San Marino	2011	2011	35.4	Supplemento al Bollettino di Statistica Anno 2011. Borgo Maggiore: Ufficio Informatica, Tecnologia, Dati e Statistica [San Marino]; 2010 (in Italian).

ISO Code	Country	Coverage start year	Coverage end year	Caesarean section rate (%)	References
SMR	San Marino	2012	2012	33.6	European Health for All Database (HFA-DB) [online database]. World Health Organization (WHO) Regional Office for Europe; 2016 (http://data.euro.who.int/hfadb, accessed 8 August 2016).
SMR	San Marino	2012	2012	33.6	Supplemento al Bollettino di Statistica Anno 2012. Borgo Maggiore: Ufficio Informatica, Tecnologia, Dati e Statistica [San Marino]; 2011 (in Italian).
SMR	San Marino	2013	2013	30.9	European Health for All Database (HFA-DB) [online database]. World Health Organization (WHO) Regional Office for Europe; 2016 (http://data.euro.who.int/hfadb, accessed 8 August 2016).
SMR	San Marino	2013	2013	30.9	Supplemento al Bollettino di Statistica Anno 2013. Borgo Maggiore: Ufficio Informatica, Tecnologia, Dati e Statistica [San Marino]; 2014 (in Italian).
SMR	San Marino	2014	2014	21.3	European Health for All Database (HFA-DB) [online database]. World Health Organization (WHO) Regional Office for Europe; 2016 (http://data.euro.who.int/hfadb, accessed 8 August 2016).
SMR	San Marino	2014	2014	21.3	Supplemento al Bollettino di Statistica Anno 2015. Borgo Maggiore: Ufficio Informatica, Tecnologia, Dati e Statistica [San Marino]; 2016 (in Italian).
SMR	San Marino	2015	2015	22.8	Supplemento al Bollettino di Statistica Anno 2015. Borgo Maggiore: Ufficio Informatica, Tecnologia, Dati e Statistica [San Marino]; 2016 (in Italian).
SMR	San Marino	2016	2016	27.5	Supplemento al Bollettino di Statistica Anno 2016. Borgo Maggiore: Ufficio Informatica, Tecnologia, Dati e Statistica [San Marino]; 2017 (in Italian).

ISO Code	Country	Coverage start year	Coverage end year	Caesarean section rate (%)	References
SMR	San Marino	2017	2017	23.2	European Health Information Gateway. European Health for All database (HFA-DB). Caesarean sections per 1000 live births [online database]. World Health Organization (WHO) Regional Office for Europe; 2019 (https://gateway.euro.who.int/en/indicators/hfa_596-7060-caesarean-sections-per-1000-live-births/, accessed 5 March 2020).
STP	Sao Tome and Principe	2003	2009	5.3	Instituto Nacional de Estatística (INE) [São Tomé e Príncipe], Ministério da Saúde, ICF Macro. Inquérito Demográfico e Sanitário, São Tomé e Príncipe, IDS STP, 2008-2009. Calverton, Maryland, USA: INE; 2010 (in Portuguese).
STP	Sao Tome and Principe	2012	2014	5.6	National Institute of Statistics, United Nations Children's Fund (UNICEF). Sao Tome and Principe Multiple Indicator Cluster Survey 2014, Final Report. São Tomé, São Tomé and Príncipe: National Institute of Statistics; 2016.
SAU	Saudi Arabia	2006	2006	18.5	Health Statistical Year Book Kingdom of Saudi Arabia 2006 (1427). Riyadh: Ministry of Health [Saudi Arabia]; 2007.
SAU	Saudi Arabia	2007	2007	19.2	Health Statistical Year Book 2007 (1428). Riyadh: Ministry of Health [Saudi Arabia]; 2008.
SAU	Saudi Arabia	2008	2008	20.4	Health Statistics Book for the Year of 2008 (1429). Riyadh: Ministry of Health [Saudi Arabia]; 2009.
SAU	Saudi Arabia	2009	2009	21.2	Health Statistical Year Book Kingdom of Saudi Arabia 1430/2009. Riyadh: Ministry of Health [Saudi Arabia]; 2010.
SAU	Saudi Arabia	2010	2010	22.3	Health Statistical Year Book Kingdom of Saudi Arabia 1431/2010. Riyadh: Ministry of Health [Saudi Arabia]; 2011.

Betran AP, *et al. BMJ Global Health* 2021; 6:e005671. doi: 10.1136/bmjgh-2021-005671

ISO Code	Country	Coverage start year	Coverage end year	Caesarean section rate (%)	References
SAU	Saudi Arabia	2012	2012	22.3	Health Statistical Year Book Kingdom of Saudi Arabia 1432/2012. Riyadh: Ministry of Health [Saudi Arabia]; 2013.
SAU	Saudi Arabia	2013	2013	25.2	Health Statistics Annual Book Kingdom of Saudi Arabia 2013 G/1434 H. Riyadh: Ministry of Health, General Directorate of Statistics & Information [Saudi Arabia]; 2014.
SAU	Saudi Arabia	2014	2014	23.6	Statistical Year Book Kingdom of Saudi Arabia 2014 G/1435 H. Riyadh: Ministry of Health [Saudi Arabia]; 2015.
SAU	Saudi Arabia	2015	2015	22.7	Statistical Year Book Kingdom of Saudi Arabia 2015 G/1436 H. Riyadh: Ministry of Health [Saudi Arabia]; 2016.
SAU	Saudi Arabia	2016	2016	30.2	Statistical Year Book Kingdom of Saudi Arabia 2016 G/1437 H. Riyadh: Ministry of Health [Saudi Arabia]; 2017.
SAU	Saudi Arabia	2017	2017	30.4	Statistical Year Book Kingdom of Saudi Arabia 2017 G/1438 H. Riyadh: Ministry of Health [Saudi Arabia]; 2018.
SAU	Saudi Arabia	2018	2018	32.0	Statistical Year Book Kingdom of Saudi Arabia 2018 G/1439 H. Riyadh: Ministry of Health [Saudi Arabia]; 2019.
SEN	Senegal	1987	1993	2.3	Salif N, Diouf PD, Ayad M (Ministère de l'Economie, des Finances et du Plan [Sénégal], Macro International). Enquête Démographique et de Santé au Sénégal (EDS-II) 1992/93. Dakar, Sénégal: Ministère de l'Economie, des Finances et du Plan [Sénégal], Macro International; 1994 (in French).

ISO Code	Country	Coverage start year	Coverage end year	Caesarean section rate (%)	References
SEN	Senegal	2000	2005	3.3	Ndiaye S, Ayad M (Centre de Recherche pour le Développement Humain [Sénégal], ORC Macro). Enquête Démographique et de Santé au Sénégal 2005. Calverton, Maryland, USA: Centre de Recherche pour le Développement Humain [Sénégal], ORC Macro; 2006 (in French).
SEN	Senegal	2005	2011	5.9	Agence Nationale de la Statistique et de la Démographie (ANSD) [Sénégal], ICF International. Enquête Démographique et de Santé à Indicateurs Multiples au Sénégal (EDS-MICS) 2010-2011. Calverton, Maryland, USA: ANSD, ICF International; 2012 (in French).
SEN	Senegal	2007	2013	3.8	Agence Nationale de la Statistique et de la Démographie (ANSD) [Sénégal], ICF International. Enquête Démographique et de Santé Continue (EDS-Continue 2012-2013). Calverton, Maryland, USA: ANSD, ICF International; 2013 (in French).
SEN	Senegal	2007	2014	4.4	Agence Nationale de la Statistique et de la Démographie (ANSD) [Sénégal], ICF International. Sénégal : Enquête Démographique et de Santé Continue (EDS-Continue 2014). Rockville, Maryland, USA : ANSD, ICF International; 2015 (in French).
SEN	Senegal	2011	2016	5.5	Agence Nationale de la Statistique et de la Démographie (ANSD) [Sénégal], ICF. Sénégal: Enquête Démographique et de Santé Continue (EDS-Continue) 2016. Rockville, Maryland, USA: ANSD, ICF; 2017 (in French).
SEN	Senegal	2012	2017	4.6	Sénégal: Enquête Démographique et de Santé Continue (EDS-Continue) 2017. Rockville, Maryland, USA: Agence Nationale de la Statistique et de la Démographie (ANSD), ICF; 2018 (in French).
SRB	Serbia	2000	2000	8.0	European Health for All Database (HFA-DB) [online database]. World Health Organization (WHO) Regional Office for Europe; 2012 (http://data.euro.who.int/hfadb, accessed 20 August 2012).
SRB	Serbia	2001	2001	8.6	Health Statistical Yearbook of Republic of Serbia 2005. Belgrade: Institute of Public Health of Serbia; 2006.

ISO Code	Country	Coverage start year	Coverage end year	Caesarean section rate (%)	References
SRB	Serbia	2002	2002	7.5	Health Statistical Yearbook of Republic of Serbia 2005. Belgrade: Institute of Public Health of Serbia; 2006.
SRB	Serbia	2003	2003	9.3	Health Statistical Yearbook of Republic of Serbia 2005. Belgrade: Institute of Public Health of Serbia; 2006.
SRB	Serbia	2004	2004	10.8	Health Statistical Yearbook of Republic of Serbia 2005. Belgrade: Institute of Public Health of Serbia; 2006.
SRB	Serbia	2005	2005	11.6	Health Statistical Yearbook of Republic of Serbia 2005. Belgrade: Institute of Public Health of Serbia; 2006.
SRB	Serbia	2006	2006	16.5	European Health for All Database (HFA-DB) [online database]. World Health Organization (WHO) Regional Office for Europe; 2012 (http://data.euro.who.int/hfadb, accessed 20 August 2012).
SRB	Serbia	2007	2007	18.1	European Health for All Database (HFA-DB) [online database]. World Health Organization (WHO) Regional Office for Europe; 2012 (http://data.euro.who.int/hfadb, accessed 20 August 2012).
SRB	Serbia	2008	2008	19.3	European Health for All Database (HFA-DB) [online database]. World Health Organization (WHO) Regional Office for Europe; 2012 (http://data.euro.who.int/hfadb, accessed 20 August 2012).
SRB	Serbia	2008	2010	24.6	Statistical Office of the Republic of Serbia, United Nations Children's Fund (UNICEF). Republic of Serbia Multiple Indicator Cluster Survey 2010, Final Report. Belgrade, Republic of Serbia: Statistical Office of the Republic of Serbia; 2011.

ISO Code	Country	Coverage start year	Coverage end year	Caesarean section rate (%)	References
SRB	Serbia	2010	2010	23.9	European Health for All Database (HFA-DB) [online database]. World Health Organization (WHO) Regional Office for Europe; 2016 (http://data.euro.who.int/hfadb, accessed 8 August 2016).
SRB	Serbia	2011	2011	25.8	European Health for All Database (HFA-DB) [online database]. World Health Organization (WHO) Regional Office for Europe; 2016 (http://data.euro.who.int/hfadb, accessed 8 August 2016).
SRB	Serbia	2012	2012	26.8	European Health for All Database (HFA-DB) [online database]. World Health Organization (WHO) Regional Office for Europe; 2016 (http://data.euro.who.int/hfadb, accessed 8 August 2016).
SRB	Serbia	2014	2014	29.7	European Health for All Database (HFA-DB) [online database]. World Health Organization (WHO) Regional Office for Europe; 2016 (http://data.euro.who.int/hfadb, accessed 8 August 2016).
SRB	Serbia	2012	2014	28.8	Statistical Office of the Republic of Serbia, UNICEF. Serbia Multiple Indicator Cluster Survey and Serbia Roma Settlements Multiple Indicator Cluster Survey, 2014, Final Report. Belgrade, Serbia: Statistical Office of the Republic of Serbia, UNICEF; 2014.
SYC	Seychelles	2005	2005	19.2	Statistical Abstract 2009. Victoria: National Bureau of Statistics [Seychelles]; 2010.
SYC	Seychelles	2006	2006	13.8	Statistical Abstract 2009. Victoria: National Bureau of Statistics [Seychelles]; 2010.
SYC	Seychelles	2007	2007	19.3	Statistical Abstract 2009. Victoria: National Bureau of Statistics [Seychelles]; 2010.

ISO Code	Country	Coverage start year	Coverage end year	Caesarean section rate (%)	References
SYC	Seychelles	2008	2008	16.0	Statistical Abstract 2009. Victoria: National Bureau of Statistics [Seychelles]; 2010.
SYC	Seychelles	2009	2009	20.3	Statistical Abstract 2009. Victoria: National Bureau of Statistics [Seychelles]; 2010.
SYC	Seychelles	2010	2010	22.8	Statistical Abstract 2010. Victoria: National Bureau of Statistics [Seychelles]; 2011.
SYC	Seychelles	2011	2011	17.8	Statistical Abstract 2014. Victoria: National Bureau of Statistics [Seychelles]; 2015.
SYC	Seychelles	2012	2012	25.3	Statistical Abstract 2014. Victoria: National Bureau of Statistics [Seychelles]; 2015.
SYC	Seychelles	2013	2013	24.3	Statistical Abstract 2014. Victoria: National Bureau of Statistics [Seychelles]; 2015.
SYC	Seychelles	2014	2014	24.9	Statistical Abstract 2014. Victoria: National Bureau of Statistics [Seychelles]; 2015.
SYC	Seychelles	2015	2015	28.2	Statistical Abstract 2016. Victoria: National Bureau of Statistics [Seychelles]; 2017.

ISO Code	Country	Coverage start year	Coverage end year	Caesarean section rate (%)	References
SYC	Seychelles	2016	2016	28.2	Statistical Abstract 2016. Victoria: National Bureau of Statistics [Seychelles]; 2017.
SYC	Seychelles	2017	2017	33.2	Statistical Abstract 2018. Victoria: National Bureau of Statistics [Seychelles]; 2019.
SYC	Seychelles	2018	2018	27.8	Statistical Abstract 2018. Victoria: National Bureau of Statistics [Seychelles]; 2019.
SLE	Sierra Leone	2003	2008	1.5	Statistics Sierra Leone (SSL), ICF Macro. Sierra Leone Demographic and Health Survey 2008. Calverton, Maryland, USA: Statistics Sierra Leone (SSL), ICF Macro; 2009.
SLE	Sierra Leone	2008	2010	4.5	Statistics Sierra Leone, United Nations Children's Fund (UNICEF) Sierra Leone. Sierra Leone Multiple Indicator Cluster Survey 2010, Final Report. Freetown, Sierra Leone: Statistics Sierra Leone, UNICEF Sierra Leone; 2011.
SLE	Sierra Leone	2008	2013	2.9	Statistics Sierra Leone (SSL), ICF International. Sierra Leone Demographic and Health Survey 2013. Freetown, Sierra Leone and Rockville, Maryland, USA: SSL, ICF International; 2014.
SLE	Sierra Leone	2012	2017	3.0	Statistics Sierra Leone, United Nations Children's Fund (UNICEF). Sierra Leone Multiple Indicator Cluster Survey 2017, Survey Findings Report. Freetown: Statistics Sierra Leone; 2018.
SGP	Singapore	2001	2003	30.5	Ganesan G. MOH Information Paper: 2004/06. Deliveries in Singapore - Volume and Resources. Singapore: Ministry of Health; 2004.

ISO Code	Country	Coverage start year	Coverage end year	Caesarean section rate (%)	References
SVK	Slovakia	1990	1990	8.7	Starostlivosť o ženu v SR 2004 (ZŠ-14/2005) [Caring for the woman in the SR 2004 (ZŠ-14/2005)]. Bratislava: Ústav zdravotníckych informácií a štatistiky [Institute of Health Information and Statistics]; 2005 (in Slovak).
SVK	Slovakia	1991	1991	9.2	Starostlivosť o ženu v SR 2010 (ZŠ-14/2011) [Caring for the woman in the SR 2010 (ZŠ-14/2011)]. Bratislava: Národné centrum zdravotníckych informácií [National Health Information Centre]; 2011 (in Slovak).
SVK	Slovakia	1992	1992	10.0	Starostlivosť o ženu v SR 2010 (ZŠ-14/2011) [Caring for the woman in the SR 2010 (ZŠ-14/2011)]. Bratislava: Národné centrum zdravotníckych informácií [National Health Information Centre]; 2011 (in Slovak).
SVK	Slovakia	1993	1993	10.6	Starostlivosť o ženu v SR 2010 (ZŠ-14/2011) [Caring for the woman in the SR 2010 (ZŠ-14/2011)]. Bratislava: Národné centrum zdravotníckych informácií [National Health Information Centre]; 2011 (in Slovak).
SVK	Slovakia	1994	1994	11.3	Starostlivosť o ženu v SR 2010 (ZŠ-14/2011) [Caring for the woman in the SR 2010 (ZŠ-14/2011)]. Bratislava: Národné centrum zdravotníckych informácií [National Health Information Centre]; 2011 (in Slovak).
SVK	Slovakia	1995	1995	11.6	Starostlivosť o ženu v SR 2010 (ZŠ-14/2011) [Caring for the woman in the SR 2010 (ZŠ-14/2011)]. Bratislava: Národné centrum zdravotníckych informácií [National Health Information Centre]; 2011 (in Slovak).
SVK	Slovakia	1996	1996	12.3	Starostlivosť o ženu v SR 2010 (ZŠ-14/2011) [Caring for the woman in the SR 2010 (ZŠ-14/2011)]. Bratislava: Národné centrum zdravotníckych informácií [National Health Information Centre]; 2011 (in Slovak).
SVK	Slovakia	1997	1997	13.1	Starostlivosť o ženu v SR 2010 (ZŠ-14/2011) [Caring for the woman in the SR 2010 (ZŠ-14/2011)]. Bratislava: Národné centrum zdravotníckych informácií [National Health Information Centre]; 2011 (in Slovak).

ISO Code	Country	Coverage start year	Coverage end year	Caesarean section rate (%)	References
SVK	Slovakia	1998	1998	13.4	Starostlivosť o ženu v SR 2010 (ZŠ-14/2011) [Caring for the woman in the SR 2010 (ZŠ-14/2011)]. Bratislava: Národné centrum zdravotníckych informácií [National Health Information Centre]; 2011 (in Slovak).
SVK	Slovakia	1999	1999	14.0	Starostlivosť o ženu v SR 2010 (ZŠ-14/2011) [Caring for the woman in the SR 2010 (ZŠ-14/2011)]. Bratislava: Národné centrum zdravotníckych informácií [National Health Information Centre]; 2011 (in Slovak).
SVK	Slovakia	2000	2000	14.9	Starostlivosť o ženu v SR 2010 (ZŠ-14/2011) [Caring for the woman in the SR 2010 (ZŠ-14/2011)]. Bratislava: Národné centrum zdravotníckych informácií [National Health Information Centre]; 2011 (in Slovak).
SVK	Slovakia	2001	2001	16.8	Starostlivosť o ženu v SR 2010 (ZŠ-14/2011) [Caring for the woman in the SR 2010 (ZŠ-14/2011)]. Bratislava: Národné centrum zdravotníckych informácií [National Health Information Centre]; 2011 (in Slovak).
SVK	Slovakia	2002	2002	18.1	Starostlivosť o ženu v SR 2010 (ZŠ-14/2011) [Caring for the woman in the SR 2010 (ZŠ-14/2011)]. Bratislava: Národné centrum zdravotníckych informácií [National Health Information Centre]; 2011 (in Slovak).
SVK	Slovakia	2003	2003	18.7	Starostlivosť o ženu v SR 2010 (ZŠ-14/2011) [Caring for the woman in the SR 2010 (ZŠ-14/2011)]. Bratislava: Národné centrum zdravotníckych informácií [National Health Information Centre]; 2011 (in Slovak).
SVK	Slovakia	2004	2004	19.4	Starostlivosť o ženu v SR 2010 (ZŠ-14/2011) [Caring for the woman in the SR 2010 (ZŠ-14/2011)]. Bratislava: Národné centrum zdravotníckych informácií [National Health Information Centre]; 2011 (in Slovak).
SVK	Slovakia	2005	2005	21.0	Starostlivosť o ženu v SR 2010 (ZŠ-14/2011) [Caring for the woman in the SR 2010 (ZŠ-14/2011)]. Bratislava: Národné centrum zdravotníckych informácií [National Health Information Centre]; 2011 (in Slovak).

ISO Code	Country	Coverage start year	Coverage end year	Caesarean section rate (%)	References
SVK	Slovakia	2006	2006	22.1	Starostlivosť o ženu v SR 2010 (ZŠ-14/2011) [Caring for the woman in the SR 2010 (ZŠ-14/2011)]. Bratislava: Národné centrum zdravotníckych informácií [National Health Information Centre]; 2011 (in Slovak).
SVK	Slovakia	2007	2007	23.7	Starostlivosť o ženu v SR 2010 (ZŠ-14/2011) [Caring for the woman in the SR 2010 (ZŠ-14/2011)]. Bratislava: Národné centrum zdravotníckych informácií [National Health Information Centre]; 2011 (in Slovak).
SVK	Slovakia	2008	2008	25.0	Starostlivosť o ženu v SR 2010 (ZŠ-14/2011) [Caring for the woman in the SR 2010 (ZŠ-14/2011)]. Bratislava: Národné centrum zdravotníckych informácií [National Health Information Centre]; 2011 (in Slovak).
SVK	Slovakia	2009	2009	27.3	Starostlivosť o ženu v SR 2010 (ZŠ-14/2011) [Caring for the woman in the SR 2010 (ZŠ-14/2011)]. Bratislava: Národné centrum zdravotníckych informácií [National Health Information Centre]; 2011 (in Slovak).
SVK	Slovakia	2010	2010	28.7	Starostlivosť o ženu v SR 2010 (ZŠ-14/2011) [Caring for the woman in the SR 2010 (ZŠ-14/2011)]. Bratislava: Národné centrum zdravotníckych informácií [National Health Information Centre]; 2011 (in Slovak).
SVK	Slovakia	2011	2011	29.5	Korbel M, Kristufkova A, Dugatova M, Danis J, Nemethova B, Kascak P et al. Analýza materskej morbidity a mortality v Slovenskej republike v rokoch 2007–2012. [Analysis of maternal morbidity and mortality in Slovak Republic in the years 2007-2012]. Ceska Gynekol. 2017;82(1):6-15 (in Slovak).
SVK	Slovakia	2012	2012	30.3	Korbel M, Kristufkova A, Dugatova M, Danis J, Nemethova B, Kascak P et al. Analýza materskej morbidity a mortality v Slovenskej republike v rokoch 2007–2012. [Analysis of maternal morbidity and mortality in Slovak Republic in the years 2007-2012]. Ceska Gynekol. 2017;82(1):6-15 (in Slovak).
SVK	Slovakia	2015	2015	31.1	European Health Information Gateway. European Health for All database (HFA-DB). Caesarean sections per 1000 live births [online database]. World Health Organization (WHO) Regional Office for Europe; 2018 (https://gateway.euro.who.int/en/indicators/hfa_596-7060-caesarean-sections-per-1000-live-births/, accessed 14 February 2019).

ISO Code	Country	Coverage start year	Coverage end year	Caesarean section rate (%)	References
SVN	Slovenia	1990	1990	8.6	European Health for All Database (HFA-DB) [online database]. World Health Organization (WHO) Regional Office for Europe; 2012 (http://data.euro.who.int/hfadb, accessed 20 August 2012).
SVN	Slovenia	1991	1991	8.9	European Health for All Database (HFA-DB) [online database]. World Health Organization (WHO) Regional Office for Europe; 2012 (http://data.euro.who.int/hfadb, accessed 20 August 2012).
SVN	Slovenia	1992	1992	9.1	European Health for All Database (HFA-DB) [online database]. World Health Organization (WHO) Regional Office for Europe; 2012 (http://data.euro.who.int/hfadb, accessed 20 August 2012).
SVN	Slovenia	1993	1993	9.6	European Health for All Database (HFA-DB) [online database]. World Health Organization (WHO) Regional Office for Europe; 2012 (http://data.euro.who.int/hfadb, accessed 20 August 2012).
SVN	Slovenia	1994	1994	9.4	European Health for All Database (HFA-DB) [online database]. World Health Organization (WHO) Regional Office for Europe; 2012 (http://data.euro.who.int/hfadb, accessed 20 August 2012).
SVN	Slovenia	1995	1995	9.2	European Health for All Database (HFA-DB) [online database]. World Health Organization (WHO) Regional Office for Europe; 2012 (http://data.euro.who.int/hfadb, accessed 20 August 2012).
SVN	Slovenia	1996	1996	10.0	European Health for All Database (HFA-DB) [online database]. World Health Organization (WHO) Regional Office for Europe; 2012 (http://data.euro.who.int/hfadb, accessed 20 August 2012).
SVN	Slovenia	1997	1997	10.2	European Health for All Database (HFA-DB) [online database]. World Health Organization (WHO) Regional Office for Europe; 2012 (http://data.euro.who.int/hfadb, accessed 20 August 2012).

ISO Code	Country	Coverage start year	Coverage end year	Caesarean section rate (%)	References
SVN	Slovenia	1998	1998	10.3	European Health for All Database (HFA-DB) [online database]. World Health Organization (WHO) Regional Office for Europe; 2012 (http://data.euro.who.int/hfadb, accessed 20 August 2012).
SVN	Slovenia	1999	1999	9.7	Perinatalni informacijski sistem Slovenije. Primerjava podatkov med regijami za leto 2003 [Perinatal Information System of the Republic of Slovenia. Comparison of data between regions for the year 2003]. Ljubljana: Inštitut za varovanje zdravja Republike Slovenije [National Institute of Public Health, Republic of Slovenia]; 2004 (in Slovene).
SVN	Slovenia	2000	2000	11.0	European Health for All Database (HFA-DB) [online database]. World Health Organization (WHO) Regional Office for Europe; 2012 (http://data.euro.who.int/hfadb, accessed 20 August 2012).
SVN	Slovenia	2001	2001	12.5	Perinatalni informacijski sistem Slovenije. Primerjava podatkov med regijami in porodnišnicami za leto 2001 [Perinatal Information System of the Republic of Slovenia. Comparison of data between regions and nursing homes for the year 2001]. Ljubljana: Inštitut za varovanje zdravja Republike Slovenije [National Institute of Public Health, Republic of Slovenia]; 2003 (in Slovene).
SVN	Slovenia	2002	2002	13.6	Perinatalni informacijski sistem Slovenije. Primerjava podatkov med regijami in porodnišnicami za leto 2002 [Perinatal Information System of the Republic of Slovenia. Comparison of data between regions and nursing homes for the year 2002]. Ljubljana: Inštitut za varovanje zdravja Republike Slovenije [National Institute of Public Health, Republic of Slovenia]; 2004 (in Slovene).
SVN	Slovenia	2003	2003	13.7	Perinatalni informacijski sistem Slovenije. Primerjava podatkov med regijami za leto 2003 [Perinatal Information System of the Republic of Slovenia. Comparison of data between regions for the year 2003]. Ljubljana: Inštitut za varovanje zdravja Republike Slovenije [National Institute of Public Health, Republic of Slovenia]; 2004 (in Slovene).
SVN	Slovenia	2004	2004	13.6	Perinatalni informacijski sistem Slovenije. Primerjava podatkov med regijami za leto 2004 [Perinatal Information System of the Republic of Slovenia. Comparison of data between regions for the year 2004]. Ljubljana: Inštitut za varovanje zdravja Republike Slovenije [National Institute of Public Health, Republic of Slovenia]; 2005 (in Slovene).
SVN	Slovenia	2005	2005	15.5	European Health for All Database (HFA-DB) [online database]. World Health Organization (WHO) Regional Office for Europe; 2012 (http://data.euro.who.int/hfadb, accessed 20 August 2012).

ISO Code	Country	Coverage start year	Coverage end year	Caesarean section rate (%)	References
SVN	Slovenia	2006	2006	16.4	European Health for All Database (HFA-DB) [online database]. World Health Organization (WHO) Regional Office for Europe; 2012 (http://data.euro.who.int/hfadb, accessed 20 August 2012).
SVN	Slovenia	2007	2007	16.8	European Health for All Database (HFA-DB) [online database]. World Health Organization (WHO) Regional Office for Europe; 2012 (http://data.euro.who.int/hfadb, accessed 20 August 2012).
SVN	Slovenia	2008	2008	18.7	European Health for All Database (HFA-DB) [online database]. World Health Organization (WHO) Regional Office for Europe; 2012 (http://data.euro.who.int/hfadb, accessed 20 August 2012).
SVN	Slovenia	2009	2009	17.3	Perinatalni informacijski sistem Republike Slovenije. Primerjava podatkov med regijami za leto 2009 [Perinatal Information System of the Republic of Slovenia. Comparison of data between regions for the year 2009]. Ljubljana: Inštitut za varovanje zdravja Republike Slovenije [National Institute of Public Health, Republic of Slovenia]; 2011 (in Slovene).
SVN	Slovenia	2010	2010	18.2	Perinatalni informacijski sistem Republike Slovenije. Primerjava podatkov med regijami za leto 2010 [Perinatal Information System of the Republic of Slovenia. Comparison of data between regions for the year 2010]. Ljubljana: Inštitut za varovanje zdravja Republike Slovenije [National Institute of Public Health, Republic of Slovenia]; 2012 (in Slovene).
SVN	Slovenia	2011	2011	19.6	European Health for All Database (HFA-DB) [online database]. World Health Organization (WHO) Regional Office for Europe; 2016 (http://data.euro.who.int/hfadb, accessed 8 August 2016).
SVN	Slovenia	2012	2012	18.9	Perinatalni informacijski sistem Republike Slovenije. Primerjava podatkov med regijami za leto 2012 [Perinatal Information System of the Republic of Slovenia. Comparison of data between regions for the year 2012]. Ljubljana: Inštitut za varovanje zdravja Republike Slovenije [National Institute of Public Health, Republic of Slovenia]; 2014 (in Slovene).
SVN	Slovenia	2013	2013	20.5	European Health for All Database (HFA-DB) [online database]. World Health Organization (WHO) Regional Office for Europe; 2016 (http://data.euro.who.int/hfadb, accessed 8 August 2016).

ISO Code	Country	Coverage start year	Coverage end year	Caesarean section rate (%)	References
SVN	Slovenia	2015	2015	21.2	European Perinatal Health Report. Core indicators of the health and care of pregnant women and babies in Europe in 2015. Euro-Peristat Project; 2018.
SLB	Solomon Islands	2001	2007	6.2	National Statistics Office [Solomon Islands], Secretariat of the Pacific Community (SPC), Macro International Inc., Asian Development Bank. Solomon Islands Demographic and Health Survey 2006-2007. Noumea: SPC; 2009.
SLB	Solomon Islands	2010	2015	5.9	Solomon Islands National Statistics Office, Solomon Islands Ministry of Health and Medical Services (SIMoHMS), Pacific Community (SPC), Australian Aid, United Nations Children’s Fund (UNICEF) . Solomon Islands Demographic and Health Survey 2015. Noumea: SPC; 2017.
ZAF	South Africa	1993	1998	15.5	Department of Health [South Africa], Macro International. South Africa Demographic and Health Survey 1998. Pretoria, South Africa: Department of Health [South Africa]; 2002.
ZAF	South Africa	1998	2003	20.6	Department of Health, Medical Research Council, ORC Macro. South Africa Demographic and Health Survey 2003. Pretoria, South Africa: Department of Health [South Africa]; 2007.
ZAF	South Africa	2011	2016	24.2	National Department of Health (NDoH), Statistics South Africa (Stats SA), South African Medical Research Council (SAMRC), ICF. South Africa Demographic and Health Survey 2016: Final Report. Pretoria, South Africa and Rockville, Maryland, USA: NDoH, Stats SA, SAMRC, ICF; 2019.
SSD	South Sudan	2008	2010	<1.0	The Republic of South Sudan: The Sudan Household Health Survey 2010. South Sudan: National Bureau of Statistics, Ministry of Health; 2012.
ESP	Spain	1990	1990	14.2	European Health for All Database (HFA-DB) [online database]. World Health Organization (WHO) Regional Office for Europe; 2012 (http://data.euro.who.int/hfadb, accessed 20 August 2012).

ISO Code	Country	Coverage start year	Coverage end year	Caesarean section rate (%)	References
ESP	Spain	1991	1991	15.0	European Health for All Database (HFA-DB) [online database]. World Health Organization (WHO) Regional Office for Europe; 2012 (http://data.euro.who.int/hfadb, accessed 20 August 2012).
ESP	Spain	1992	1992	16.2	European Health for All Database (HFA-DB) [online database]. World Health Organization (WHO) Regional Office for Europe; 2012 (http://data.euro.who.int/hfadb, accessed 20 August 2012).
ESP	Spain	1993	1993	17.3	European Health for All Database (HFA-DB) [online database]. World Health Organization (WHO) Regional Office for Europe; 2012 (http://data.euro.who.int/hfadb, accessed 20 August 2012).
ESP	Spain	1994	1994	17.8	European Health for All Database (HFA-DB) [online database]. World Health Organization (WHO) Regional Office for Europe; 2012 (http://data.euro.who.int/hfadb, accessed 20 August 2012).
ESP	Spain	1995	1995	18.8	European Health for All Database (HFA-DB) [online database]. World Health Organization (WHO) Regional Office for Europe; 2012 (http://data.euro.who.int/hfadb, accessed 20 August 2012).
ESP	Spain	1996	1996	19.3	European Health for All Database (HFA-DB) [online database]. World Health Organization (WHO) Regional Office for Europe; 2012 (http://data.euro.who.int/hfadb, accessed 20 August 2012).
ESP	Spain	1997	1997	19.8	Estadística de Establecimientos Sanitarios con Régimen de Internado. Tablas Estatales 97. 9.1 Actividad Obstétrica en los hospitales por dependencia 1997 [website]. Madrid: Ministerio de Sanidad, Servicios Sociales e Igualdad [Ministry of Health, Social Services and Equality, Spain]; 2012 (http://www.msssi.gob.es/estadEstudios/estadisticas/estHospiInternado/inforAnual/tab, accessed 8 October 2012, in Spanish).
ESP	Spain	1998	1998	20.5	Estadística de Establecimientos Sanitarios con Régimen de Internado 1998. España: Ministerio de Sanidad y Consumo, Dirección General de Salud Pública; 1999 (in Spanish).

ISO Code	Country	Coverage start year	Coverage end year	Caesarean section rate (%)	References
ESP	Spain	1999	1999	21.1	Estadística de Establecimientos Sanitarios con Régimen de Internado. Tablas Estatales 1999. 9.1 Actividad Obstétrica en los hospitales por dependencia. Año 1999 [website]. Madrid: Ministerio de Sanidad, Servicios Sociales e Igualdad [Ministry of Health, Social Services and Equality, Spain]; 2012 (http://www.msssi.gob.es/estadEstudios/estadisticas/estHospiInternado/inforAnual/tab, accessed 8 October 2012, in Spanish).
ESP	Spain	2000	2000	21.7	Estadística de Establecimientos Sanitarios con Régimen de Internado. Indicadores Hospitalarios Evolución 2000-2008 (Informe resumen). Madrid: Agencia de Calidad del SNS, Instituto de Información Sanitaria [Spain]; 2010 (in Spanish).
ESP	Spain	2001	2001	22.5	Estadística de Establecimientos Sanitarios con Régimen de Internado. Indicadores Hospitalarios Evolución 2000-2008 (Informe resumen). Madrid: Agencia de Calidad del SNS, Instituto de Información Sanitaria [Spain]; 2010 (in Spanish).
ESP	Spain	2002	2002	23.5	Estadística de Establecimientos Sanitarios con Régimen de Internado. Indicadores Hospitalarios Evolución 2000-2008 (Informe resumen). Madrid: Agencia de Calidad del SNS, Instituto de Información Sanitaria [Spain]; 2010 (in Spanish).
ESP	Spain	2003	2003	24.0	Estadística de Establecimientos Sanitarios con Régimen de Internado. Indicadores Hospitalarios Evolución 2000-2008 (Informe resumen). Madrid: Agencia de Calidad del SNS, Instituto de Información Sanitaria [Spain]; 2010 (in Spanish).
ESP	Spain	2004	2004	24.3	Estadística de Establecimientos Sanitarios con Régimen de Internado. Indicadores Hospitalarios Evolución 2000-2008 (Informe resumen). Madrid: Agencia de Calidad del SNS, Instituto de Información Sanitaria [Spain]; 2010 (in Spanish).
ESP	Spain	2005	2005	25.2	Estadística de Establecimientos Sanitarios con Régimen de Internado. Indicadores Hospitalarios Evolución 2000-2008 (Informe resumen). Madrid: Agencia de Calidad del SNS, Instituto de Información Sanitaria [Spain]; 2010 (in Spanish).
ESP	Spain	2006	2006	26.0	Estadística de Establecimientos Sanitarios con Régimen de Internado. Indicadores Hospitalarios Evolución 2000-2008 (Informe resumen). Madrid: Agencia de Calidad del SNS, Instituto de Información Sanitaria [Spain]; 2010 (in Spanish).

ISO Code	Country	Coverage start year	Coverage end year	Caesarean section rate (%)	References
ESP	Spain	2007	2007	25.4	Estadística de Establecimientos Sanitarios con Régimen de Internado. Indicadores Hospitalarios Evolución 2000-2008 (Informe resumen). Madrid: Agencia de Calidad del SNS, Instituto de Información Sanitaria [Spain]; 2010 (in Spanish).
ESP	Spain	2008	2008	24.9	Estadística de Establecimientos Sanitarios con Régimen de Internado. Indicadores Hospitalarios Evolución 2000-2008 (Informe resumen). Madrid: Agencia de Calidad del SNS, Instituto de Información Sanitaria [Spain]; 2010 (in Spanish).
ESP	Spain	2009	2009	25.3	Estadística de Establecimientos Sanitarios con Régimen de Internado (ESCRI) 2009. Tablas Nacionales. Madrid: Ministerio de Sanidad, Servicios Sociales e Igualdad [Ministry of Health, Social Services and Equality, Spain], Instituto de Información Sanitaria; 2010 (in Spanish).
ESP	Spain	2010	2010	25.3	Estadística de Centros Sanitarios de atención especializada. Resultados Provisionales año 2010. Madrid: Ministerio de Sanidad, Servicios Sociales e Igualdad [Ministry of Health, Social Services and Equality, Spain], Subdirección General de Información Sanitaria e Innovación; 2011 (in Spanish).
ESP	Spain	2011	2011	25.0	Estadística de Centros Sanitarios de Atención Especializada 2011. Madrid: Ministerio de Sanidad, Servicios Sociales e Igualdad [Ministry of Health, Social Services and Equality, Spain], Dirección General de Salud Pública, Calidad e Innovación, Subdirección General de Información Sanitaria e Innovación; 2013 (in Spanish).
ESP	Spain	2012	2012	25.2	European Health for All Database (HFA-DB) [online database]. World Health Organization (WHO) Regional Office for Europe; 2016 (http://data.euro.who.int/hfadb, accessed 8 August 2016).
ESP	Spain	2013	2013	27.3	Births. Year 2013. Births by type of delivery and order of birth [online database]. Madrid: Instituto Nacional Estadística [National Statistics Institute, Spain]; 2015 (http://www.ine.es/jaxi/tabla.do?type=pcaxis&path=/t20/e301/nacim/a2013/l1/&file=01018.px, accessed 15 January 2015).
ESP	Spain	2014	2014	27.3	Nacimientos. Año 2014. Nacimientos por tipo de parto y orden del nacimiento [Births. Year 2013. Births by type of delivery and order of birth] [online database]. Madrid: Instituto Nacional Estadística [National Statistics Institute, Spain]; 2016 (http://www.ine.es/jaxi/Datos.htm?type=pcaxis&path=/t20/e301/nacim/a2014/l1/&file=01018.px, accessed 3 August 2016, in Spanish).

ISO Code	Country	Coverage start year	Coverage end year	Caesarean section rate (%)	References
ESP	Spain	2015	2015	26.7	Nacimientos. Año 2015. Nacimientos por tipo de parto y orden del nacimiento [Births. Year 2015. Births by type of delivery and order of birth] [online database]. Madrid: Instituto Nacional Estadística [National Statistics Institute, Spain]; 2018 (http://www.ine.es/jaxi/Tabla.htm?path=/t20/e301/nacim/a2015/&file=01011.px, accessed 11 February 2019, in Spanish).
LKA	Sri Lanka	2003	2003	20.0	Annual Health Statistics Sri Lanka 2003. Colombo: Medical Statistics Unit [Sri Lanka]; 2004.
LKA	Sri Lanka	2005	2005	22.1	Annual Health Statistics Sri Lanka 2005. Colombo: Medical Statistics Unit [Sri Lanka]; 2006.
LKA	Sri Lanka	2006	2006	23.6	Annual Health Statistics Sri Lanka 2006. Colombo: Medical Statistics Unit [Sri Lanka]; 2007.
LKA	Sri Lanka	2001	2007	23.8	Department of Census and Statistics (DCS), Ministry of Healthcare and Nutrition (MOH). Sri Lanka Demographic and Health Survey 2006-07. Colombo, Sri Lanka: DCS, MOH; 2009.
LKA	Sri Lanka	2007	2007	24.5	Annual Health Statistics Sri Lanka 2007. Colombo: Ministry of Healthcare and Nutrition, Department of Health Services, Medical Statistics Unit [Sri Lanka]; 2008.
LKA	Sri Lanka	2008	2008	25.7	Annual Health Bulletin 2008 Sri Lanka. Colombo: Ministry of Health, Medical Statistics Unit [Sri Lanka]; 2009.
LKA	Sri Lanka	2012	2012	30.5	Annual Health Bulletin 2012 Sri Lanka. Colombo: Ministry of Health, Medical Statistics Unit [Sri Lanka]; 2013.

ISO Code	Country	Coverage start year	Coverage end year	Caesarean section rate (%)	References
LKA	Sri Lanka	2013	2013	31.3	Annual Health Bulletin 2013 Sri Lanka. Colombo: Ministry of Health, Medical Statistics Unit [Sri Lanka]; 2014.
LKA	Sri Lanka	2014	2014	32.2	Annual Health Bulletin 2014 Sri Lanka. Colombo: Ministry of Health, Nutrition and Indigenous Medicine, Medical Statistics Unit [Sri Lanka]; 2016.
LKA	Sri Lanka	2015	2015	33.2	Annual Health Bulletin 2015 Sri Lanka. Colombo: Ministry of Health, Nutrition and Indigenous Medicine, Medical Statistics Unit [Sri Lanka]; 2017.
LBY	State of Libya	1990	1995	7.2	Jurdi R, Khawaja M. Caesarean section rates in the Arab region: a cross-national study. Health Policy Plan. 2004;19(2):101-10.
SDN	Sudan	2008	2010	6.6	Sudan Household and Health Survey (SHHS2) - Round 2, 2010, National Report. Khartoum: Federal Ministry of Health, Central Bureau of Statistics; 2012.
SDN	Sudan	2012	2014	9.1	Central Bureau of Statistics (CBS), United Nations Children's Fund (UNICEF) Sudan. Multiple Indicator Cluster Survey 2014 of Sudan, Final Report. Khartoum, Sudan: UNICEF, Central Bureau of Statistics (CBS); 2016.
SUR	Suriname	2008	2010	19.0	Ministry of Social Affairs and Housing, General Bureau of Statistics, United Nations Children's Fund (UNICEF). Suriname Multiple Indicator Cluster Survey 2010, Final Report. Paramaribo: Ministry of Social Affairs and Housing, General Bureau of Statistics, UNICEF; 2012.
SUR	Suriname	2016	2018	16.1	Suriname Multiple Indicator Cluster Survey 2018, Survey Findings Report. Paramaribo: Ministry of Social Affairs and Public Housing, United Nations Children's Fund (UNICEF); 2019.

ISO Code	Country	Coverage start year	Coverage end year	Caesarean section rate (%)	References
SWZ	Eswatini	2001	2007	7.9	Central Statistical Office (CSO) [Swaziland], Macro International Inc. Swaziland Demographic and Health Survey 2006-07. Mbabane, Swaziland: Central Statistical Office, Macro International Inc.; 2008.
SWZ	Eswatini	2008	2010	12.3	Central Statistical Office, United Nations Children's Fund (UNICEF). Swaziland Multiple Indicator Cluster Survey 2010. Final Report. Mbabane, Swaziland: Central Statistical Office, UNICEF; 2011.
SWZ	Eswatini	2012	2014	11.6	Central Statistical Office, United Nations Children's Fund (UNICEF). Swaziland Multiple Indicator Cluster Survey 2014. Final Report. Mbabane, Swaziland: Central Statistical Office, UNICEF; 2016.
SWE	Sweden	1990	1990	10.6	Graviditeter, förlossningar och nyfödda barn. Medicinska födelseregistret 1973-2010. Assisterad befruktning, 1991-2009. [Pregnancies, Deliveries and Newborn Infants. The Swedish Medical Birth Register 1973–2010. Assisted Reproduction, treatment 1991–2009]. Stockholm: Socialstyrelsen [The National Board of Health and Welfare, Sweden]; 2012 (in Swedish).
SWE	Sweden	1991	1991	10.9	Graviditeter, förlossningar och nyfödda barn. Medicinska födelseregistret 1973-2010. Assisterad befruktning, 1991-2009. [Pregnancies, Deliveries and Newborn Infants. The Swedish Medical Birth Register 1973–2010. Assisted Reproduction, treatment 1991–2009]. Stockholm: Socialstyrelsen [The National Board of Health and Welfare, Sweden]; 2012 (in Swedish).
SWE	Sweden	1992	1992	10.8	Graviditeter, förlossningar och nyfödda barn. Medicinska födelseregistret 1973-2010. Assisterad befruktning, 1991-2009. [Pregnancies, Deliveries and Newborn Infants. The Swedish Medical Birth Register 1973–2010. Assisted Reproduction, treatment 1991–2009]. Stockholm: Socialstyrelsen [The National Board of Health and Welfare, Sweden]; 2012 (in Swedish).
SWE	Sweden	1993	1993	11.2	Graviditeter, förlossningar och nyfödda barn. Medicinska födelseregistret 1973-2010. Assisterad befruktning, 1991-2009. [Pregnancies, Deliveries and Newborn Infants. The Swedish Medical Birth Register 1973–2010. Assisted Reproduction, treatment 1991–2009]. Stockholm: Socialstyrelsen [The National Board of Health and Welfare, Sweden]; 2012 (in Swedish).
SWE	Sweden	1994	1994	11.4	Graviditeter, förlossningar och nyfödda barn. Medicinska födelseregistret 1973-2010. Assisterad befruktning, 1991-2009. [Pregnancies, Deliveries and Newborn Infants. The Swedish Medical Birth Register 1973–2010. Assisted Reproduction, treatment 1991–2009]. Stockholm: Socialstyrelsen [The National Board of Health and Welfare, Sweden]; 2012 (in Swedish).

ISO Code	Country	Coverage start year	Coverage end year	Caesarean section rate (%)	References
SWE	Sweden	1995	1995	11.7	Graviditeter, förlossningar och nyfödda barn. Medicinska födelseregistret 1973-2010. Assisterad befruktning, 1991-2009. [Pregnancies, Deliveries and Newborn Infants. The Swedish Medical Birth Register 1973–2010. Assisted Reproduction, treatment 1991–2009]. Stockholm: Socialstyrelsen [The National Board of Health and Welfare, Sweden]; 2012 (in Swedish).
SWE	Sweden	1996	1996	12.1	Graviditeter, förlossningar och nyfödda barn. Medicinska födelseregistret 1973-2010. Assisterad befruktning, 1991-2009. [Pregnancies, Deliveries and Newborn Infants. The Swedish Medical Birth Register 1973–2010. Assisted Reproduction, treatment 1991–2009]. Stockholm: Socialstyrelsen [The National Board of Health and Welfare, Sweden]; 2012 (in Swedish).
SWE	Sweden	1997	1997	12.9	Graviditeter, förlossningar och nyfödda barn. Medicinska födelseregistret 1973-2010. Assisterad befruktning, 1991-2009. [Pregnancies, Deliveries and Newborn Infants. The Swedish Medical Birth Register 1973–2010. Assisted Reproduction, treatment 1991–2009]. Stockholm: Socialstyrelsen [The National Board of Health and Welfare, Sweden]; 2012 (in Swedish).
SWE	Sweden	1998	1998	13.8	Graviditeter, förlossningar och nyfödda barn. Medicinska födelseregistret 1973-2010. Assisterad befruktning, 1991-2009. [Pregnancies, Deliveries and Newborn Infants. The Swedish Medical Birth Register 1973–2010. Assisted Reproduction, treatment 1991–2009]. Stockholm: Socialstyrelsen [The National Board of Health and Welfare, Sweden]; 2012 (in Swedish).
SWE	Sweden	1999	1999	14.2	Graviditeter, förlossningar och nyfödda barn. Medicinska födelseregistret 1973-2010. Assisterad befruktning, 1991-2009. [Pregnancies, Deliveries and Newborn Infants. The Swedish Medical Birth Register 1973–2010. Assisted Reproduction, treatment 1991–2009]. Stockholm: Socialstyrelsen [The National Board of Health and Welfare, Sweden]; 2012 (in Swedish).
SWE	Sweden	2000	2000	14.8	Graviditeter, förlossningar och nyfödda barn. Medicinska födelseregistret 1973-2010. Assisterad befruktning, 1991-2009. [Pregnancies, Deliveries and Newborn Infants. The Swedish Medical Birth Register 1973–2010. Assisted Reproduction, treatment 1991–2009]. Stockholm: Socialstyrelsen [The National Board of Health and Welfare, Sweden]; 2012 (in Swedish).
SWE	Sweden	2001	2001	16.0	Graviditeter, förlossningar och nyfödda barn. Medicinska födelseregistret 1973-2010. Assisterad befruktning, 1991-2009. [Pregnancies, Deliveries and Newborn Infants. The Swedish Medical Birth Register 1973–2010. Assisted Reproduction, treatment 1991–2009]. Stockholm: Socialstyrelsen [The National Board of Health and Welfare, Sweden]; 2012 (in Swedish).
SWE	Sweden	2002	2002	16.1	Graviditeter, förlossningar och nyfödda barn. Medicinska födelseregistret 1973-2010. Assisterad befruktning, 1991-2009. [Pregnancies, Deliveries and Newborn Infants. The Swedish Medical Birth Register 1973–2010. Assisted Reproduction, treatment 1991–2009]. Stockholm: Socialstyrelsen [The National Board of Health and Welfare, Sweden]; 2012 (in Swedish).

ISO Code	Country	Coverage start year	Coverage end year	Caesarean section rate (%)	References
SWE	Sweden	2003	2003	16.4	Graviditeter, förlossningar och nyfödda barn. Medicinska födelseregistret 1973-2010. Assisterad befruktning, 1991-2009. [Pregnancies, Deliveries and Newborn Infants. The Swedish Medical Birth Register 1973–2010. Assisted Reproduction, treatment 1991–2009]. Stockholm: Socialstyrelsen [The National Board of Health and Welfare, Sweden]; 2012 (in Swedish).
SWE	Sweden	2004	2004	16.8	Graviditeter, förlossningar och nyfödda barn. Medicinska födelseregistret 1973-2010. Assisterad befruktning, 1991-2009. [Pregnancies, Deliveries and Newborn Infants. The Swedish Medical Birth Register 1973–2010. Assisted Reproduction, treatment 1991–2009]. Stockholm: Socialstyrelsen [The National Board of Health and Welfare, Sweden]; 2012 (in Swedish).
SWE	Sweden	2005	2005	17.2	Graviditeter, förlossningar och nyfödda barn. Medicinska födelseregistret 1973-2010. Assisterad befruktning, 1991-2009. [Pregnancies, Deliveries and Newborn Infants. The Swedish Medical Birth Register 1973–2010. Assisted Reproduction, treatment 1991–2009]. Stockholm: Socialstyrelsen [The National Board of Health and Welfare, Sweden]; 2012 (in Swedish).
SWE	Sweden	2006	2006	17.7	Graviditeter, förlossningar och nyfödda barn. Medicinska födelseregistret 1973-2010. Assisterad befruktning, 1991-2009. [Pregnancies, Deliveries and Newborn Infants. The Swedish Medical Birth Register 1973–2010. Assisted Reproduction, treatment 1991–2009]. Stockholm: Socialstyrelsen [The National Board of Health and Welfare, Sweden]; 2012 (in Swedish).
SWE	Sweden	2007	2007	17.6	Graviditeter, förlossningar och nyfödda barn. Medicinska födelseregistret 1973-2010. Assisterad befruktning, 1991-2009. [Pregnancies, Deliveries and Newborn Infants. The Swedish Medical Birth Register 1973–2010. Assisted Reproduction, treatment 1991–2009]. Stockholm: Socialstyrelsen [The National Board of Health and Welfare, Sweden]; 2012 (in Swedish).
SWE	Sweden	2008	2008	17.2	Graviditeter, förlossningar och nyfödda barn. Medicinska födelseregistret 1973-2010. Assisterad befruktning, 1991-2009. [Pregnancies, Deliveries and Newborn Infants. The Swedish Medical Birth Register 1973–2010. Assisted Reproduction, treatment 1991–2009]. Stockholm: Socialstyrelsen [The National Board of Health and Welfare, Sweden]; 2012 (in Swedish).
SWE	Sweden	2009	2009	17.5	Graviditeter, förlossningar och nyfödda barn. Medicinska födelseregistret 1973-2010. Assisterad befruktning, 1991-2009. [Pregnancies, Deliveries and Newborn Infants. The Swedish Medical Birth Register 1973–2010. Assisted Reproduction, treatment 1991–2009]. Stockholm: Socialstyrelsen [The National Board of Health and Welfare, Sweden]; 2012 (in Swedish).
SWE	Sweden	2010	2010	16.9	Graviditeter, förlossningar och nyfödda barn. Medicinska födelseregistret 1973-2010. Assisterad befruktning, 1991-2009. [Pregnancies, Deliveries and Newborn Infants. The Swedish Medical Birth Register 1973–2010. Assisted Reproduction, treatment 1991–2009]. Stockholm: Socialstyrelsen [The National Board of Health and Welfare, Sweden]; 2012 (in Swedish).

ISO Code	Country	Coverage start year	Coverage end year	Caesarean section rate (%)	References
SWE	Sweden	2011	2011	17.0	Graviditeter, förlossningar och nyfödda barn. Medicinska födelseregistret 1973-2011. Assisterad befruktning, 1991-2010. [Pregnancies, Deliveries and Newborn Infants. The Swedish Medical Birth Register 1973–2011. Assisted Reproduction, treatment 1991–2010]. Stockholm: Socialstyrelsen [The National Board of Health and Welfare, Sweden]; 2013 (in Swedish).
SWE	Sweden	2012	2012	16.3	European Health for All Database (HFA-DB) [online database]. World Health Organization (WHO) Regional Office for Europe; 2016 (http://data.euro.who.int/hfadb, accessed 8 August 2016).
SWE	Sweden	2013	2013	17.3	Graviditeter, förlossningar och nyfödda barn. Medicinska födelseregistret 1973-2013. Assisterad befruktning, 1991-2012. [Pregnancies, Deliveries and Newborn Infants. The Swedish Medical Birth Register 1973–2013. Assisted Reproduction, treatment 1991–2012]. Stockholm: Socialstyrelsen [The National Board of Health and Welfare, Sweden]; 2014 (in Swedish).
SWE	Sweden	2014	2014	17.7	Graviditeter, förlossningar och nyfödda barn. Medicinska födelseregistret 1973-2014. Assisterad befruktning, 1991-2013. [Pregnancies, Deliveries and Newborn Infants. The Swedish Medical Birth Register 1973–2014. Assisted Reproduction, treatment 1991–2013]. Stockholm: Socialstyrelsen [The National Board of Health and Welfare, Sweden]; 2015 (in Swedish).
SWE	Sweden	2015	2015	17.4	Statistik om graviditeter, förlossningar och nyfödda barn 2015 [Statistics on Pregnancies, Deliveries and Newborn Infants 2015]. Stockholm: Socialstyrelsen [The National Board of Health and Welfare, Sweden]; 2017 (in Swedish).
SWE	Sweden	2016	2016	17.6	Statistik om graviditeter, förlossningar och nyfödda barn 2016 [Statistics on Pregnancies, Deliveries and Newborn Infants 2016]. Stockholm: Socialstyrelsen [The National Board of Health and Welfare, Sweden]; 2018 (in Swedish).
SWE	Sweden	2017	2017	17.3	Statistik om graviditeter, förlossningar och nyfödda barn 2017 [Statistics on Pregnancies, Deliveries and Newborn Infants 2017]. Stockholm: Socialstyrelsen [The National Board of Health and Welfare, Sweden]; 2019 (in Swedish).
SWE	Sweden	2018	2018	17.3	Statistik om graviditeter, förlossningar och nyfödda barn 2018 [Statistics on Pregnancies, Deliveries and Newborn Infants 2018]. Stockholm: Socialstyrelsen [The National Board of Health and Welfare, Sweden]; 2020 (in Swedish).

ISO Code	Country	Coverage start year	Coverage end year	Caesarean section rate (%)	References
CHE	Switzerland	1999	1999	16.8	European Health for All Database (HFA-DB) [online database]. World Health Organization (WHO) Regional Office for Europe; 2012 (http://data.euro.who.int/hfadb, accessed 20 August 2012).
CHE	Switzerland	2000	2000	25.0	Gebären in Schweizer Spitälern. Spitalaufenthalte während Schwangerschaft und Entbindung. 1/2007. Neuchâtel: Bundesamt für Statistik (BFS) [Federal Statistical Office, Switzerland]; 2007 (in German).
CHE	Switzerland	2001	2001	26.5	Gebären in Schweizer Spitälern. Spitalaufenthalte während Schwangerschaft und Entbindung. 1/2007. Neuchâtel: Bundesamt für Statistik (BFS) [Federal Statistical Office, Switzerland]; 2007 (in German).
CHE	Switzerland	2002	2002	27.6	Gebären in Schweizer Spitälern. Spitalaufenthalte während Schwangerschaft und Entbindung. 1/2007. Neuchâtel: Bundesamt für Statistik (BFS) [Federal Statistical Office, Switzerland]; 2007 (in German).
CHE	Switzerland	2003	2003	29.4	Gebären in Schweizer Spitälern. Spitalaufenthalte während Schwangerschaft und Entbindung. 1/2007. Neuchâtel: Bundesamt für Statistik (BFS) [Federal Statistical Office, Switzerland]; 2007 (in German).
CHE	Switzerland	2004	2004	29.2	Gebären in Schweizer Spitälern. Spitalaufenthalte während Schwangerschaft und Entbindung. 1/2007. Neuchâtel: Bundesamt für Statistik (BFS) [Federal Statistical Office, Switzerland]; 2007 (in German).
CHE	Switzerland	2005	2005	29.0	European Health for All Database (HFA-DB) [online database]. World Health Organization (WHO) Regional Office for Europe; 2012 (http://data.euro.who.int/hfadb, accessed 20 August 2012).
CHE	Switzerland	2006	2006	30.7	European Health for All Database (HFA-DB) [online database]. World Health Organization (WHO) Regional Office for Europe; 2012 (http://data.euro.who.int/hfadb, accessed 20 August 2012).

ISO Code	Country	Coverage start year	Coverage end year	Caesarean section rate (%)	References
CHE	Switzerland	2007	2007	32.2	Medizinische Statistik der Krankenhäuser. Anzahl Kaiserschnitte an Entbindungen, Erhebungsjahr 2007 [online database]. Neuchâtel: Bundesamt für Statistik (BFS) [Federal Statistical Office, Switzerland]; 2008 (http://bfs.admin.ch/bfs/portal/de/index/themen/14/04/01/data/01/05.html, accessed 7 November 2013, in German).
CHE	Switzerland	2008	2008	33.0	Medizinische Statistik der Krankenhäuser. Anzahl Kaiserschnitte an Entbindungen, Erhebungsjahr 2008 [online database]. Neuchâtel: Bundesamt für Statistik (BFS) [Federal Statistical Office, Switzerland]; 2009 (http://bfs.admin.ch/bfs/portal/de/index/themen/14/04/01/data/01/05.html, accessed 7 November 2013, in German).
CHE	Switzerland	2009	2009	32.8	Medizinische Statistik der Krankenhäuser. Anzahl Kaiserschnitte an Entbindungen, Erhebungsjahr 2009 [online database]. Neuchâtel: Bundesamt für Statistik (BFS) [Federal Statistical Office, Switzerland]; 2011 (http://bfs.admin.ch/bfs/portal/de/index/themen/14/04/01/data/01/05.html, accessed 7 November 2013, in German).
CHE	Switzerland	2010	2010	32.8	Medizinische Statistik der Krankenhäuser. Anzahl Kaiserschnitte an Entbindungen, Erhebungsjahr 2010 [online database]. Neuchâtel: Bundesamt für Statistik (BFS) [Federal Statistical Office, Switzerland]; 2011 (http://bfs.admin.ch/bfs/portal/de/index/themen/14/04/01/data/01/05.html, accessed 7 November 2013, in German).
CHE	Switzerland	2011	2011	33.3	Medizinische Statistik der Krankenhäuser. Anzahl Kaiserschnitte an Entbindungen, Erhebungsjahr 2011 [online database]. Neuchâtel: Bundesamt für Statistik (BFS) [Federal Statistical Office, Switzerland]; 2013 (http://bfs.admin.ch/bfs/portal/de, accessed 19 December 2017, in German).
CHE	Switzerland	2012	2012	33.4	Medizinische Statistik der Krankenhäuser. Anzahl Kaiserschnitte an Entbindungen, Erhebungsjahr 2012 [online database]. Neuchâtel: Bundesamt für Statistik (BFS) [Federal Statistical Office, Switzerland]; 2014 (http://bfs.admin.ch/bfs/portal/de, accessed 19 December 2017, in German).
CHE	Switzerland	2013	2013	33.3	Medizinische Statistik der Krankenhäuser. Anzahl Kaiserschnitte an Entbindungen, Erhebungsjahr 2013 [online database]. Neuchâtel: Bundesamt für Statistik (BFS) [Federal Statistical Office, Switzerland]; 2015 (http://bfs.admin.ch/bfs/portal/de, accessed 19 December 2017, in German).
CHE	Switzerland	2014	2014	33.7	Medizinische Statistik der Krankenhäuser. Anzahl Kaiserschnitte an Entbindungen, Erhebungsjahr 2014 [online database]. Neuchâtel: Bundesamt für Statistik (BFS) [Federal Statistical Office, Switzerland]; 2015 (http://bfs.admin.ch/bfs/portal/de, accessed 19 December 2017, in German).

ISO Code	Country	Coverage start year	Coverage end year	Caesarean section rate (%)	References
CHE	Switzerland	2015	2015	33.3	Medizinische Statistik der Krankenhäuser. Anzahl Kaiserschnitte an Entbindungen, Erhebungsjahr 2015 [online database]. Neuchâtel: Bundesamt für Statistik (BFS) [Federal Statistical Office, Switzerland]; 2017 (http://bfs.admin.ch/bfs/portal/de, accessed 19 December 2017, in German).
CHE	Switzerland	2016	2016	33.2	Medizinische Statistik der Krankenhäuser. Anzahl Kaiserschnitte an Entbindungen, Erhebungsjahr 2016 [online database]. Neuchâtel: Bundesamt für Statistik (BFS) [Federal Statistical Office, Switzerland]; 2018 (https://www.bfs.admin.ch/bfs/de/home/statistiken/kataloge-datenbanken/tabellen.assetdetail.6406960.html, accessed 15 February 2019, in German).
CHE	Switzerland	2017	2017	32.3	Medizinische Statistik der Krankenhäuser. Anzahl Kaiserschnitte an Entbindungen, Erhebungsjahr 2017 [online database]. Neuchâtel: Bundesamt für Statistik (BFS) [Federal Statistical Office, Switzerland]; 2018 (https://www.bfs.admin.ch/bfs/de/home/statistiken/kataloge-datenbanken/tabellen.assetdetail.6406960.html, accessed 15 February 2019, in German).
CHE	Switzerland	2018	2018	32.1	Statistique médicale des hôpitaux. Nombre et taux de césariennes en fonction du nombre d'accouchements, en 2018 [online database]. Neuchâtel: Office fédéral de la statistique OFS [Federal Statistical Office, Switzerland]; 2019 (https://www.bfs.admin.ch/bfs/en/home/statistics/catalogues-databases/tables.assetdetail.10787004.html, accessed 6 March 2020, in French).
SYR	Syrian Arab Republic	1996	2001	15.0	The Family Health Survey in the Syrian Arab Republic [2001]. Syrian Arab Republic: Central Bureau of Statistics [Syrian Arab Republic], League of Arab States PAPFAM; 2002.
SYR	Syrian Arab Republic	2004	2009	26.0	Syrian Households [2009]. Syrian Arab Republic: Central Bureau of Statistics [Syrian Arab Republic], League of Arab States PAPFAM, United Nations Children's Fund (UNICEF), United Nations Population Fund (UNFPA), World Health Organization (WHO); 2010.
TJK	Tajikistan	1990	1990	1.9	European Health for All Database (HFA-DB) [online database]. World Health Organization (WHO) Regional Office for Europe; 2012 (http://data.euro.who.int/hfadb, accessed 20 August 2012).
TJK	Tajikistan	1991	1991	2.1	European Health for All Database (HFA-DB) [online database]. World Health Organization (WHO) Regional Office for Europe; 2012 (http://data.euro.who.int/hfadb, accessed 20 August 2012).

ISO Code	Country	Coverage start year	Coverage end year	Caesarean section rate (%)	References
TJK	Tajikistan	1992	1992	2.1	European Health for All Database (HFA-DB) [online database]. World Health Organization (WHO) Regional Office for Europe; 2012 (http://data.euro.who.int/hfadb, accessed 20 August 2012).
TJK	Tajikistan	1993	1993	1.6	European Health for All Database (HFA-DB) [online database]. World Health Organization (WHO) Regional Office for Europe; 2012 (http://data.euro.who.int/hfadb, accessed 20 August 2012).
TJK	Tajikistan	1994	1994	1.3	European Health for All Database (HFA-DB) [online database]. World Health Organization (WHO) Regional Office for Europe; 2012 (http://data.euro.who.int/hfadb, accessed 20 August 2012).
TJK	Tajikistan	1995	1995	1.2	European Health for All Database (HFA-DB) [online database]. World Health Organization (WHO) Regional Office for Europe; 2012 (http://data.euro.who.int/hfadb, accessed 20 August 2012).
TJK	Tajikistan	1996	1996	1.7	European Health for All Database (HFA-DB) [online database]. World Health Organization (WHO) Regional Office for Europe; 2012 (http://data.euro.who.int/hfadb, accessed 20 August 2012).
TJK	Tajikistan	1997	1997	1.3	European Health for All Database (HFA-DB) [online database]. World Health Organization (WHO) Regional Office for Europe; 2012 (http://data.euro.who.int/hfadb, accessed 20 August 2012).
TJK	Tajikistan	1998	1998	1.3	European Health for All Database (HFA-DB) [online database]. World Health Organization (WHO) Regional Office for Europe; 2012 (http://data.euro.who.int/hfadb, accessed 20 August 2012).
TJK	Tajikistan	1999	1999	1.2	European Health for All Database (HFA-DB) [online database]. World Health Organization (WHO) Regional Office for Europe; 2012 (http://data.euro.who.int/hfadb, accessed 20 August 2012).

ISO Code	Country	Coverage start year	Coverage end year	Caesarean section rate (%)	References
TJK	Tajikistan	2000	2000	1.5	European Health for All Database (HFA-DB) [online database]. World Health Organization (WHO) Regional Office for Europe; 2012 (http://data.euro.who.int/hfadb, accessed 20 August 2012).
TJK	Tajikistan	2001	2001	1.4	European Health for All Database (HFA-DB) [online database]. World Health Organization (WHO) Regional Office for Europe; 2012 (http://data.euro.who.int/hfadb, accessed 20 August 2012).
TJK	Tajikistan	2002	2002	1.6	European Health for All Database (HFA-DB) [online database]. World Health Organization (WHO) Regional Office for Europe; 2012 (http://data.euro.who.int/hfadb, accessed 20 August 2012).
TJK	Tajikistan	2003	2003	1.6	European Health for All Database (HFA-DB) [online database]. World Health Organization (WHO) Regional Office for Europe; 2012 (http://data.euro.who.int/hfadb, accessed 20 August 2012).
TJK	Tajikistan	2004	2004	1.9	European Health for All Database (HFA-DB) [online database]. World Health Organization (WHO) Regional Office for Europe; 2012 (http://data.euro.who.int/hfadb, accessed 20 August 2012).
TJK	Tajikistan	2005	2005	2.0	European Health for All Database (HFA-DB) [online database]. World Health Organization (WHO) Regional Office for Europe; 2012 (http://data.euro.who.int/hfadb, accessed 20 August 2012).
TJK	Tajikistan	2006	2006	2.1	European Health for All Database (HFA-DB) [online database]. World Health Organization (WHO) Regional Office for Europe; 2012 (http://data.euro.who.int/hfadb, accessed 20 August 2012).
TJK	Tajikistan	2007	2007	2.4	European Health for All Database (HFA-DB) [online database]. World Health Organization (WHO) Regional Office for Europe; 2012 (http://data.euro.who.int/hfadb, accessed 20 August 2012).

ISO Code	Country	Coverage start year	Coverage end year	Caesarean section rate (%)	References
TJK	Tajikistan	2008	2008	2.8	European Health for All Database (HFA-DB) [online database]. World Health Organization (WHO) Regional Office for Europe; 2012 (http://data.euro.who.int/hfadb, accessed 20 August 2012).
TJK	Tajikistan	2011	2011	4.1	European Health for All Database (HFA-DB) [online database]. World Health Organization (WHO) Regional Office for Europe; 2016 (http://data.euro.who.int/hfadb, accessed 8 August 2016).
TJK	Tajikistan	2012	2012	4.6	European Health for All Database (HFA-DB) [online database]. World Health Organization (WHO) Regional Office for Europe; 2016 (http://data.euro.who.int/hfadb, accessed 8 August 2016).
TJK	Tajikistan	2007	2012	4.0	Statistical Agency under the President of the Republic of Tajikistan (SA), Ministry of Health [Tajikistan], ICF International. Tajikistan Demographic and Health Survey 2012. Dushanbe, Tajikistan, Calverton, Maryland, USA: SA, MOH, ICF International; 2013.
TJK	Tajikistan	2013	2013	6.0	European Health for All Database (HFA-DB) [online database]. World Health Organization (WHO) Regional Office for Europe; 2016 (http://data.euro.who.int/hfadb, accessed 8 August 2016).
TJK	Tajikistan	2012	2017	5.3	Tajikistan Demographic and Health Survey 2017. Rockville, Maryland, USA: Statistical Agency under the President of the Republic of Tajikistan (SA), Ministry of Health and Social Protection of Population of the Republic of Tajikistan (MOHSP), ICF; 2018.
THA	Thailand	1990	1990	15.2	Hanvoravongchai P, Letiendumrong J, Teerawattananon Y, Tangcharoensathien V. Implications of Private Practice in Public Hospitals on the Cesarean Section Rate in Thailand. Human resources for health development journal: HRDJ. 2000;4:2-12.
THA	Thailand	1991	1991	16.0	Hanvoravongchai P, Letiendumrong J, Teerawattananon Y, Tangcharoensathien V. Implications of Private Practice in Public Hospitals on the Cesarean Section Rate in Thailand. Human resources for health development journal: HRDJ. 2000;4:2-12.

ISO Code	Country	Coverage start year	Coverage end year	Caesarean section rate (%)	References
THA	Thailand	1992	1992	17.0	Hanvoravongchai P, Letiendumrong J, Teerawattananon Y, Tangcharoensathien V. Implications of Private Practice in Public Hospitals on the Cesarean Section Rate in Thailand. Human resources for health development journal: HRDJ. 2000;4:2-12.
THA	Thailand	1993	1993	18.4	Hanvoravongchai P, Letiendumrong J, Teerawattananon Y, Tangcharoensathien V. Implications of Private Practice in Public Hospitals on the Cesarean Section Rate in Thailand. Human resources for health development journal: HRDJ. 2000;4:2-12.
THA	Thailand	1994	1994	20.0	Hanvoravongchai P, Letiendumrong J, Teerawattananon Y, Tangcharoensathien V. Implications of Private Practice in Public Hospitals on the Cesarean Section Rate in Thailand. Human resources for health development journal: HRDJ. 2000;4:2-12.
THA	Thailand	1995	1995	21.1	Hanvoravongchai P, Letiendumrong J, Teerawattananon Y, Tangcharoensathien V. Implications of Private Practice in Public Hospitals on the Cesarean Section Rate in Thailand. Human resources for health development journal: HRDJ. 2000;4:2-12.
THA	Thailand	1996	1996	22.4	Hanvoravongchai P, Letiendumrong J, Teerawattananon Y, Tangcharoensathien V. Implications of Private Practice in Public Hospitals on the Cesarean Section Rate in Thailand. Human resources for health development journal: HRDJ. 2000;4:2-12.
THA	Thailand	2010	2012	32.0	National Statistical Office (NSO), United Nations Children's Fund (UNICEF). Thailand Multiple Indicator Cluster Survey 2012. Final Report. Bangkok: NSO, UNICEF; 2013.
THA	Thailand	2013	2016	32.7	National Statistical Office (NSO), United Nations Children's Fund (UNICEF). Thailand Multiple Indicator Cluster Survey 2015-16. Final Report. Bangkok: NSO, UNICEF; 2016.
MKD	The former Yugoslav Republic of Macedonia	1995	1995	1.4	European Health for All Database (HFA-DB) [online database]. World Health Organization (WHO) Regional Office for Europe; 2012 (http://data.euro.who.int/hfadb, accessed 20 August 2012).

ISO Code	Country	Coverage start year	Coverage end year	Caesarean section rate (%)	References
MKD	The former Yugoslav Republic of Macedonia	1997	1997	7.8	European Health for All Database (HFA-DB) [online database]. World Health Organization (WHO) Regional Office for Europe; 2012 (http://data.euro.who.int/hfadb, accessed 20 August 2012).
MKD	The former Yugoslav Republic of Macedonia	1998	1998	8.6	European Health for All Database (HFA-DB) [online database]. World Health Organization (WHO) Regional Office for Europe; 2012 (http://data.euro.who.int/hfadb, accessed 20 August 2012).
MKD	The former Yugoslav Republic of Macedonia	1999	1999	9.8	European Health for All Database (HFA-DB) [online database]. World Health Organization (WHO) Regional Office for Europe; 2012 (http://data.euro.who.int/hfadb, accessed 20 August 2012).
MKD	The former Yugoslav Republic of Macedonia	2000	2000	9.1	European Health for All Database (HFA-DB) [online database]. World Health Organization (WHO) Regional Office for Europe; 2012 (http://data.euro.who.int/hfadb, accessed 20 August 2012).
MKD	The former Yugoslav Republic of Macedonia	2001	2001	10.2	European Health for All Database (HFA-DB) [online database]. World Health Organization (WHO) Regional Office for Europe; 2012 (http://data.euro.who.int/hfadb, accessed 20 August 2012).
MKD	The former Yugoslav Republic of Macedonia	2002	2002	10.5	European Health for All Database (HFA-DB) [online database]. World Health Organization (WHO) Regional Office for Europe; 2012 (http://data.euro.who.int/hfadb, accessed 20 August 2012).
MKD	The former Yugoslav Republic of Macedonia	2003	2003	11.4	European Health for All Database (HFA-DB) [online database]. World Health Organization (WHO) Regional Office for Europe; 2012 (http://data.euro.who.int/hfadb, accessed 20 August 2012).
MKD	The former Yugoslav Republic of Macedonia	2006	2006	16.9	European Health for All Database (HFA-DB) [online database]. World Health Organization (WHO) Regional Office for Europe; 2012 (http://data.euro.who.int/hfadb, accessed 20 August 2012).

ISO Code	Country	Coverage start year	Coverage end year	Caesarean section rate (%)	References
MKD	The former Yugoslav Republic of Macedonia	2007	2007	19.8	European Health for All Database (HFA-DB) [online database]. World Health Organization (WHO) Regional Office for Europe; 2012 (http://data.euro.who.int/hfadb, accessed 20 August 2012).
MKD	The former Yugoslav Republic of Macedonia	2008	2008	18.0	European Health for All Database (HFA-DB) [online database]. World Health Organization (WHO) Regional Office for Europe; 2012 (http://data.euro.who.int/hfadb, accessed 20 August 2012).
MKD	The former Yugoslav Republic of Macedonia	2009	2009	17.6	European Health for All Database (HFA-DB) [online database]. World Health Organization (WHO) Regional Office for Europe; 2012 (http://data.euro.who.int/hfadb, accessed 20 August 2012).
MKD	The former Yugoslav Republic of Macedonia	2009	2011	24.9	Macedonia Multiple Indicator Cluster Survey 2011 (MICS 2011). Republic of Macedonia: Ministry of Health, Ministry of Education and Science, Ministry of Labour and Social Policy of the Government of Republic of Macedonia, United Nations Children's Fund (UNICEF), United Nations Population Fund (UNFPA); 2014.
MKD	The former Yugoslav Republic of Macedonia	2011	2011	23.4	European Health for All Database (HFA-DB) [online database]. World Health Organization (WHO) Regional Office for Europe; 2016 (http://data.euro.who.int/hfadb, accessed 8 August 2016).
MKD	The former Yugoslav Republic of Macedonia	2012	2012	24.9	European Health for All Database (HFA-DB) [online database]. World Health Organization (WHO) Regional Office for Europe; 2016 (http://data.euro.who.int/hfadb, accessed 8 August 2016).
MKD	The former Yugoslav Republic of Macedonia	2013	2013	27.4	European Health for All Database (HFA-DB) [online database]. World Health Organization (WHO) Regional Office for Europe; 2016 (http://data.euro.who.int/hfadb, accessed 8 August 2016).
MKD	The former Yugoslav Republic of Macedonia	2014	2014	28.9	European Health for All Database (HFA-DB) [online database]. World Health Organization (WHO) Regional Office for Europe; 2016 (http://data.euro.who.int/hfadb, accessed 8 August 2016).

ISO Code	Country	Coverage start year	Coverage end year	Caesarean section rate (%)	References
TLS	Timor-Leste	1998	2003	0.3	Ministry of Health and National Statistics Office [Timor-Leste], University of Newcastle [Australia], The Australian National University [Australia], ACIL Australia Pty Ltd [Australia]. Timor-Leste 2003 Demographic and Health Survey. Newcastle: University of Newcastle; 2004.
TLS	Timor-Leste	2004	2010	1.7	National Statistics Directorate (NSD) [Timor-Leste], Ministry of Finance [Timor-Leste], ICF Macro. Timor-Leste Demographic and Health Survey 2009-10. Dili, Timor-Leste: NSD [Timor-Leste], ICF Macro; 2010.
TLS	Timor-Leste	2011	2016	3.5	Timor-Leste Demographic and Health Survey 2016. Dili, Timor-Leste and Rockville, Maryland, USA: General Directorate of Statistics (GDS), ICF; 2018.
TGO	Togo	1995	1998	2.0	Anipah K, Mboup G, Ouro-Gnao AM, Boukpessi B, Messan PA, Salami-Odjo R (Ministère de la Planification et du Développement Economique Direction de la Statistique, Macro International Inc). Enquête Démographique et de Santé, Togo 1998. Calverton, Maryland USA: Ministère de la Planification et du Développement Economique Direction de la Statistique, Macro International Inc.; 1999 (in French).
TGO	Togo	2008	2010	8.8	Enquête par grappes à indicateurs multiples MICS Togo, 2010, Rapport final. Lomé: Direction Générale de la Statistique et de la Comptabilité Nationale (DGSCN), Fonds des Nations Unies pour l'Enfance (UNICEF); 2012 (in French).
TGO	Togo	2008	2014	6.5	Ministère de la Planification, du Développement et de l'Aménagement du Territoire (MPDAT), Ministère de la Santé (MS), ICF International. Enquête Démographique et de Santé au Togo 2013-2014. Rockville, Maryland, USA : MPDAT, MS, ICF International; 2015 (in French).
TGO	Togo	2015	2017	8.6	Enquête par grappes à indicateurs multiples (MICS6) Togo, 2017, Rapport final. Lomé: Institut National de la Statistique et des Etudes Economiques et Démographiques (INSEED), UNICEF; 2018 (in French).
TON	Tonga	2001	2001	5.8	Report of the Minister of Health for the Year 2005. Tonga: Ministry of Health [Tonga]; 2006.

ISO Code	Country	Coverage start year	Coverage end year	Caesarean section rate (%)	References
TON	Tonga	2002	2002	8.9	Report of the Minister of Health for the Year 2005. Tonga: Ministry of Health [Tonga]; 2006.
TON	Tonga	2003	2003	8.4	Report of the Minister of Health for the Year 2005. Tonga: Ministry of Health [Tonga]; 2006.
TON	Tonga	2004	2004	9.6	Report of the Minister of Health for the Year 2005. Tonga: Ministry of Health [Tonga]; 2006.
TON	Tonga	2005	2005	11.1	Report of the Minister of Health for the Year 2005. Tonga: Ministry of Health [Tonga]; 2006.
TON	Tonga	2007	2012	17.4	Tonga Department of Statistics and Tonga Ministry of Health, Secretariat of the Pacific Community (SPC), United Nations Population Fund (UNFPA). Tonga Demographic and Health Survey 2012. Final Report. Noumea: SPC; 2013.
TTO	Trinidad and Tobago	2007	2007	18.4	Mungrue K, Nixon C, David Y, Dookwah D, Durga S, Greene K et al. Trinidadian women's knowledge, perceptions, and preferences regarding cesarean section: How do they make choices? Int J Womens Health. 2010;2:387-91.
TUN	Tunisia	1989	1995	9.9	L'Enquête Tunisienne sur la Santé de la Mère et de l'Enfant 1994-1995 (ETSME). Rapport Principal. Tunisie; Ministère de la Santé Publique; 1996 (in French).
TUN	Tunisia	1999	2000	17.8	Enquête sur la Santé et le Bien-être de la Mère et l'Enfant 2000 (MICS 2). Tunis: Ministère de la Santé Publique [Tunisie], Fonds des Nations Unies pour l'Enfance (UNICEF); 2000 (in French).

ISO Code	Country	Coverage start year	Coverage end year	Caesarean section rate (%)	References
TUN	Tunisia	2001	2006	20.5	Enquête sur la Santé et le Bien-être de la Mère et l'Enfant 2006 (MICS 3). Tunis: Ministère de la Santé Publique [Tunisie], Fonds des Nations Unies pour l'Enfance (UNICEF); 2008 (in French).
TUN	Tunisia	2009	2012	26.7	Ministère du Développement et de la Coopération Internationale (MDCI), Institut National de la Statistique, Fonds des Nations Unies pour l'Enfance (UNICEF).<br>Suivi de la situation des enfants et des femmes en Tunisie - Enquête par grappes à indicateurs multiples 2011-2012, Rapport Final. Tunisie: MDCI, Institut National de la Statistique, UNICEF; 2013 (in French).
TUN	Tunisia	2016	2018	43.2	Institut National de la Statistique, UNICEF. Enquête par grappes à indicateurs multiples (MICS) 2018, Rapport Final. Tunisie: Le Ministère du Développement de l'Investissement et de la Coopération Internationale (MDICI); 2019 (in French).
TUR	Turkey	1993	1998	13.9	Ministry of Health [Turkey], Hacettepe University Institute of Population Studies [Turkey], Macro International. Turkish Demographic and Health Survey 1993. Ankara, Turkey: Ministry of Health [Turkey], Hacettepe University Institute of Population Studies [Turkey], Macro International; 1994.
TUR	Turkey	1998	2003	21.2	Hacettepe University Institute of Population Studies [Turkey]. Turkey Demographic and Health Survey, 2003. Ankara, Turkey: Hacettepe University Institute of Population Studies [Turkey], Ministry of Health General Directorate of Mother and Child Health and Family Planning [Turkey], State Planning Organization, European Union; 2004.
TUR	Turkey	2007	2007	37.5	European Health for All Database (HFA-DB) [online database]. World Health Organization (WHO) Regional Office for Europe; 2012 (http://data.euro.who.int/hfadb, accessed 20 August 2012).
TUR	Turkey	2003	2008	36.7	Hacettepe University Institute of Population Studies. Turkey Demographic and Health Survey, 2008. Ankara, Turkey: Hacettepe University Institute of Population Studies, Ministry of Health General Directorate of Mother and Child Health and Family Planning, T.R. Prime Ministry Undersecretary of State Planning Organization, TÜBİTAK; 2009.
TUR	Turkey	2008	2008	40.9	European Health for All Database (HFA-DB) [online database]. World Health Organization (WHO) Regional Office for Europe; 2012 (http://data.euro.who.int/hfadb, accessed 20 August 2012).

ISO Code	Country	Coverage start year	Coverage end year	Caesarean section rate (%)	References
TUR	Turkey	2009	2009	44.5	European Health for All Database (HFA-DB) [online database]. World Health Organization (WHO) Regional Office for Europe; 2012 (http://data.euro.who.int/hfadb, accessed 20 August 2012).
TUR	Turkey	2012	2012	47.7	European Health for All Database (HFA-DB) [online database]. World Health Organization (WHO) Regional Office for Europe; 2016 (http://data.euro.who.int/hfadb, accessed 8 August 2016).
TUR	Turkey	2013	2013	50.4	European Health for All Database (HFA-DB) [online database]. World Health Organization (WHO) Regional Office for Europe; 2016 (http://data.euro.who.int/hfadb, accessed 8 August 2016).
TUR	Turkey	2008	2013	48.1	Hacettepe University Institute of Population Studies. 2013 Turkey Demographic and Health Survey. Ankara, Turkey: Hacettepe University Institute of Population Studies, T.R. Ministry of Development, TÜBİTAK; 2014.
TUR	Turkey	2014	2014	50.8	European Health Information Gateway. European Health for All database (HFA-DB). Caesarean sections per 1000 live births [online database]. World Health Organization (WHO) Regional Office for Europe; 2018 (https://gateway.euro.who.int/en/indicators/hfa_596-7060-caesarean-sections-per-1000-live-births/, accessed 14 February 2019).
TKM	Turkmenistan	1991	1991	3.0	European Health for All Database (HFA-DB) [online database]. World Health Organization (WHO) Regional Office for Europe; 2012 (http://data.euro.who.int/hfadb, accessed 20 August 2012).
TKM	Turkmenistan	1992	1992	2.7	European Health for All Database (HFA-DB) [online database]. World Health Organization (WHO) Regional Office for Europe; 2012 (http://data.euro.who.int/hfadb, accessed 20 August 2012).
TKM	Turkmenistan	1993	1993	2.8	European Health for All Database (HFA-DB) [online database]. World Health Organization (WHO) Regional Office for Europe; 2012 (http://data.euro.who.int/hfadb, accessed 20 August 2012).

ISO Code	Country	Coverage start year	Coverage end year	Caesarean section rate (%)	References
TKM	Turkmenistan	1994	1994	2.5	European Health for All Database (HFA-DB) [online database]. World Health Organization (WHO) Regional Office for Europe; 2012 (http://data.euro.who.int/hfadb, accessed 20 August 2012).
TKM	Turkmenistan	1995	1995	2.5	European Health for All Database (HFA-DB) [online database]. World Health Organization (WHO) Regional Office for Europe; 2012 (http://data.euro.who.int/hfadb, accessed 20 August 2012).
TKM	Turkmenistan	1996	1996	2.6	European Health for All Database (HFA-DB) [online database]. World Health Organization (WHO) Regional Office for Europe; 2012 (http://data.euro.who.int/hfadb, accessed 20 August 2012).
TKM	Turkmenistan	1995	2000	3.1	Gurbansoltan Eje Clinical Research Center for Maternal and Child Health (GECRCMCH), Ministry of Health and Medical Industry [Turkmenistan], ORC Macro. Turkmenistan Demographic and Health Survey 2000. Calverton, Maryland, USA: GECRCMCH, ORC Macro; 2001.
TKM	Turkmenistan	2000	2000	3.2	European Health for All Database (HFA-DB) [online database]. World Health Organization (WHO) Regional Office for Europe; 2012 (http://data.euro.who.int/hfadb, accessed 20 August 2012).
TKM	Turkmenistan	2001	2001	3.1	European Health for All Database (HFA-DB) [online database]. World Health Organization (WHO) Regional Office for Europe; 2012 (http://data.euro.who.int/hfadb, accessed 20 August 2012).
TKM	Turkmenistan	2002	2002	3.4	European Health for All Database (HFA-DB) [online database]. World Health Organization (WHO) Regional Office for Europe; 2012 (http://data.euro.who.int/hfadb, accessed 20 August 2012).
TKM	Turkmenistan	2003	2003	3.0	European Health for All Database (HFA-DB) [online database]. World Health Organization (WHO) Regional Office for Europe; 2012 (http://data.euro.who.int/hfadb, accessed 20 August 2012).

ISO Code	Country	Coverage start year	Coverage end year	Caesarean section rate (%)	References
TKM	Turkmenistan	2004	2004	2.5	European Health for All Database (HFA-DB) [online database]. World Health Organization (WHO) Regional Office for Europe; 2012 (http://data.euro.who.int/hfadb, accessed 20 August 2012).
TKM	Turkmenistan	2005	2005	3.2	European Health for All Database (HFA-DB) [online database]. World Health Organization (WHO) Regional Office for Europe; 2012 (http://data.euro.who.int/hfadb, accessed 20 August 2012).
TKM	Turkmenistan	2006	2006	3.4	European Health for All Database (HFA-DB) [online database]. World Health Organization (WHO) Regional Office for Europe; 2012 (http://data.euro.who.int/hfadb, accessed 20 August 2012).
TKM	Turkmenistan	2007	2007	3.8	European Health for All Database (HFA-DB) [online database]. World Health Organization (WHO) Regional Office for Europe; 2012 (http://data.euro.who.int/hfadb, accessed 20 August 2012).
TKM	Turkmenistan	2008	2008	3.9	European Health for All Database (HFA-DB) [online database]. World Health Organization (WHO) Regional Office for Europe; 2012 (http://data.euro.who.int/hfadb, accessed 20 August 2012).
TKM	Turkmenistan	2009	2009	4.5	European Health for All Database (HFA-DB) [online database]. World Health Organization (WHO) Regional Office for Europe; 2012 (http://data.euro.who.int/hfadb, accessed 20 August 2012).
TKM	Turkmenistan	2010	2010	4.7	European Health for All Database (HFA-DB) [online database]. World Health Organization (WHO) Regional Office for Europe; 2016 (http://data.euro.who.int/hfadb, accessed 8 August 2016).
TKM	Turkmenistan	2011	2011	5.9	European Health for All Database (HFA-DB) [online database]. World Health Organization (WHO) Regional Office for Europe; 2016 (http://data.euro.who.int/hfadb, accessed 8 August 2016).

ISO Code	Country	Coverage start year	Coverage end year	Caesarean section rate (%)	References
TKM	Turkmenistan	2012	2012	6.6	European Health for All Database (HFA-DB) [online database]. World Health Organization (WHO) Regional Office for Europe; 2016 (http://data.euro.who.int/hfadb, accessed 8 August 2016).
TKM	Turkmenistan	2013	2013	7.5	European Health for All Database (HFA-DB) [online database]. World Health Organization (WHO) Regional Office for Europe; 2016 (http://data.euro.who.int/hfadb, accessed 8 August 2016).
TKM	Turkmenistan	2013	2016	6.3	The State Committee of Statistics of Turkmenistan, United Nations Children's Fund (UNICEF). 2015-2016 Turkmenistan Multiple Indicator Cluster Survey, Final Report. Ashgabat, Turkmenistan: The State Committee of Statistics of Turkmenistan, UNICEF; 2016.
TKM	Turkmenistan	2017	2017	8.4	European Health Information Gateway. European Health for All database (HFA-DB). Caesarean sections per 1000 live births [online database]. World Health Organization (WHO) Regional Office for Europe; 2019 (https://gateway.euro.who.int/en/indicators/hfa_596-7060-caesarean-sections-per-1000-live-births/, accessed 5 March 2020).
TKM	Turkmenistan	2018	2018	9.3	European Health Information Gateway. European Health for All database (HFA-DB). Caesarean sections per 1000 live births [online database]. World Health Organization (WHO) Regional Office for Europe; 2019 (https://gateway.euro.who.int/en/indicators/hfa_596-7060-caesarean-sections-per-1000-live-births/, accessed 5 March 2020).
TUV	Tuvalu	2002	2007	7.1	Central Statistics Division [Tuvalu], Secretariat of the Pacific Community (SPC), Macro International Inc., Asian Development Bank. Tuvalu Demographic and Health Survey 2007. Noumea: SPC; 2009.
UGA	Uganda	1990	1995	2.6	Statistics Department [Uganda], Macro International Inc. Uganda Demographic and Health Survey, 1995. Calverton, Maryland: Statistics Department [Uganda], Macro International Inc.; 1996.
UGA	Uganda	1995	2001	2.5	Uganda Bureau of Statistics (UBOS), ORC Macro. Uganda Demographic and Health Survey 2000-2001. Calverton, Maryland, USA: UBOS, ORC Macro; 2001.

ISO Code	Country	Coverage start year	Coverage end year	Caesarean section rate (%)	References
UGA	Uganda	2001	2006	3.1	Uganda Bureau of Statistics (UBOS), Macro International Inc. Uganda Demographic and Health Survey 2006. Calverton, Maryland, USA: UBOS, Macro International Inc.; 2007.
UGA	Uganda	2006	2011	5.3	Uganda Bureau of Statistics (UBOS), ICF International. Uganda Demographic and Health Survey 2011. Kampala, Uganda: UBOS, ICF International; 2012.
UGA	Uganda	2011	2016	6.2	Uganda Bureau of Statistics (UBOS), ICF. Uganda Demographic and Health Survey 2016. Kampala, Uganda and Rockville, Maryland, USA: UBOS, ICF; 2018.
UKR	Ukraine	1990	1990	5.7	European Health for All Database (HFA-DB) [online database]. World Health Organization (WHO) Regional Office for Europe; 2012 (http://data.euro.who.int/hfadb, accessed 20 August 2012).
UKR	Ukraine	1991	1991	6.2	European Health for All Database (HFA-DB) [online database]. World Health Organization (WHO) Regional Office for Europe; 2012 (http://data.euro.who.int/hfadb, accessed 20 August 2012).
UKR	Ukraine	1992	1992	5.1	European Health for All Database (HFA-DB) [online database]. World Health Organization (WHO) Regional Office for Europe; 2012 (http://data.euro.who.int/hfadb, accessed 20 August 2012).
UKR	Ukraine	1993	1993	7.0	European Health for All Database (HFA-DB) [online database]. World Health Organization (WHO) Regional Office for Europe; 2012 (http://data.euro.who.int/hfadb, accessed 20 August 2012).
UKR	Ukraine	1994	1994	7.2	European Health for All Database (HFA-DB) [online database]. World Health Organization (WHO) Regional Office for Europe; 2012 (http://data.euro.who.int/hfadb, accessed 20 August 2012).

ISO Code	Country	Coverage start year	Coverage end year	Caesarean section rate (%)	References
UKR	Ukraine	1995	1995	7.5	European Health for All Database (HFA-DB) [online database]. World Health Organization (WHO) Regional Office for Europe; 2012 (http://data.euro.who.int/hfadb, accessed 20 August 2012).
UKR	Ukraine	1996	1996	8.1	European Health for All Database (HFA-DB) [online database]. World Health Organization (WHO) Regional Office for Europe; 2012 (http://data.euro.who.int/hfadb, accessed 20 August 2012).
UKR	Ukraine	1997	1997	8.6	European Health for All Database (HFA-DB) [online database]. World Health Organization (WHO) Regional Office for Europe; 2012 (http://data.euro.who.int/hfadb, accessed 20 August 2012).
UKR	Ukraine	1998	1998	9.1	European Health for All Database (HFA-DB) [online database]. World Health Organization (WHO) Regional Office for Europe; 2012 (http://data.euro.who.int/hfadb, accessed 20 August 2012).
UKR	Ukraine	1999	1999	9.5	European Health for All Database (HFA-DB) [online database]. World Health Organization (WHO) Regional Office for Europe; 2012 (http://data.euro.who.int/hfadb, accessed 20 August 2012).
UKR	Ukraine	2000	2000	10.4	European Health for All Database (HFA-DB) [online database]. World Health Organization (WHO) Regional Office for Europe; 2012 (http://data.euro.who.int/hfadb, accessed 20 August 2012).
UKR	Ukraine	2001	2001	11.4	European Health for All Database (HFA-DB) [online database]. World Health Organization (WHO) Regional Office for Europe; 2012 (http://data.euro.who.int/hfadb, accessed 20 August 2012).
UKR	Ukraine	2002	2002	12.2	European Health for All Database (HFA-DB) [online database]. World Health Organization (WHO) Regional Office for Europe; 2012 (http://data.euro.who.int/hfadb, accessed 20 August 2012).

ISO Code	Country	Coverage start year	Coverage end year	Caesarean section rate (%)	References
UKR	Ukraine	2003	2003	12.8	European Health for All Database (HFA-DB) [online database]. World Health Organization (WHO) Regional Office for Europe; 2012 (http://data.euro.who.int/hfadb, accessed 20 August 2012).
UKR	Ukraine	2004	2004	13.4	European Health for All Database (HFA-DB) [online database]. World Health Organization (WHO) Regional Office for Europe; 2012 (http://data.euro.who.int/hfadb, accessed 20 August 2012).
UKR	Ukraine	2005	2005	13.7	European Health for All Database (HFA-DB) [online database]. World Health Organization (WHO) Regional Office for Europe; 2012 (http://data.euro.who.int/hfadb, accessed 20 August 2012).
UKR	Ukraine	2006	2006	14.2	European Health for All Database (HFA-DB) [online database]. World Health Organization (WHO) Regional Office for Europe; 2012 (http://data.euro.who.int/hfadb, accessed 20 August 2012).
UKR	Ukraine	2007	2007	15.2	European Health for All Database (HFA-DB) [online database]. World Health Organization (WHO) Regional Office for Europe; 2012 (http://data.euro.who.int/hfadb, accessed 20 August 2012).
UKR	Ukraine	2008	2008	15.6	European Health for All Database (HFA-DB) [online database]. World Health Organization (WHO) Regional Office for Europe; 2012 (http://data.euro.who.int/hfadb, accessed 20 August 2012).
UKR	Ukraine	2009	2009	15.9	European Health for All Database (HFA-DB) [online database]. World Health Organization (WHO) Regional Office for Europe; 2012 (http://data.euro.who.int/hfadb, accessed 20 August 2012).
UKR	Ukraine	2010	2010	15.8	European Health for All Database (HFA-DB) [online database]. World Health Organization (WHO) Regional Office for Europe; 2016 (http://data.euro.who.int/hfadb, accessed 8 August 2016).

ISO Code	Country	Coverage start year	Coverage end year	Caesarean section rate (%)	References
UKR	Ukraine	2012	2012	16.2	European Health for All Database (HFA-DB) [online database]. World Health Organization (WHO) Regional Office for Europe; 2016 (http://data.euro.who.int/hfadb, accessed 8 August 2016).
UKR	Ukraine	2010	2012	12.1	State Statistics Service, Ukrainian Center for Social Reforms, United Nations Children's Fund (UNICEF). Ukraine Multiple Indicator Cluster Survey 2012, Final Report. Kiev, Ukraine: State Statistics Committee, the Ukrainian Center for Social Reforms; 2013.
UKR	Ukraine	2013	2013	16.9	European Health for All Database (HFA-DB) [online database]. World Health Organization (WHO) Regional Office for Europe; 2016 (http://data.euro.who.int/hfadb, accessed 8 August 2016).
UKR	Ukraine	2014	2014	16.5	European Health Information Gateway. European Health for All database (HFA-DB). Caesarean sections per 1000 live births [online database]. World Health Organization (WHO) Regional Office for Europe; 2018 (https://gateway.euro.who.int/en/indicators/hfa_596-7060-caesarean-sections-per-1000-live-births/, accessed 14 February 2019).
UKR	Ukraine	2015	2015	17.9	European Health Information Gateway. European Health for All database (HFA-DB). Caesarean sections per 1000 live births [online database]. World Health Organization (WHO) Regional Office for Europe; 2018 (https://gateway.euro.who.int/en/indicators/hfa_596-7060-caesarean-sections-per-1000-live-births/, accessed 14 February 2019).
ARE	United Arab Emirates	2006	2006	18.5	Open Data: Deliveries and births by nationality, mode of delivery, condition of new born & district [2006] [website]. Abu Dhabi: Ministry of Health [UAE]; 2007 (www.http://213.42.151.126/Statistics/Statistics_2006/index-en.html, accessed 9 October 2012).
ARE	United Arab Emirates	2007	2007	20.9	Open Data: Births by nationality, mode of delivery & condition of new born [2007] [website]. Abu Dhabi: Ministry of Health [UAE]; 2008 (www.http://213.42.151.126/Statistics/Statistics_2007/index-en.html, accessed 9 October 2012).
ARE	United Arab Emirates	2013	2013	23.9	Open Data: Births by nationality, mode of delivery & condition of new born [2013] [website]. Abu Dhabi: Ministry of Health [UAE]; 2014 (www.moh.gov.ae/en/OpenData.aspx?Category=Statistics, accessed 15 January 2015).

ISO Code	Country	Coverage start year	Coverage end year	Caesarean section rate (%)	References
GBR	United Kingdom-England	1990	1991	12.4	NHS Maternity Statistics, 2016-17. Hospital Episode Statistics. Table 1.d: Method of delivery [website]. NHS Hospitals, England. Leeds: NHS Digital; 2017 (http://digital.nhs.uk/pubs/maternity1617, accessed 20 December 2017).
GBR	United Kingdom-England	1991	1992	12.9	NHS Maternity Statistics, 2016-17. Hospital Episode Statistics. Table 1.d: Method of delivery [website]. NHS Hospitals, England. Leeds: NHS Digital; 2017 (http://digital.nhs.uk/pubs/maternity1617, accessed 20 December 2017).
GBR	United Kingdom-England	1992	1993	13.7	NHS Maternity Statistics, 2016-17. Hospital Episode Statistics. Table 1.d: Method of delivery [website]. NHS Hospitals, England. Leeds: NHS Digital; 2017 (http://digital.nhs.uk/pubs/maternity1617, accessed 20 December 2017).
GBR	United Kingdom-England	1993	1994	15.0	NHS Maternity Statistics, 2016-17. Hospital Episode Statistics. Table 1.d: Method of delivery [website]. NHS Hospitals, England. Leeds: NHS Digital; 2017 (http://digital.nhs.uk/pubs/maternity1617, accessed 20 December 2017).
GBR	United Kingdom-England	1994	1995	15.5	NHS Maternity Statistics, 2016-17. Hospital Episode Statistics. Table 1.d: Method of delivery [website]. NHS Hospitals, England. Leeds: NHS Digital; 2017 (http://digital.nhs.uk/pubs/maternity1617, accessed 20 December 2017).
GBR	United Kingdom-England	1995	1996	16.4	NHS Maternity Statistics, 2016-17. Hospital Episode Statistics. Table 1.d: Method of delivery [website]. NHS Hospitals, England. Leeds: NHS Digital; 2017 (http://digital.nhs.uk/pubs/maternity1617, accessed 20 December 2017).
GBR	United Kingdom-England	1996	1997	17.0	NHS Maternity Statistics, 2016-17. Hospital Episode Statistics. Table 1.d: Method of delivery [website]. NHS Hospitals, England. Leeds: NHS Digital; 2017 (http://digital.nhs.uk/pubs/maternity1617, accessed 20 December 2017).
GBR	United Kingdom-England	1997	1998	18.3	NHS Maternity Statistics, 2016-17. Hospital Episode Statistics. Table 1.d: Method of delivery [website]. NHS Hospitals, England. Leeds: NHS Digital; 2017 (http://digital.nhs.uk/pubs/maternity1617, accessed 20 December 2017).

ISO Code	Country	Coverage start year	Coverage end year	Caesarean section rate (%)	References
GBR	United Kingdom-England	1998	1999	19.1	NHS Maternity Statistics, 2016-17. Hospital Episode Statistics. Table 1.d: Method of delivery [website]. NHS Hospitals, England. Leeds: NHS Digital; 2017 (http://digital.nhs.uk/pubs/maternity1617, accessed 20 December 2017).
GBR	United Kingdom-England	1999	2000	20.6	NHS Maternity Statistics, 2016-17. Hospital Episode Statistics. Table 1.d: Method of delivery [website]. NHS Hospitals, England. Leeds: NHS Digital; 2017 (http://digital.nhs.uk/pubs/maternity1617, accessed 20 December 2017).
GBR	United Kingdom-England	2000	2001	21.5	NHS Maternity Statistics, 2016-17. Hospital Episode Statistics. Table 1.d: Method of delivery [website]. NHS Hospitals, England. Leeds: NHS Digital; 2017 (http://digital.nhs.uk/pubs/maternity1617, accessed 20 December 2017).
GBR	United Kingdom-England	2001	2002	22.0	NHS Maternity Statistics, 2016-17. Hospital Episode Statistics. Table 1.d: Method of delivery [website]. NHS Hospitals, England. Leeds: NHS Digital; 2017 (http://digital.nhs.uk/pubs/maternity1617, accessed 20 December 2017).
GBR	United Kingdom-England	2002	2003	22.0	NHS Maternity Statistics, 2016-17. Hospital Episode Statistics. Table 1.d: Method of delivery [website]. NHS Hospitals, England. Leeds: NHS Digital; 2017 (http://digital.nhs.uk/pubs/maternity1617, accessed 20 December 2017).
GBR	United Kingdom-England	2003	2004	22.7	NHS Maternity Statistics, 2016-17. Hospital Episode Statistics. Table 1.d: Method of delivery [website]. NHS Hospitals, England. Leeds: NHS Digital; 2017 (http://digital.nhs.uk/pubs/maternity1617, accessed 20 December 2017).
GBR	United Kingdom-England	2004	2005	23.0	NHS Maternity Statistics, 2016-17. Hospital Episode Statistics. Table 1.d: Method of delivery [website]. NHS Hospitals, England. Leeds: NHS Digital; 2017 (http://digital.nhs.uk/pubs/maternity1617, accessed 20 December 2017).
GBR	United Kingdom-England	2005	2006	24.1	NHS Maternity Statistics, 2016-17. Hospital Episode Statistics. Table 1.d: Method of delivery [website]. NHS Hospitals, England. Leeds: NHS Digital; 2017 (http://digital.nhs.uk/pubs/maternity1617, accessed 20 December 2017).

ISO Code	Country	Coverage start year	Coverage end year	Caesarean section rate (%)	References
GBR	United Kingdom-England	2006	2007	24.2	NHS Maternity Statistics, 2016-17. Hospital Episode Statistics. Table 1.d: Method of delivery [website]. NHS Hospitals, England. Leeds: NHS Digital; 2017 (http://digital.nhs.uk/pubs/maternity1617, accessed 20 December 2017).
GBR	United Kingdom-England	2007	2008	24.6	NHS Maternity Statistics, 2016-17. Hospital Episode Statistics. Table 1.d: Method of delivery [website]. NHS Hospitals, England. Leeds: NHS Digital; 2017 (http://digital.nhs.uk/pubs/maternity1617, accessed 20 December 2017).
GBR	United Kingdom-England	2008	2009	24.6	NHS Maternity Statistics, 2016-17. Hospital Episode Statistics. Table 1.d: Method of delivery [website]. NHS Hospitals, England. Leeds: NHS Digital; 2017 (http://digital.nhs.uk/pubs/maternity1617, accessed 20 December 2017).
GBR	United Kingdom-England	2009	2010	24.8	NHS Maternity Statistics, 2016-17. Hospital Episode Statistics. Table 1.d: Method of delivery [website]. NHS Hospitals, England. Leeds: NHS Digital; 2017 (http://digital.nhs.uk/pubs/maternity1617, accessed 20 December 2017).
GBR	United Kingdom-England	2010	2011	24.9	NHS Maternity Statistics, 2016-17. Hospital Episode Statistics. Table 1.d: Method of delivery [website]. NHS Hospitals, England. Leeds: NHS Digital; 2017 (http://digital.nhs.uk/pubs/maternity1617, accessed 20 December 2017).
GBR	United Kingdom-England	2011	2012	25.0	NHS Maternity Statistics, 2016-17. Hospital Episode Statistics. Table 1.d: Method of delivery [website]. NHS Hospitals, England. Leeds: NHS Digital; 2017 (http://digital.nhs.uk/pubs/maternity1617, accessed 20 December 2017).
GBR	United Kingdom-England	2012	2013	25.5	NHS Maternity Statistics, 2016-17. Hospital Episode Statistics. Table 1.d: Method of delivery [website]. NHS Hospitals, England. Leeds: NHS Digital; 2017 (http://digital.nhs.uk/pubs/maternity1617, accessed 20 December 2017).
GBR	United Kingdom-England	2013	2014	26.2	NHS Maternity Statistics, 2016-17. Hospital Episode Statistics. Table 1.d: Method of delivery [website]. NHS Hospitals, England. Leeds: NHS Digital; 2017 (http://digital.nhs.uk/pubs/maternity1617, accessed 20 December 2017).

ISO Code	Country	Coverage start year	Coverage end year	Caesarean section rate (%)	References
GBR	United Kingdom-England	2014	2015	26.5	NHS Maternity Statistics, 2016-17. Hospital Episode Statistics. Table 1.d: Method of delivery [website]. NHS Hospitals, England. Leeds: NHS Digital; 2017 (http://digital.nhs.uk/pubs/maternity1617, accessed 20 December 2017).
GBR	United Kingdom-England	2015	2016	27.1	NHS Maternity Statistics, 2016-17. Hospital Episode Statistics. Table 1.d: Method of delivery [website]. NHS Hospitals, England. Leeds: NHS Digital; 2017 (http://digital.nhs.uk/pubs/maternity1617, accessed 20 December 2017).
GBR	United Kingdom-England	2016	2017	27.8	NHS Maternity Statistics, 2016-17. Hospital Episode Statistics. Table 1.d: Method of delivery [website]. NHS Hospitals, England. Leeds: NHS Digital; 2017 (http://digital.nhs.uk/pubs/maternity1617, accessed 20 December 2017).
GBR	United Kingdom-England	2017	2018	28.4	NHS Maternity Statistics, 2017-18. Summary report 5: Proportion of deliveries by method of delivery and age of mother, 2017-18 (HES) [website]. NHS Hospitals, England. Leeds: NHS Digital; 2018 (https://digital.nhs.uk/data-and-information/publications/statistical/nhs-maternity-statistics/2017-18, accessed 6 March 2020).
GBR	United Kingdom-Northern Ireland	2011	2012	28.4	Northern Ireland Hospital Statistics: Inpatient and Day Case Activity Statistics 2011/12. Belfast: Information & Analysis Directorate, Department of Health, Social Services & Public Safety [Northern Ireland]; 2012.
GBR	United Kingdom-Northern Ireland	2012	2013	29.8	Northern Ireland Hospital Statistics: Inpatient and Day Case Activity Statistics 2012/13. Belfast: Information & Analysis Directorate, Department of Health, Social Services & Public Safety [Northern Ireland]; 2013.
GBR	United Kingdom-Northern Ireland	2013	2014	29.1	Hospital Statistics: Inpatient and Day Case Activity Statistics 2013/14. Belfast: Information & Analysis Directorate, Department of Health, Social Services & Public Safety [Northern Ireland]; 2014.
GBR	United Kingdom-Northern Ireland	2014	2015	29.0	Hospital Statistics: Inpatient and Day Case Activity Statistics 2014/15. Belfast: Information & Analysis Directorate, Department of Health, Social Services & Public Safety [Northern Ireland]; 2015.

ISO Code	Country	Coverage start year	Coverage end year	Caesarean section rate (%)	References
GBR	United Kingdom-Northern Ireland	2015	2016	29.9	Hospital Statistics: Inpatient and Day Case Activity Statistics 2015/16. Belfast: Information & Analysis Directorate, Department of Health (DoH) [Northern Ireland]; 2016.
GBR	United Kingdom-Northern Ireland	2015	2015	30.2	Birth NI: A Survey of Women's Experience of Maternity Care in Northern Ireland. Belfast: School of Nursing and Midwifery, Queen's University of Belfast; 2016.
GBR	United Kingdom-Scotland	1990	1991	14.1	Births in Scottish Hospitals. Year ending 31 March 2017. Table 6.1: Method of delivery in Scotland. Edinburgh and Glasgow: Information Services Division, National Services Scotland (NHS); 2017.
GBR	United Kingdom-Scotland	1991	1992	14.6	Births in Scottish Hospitals. Year ending 31 March 2017. Table 6.1: Method of delivery in Scotland. Edinburgh and Glasgow: Information Services Division, National Services Scotland (NHS); 2017.
GBR	United Kingdom-Scotland	1992	1993	14.6	Births in Scottish Hospitals. Year ending 31 March 2017. Table 6.1: Method of delivery in Scotland. Edinburgh and Glasgow: Information Services Division, National Services Scotland (NHS); 2017.
GBR	United Kingdom-Scotland	1993	1994	15.2	Births in Scottish Hospitals. Year ending 31 March 2017. Table 6.1: Method of delivery in Scotland. Edinburgh and Glasgow: Information Services Division, National Services Scotland (NHS); 2017.
GBR	United Kingdom-Scotland	1994	1995	15.4	Births in Scottish Hospitals. Year ending 31 March 2017. Table 6.1: Method of delivery in Scotland. Edinburgh and Glasgow: Information Services Division, National Services Scotland (NHS); 2017.
GBR	United Kingdom-Scotland	1995	1996	15.9	Births in Scottish Hospitals. Year ending 31 March 2017. Table 6.1: Method of delivery in Scotland. Edinburgh and Glasgow: Information Services Division, National Services Scotland (NHS); 2017.

ISO Code	Country	Coverage start year	Coverage end year	Caesarean section rate (%)	References
GBR	United Kingdom-Scotland	1996	1997	16.0	Births in Scottish Hospitals. Year ending 31 March 2017. Table 6.1: Method of delivery in Scotland. Edinburgh and Glasgow: Information Services Division, National Services Scotland (NHS); 2017.
GBR	United Kingdom-Scotland	1997	1998	16.6	Births in Scottish Hospitals. Year ending 31 March 2017. Table 6.1: Method of delivery in Scotland. Edinburgh and Glasgow: Information Services Division, National Services Scotland (NHS); 2017.
GBR	United Kingdom-Scotland	1998	1999	17.7	Births in Scottish Hospitals. Year ending 31 March 2017. Table 6.1: Method of delivery in Scotland. Edinburgh and Glasgow: Information Services Division, National Services Scotland (NHS); 2017.
GBR	United Kingdom-Scotland	1999	2000	18.9	Births in Scottish Hospitals. Year ending 31 March 2017. Table 6.1: Method of delivery in Scotland. Edinburgh and Glasgow: Information Services Division, National Services Scotland (NHS); 2017.
GBR	United Kingdom-Scotland	2000	2001	19.9	Births in Scottish Hospitals. Year ending 31 March 2017. Table 6.1: Method of delivery in Scotland. Edinburgh and Glasgow: Information Services Division, National Services Scotland (NHS); 2017.
GBR	United Kingdom-Scotland	2001	2002	21.1	Births in Scottish Hospitals. Year ending 31 March 2017. Table 6.1: Method of delivery in Scotland. Edinburgh and Glasgow: Information Services Division, National Services Scotland (NHS); 2017.
GBR	United Kingdom-Scotland	2002	2003	22.4	Births in Scottish Hospitals. Year ending 31 March 2017. Table 6.1: Method of delivery in Scotland. Edinburgh and Glasgow: Information Services Division, National Services Scotland (NHS); 2017.
GBR	United Kingdom-Scotland	2003	2004	23.2	Births in Scottish Hospitals. Year ending 31 March 2017. Table 6.1: Method of delivery in Scotland. Edinburgh and Glasgow: Information Services Division, National Services Scotland (NHS); 2017.

Betran AP, *et al. BMJ Global Health* 2021; 6:e005671. doi: 10.1136/bmjgh-2021-005671

ISO Code	Country	Coverage start year	Coverage end year	Caesarean section rate (%)	References
GBR	United Kingdom-Scotland	2004	2005	23.4	Births in Scottish Hospitals. Year ending 31 March 2017. Table 6.1: Method of delivery in Scotland. Edinburgh and Glasgow: Information Services Division, National Services Scotland (NHS); 2017.
GBR	United Kingdom-Scotland	2005	2006	23.9	Births in Scottish Hospitals. Year ending 31 March 2017. Table 6.1: Method of delivery in Scotland. Edinburgh and Glasgow: Information Services Division, National Services Scotland (NHS); 2017.
GBR	United Kingdom-Scotland	2006	2007	24.5	Births in Scottish Hospitals. Year ending 31 March 2017. Table 6.1: Method of delivery in Scotland. Edinburgh and Glasgow: Information Services Division, National Services Scotland (NHS); 2017.
GBR	United Kingdom-Scotland	2007	2008	24.7	Births in Scottish Hospitals. Year ending 31 March 2017. Table 6.1: Method of delivery in Scotland. Edinburgh and Glasgow: Information Services Division, National Services Scotland (NHS); 2017.
GBR	United Kingdom-Scotland	2008	2009	24.7	Births in Scottish Hospitals. Year ending 31 March 2017. Table 6.1: Method of delivery in Scotland. Edinburgh and Glasgow: Information Services Division, National Services Scotland (NHS); 2017.
GBR	United Kingdom-Scotland	2009	2010	24.9	Births in Scottish Hospitals. Year ending 31 March 2017. Table 6.1: Method of delivery in Scotland. Edinburgh and Glasgow: Information Services Division, National Services Scotland (NHS); 2017.
GBR	United Kingdom-Scotland	2010	2011	25.3	Births in Scottish Hospitals. Year ending 31 March 2017. Table 6.1: Method of delivery in Scotland. Edinburgh and Glasgow: Information Services Division, National Services Scotland (NHS); 2017.
GBR	United Kingdom-Scotland	2011	2012	26.5	Births in Scottish Hospitals. Year ending 31 March 2017. Table 6.1: Method of delivery in Scotland. Edinburgh and Glasgow: Information Services Division, National Services Scotland (NHS); 2017.

Betran AP, et al. BMJ Global Health 2021; 6:e005671. doi: 10.1136/bmjgh-2021-005671

ISO Code	Country	Coverage start year	Coverage end year	Caesarean section rate (%)	References
GBR	United Kingdom-Scotland	2012	2013	27.7	Births in Scottish Hospitals. Year ending 31 March 2017. Table 6.1: Method of delivery in Scotland. Edinburgh and Glasgow: Information Services Division, National Services Scotland (NHS); 2017.
GBR	United Kingdom-Scotland	2013	2014	27.3	Births in Scottish Hospitals. Year ending 31 March 2017. Table 6.1: Method of delivery in Scotland. Edinburgh and Glasgow: Information Services Division, National Services Scotland (NHS); 2017.
GBR	United Kingdom-Scotland	2014	2015	29.2	Births in Scottish Hospitals. Year ending 31 March 2017. Table 6.1: Method of delivery in Scotland. Edinburgh and Glasgow: Information Services Division, National Services Scotland (NHS); 2017.
GBR	United Kingdom-Scotland	2015	2016	30.1	Births in Scottish Hospitals. Year ending 31 March 2017. Table 6.1: Method of delivery in Scotland. Edinburgh and Glasgow: Information Services Division, National Services Scotland (NHS); 2017.
GBR	United Kingdom-Scotland	2016	2017	31.2	Births in Scottish Hospitals. Year ending 31 March 2017. Table 6.1: Method of delivery in Scotland. Edinburgh and Glasgow: Information Services Division, National Services Scotland (NHS); 2017.
GBR	United Kingdom-Scotland	2017	2018	32.5	Births in Scottish Hospitals. Year ending 31 March 2019. Table 6.1: Method of delivery in Scotland. Edinburgh and Glasgow: Information Services Division, National Services Scotland (NHS); 2019.
GBR	United Kingdom-Wales	1995	1996	19.1	Number of deliveries in Wales by delivery method, year and length of stay [online database]. Cardiff: Maternity data, Patient Episode Database for Wales, NHS Wales Informatics Service (NWIS), Welsh Government; 2009 (http://gov.wales/statistics-and-research, accessed 5 February 2018).
GBR	United Kingdom-Wales	1996	1997	19.1	Number of deliveries in Wales by delivery method, year and length of stay [online database]. Cardiff: Maternity data, Patient Episode Database for Wales, NHS Wales Informatics Service (NWIS), Welsh Government; 2009 (http://gov.wales/statistics-and-research, accessed 5 February 2018).

ISO Code	Country	Coverage start year	Coverage end year	Caesarean section rate (%)	References
GBR	United Kingdom-Wales	1997	1998	20.2	Number of deliveries in Wales by delivery method, year and length of stay [online database]. Cardiff: Maternity data, Patient Episode Database for Wales, NHS Wales Informatics Service (NWIS), Welsh Government; 2009 (http://gov.wales/statistics-and-research, accessed 5 February 2018).
GBR	United Kingdom-Wales	1998	1999	21.3	Number of deliveries in Wales by delivery method, year and length of stay [online database]. Cardiff: Maternity data, Patient Episode Database for Wales, NHS Wales Informatics Service (NWIS), Welsh Government; 2009 (http://gov.wales/statistics-and-research, accessed 5 February 2018).
GBR	United Kingdom-Wales	1999	2000	22.9	Number of deliveries in Wales by delivery method, year and length of stay [online database]. Cardiff: Maternity data, Patient Episode Database for Wales, NHS Wales Informatics Service (NWIS), Welsh Government; 2009 (http://gov.wales/statistics-and-research, accessed 5 February 2018).
GBR	United Kingdom-Wales	2000	2001	23.8	Number of deliveries in Wales by delivery method, year and length of stay [online database]. Cardiff: Maternity data, Patient Episode Database for Wales, NHS Wales Informatics Service (NWIS), Welsh Government; 2009 (http://gov.wales/statistics-and-research, accessed 5 February 2018).
GBR	United Kingdom-Wales	2001	2002	24.5	Number of deliveries in Wales by delivery method, year and length of stay [online database]. Cardiff: Maternity data, Patient Episode Database for Wales, NHS Wales Informatics Service (NWIS), Welsh Government; 2009 (http://gov.wales/statistics-and-research, accessed 5 February 2018).
GBR	United Kingdom-Wales	2002	2003	24.4	Number of deliveries in Wales by delivery method, year and length of stay [online database]. Cardiff: Maternity data, Patient Episode Database for Wales, NHS Wales Informatics Service (NWIS), Welsh Government; 2009 (http://gov.wales/statistics-and-research, accessed 5 February 2018).
GBR	United Kingdom-Wales	2003	2004	23.8	Number of deliveries in Wales by delivery method, year and length of stay [online database]. Cardiff: Maternity data, Patient Episode Database for Wales, NHS Wales Informatics Service (NWIS), Welsh Government; 2009 (http://gov.wales/statistics-and-research, accessed 5 February 2018).
GBR	United Kingdom-Wales	2004	2005	24.5	Number of deliveries in Wales by delivery method, year and length of stay [online database]. Cardiff: Maternity data, Patient Episode Database for Wales, NHS Wales Informatics Service (NWIS), Welsh Government; 2009 (http://gov.wales/statistics-and-research, accessed 5 February 2018).

ISO Code	Country	Coverage start year	Coverage end year	Caesarean section rate (%)	References
GBR	United Kingdom-Wales	2005	2006	26.2	Number of deliveries in Wales by delivery method, year and length of stay [online database]. Cardiff: Maternity data, Patient Episode Database for Wales, NHS Wales Informatics Service (NWIS), Welsh Government; 2009 (http://gov.wales/statistics-and-research, accessed 5 February 2018).
GBR	United Kingdom-Wales	2006	2007	26.9	Number of deliveries in Wales by delivery method, year and length of stay [online database]. Cardiff: Maternity data, Patient Episode Database for Wales, NHS Wales Informatics Service (NWIS), Welsh Government; 2009 (http://gov.wales/statistics-and-research, accessed 5 February 2018).
GBR	United Kingdom-Wales	2007	2008	26.1	Number of deliveries in Wales by delivery method, year and length of stay [online database]. Cardiff: Maternity data, Patient Episode Database for Wales, NHS Wales Informatics Service (NWIS), Welsh Government; 2009 (http://gov.wales/statistics-and-research, accessed 5 February 2018).
GBR	United Kingdom-Wales	2008	2009	26.6	Maternity Statistics, Wales: Method of Delivery, 2000-2010. Cardiff: Statistical Directorate, Welsh Assembly Government; 2011.
GBR	United Kingdom-Wales	2009	2010	26.6	Maternity data, Patient Episode Database for Wales: Delivery by type in each LHB in Wales [online database]. Cardiff: Maternity data, Patient Episode Database for Wales, NHS Wales Informatics Service (NWIS), Welsh Government; 2015 (http://gov.wales/statistics-and-research/maternity-method-delivery/?lang=en, accessed 5 February 2018).
GBR	United Kingdom-Wales	2010	2011	26.0	Maternity data, Patient Episode Database for Wales: Delivery by type in each LHB in Wales [online database]. Cardiff: Maternity data, Patient Episode Database for Wales, NHS Wales Informatics Service (NWIS), Welsh Government; 2015 (http://gov.wales/statistics-and-research/maternity-method-delivery/?lang=en, accessed 5 February 2018).
GBR	United Kingdom-Wales	2011	2012	25.7	Maternity data, Patient Episode Database for Wales: Delivery by type in each LHB in Wales [online database]. Cardiff: Maternity data, Patient Episode Database for Wales, NHS Wales Informatics Service (NWIS), Welsh Government; 2015 (http://gov.wales/statistics-and-research/maternity-method-delivery/?lang=en, accessed 5 February 2018).
GBR	United Kingdom-Wales	2012	2013	27.5	Maternity data, Patient Episode Database for Wales: Delivery by type in each LHB in Wales [online database]. Cardiff: Maternity data, Patient Episode Database for Wales, NHS Wales Informatics Service (NWIS), Welsh Government; 2015 (http://gov.wales/statistics-and-research/maternity-method-delivery/?lang=en, accessed 5 February 2018).

ISO Code	Country	Coverage start year	Coverage end year	Caesarean section rate (%)	References
GBR	United Kingdom-Wales	2013	2014	26.9	Maternity data, Patient Episode Database for Wales: Delivery by type in each LHB in Wales [online database]. Cardiff: Maternity data, Patient Episode Database for Wales, NHS Wales Informatics Service (NWIS), Welsh Government; 2015 (http://gov.wales/statistics-and-research/maternity-method-delivery/?lang=en, accessed 5 February 2018).
GBR	United Kingdom-Wales	2014	2015	26.3	Maternity data, Patient Episode Database for Wales: Delivery by type in each LHB in Wales [online database]. Cardiff: Maternity data, Patient Episode Database for Wales, NHS Wales Informatics Service (NWIS), Welsh Government; 2015 (http://gov.wales/statistics-and-research/maternity-method-delivery/?lang=en, accessed 5 February 2018).
GBR	United Kingdom-Wales	2015	2016	25.6	Maternity Statistics, Wales, 2015-16 (Experimental Statistics). Wales: Statistics for Wales, Welsh Government; 2017.
TZA	United Republic of Tanzania	1991	1996	2.1	Bureau of Statistics [Tanzania], Macro International Inc. Tanzania Demographic and Health Survey 1996. Calverton, Maryland: Bureau of Statistics, Macro International; 1997.
TZA	United Republic of Tanzania	1994	1999	2.9	National Bureau of Statistics [Tanzania], Macro International Inc. Tanzania Reproductive and Child Health Survey 1999. Calverton, Maryland, USA: National Bureau of Statistics, Macro International Inc.; 2000.
TZA	United Republic of Tanzania	1999	2005	3.2	National Bureau of Statistics (NBS) [Tanzania], ORC Macro. Tanzania Demographic and Health Survey 2004-05. Dar es Salaam, Tanzania: National Bureau of Statistics, ORC Macro; 2005.
TZA	United Republic of Tanzania	2005	2010	4.5	National Bureau of Statistics (NBS) [Tanzania], ICF Macro. Tanzania Demographic and Health Survey 2010. Dar es Salaam, Tanzania: NBS, ICF Macro; 2011.
TZA	United Republic of Tanzania	2010	2016	5.9	Ministry of Health, Community Development, Gender, Elderly and Children (MoHCDGEC) [Tanzania], Ministry of Health (MoH) [Zanzibar], National Bureau of Statistics (NBS) [Tanzania], Office of Chief Government Statistician (OCGS) [Zanzibar], ICF. Tanzania Demographic and Health Survey and Malaria Indicator Survey (TDHS-MIS) 2015-16. Dar es Salaam, Tanzania: MoHCDGEC, MoH, NBS, OCGS, ICF; 2016.

ISO Code	Country	Coverage start year	Coverage end year	Caesarean section rate (%)	References
USA	United States of America	1990	1990	22.7	Martin JA, Hamilton BE, Sutton PD, Ventura SJ, Osterman MJK, Mathews TJ. Births: Final Data for 2008. National Vital Statistics Reports; vol. 59, no.1. Hyattsville, MD: National Center for Health Statistics; 2010.
USA	United States of America	1991	1991	22.6	Martin JA, Hamilton BE, Sutton PD, Ventura SJ, Osterman MJK, Mathews TJ. Births: Final Data for 2008. National Vital Statistics Reports; vol. 59, no.1. Hyattsville, MD: National Center for Health Statistics; 2010.
USA	United States of America	1992	1992	22.3	Martin JA, Hamilton BE, Sutton PD, Ventura SJ, Osterman MJK, Mathews TJ. Births: Final Data for 2008. National Vital Statistics Reports; vol. 59, no.1. Hyattsville, MD: National Center for Health Statistics; 2010.
USA	United States of America	1993	1993	21.8	Martin JA, Hamilton BE, Sutton PD, Ventura SJ, Osterman MJK, Mathews TJ. Births: Final Data for 2008. National Vital Statistics Reports; vol. 59, no.1. Hyattsville, MD: National Center for Health Statistics; 2010.
USA	United States of America	1994	1994	21.2	Martin JA, Hamilton BE, Sutton PD, Ventura SJ, Osterman MJK, Mathews TJ. Births: Final Data for 2008. National Vital Statistics Reports; vol. 59, no.1. Hyattsville, MD: National Center for Health Statistics; 2010.
USA	United States of America	1995	1995	20.8	Martin JA, Hamilton BE, Sutton PD, Ventura SJ, Osterman MJK, Mathews TJ. Births: Final Data for 2008. National Vital Statistics Reports; vol. 59, no.1. Hyattsville, MD: National Center for Health Statistics; 2010.
USA	United States of America	1996	1996	20.7	Martin JA, Hamilton BE, Sutton PD, Ventura SJ, Osterman MJK, Mathews TJ. Births: Final Data for 2008. National Vital Statistics Reports; vol. 59, no.1. Hyattsville, MD: National Center for Health Statistics; 2010.
USA	United States of America	1997	1997	20.8	Ventura SJ, Martin JA, Curtin SC, Mathews TJ. Births: Final Data for 1997. National Vital Statistics Reports; vol. 47, no. 18. Hyattsville, Maryland: National Center for Health Statistics; 1999.

ISO Code	Country	Coverage start year	Coverage end year	Caesarean section rate (%)	References
USA	United States of America	1998	1998	21.2	Ventura SJ, Martin JA, Curtin SC, Mathews TJ, Park MM. Births: Final data for 1998. National vital statistics reports; vol. 48, no. 3. Hyattsville, Maryland: National Center for Health Statistics; 2000.
USA	United States of America	1999	1999	22.0	Ventura SJ, Martin JA, Curtin SC, Menacker F, Hamilton BE. Births: Final data for 1999. National Vital Statistics Reports; vol. 49, no. 1. Hyattsville, Maryland: National Center for Health Statistics; 2001.
USA	United States of America	2000	2000	22.9	Martin JA, Hamilton BE, Ventura SJ, Menacker F, Park MM. Births: Final data for 2000. National Vital Statistics Reports; vol. 50, no. 5. Hyattsville, Maryland: National Center for Health Statistics; 2002.
USA	United States of America	2001	2001	24.4	Martin JA, Hamilton BE, Ventura SJ, Menacker F, Park MM, Sutton PD. Births: Final data for 2001. National Vital Statistics Reports; vol. 51, no. 2. Hyattsville, Maryland: National Center for Health Statistics; 2002.
USA	United States of America	2002	2002	26.1	Martin JA, Hamilton BE, Sutton PD, Ventura SJ, Menacker F, Munson ML. Births: Final data for 2002. National Vital Statistics Reports; vol. 52, no. 10. Hyattsville, Maryland: National Center for Health Statistics; 2003.
USA	United States of America	2003	2003	27.5	Martin JA, Hamilton BE, Sutton PD, Ventura SJ, Menacker F, Munson ML. Births: Final data for 2003. National Vital Statistics Reports; vol. 54, no. 2. Hyattsville, MD: National Center for Health Statistics; 2005.
USA	United States of America	2004	2004	29.1	Martin JA, Hamilton BE, Sutton PD, Ventura SJ, Menacker F, Kirmeyer S. Births: Final data for 2004. National Vital Statistics Reports; vol. 55, no. 1. Hyattsville, MD: National Center for Health Statistics; 2006.
USA	United States of America	2005	2005	30.3	Martin JA, Hamilton BE, Sutton PD, Ventura SJ, Menacker F, Kirmeyer S et al. Births: Final data for 2005. National Vital Statistics Reports; vol. 56, no. 6. Hyattsville, MD: National Center for Health Statistics; 2007.

ISO Code	Country	Coverage start year	Coverage end year	Caesarean section rate (%)	References
USA	United States of America	2006	2006	31.1	Martin JA, Hamilton BE, Sutton PD, Ventura SJ, Menacker F, Kirmeyer S et al. Births: Final data for 2006. National Vital Statistics Reports; vol.57, no. 7. Hyattsville, MD: National Center for Health Statistics; 2009.
USA	United States of America	2007	2007	31.8	Martin JA, Hamilton BE, Sutton PD, Ventura SJ, Mathews TJ, Kirmeyer S et al. Births: Final Data for 2007. National Vital Statistics Reports; vol. 58, no.24. Hyattsville, MD: National Center for Health Statistics; 2010.
USA	United States of America	2008	2008	32.3	Martin JA, Hamilton BE, Sutton PD, Ventura SJ, Mathews TJ, Osterman MJK. Births: Final Data for 2008. National Vital Statistics Reports; vol. 59, no.1. Hyattsville, MD: National Center for Health Statistics; 2010.
USA	United States of America	2009	2009	32.9	Martin JA, Hamilton BE, Ventura SJ, Osterman MJK, Kirmeyer S, Mathews TJ et al. Births: Final Data for 2009. National Vital Statistics Reports; vol. 60, no.1. Hyattsville, MD: National Center for Health Statistics; 2011.
USA	United States of America	2010	2010	32.8	Martin JA, Hamilton BE, Ventura SJ, Osterman MJK, Wilson EC, Mathews TJ. Births: Final Data for 2010. National Vital Statistics Reports; vol. 61, no. 1. Hyattsville, MD: National Center for Health Statistics; 2012.
USA	United States of America	2011	2011	32.8	Martin JA, Hamilton BE, Ventura SJ, Osterman MJK, Mathews TJ. Births: Final data for 2011. National Vital Statistics Reports; vol 62, no 1. Hyattsville, MD: National Center for Health Statistics; 2013.
USA	United States of America	2012	2012	32.8	Martin JA, Hamilton BE, Osterman MJK, Curtin SC, Mathews TJ. Births: Final data for 2012. National Vital Statistics Reports; vol 62, no 9. Hyattsville, MD: National Center for Health Statistics; 2013.
USA	United States of America	2013	2013	32.7	Martin JA, Hamilton BE, Osterman MJK, Curtin SC, Mathews TJ. Births: Final data for 2013. National Vital Statistics Reports; vol 64, no 1. Hyattsville, MD: National Center for Health Statistics; 2015.

ISO Code	Country	Coverage start year	Coverage end year	Caesarean section rate (%)	References
USA	United States of America	2014	2014	32.2	Hamilton BE, Martin JA, Osterman MJK, Curtin SC, Mathews TJ. Births: Final Data for 2014. National Vital Statistics Reports; vol 64, no 12. Hyattsville, MD: National Center for Health Statistics; 2015.
USA	United States of America	2015	2015	32.0	Martin JA, Hamilton BE, Osterman MJK, Driscoll AK, Mathews TJ. Births: Final Data for 2015. National Vital Statistics Reports; vol 66, no 1. Hyattsville, MD: National Center for Health Statistics; 2017.
USA	United States of America	2016	2016	31.9	Martin JA, Hamilton BE, Osterman MJK, Driscoll AK, Drake P. Births: Final Data for 2016. National Vital Statistics Reports; vol 67, no 1. Hyattsville, MD: National Center for Health Statistics; 2018.
USA	United States of America	2017	2017	32.0	Martin JA, Hamilton BE, Osterman MJK, Driscoll AK, Drake P. Births: Final Data for 2017. National Vital Statistics Reports; vol 67, no 8. Hyattsville, MD: National Center for Health Statistics; 2018.
USA	United States of America	2018	2018	31.9	Martin JA, Hamilton BE, Osterman MJK, Driscoll AK. Births: Final Data for 2018. National Vital Statistics Reports; vol 68, no 13. Hyattsville, MD: National Center for Health Statistics; 2019.
URY	Uruguay	1993	1993	23.9	Cuadros Estadísticos. Montevideo: Instituto Nacional de Estadística (INE); 2001 (in Spanish).
URY	Uruguay	1996	1996	22.0	Estadísticas Vitales. Natalidad 1996. Forma de terminación del embarazo [website]. Montevideo: Ministerio de Salud Pública [Ministry of Public Health, Uruguay]; 1997 (http://www.msp.gub.uy/subcategorias_19_1.html, accessed 10 October 2012, in Spanish).
URY	Uruguay	1997	1997	21.6	Estadísticas Vitales. Natalidad 1997. Forma de terminación del embarazo [website]. Montevideo: Ministerio de Salud Pública [Ministry of Public Health, Uruguay]; 1998 (http://www.msp.gub.uy/subcategorias_19_1.html, accessed 10 October 2012, in Spanish).

ISO Code	Country	Coverage start year	Coverage end year	Caesarean section rate (%)	References
URY	Uruguay	1999	1999	22.4	Estadísticas Vitales. Natalidad 1999. Forma de terminación del embarazo [website]. Montevideo: Ministerio de Salud Pública [Ministry of Public Health, Uruguay]; 2000 (http://www.msp.gub.uy/subcategorias_19_1.html, accessed 10 October 2012, in Spanish).
URY	Uruguay	2000	2000	23.1	Estadísticas Vitales. Natalidad 2000. Forma de terminación del embarazo [website]. Montevideo: Ministerio de Salud Pública [Ministry of Public Health, Uruguay]; 2001 (http://www.msp.gub.uy/subcategorias_19_1.html, accessed 10 October 2012, in Spanish).
URY	Uruguay	2001	2001	25.7	Estadísticas Vitales. Natalidad 2001. Forma de terminación del embarazo [website]. Montevideo: Ministerio de Salud Pública [Ministry of Public Health, Uruguay]; 2002 (http://www.msp.gub.uy/subcategorias_19_1.html, accessed 10 October 2012, in Spanish).
URY	Uruguay	2002	2002	25.8	Estadísticas Vitales. Natalidad 2002. Forma de terminación del embarazo [website]. Montevideo: Ministerio de Salud Pública [Ministry of Public Health, Uruguay]; 2003 (http://www.msp.gub.uy/subcategorias_19_1.html, accessed 10 October 2012, in Spanish).
URY	Uruguay	2003	2003	26.6	Estadísticas Vitales. Natalidad 2003. Forma de terminación del embarazo [website]. Montevideo: Ministerio de Salud Pública [Ministry of Public Health, Uruguay]; 2004 (http://www.msp.gub.uy/subcategorias_19_1.html, accessed 10 October 2012, in Spanish).
URY	Uruguay	2004	2004	28.9	Estadísticas Vitales. Natalidad 2004. Forma de terminación del embarazo [website]. Montevideo: Ministerio de Salud Pública [Ministry of Public Health, Uruguay]; 2005 (http://www.msp.gub.uy/subcategorias_19_1.html, accessed 10 October 2012, in Spanish).
URY	Uruguay	2005	2005	31.0	Estadísticas Vitales. Natalidad 2005. Forma de terminación del embarazo [website]. Montevideo: Ministerio de Salud Pública [Ministry of Public Health, Uruguay]; 2006 (http://www.msp.gub.uy/subcategorias_19_1.html, accessed 10 October 2012, in Spanish).
URY	Uruguay	2006	2006	31.2	Estadísticas Vitales. Natalidad 2006. Forma de terminación del embarazo [website]. Montevideo: Ministerio de Salud Pública [Ministry of Public Health, Uruguay]; 2007 (http://www.msp.gub.uy/subcategorias_19_1.html, accessed 10 October 2012, in Spanish).

ISO Code	Country	Coverage start year	Coverage end year	Caesarean section rate (%)	References
URY	Uruguay	2007	2007	33.0	Estadísticas Vitales. Natalidad 2007. Forma de terminación del embarazo [website]. Montevideo: Ministerio de Salud Pública [Ministry of Public Health, Uruguay]; 2008 (http://www.msp.gub.uy/subcategorias_19_1.html, accessed 10 October 2012, in Spanish).
URY	Uruguay	2011	2011	40.1	República Oriental del Uruguay - Estadísticas Vitales - Natalidad 2011-2015, Certificado de Nacido Vivo de Uruguay. Natalidad 2011_p: Tipo de cesárea (tipoces) [website]. Montevideo: Ministerio de Salud Pública [Ministry of Public Health, Uruguay]; 2016 (http://estadisticas.msp.gub.uy/index.php/catalog/20/data_dictionary#page=F10&tab=data-dictionary, accessed 4 August 2016, in Spanish).
URY	Uruguay	2012	2012	39.9	Estadísticas Vitales. Nacimientos ocurridos y registrados según tipo de parto y grupo de edad materna. Uruguay 1996-2012. Montevideo: Ministerio de Salud Pública [Ministry of Public Health, Uruguay]; 2014 (in Spanish).
URY	Uruguay	2013	2013	41.0	República Oriental del Uruguay - Estadísticas Vitales - Natalidad 2011-2015, Certificado de Nacido Vivo de Uruguay. Natalidad 2013_p: Tipo de cesárea (tipoces) [website]. Montevideo: Ministerio de Salud Pública [Ministry of Public Health, Uruguay]; 2016 (http://estadisticas.msp.gub.uy/index.php/catalog/20/data_dictionary#page=F14&tab=data-dictionary, accessed 4 August 2016, in Spanish).
URY	Uruguay	2014	2014	39.5	República Oriental del Uruguay - Estadísticas Vitales - Natalidad 2011-2015, Certificado de Nacido Vivo de Uruguay. Natalidad 2014_p: Tipo de cesárea (tipoces) [website]. Montevideo: Ministerio de Salud Pública [Ministry of Public Health, Uruguay]; 2016 (http://estadisticas.msp.gub.uy/index.php/catalog/20/data_dictionary#page=F16&tab=data-dictionary, accessed 4 August 2016, in Spanish).
URY	Uruguay	2015	2015	39.4	República Oriental del Uruguay - Estadísticas Vitales - Natalidad 2011-2015, Certificado de Nacido Vivo de Uruguay. Natalidad 2015_p: Tipo de cesárea (tipoces) [website]. Montevideo: Ministerio de Salud Pública [Ministry of Public Health, Uruguay]; 2016 (http://estadisticas.msp.gub.uy/index.php/catalog/20/data_dictionary#page=F9&tab=data-dictionary, accessed 4 August 2016, in Spanish).
UZB	Uzbekistan	1993	1996	3.0	Institute of Obstetrics and Gynecology Ministry of Health [Uzbekistan], Macro International Inc. Uzbekistan Demographic and Health Survey, 1996. Calverton, Maryland: Macro International Inc.; 1997.
UZB	Uzbekistan	1996	1996	2.2	European Health for All Database (HFA-DB) [online database]. World Health Organization (WHO) Regional Office for Europe; 2012 (http://data.euro.who.int/hfadb, accessed 20 August 2012).

ISO Code	Country	Coverage start year	Coverage end year	Caesarean section rate (%)	References
UZB	Uzbekistan	1997	1997	2.3	European Health for All Database (HFA-DB) [online database]. World Health Organization (WHO) Regional Office for Europe; 2012 (http://data.euro.who.int/hfadb, accessed 20 August 2012).
UZB	Uzbekistan	1998	1998	2.4	European Health for All Database (HFA-DB) [online database]. World Health Organization (WHO) Regional Office for Europe; 2012 (http://data.euro.who.int/hfadb, accessed 20 August 2012).
UZB	Uzbekistan	1999	1999	2.7	European Health for All Database (HFA-DB) [online database]. World Health Organization (WHO) Regional Office for Europe; 2012 (http://data.euro.who.int/hfadb, accessed 20 August 2012).
UZB	Uzbekistan	2000	2000	3.1	European Health for All Database (HFA-DB) [online database]. World Health Organization (WHO) Regional Office for Europe; 2012 (http://data.euro.who.int/hfadb, accessed 20 August 2012).
UZB	Uzbekistan	2001	2001	3.3	European Health for All Database (HFA-DB) [online database]. World Health Organization (WHO) Regional Office for Europe; 2012 (http://data.euro.who.int/hfadb, accessed 20 August 2012).
UZB	Uzbekistan	2002	2002	3.6	European Health for All Database (HFA-DB) [online database]. World Health Organization (WHO) Regional Office for Europe; 2012 (http://data.euro.who.int/hfadb, accessed 20 August 2012).
UZB	Uzbekistan	2003	2003	3.8	European Health for All Database (HFA-DB) [online database]. World Health Organization (WHO) Regional Office for Europe; 2012 (http://data.euro.who.int/hfadb, accessed 20 August 2012).
UZB	Uzbekistan	2004	2004	4.4	European Health for All Database (HFA-DB) [online database]. World Health Organization (WHO) Regional Office for Europe; 2012 (http://data.euro.who.int/hfadb, accessed 20 August 2012).

ISO Code	Country	Coverage start year	Coverage end year	Caesarean section rate (%)	References
UZB	Uzbekistan	2005	2005	4.9	European Health for All Database (HFA-DB) [online database]. World Health Organization (WHO) Regional Office for Europe; 2012 (http://data.euro.who.int/hfadb, accessed 20 August 2012).
UZB	Uzbekistan	2006	2006	6.1	European Health for All Database (HFA-DB) [online database]. World Health Organization (WHO) Regional Office for Europe; 2012 (http://data.euro.who.int/hfadb, accessed 20 August 2012).
UZB	Uzbekistan	2007	2007	6.3	European Health for All Database (HFA-DB) [online database]. World Health Organization (WHO) Regional Office for Europe; 2012 (http://data.euro.who.int/hfadb, accessed 20 August 2012).
UZB	Uzbekistan	2008	2008	6.7	European Health for All Database (HFA-DB) [online database]. World Health Organization (WHO) Regional Office for Europe; 2012 (http://data.euro.who.int/hfadb, accessed 20 August 2012).
UZB	Uzbekistan	2009	2009	7.7	European Health for All Database (HFA-DB) [online database]. World Health Organization (WHO) Regional Office for Europe; 2012 (http://data.euro.who.int/hfadb, accessed 20 August 2012).
UZB	Uzbekistan	2010	2010	8.6	European Health for All Database (HFA-DB) [online database]. World Health Organization (WHO) Regional Office for Europe; 2016 (http://data.euro.who.int/hfadb, accessed 8 August 2016).
UZB	Uzbekistan	2011	2011	9.0	European Health for All Database (HFA-DB) [online database]. World Health Organization (WHO) Regional Office for Europe; 2016 (http://data.euro.who.int/hfadb, accessed 8 August 2016).
UZB	Uzbekistan	2012	2012	10.5	European Health for All Database (HFA-DB) [online database]. World Health Organization (WHO) Regional Office for Europe; 2016 (http://data.euro.who.int/hfadb, accessed 8 August 2016).

ISO Code	Country	Coverage start year	Coverage end year	Caesarean section rate (%)	References
UZB	Uzbekistan	2013	2013	11.1	European Health for All Database (HFA-DB) [online database]. World Health Organization (WHO) Regional Office for Europe; 2016 (http://data.euro.who.int/hfadb, accessed 8 August 2016).
UZB	Uzbekistan	2014	2014	11.8	European Health for All Database (HFA-DB) [online database]. World Health Organization (WHO) Regional Office for Europe; 2016 (http://data.euro.who.int/hfadb, accessed 8 August 2016).
UZB	Uzbekistan	2015	2015	13.6	UNICEF CRING: Uzbekistan; 2015.
VUT	Vanuatu	2008	2013	11.9	Vanuatu Ministry of Health, Vanuatu National Statistics Office, Secretariat of the Pacific Community (SPC). Vanuatu Demographic and Health Survey 2013. Final Report. Noumea: SPC; 2014.
VEN	Venezuela (Bolivarian Republic of)	2009	2009	32.2	Anuario Estadístico 2009. Indicadores de redes de servicios y programas de salud. Caracas: Ministerio del Poder Popular para la Salud (MPPS) [Venezuela]; 2010 (in Spanish).
VNM	Viet Nam	1994	1997	3.4	National Committee for Population and Family Planning. Viet Nam Demographic and Health Survey 1997. Ha Noi, Viet Nam: National Committee for Population and Family Planning; 1999.
VNM	Viet Nam	1999	2002	9.9	Committee for Population, Family and Children [Vietnam], ORC Macro. Vietnam Demographic and Health Survey 2002. Calverton, Maryland, USA: Committee for Population, Family and Children, ORC Macro; 2003.
VNM	Viet Nam	2009	2011	20.0	General Statistics Office (GSO), United Nations Children's Fund (UNICEF), United Nations Population Fund (UNFPA). Viet Nam Multiple Indicator Cluster Survey - Final Report, 2011. Ha Noi: General Statistical Office (GSO), UNICEF, UNFPA; 2011.

ISO Code	Country	Coverage start year	Coverage end year	Caesarean section rate (%)	References
VNM	Viet Nam	2011	2014	27.5	General Statistics Office (GSO), United Nations Children's Fund (UNICEF). Viet Nam Multiple Indicator Cluster Survey 2014, Final Report. Ha Noi: General Statistics Office, UNICEF; 2015.
YEM	Yemen	1992	1997	1.4	Central Statistical Organization (CSO) [Yemen], Macro International Inc. (MI). Yemen Demographic and Maternal and Child Health Survey 1997. Calverton, Maryland, USA: CSO, MI; 1998.
YEM	Yemen	1998	2003	8.6	The Yemen Family Health Survey: Principal Report. Cairo: Ministry of Health and Population [Yemen], Central Statistical Organization [Yemen], League of Arab States; 2004.
YEM	Yemen	2009	2009	6.5	Annual Statistical Health Report 2009. Sana'a: Ministry of Health and Population [Yemen], 2010 (in Arabic).
YEM	Yemen	2008	2013	4.8	Ministry of Public Health and Population (MOPHP) [Yemen], Central Statistical Organization (CSO) [Yemen], Pan Arab Program for Family Health (PAPFAM), ICF International. Yemen National Health and Demographic Survey 2013. Rockville, Maryland, USA: MOPHP, CSO, PAPFAM, ICF International; 2015.
ZMB	Zambia	1987	1992	2.6	Gaisie K, Cross AR, Nsemukila G (University of Zambia, Central Statistical Office [Zambia], Macro International). Zambia Demographic and Health Survey 1992. Columbia, Maryland, USA: University of Zambia, Central Statistical Office [Zambia], Macro International; 1992.
ZMB	Zambia	1991	1996	1.9	Central Statistical Office Ministry of Health [Zambia], Macro International Inc. Zambia Demographic and Health Survey, 1996. Calverton, Maryland: Central Statistical Office [Zambia], Macro International Inc.; 1997.
ZMB	Zambia	1996	2002	2.1	Central Statistical Office [Zambia], Central Board of Health [Zambia], ORC Macro. Zambia Demographic and Health Survey 2001-2002. Calverton, Maryland, USA: Central Statistical Office [Zambia], Central Board of Health [Zambia], ORC Macro; 2003.

ISO Code	Country	Coverage start year	Coverage end year	Caesarean section rate (%)	References
ZMB	Zambia	2003	2003	6.9	Annual Health Statistical Bulletin 2003. Lusaka: Central Board of Health [Zambia]; 2004.
ZMB	Zambia	2002	2007	3.0	Central Statistical Office (CSO), Ministry of Health (MOH), Tropical Diseases Research Centre (TDRC), University of Zambia, Macro International Inc. Zambia Demographic and Health Survey 2007. Calverton, Maryland, USA: CSO, Macro International Inc.; 2009.
ZMB	Zambia	2008	2014	4.4	Central Statistical Office (CSO) [Zambia], Ministry of Health (MOH) [Zambia], ICF International. Zambia Demographic and Health Survey 2013-14. Rockville, Maryland, USA: Central Statistical Office, Ministry of Health, ICF International; 2015.
ZMB	Zambia	2014	2019	5.0	Zambia Demographic and Health Survey 2018. Lusaka, Rockville, Maryland, USA: Zambia Statistics Agency, Ministry of Health (MOH) [Zambia], ICF; 2019.
ZWE	Zimbabwe	1989	1994	6.0	Central Statistical Office [Zimbabwe], Macro International Inc. Zimbabwe Demographic and Health Survey, 1994. Calverton, Maryland, USA: Central Statistical Office [Zimbabwe], Macro International Inc.; 1995.
ZWE	Zimbabwe	1994	1999	6.7	Central Statistical Office [Zimbabwe], Macro International Inc. Zimbabwe Demographic and Health Survey 1999. Calverton, Maryland, USA: Central Statistical Office [Zimbabwe], Macro International Inc.; 2000.
ZWE	Zimbabwe	2000	2006	4.8	Central Statistical Office (CSO) [Zimbabwe], Macro International Inc. Zimbabwe Demographic and Health Survey 2005-06. Calverton, Maryland, USA: CSO [Zimbabwe], Macro International Inc.; 2007.
ZWE	Zimbabwe	2005	2011	4.5	Zimbabwe National Statistics Agency (ZIMSTAT), ICF International. Zimbabwe Demographic and Health Survey 2010-11. Calverton, Maryland: ZIMSTAT, ICF International Inc.; 2012.

ISO Code	Country	Coverage start year	Coverage end year	Caesarean section rate (%)	References
ZWE	Zimbabwe	2010	2015	5.8	Zimbabwe National Statistics Agency, ICF International. Zimbabwe Demographic and Health Survey 2015: Final Report. Rockville, Maryland, USA: Zimbabwe National Statistics Agency (ZIMSTAT), ICF International; 2016.
ZWE	Zimbabwe	2017	2019	9.0	Zimbabwe Multiple Indicator Cluster Survey 2019, Snapshots of Key Findings. Harare: Zimbabwe National Statistics Agency (ZIMSTAT), United Nations Children's Fund (UNICEF); 2019.
